# Red-Shifted Glycoporphyrins: Synthesis via Sonogashira Cross-Coupling and Studies on Reactive Oxygen Species Production

**DOI:** 10.1021/acs.orglett.5c02038

**Published:** 2025-07-14

**Authors:** Dariusz Baran, Maciej Malinowski

**Affiliations:** 201870 Warsaw University of Technology, Faculty of Chemistry, ul. Noakowskiego 3, 00-664 Warsaw, Poland

## Abstract

Glycoporphyrins are in the spotlight as third-generation photosensitizers. Herein, we present a new Sonogashira-based methodology toward C–C-bonded, diverse carbohydrate–porphyrin hybrids. The sugar unit is for the first time effectively conjugated with a porphyrin system, resulting in a bathochromic shift of absorption maxima. The products generate reactive oxygen species with the use of 690–740 nm visible light lamps, making them promising photosensitizers in phototherapies.

## Graphical abstract

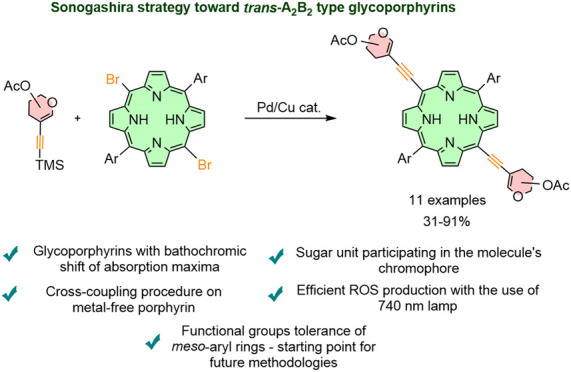

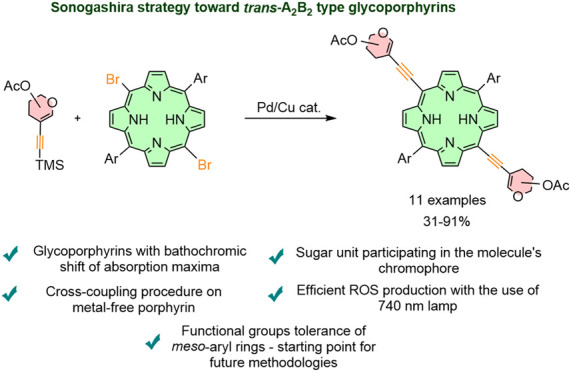

Alternative anticancer treatments are gaining momentum in modern studies. In particular, light-guided therapies such as PDT (photodynamic therapy) or PTT (photothermal therapy) are expanding their audience since they offer a great possibility of cancer elimination with minimal (if any) side effects during therapy.^1,2^ Successful PDT relies on the selection of the proper photosensitizer (PS) that upon irradiation at a specific wavelength transforms local oxygen into reactive oxygen species (ROS). Therefore, modern studies pursue efficient PS that better meets the requirements of PDT.^3,4^ Among the scaffolds, the family of porphyrinoids is one of the most exploited as PS, due to their high efficiency of ROS production and strong absorption in the visible light spectrum. Nevertheless, there are still some aspects that have to be improved to call porphyrin-based PS the future of phototherapy. First, synthetic porphyrins rarely solubilize in a physiological milieu, and they exhibit no directing abilities toward cancer cells.

Interestingly, glycoporphyrins (carbohydrate–porphyrin conjugates) are a subgroup of PS that perfectly meet the aforementioned criteria. A sugar moiety might be used as a Trojan Horse, due to its affinity for cancer lectins, so PS benefits from gaining targeting abilities toward cancer cells.^5–7^ Furthermore, hydrophilic saccharide units improve the solubility of the whole system in polar solvents. These findings have created impactful interest in glycoporphyrins as a third generation of PS.^8,9^ However, the development of a versatile synthetic methodology toward sugar–porphyrin hybrids remains a challenging task due to the distinct chemical nature of both subunits. Therefore, new protocols are regularly introduced to overcome the limitations.^10–15^ Nevertheless, there is still one parameter that has not been explored with the use of sugar–porphyrin PS until now. It is sugar-influenced modification of the porphyrin UV–vis absorption profile. It is highly convenient if PS absorbs lower-energy and more penetrating red-shifted wavelengths.

Unfunctionalized, synthetic porphyrins usually lack significant absorption above 645 nm (the last Q band, the most promising for use in PDT). Some of the less accessible porphyrinoids (chlorins or bacteriochlorins) are characterized by a more promising UV–vis profile, and new methodologies of glycosylation are being introduced in their chemistry.^16,17^ In the case of glycoporphyrin chemistry, to the best of our knowledge, the subject of a bathochromic shift introduced by the sugar moiety has not been yet recognized. Herein, we present our studies on the synthesis of C–C-conjugated glycoporphyrins with red-shifted absorption maxima.

Recently, we have reported palladium-catalyzed methodologies toward C–C-linked carbohydrate–porphyrin hybrids.^18,19^ However, our previous approaches exploited functionalization of porphyrin system through *meso*-aryl rings. While synthetic simplicity justified this strategy, the aryl rings usually adopt an orthogonal orientation relative to the porphyrin core, which results in no significant changes in the UV–vis profile of such molecules. Herein, we developed the concept of synthetically more challenging, less symmetric *trans*-A_2_B_2_-type porphyrins (Scheme 1). Sonogashira reaction on this scaffold has been explored in some recent studies, and changes in the UV–vis profile have been exploited with aromatic substituents.^20–22^ In this paper, we present modified conditions for Sonogashira coupling, allowing for direct glycosylation of the *meso* position. As a result, C–C-conjugated porphyrins are formed with glycal moieties participating in the porphyrin core-conjugated system leading to red-shifted glycoporphyrins. Additionally, by choosing the MacDonald-type A_2_B_2_ porphyrin synthesis strategy,^23^ we were able to diversify the scope of porphyrin examples. With that, further functionalization of photosensitizers through functional groups localized at *meso* substituents is still possible.

**Scheme 1:**
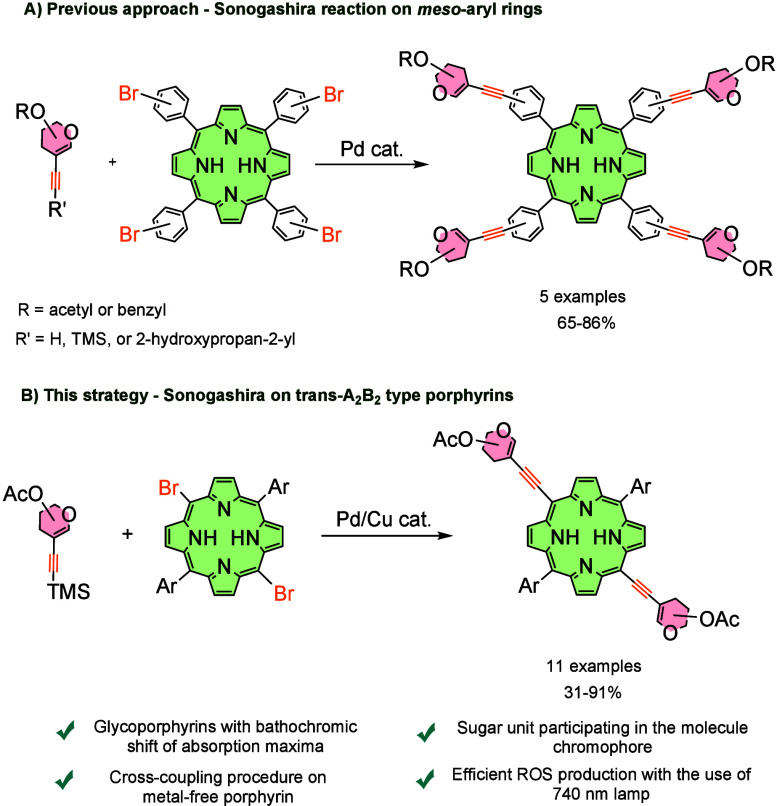
General Context of the Studies

Two subunits were required in our strategy. We chose glycals as one of the most diverse groups of sugar synthons.^24–27^ The alkynylated glucal derivative (**2a**) was synthesized following our own protocol^18^ starting from iodoglucal **1a**, the popular starting material for modern sugar methodologies.^28–37^ To obtain dibromoporphyrin **5a**, we have explored some published synthetic paths looking for the most convenient path in terms of economical availability and reproducibility. In the first step (Scheme 2), we applied an economic procedure toward dipyrromethane (**1**).^38^ Despite a low yield (24%), the aqueous procedure itself was easy to scale up and used only 3 equiv of excess pyrrole. Next, macrocyclization was conducted with the use of TFA, leading to a *trans*-A_2_B_2_-type porphyrin with a good yield of 58%. Final starting material **5a** was synthesized following the bromination protocols with the use of pyridine (and methanol in some cases; see the Supporting Information), leading to dibromoporphyrin **5a** with an excellent yield of 99%.

**Scheme 2:**
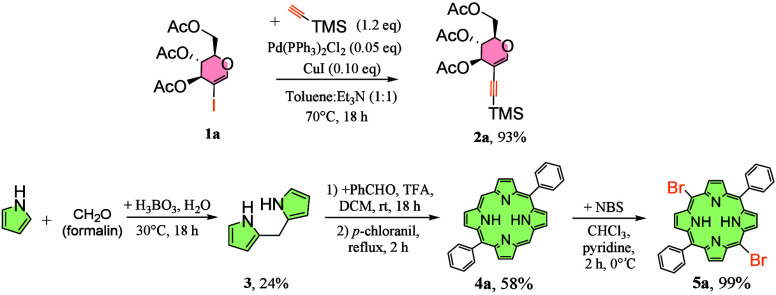
Synthesis of Starting Materials

As a starting point of optimization studies toward **6a** (for detailed studies, see the Supporting Information), we tried previously developed conditions for *meso*-aryl glycosylation.^18^ However, satisfactory results were achieved neither at the higher temperature nor at room temperature (Table 1, entries 1 and 2). Both positions differ significantly in electron density and reactivity; therefore, such differences were not surprising. In the next step, we decided to look for milder conditions that better promoted reactivity at the *meso* position. Indeed, with PdCl_2_ in a toluene/triethylamine mixture, the cross-coupled product was isolated in a fair yield of 45% (Table 1, entry 3). We observed slight progress of substrate conversion at 40 °C (entry 4), and the reaction accelerates significantly when the palladium source is changed to a more soluble one in organic solvents. The product is then formed in a very good yield of 75% (entry 4 vs entry 5). We continued the optimization to identify the most optimal ligand and observed that among those tested, BrettPhos provides the highest yield of 82% (entry 5 vs entries 6–8). In a scale-up experiment, we discovered that this yield might be even increased to 91% (minor losses during the purification process). In small scale experiments, another increase in yield might be achieved with an increased concentration of the mixture (entry 9); however, these conditions did not scale up easily and were optimal for **6a** solely.

**Table 1:**
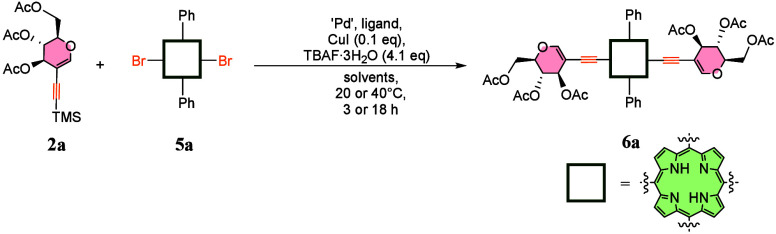
Optimization of the Sonogashira Reaction^a^

entry	solvent^b^	catalyst (equiv)	ligand (equiv)	temp (°C), time (h)	yield of **6a** (%)
1	dioxane^c^/Et_3_N	Pd-XPhos-G3 (0.3)	XPhos (0.3)	90, 18	0^d^
2	dioxane/Et_3_N	Pd-XPhos-G3 (0.05)	XPhos (0.05)	20, 18	traces^d^
3	toluene/Et_3_N	PdCl_2_ (0.1)	XPhos (0.2)	20, 18	45
4	toluene/Et_3_N	PdCl_2_ (0.1)	XPhos (0.2)	40, 18	47
5	toluene/Et_3_N	Pd(OAc)_2_ (0.1)	XPhos (0.2)	40, 3	75
6	toluene/Et_3_N	Pd(OAc)_2_ (0.1)	SPhos (0.2)	40, 3	52
7	toluene/Et_3_N	Pd(OAc)_2_ (0.1)	XantPhos (0.2)	40, 3	70
8	toluene/Et_3_N	Pd(OAc)_2_ (0.1)	BrettPhos (0.2)	40, 3	82 (91)^e^
9	toluene/Et_3_N	Pd(OAc)_2_ (0.1)	BrettPhos (0.2)	40, 3	88^f^

a General conditions: porphyrin **5a** (0.038 mmol, 1.0 equiv), ethynylated glucal **2a** (4.0 equiv), TBAF·3H_2_O (4.1 equiv), copper­(I) iodide (0.1 equiv), toluene (2.5 mL), triethylamine (2.5 mL), argon atmosphere.

b A 1:1 mixture (v:v).

c 1,4-Dioxane.

d Reaction without CuI.

e A 10-fold scale reaction.

f Toluene (1.25 mL), triethylamine (1.25 mL).

The UV–vis spectrum of **6a** confirmed the validity of the initial concept of our studies and proved the participation of the sugar unit in the conjugation with the porphyrin chromophore system. We selected two model porphyrins as the most convenient references: **TPP** (5,10,15,20-tetraphenyl-porphyrin) and 5,15-diphenyl-10,20-bis­((trimethylsilyl)­ethynyl)-porphyrin (**EtDPP**; for the synthesis, see the Supporting Information). Glycosylated porphyrin **6a** was characterized with red-shifted Q bands absorbing the visible light from the region up to 705 nm (maximum at 691 nm) (Figure 1). The lowest energetic local maximum of **TPP** occurs at 647 nm, while for **EtDPP**, the Q band is shifted to 678 nm. The additional difference in absorption maxima between **6a** and **EtDPP** proves that the glycal unit participates in the conjugation system and influences the final outcome on UV–vis of the whole hybrid.

**Figure 1:**
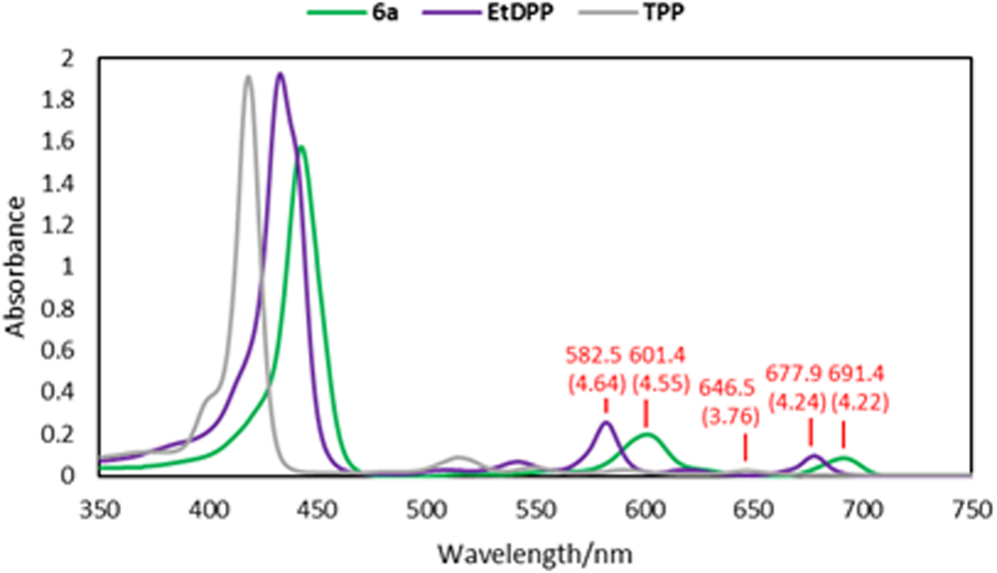
UV–vis spectra (CHCl_3_) of **6a** in comparison with those of reference porphyrins **TPP** and **EtDPP**.

We further challenged our methodology to determine the scope with regard to glycal moieties for two key reasons. First, as we observed conjugation between the sugar residue and macroheterocycle through the ethynyl linker, we expected that the carbohydrate unit itself is probably the only direct factor that might influence the level of the bathochromic shift on UV–vis spectra. Second, considering the final application of our hybrids in PDT and their ultimate interactions with sugar-recognizing cancer lectins, we expected that each PS might have a different affinity for cancer cells. Hence, the library of glycosylated PS might help to determine the most promising sugar units for applications in PDT. In our studies, we exemplified glycoporphyrins by using four different sugar derivatives (d-glucal-, d-galactal-, d-xylal-, and l-rammnal- (**6a**–**6d**, respectively)) with good overall yields (Scheme 3).

**Scheme 3:**
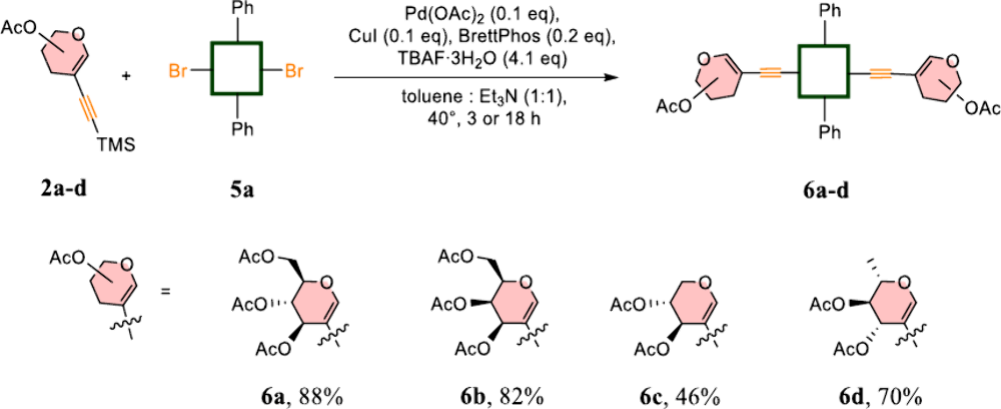
Differentiation of Sugar Residues

Products **6a**–**6d** were characterized with bathochromic shifts of the absorption maxima. Among the glycals tested, the d-galactal moiety seems to adopt the conformation that maximizes the conjugation with the macroheterocycle ring (Figure 2). Further development of this phenomenon may create a starting point for new glycosylated PSs.

**Figure 2:**
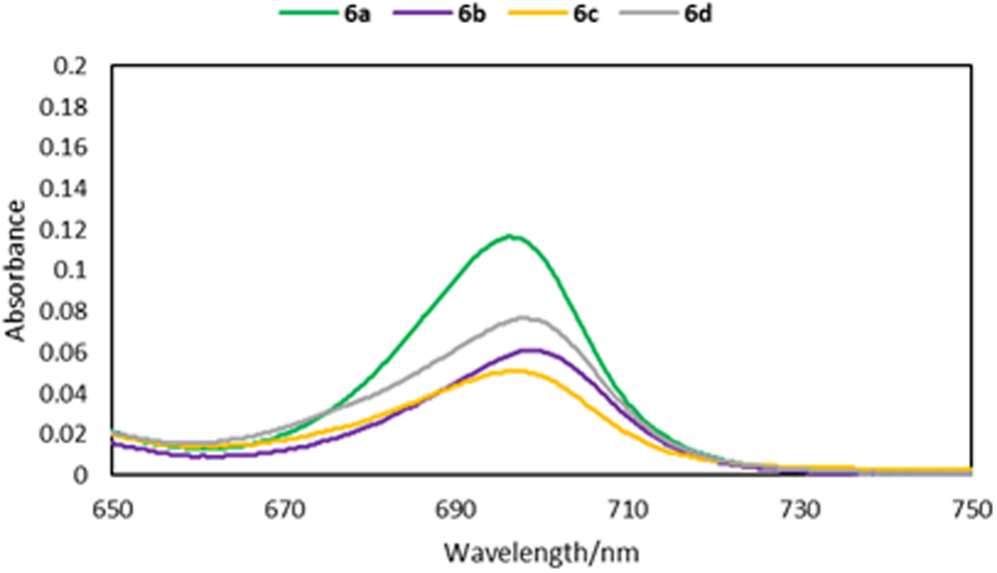
UV–vis spectra (DMF) and comparison of the Q bands (**6a**–**6d**).

To further challenge our methodology, we decided to test its versatility with regard to the porphyrin scaffold (Scheme 4). We created macroheterocycles with different *meso*-aryl moieties. The included parameters were electron density (**5b** and **5c**), the presence of functional groups (**5b** and **5f–5h**), and the possibility of using heteroaromatic rings (**5e**). Glycoporphyrins (**7b**–**7h**) were obtained with overall yields ranging from fair to excellent (31–90%). The main limitation was related to the synthetic accessibility and solubility of dibromoporphyrins (**5b**–**5h**). Alkyl substituents significantly improve the solubility of the porphyrins, which is crucial for successful cross-coupling. The impact of this factor was particularly visible in comparison of the reactivity of **5h** and **5i**, and the *meta*-cyano derivative was soluble enough to provide **7h** with an acceptable yield of 31%; on the other hand, only traces of **7i** were detected in the reaction mixture of **5i**. Last but not least, halogen atoms remaining at macrocycles **7f** and **7g** might be an interesting starting point for further functionalization and new cross-coupling strategies. As expected, apart from **7e**, *meso*-aryl rings have a negligible influence on the UV–vis spectra of porphyrin systems.

**Scheme 4:**
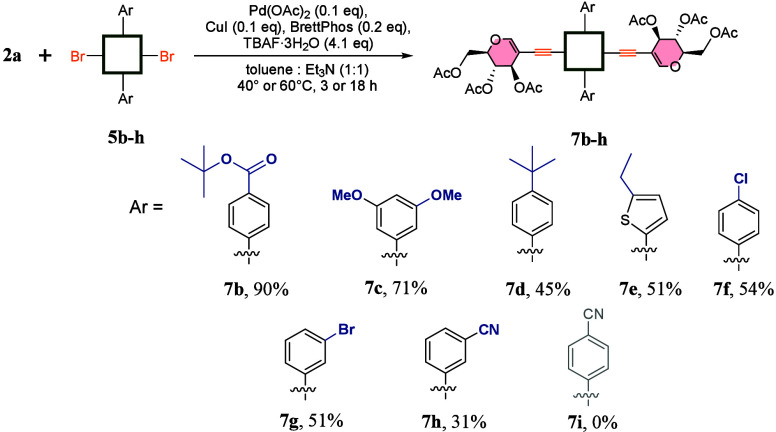
Scope with Regard to *meso*-Aryl Substituents

The final step was exemplified on model glycoporphyrin **6a** with our previously reported procedure of hydroxyl group deprotection (Scheme 5). Deacetylated product **8a** was isolated in a very good yield of 84%, proving the utility of the whole synthetic methodology.

**Scheme 5:**
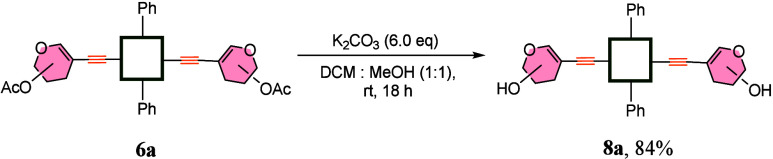
Deprotection Step of the Synthesis

To determine the ability of our compounds to produce ROS, we followed a well-established protocol using single-wavelength absorbance measurement^39–41^ with 9,10-dimethylanthracene (DMA) as an indicator of ROS production (Figure 3; details in the Supporting Information). To our delight, we observed that both acetylated porphyrins and porphyrins with free hydroxyl groups (**6a** vs **8a**) efficiently generate ROS. Furthermore, we were able to produce ROS using a 690 nm lamp (Figure 3A) and an even less energetic and more penetrating 740 nm lamp (Figure 3B, commercial lamp used with about 25% of peak emission at 720 nm). The latter result was particularly impactful as it gives direct proof of glycal’s influence on ROS production in the biologically attractive visible light region. The reference PS (**EtDPP**) did not induce significant oxidation of DMA under these conditions, while **8a** could be used with such a light source.

**Figure 3:**
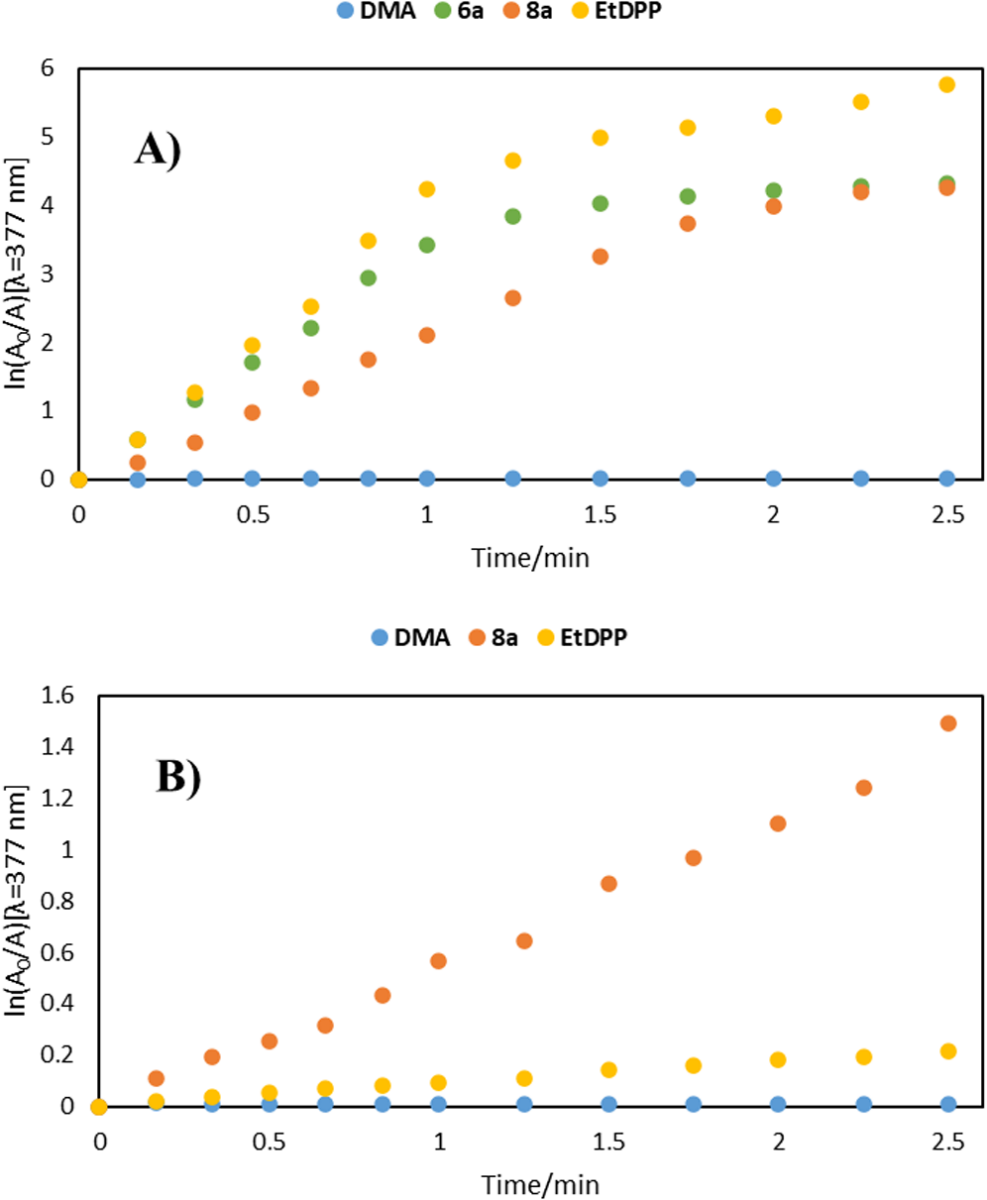
Photooxidation of DMA (300 μM) and PS (18 μM) ((A) λ_irr_ = 690 nm and (B) λ_irr_ = 740 nm) photosensitized in DMF.

To further study the potential of our compound, we preliminarily estimated the quantum yield of **6a** and **8a** using a porphyrin reference (**TPP** in our case).^42,43^ The samples were irradiated at 550 nm, where both **6a** and **8a** as well as **TPP** showed low, yet non-zero absorbance. Other irradiation wavelengths such as ∼425 nm (Soret band for **TPP**) could not be used due to the bathochromic shift of our compounds, which made it impossible to ensure similar absorbance of the tested samples. In contrary, near 550 nm all tested porphyrins showed similar absorbance, which still allowed for ROS production (Figure 4).^44^

**Figure 4:**
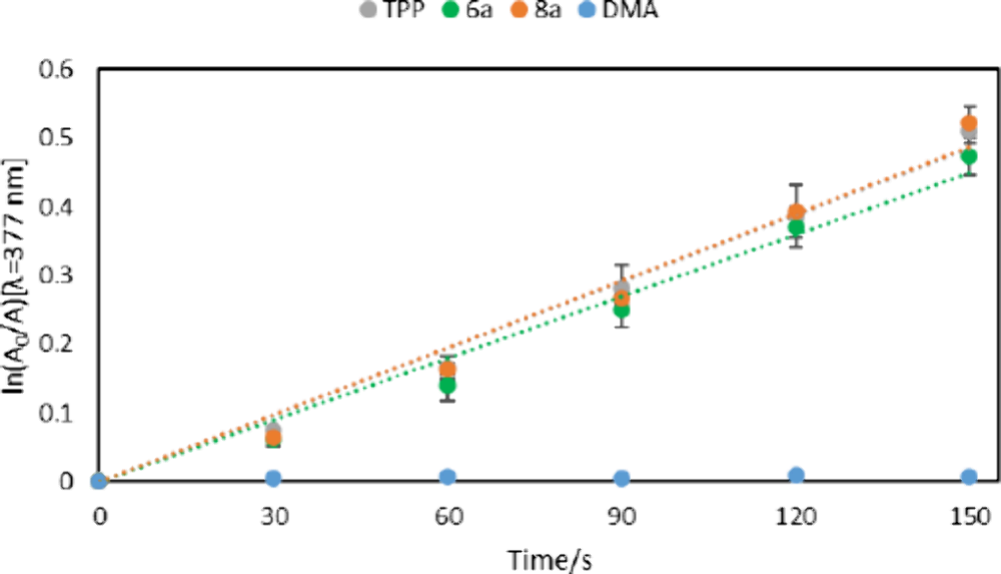
First-order plots for the photooxidation of DMA (300 μM), photosensitized by **TPP**, **6a**, and **8a** in DMF (λ_irr_ = 550 nm). Values represent the mean ± standard deviation of three separate experiments. Molarities of PS were adjusted to equalize the absorbance of each sample at 550 nm.

Obtained kinetic data were used to calculate the kinetic parameters and quantum yields of **6a** and **8a** in DMF by comparing the slope for the PS with that of the reference (**TPP** (Table 2)). Our glycoporphyrins showed quantum yields comparable to that of **TPP**. Further testing is required to confirm this finding, preferably using reference PS with similar wavelengths of absorption maxima, e.g., Foscan.

**Table 2:** Kinetic Parameters for the Photooxidation of DMA, Including the Observed Rate Constant (*k*_obs_) and Singlet Molecular Oxygen Quantum Yield (Φ_Δ_) in DMF

	**TPP**	**6a**	**8a**
*k*_obs_^DMF^ (s^–1^)	(3.2 ± 0.2) × 10^–3^	(3.1 ± 0.2) × 10^–3^	(3.3 ± 0.2) × 10^–3^
Φ_Δ_^DMF^	0.64 ^45^	0.62 ± 0.04	0.66 ± 0.04

Our studies present a new methodology for hydrolytically stable conjugation between 2-ethynylglycal moieties and A_2_B_2_-type porphyrins. Sonogashira reactivity allowed us to combine two subunits via C–C bonds. As a result, 11 new glycoporphyrins were obtained with proven ROS generation capability and impactful spectroscopic features for application in PDT. The observed red-shift of the absorption maxima provides access to promising third-generation PSs.

## Supplementary Material



## Data Availability

The data underlying this study are available in the published article, in its Supporting Information, and openly available in the Zenodo database at 10.5281/zenodo.15728134.

## Notes

Experimental procedures and characterizations and UV–vis and NMR spectra (PDF)
