# Supplementary material for: Red-Shifted Glycoporphyrins: Synthesis via Sonogashira Cross-Coupling and Studies on Reactive Oxygen Species Production
Source: Org Lett. 2025 Jul 14;27(29):7779–84. doi: 10.1021/acs.orglett.5c02038 (PMC12305654; doi:10.1021/acs.orglett.5c02038)

Supporting Information

Red-shifted glycoporphyrins – synthesis *via* Sonogashira cross-coupling and studies on reactive oxygen species production

Dariusz Baran, Maciej Malinowski*

General experimental methods

All chemical operations were carried out using sealed tubes or round-bottom flasks. Acetonitrile (POCH) was distilled under argon, over CaH_2. Triethylamine (Chempur) and pyridine (POCH) were dried over KOH. 1,4-dioxane (POCH) and toluene (Chempur) were dried over molecular sieves (4Å). Other solvents were used without further purification.

Pyrrole, NIS, ethynyltrimethylsilane, *p*-chloranil and TBAF·$3H_2O$ were from Fluorochem. Formalin was from WarChem. L-Rhamnose, peracetylated D-galactal **SI2** and 5-ethyl-2-thiocarboxaldehyde were from Angene. D-Xylose, NBS, 4-chlorobenzaldehyde and CuI were from Sigma-Aldrich. Peracetylated D-glucal **SI1**, 3-cyanobenzaldehyde, 3-bromobenzaldehyde, 3,5-dimethoxybenzaldehyde, *tert*-butyl 4-formylbenzoate and BrettPhos were from AmBeed. 4-*tert*-butylbenzaldehyde and $Pd(OAc)_2$ were from ApolloScientific. TFA was from ThermoScientific.

Reactions were monitored by thin-layer chromatography on silica gel plates (VWR, F254 silica, 200 µm layer thickness, aluminium sheets). Sugar derivatives were spotted by immersing in 5% ethanolic solution of phosphomolybdic acid followed by heating or rendered under ultraviolet lamp. Porphyrin derivatives were observed under daylight or rendered under ultraviolet lamp. All reactions were performed using block heaters unless stated otherwise.

^{1}H NMR (600 MHz) and ^{13}C NMR (151 MHz) were recorded at 293 K. Chemical shifts are given in ppm (δ) and are referenced to internal solvent signal or to TMS used as an internal standard. Multiplicities are declared as follows: s (singlet), broad s (broad singlet), d (doublet), broad d (broad doublet), t (triplet), q (quartet), p (pentet) dd (doublet of doublets), ddd (doublet of doublets of doublets), dddd (doublet of doublets of doublets of doublets), dt (doublet of triplets), td (triplet of doublets), m (multiplet). Coupling constants *J* are given in Hz. Optical rotations were measured on polarimeter (Anton-Paar MCP-150) at 598 nm. $[\alpha]_D^{20}$ is expressed in °·cm^3·g^{-1}·dm^{-1} and c is expressed in g/100 cm^3. High resolution mass spectra (HRMS, ESI) and mass spectra (MS) were measured on a LTQ Orbitrap Velos spectrometer, in positive ionization mode. UV-Vis spectra and single-wavelength absorbance measurements for ROS production studies were measured on a LLG-*uniSPEC* 2 spectrophotometer or VWR® UV-6300PC spectrophotometer. Microwave reactions were conducted in AntonPaar MonoWave 400 microwave reactor.

Table of contents

1. Optimisation studies on sila-Sonogashira cross-coupling between peracetylated (2-trimethylsilylethynyl)-D-glucal (**2a**) and dibrominated porphyrin **5a** 6

1.1 General optimisation 6

1.2 Ligand optimisation 7

2. Synthesis of substrates 8

2.2 Synthesis and characterisation of 2-trimethylsilylethynylated glycals (**2a-d**) 14

2.3 Synthesis and characterisation of di(pyrrol-2-yl)methane 17

2.4 Synthesis and characterisation of *trans*-A_2B_2 porphyrins (**4a-h**) 18

2.5 Synthesis and characterisation of dibrominated *trans*-A_2B_2 porphyrins (**5a-h**) 26

3. Synthesis of new compounds 34

3.1 Synthesis and characterisation of new *trans*-A_2B_2 glycoporphyrins with different sugar motifs (**6a-d**) 34

3.2 Synthesis and characterisation of new glycoporphyrins with different aryl fragments (**7b-h**) ... 39

3.2 Synthesis and characterisation of deprotected glycoporphyrin **8a** 46

4. Synthesis of known porphyrins for ROS studies 47

5. Reactive oxygen species generation plots of new glycoporphyrins 50

6. UV-vis spectra of new compounds 55

7. NMR spectra of known compounds 62

8. NMR spectra of new compounds 72

General procedure for ROS production detection by 9-10-dimethylanthracene (DMA) photo-oxidation

2.4 µmol of tested porphyrin was dissolved in 10.0 ml of DMF. From this solution, 0.15 ml was taken and added to a quartz cell. Next, 0.85 ml of DMF was added, followed by 1.00 ml of 0.6 mM solution of DMA in DMF. Cell content was mixed to ensure stable absorbance reading.

The test sample was irradiated by EvoluChem 690 PF lamp (18 W, 690 nm) or EvoluChem 740 PF lamp (18 W, 740 nm) from the distance of 24 cm. After 10 seconds of irradiation, the absorbance of the test sample was measured using spectrophotometer. This procedure was then repeated until 1 minute of total irradiation time. Later, irradiation time increased to 15 seconds between each absorbance measurement, until 2.5 minutes of total irradiation time.

Control samples without photosensitizer were prepared using 1.00 ml of DMF and 1.00 ml of DMA solution. Blank samples without DMA were prepared using 0.15 ml of tested porphyrin solution and 1.85 ml of DMF.

For **EtDPP** due to its unsatisfactory solubility in DMF, tested solution was prepared in chloroform. This solution was later diluted with DMF by using the same protocol as any other samples of photosensitisers.

General procedure for the estimation of quantum yield by 9-10-dimethylanthracene (DMA) photo-oxidation

2.4 µmol of tested porphyrin was dissolved in 10.0 ml of DMF. For **TPP**, similar solution was made using 2.4 µmol of porphyrin and 100 ml of DMF. From prepared solutions, such volume was taken and added to the quartz cell to make the final solution's absorbance equal to 0.05 at 550 nm (0.50 ml for **TPP**, 0.080 ml for **6a**, 0.16 ml for **8a**). Next, 1.00 ml of 0.6 mM solution of DMA in DMF was added to the quartz cell. Lastly, the quartz cell was filled up to 2.0 ml with DMF. Cell content was mixed to ensure stable absorbance reading.

The test sample was irradiated by EvoluChem 550 PF lamp (18 W, 550 nm) from the distance of 24 cm. After 30 seconds of irradiation, the absorbance of the test sample was measured using spectrophotometer. This procedure was then repeated until 2.5 minutes of total irradiation time. For each porphyrin, 3 tests were performed.

Control sample without photosensitizer were prepared using 1.00 ml of DMF and 1.00 ml of DMA solution. Blank samples without DMA were prepared using appropriate volumes of tested porphyrin solutions and filled up to 2.0 ml with DMF.

1. Optimisation studies on sila-Sonogashira cross-coupling between peracetylated (2-trimethylsilylethynyl)-D-glucal (**2a**) and dibrominated porphyrin **5a**

1.1 General optimisation

Entry[a]	Eq of **2a**	Catalyst (eq)	CuI (eq)	Ligand (eq)	Solvent (v/v ratio)	Tempera-ture	Time	Yield[b] **6a**
**SI1**	4	Pd-Xphos-G3 (0.30)	-	Xphos (0.30)	1,4-Dioxane:Et_3N (1:1)	90 °C	18 h	0%
**SI2**	4	$Pd(PPh)_2Cl_2$ (0.12)	0.18	-	THF:Et_3N (1:1)	20 °C	18 h	29%[c]
**SI3**	4	Pd-Xphos-G3 (0.05)	-	XPhos (0.05)	1,4-Dioxane:Et_3N (1:1)	20 °C	18 h	Traces
**SI4**	4	$PdCl_2$ (0.10)	0.10	XPhos (0.20)	Toluene:Et_3N (1:1)	20 °C	18 h	45%[c]
**SI5**	4	$PdCl_2$ (0.18)	0.10	XPhos (0.20)	DMF:Et_3N (1:1)	20 °C	18 h	Traces
**SI6**	4	$PdCl_2$ (0.12)	0.10	XPhos (0.20)	1,4-Dioxane:Et_3N (1:1)	20 °C	18 h	Traces
**SI7**	4	$PdCl_2$ (0.28)	0.10	XPhos (0.20)	THF:Et_3N (1:1)	20 °C	18 h	Traces
**SI8**	4	$PdCl_2$ (0.18)	0.18	XPhos (0.20)	Toluene:Et_3N (1:1)	20 °C	18 h	7%[c]
**SI9**	4	$PdCl_2$ (0.10)	0.10	XPhos (0.20)	Toluene:Et_3N (1:1)	40 °C	18 h	47%[c]
**SI10**	4	$Pd(OAc)_2$ (0.10)	0.10	XPhos (0.20)	Toluene:Et_3N (1:1)	40 °C	18 h	29%[c]
**SI11**	3	$PdCl_2$ (0.22)	0.10	XPhos (0.2))	Toluene:Et_3N (1:1)	40 °C	18 h	13%
**SI12**	4	$PdCl_2$ (0.13)	0.10	XPhos (0.24)	Toluene:Et_3N (1:1)	20 °C	18 h	13%[c]
**SI13**	4	$PdCl_2$ (0.10)	0.10	XPhos (0.20)	Toluene:Et_3N (1:1)	40 °C	18 h	Traces
**SI14**	4	$PdCl_2$ (0.13)	0.10	XPhos (0.20)	Toluene:Et_3N (2:1)	40 °C	18 h	11%[c]
**SI15**	4	$PdCl_2$ (0.12)	0.12	XPhos (0.19)	Toluene:Et_3N (1:2)	40 °C	18 h	21%
**SI16**	4	$Pd(OAc)_2$ (0.10)	0.10	XPhos (0.20)	Toluene:Et_3N (1:1)	60 °C	18 h	70%
**SI17**	4	$PdCl_2$ (0.10)	0.10	XPhos (0.20)	Toluene:Et_3N (1:1)	60 °C	18 h	63%
**SI18**	4	$Pd(OAc)_2$ (0.10)	0.10	XPhos (0.20)	Toluene:Et_3N (1:2)	60 °C	18 h	41%
**SI19**	4	$Pd(OAc)_2$ (0.10)	-	XPhos (0.20)	Toluene:Et_3N (1:1)	60 °C	18 h	26%
**SI20**	4	$Pd(OAc)_2$ (0.10)	0.10	XPhos (0.20)	Toluene:Et_3N (1:1)	60 °C	18 h	57%
**SI21**	4	$Pd(OAc)_2$ (0.10)	0.10	XPhos (0.20)	Toluene:Et_3N (1:1)	20 °C	18 h	41%
**SI22**	4	$Pd(OAc)_2$ (0.10)	0.10	-	Toluene:Et_3N (1:1)	60 °C	18 h	50%
**SI23**	4	$Pd(OAc)_2$ (0.10)	0.10	XPhos (0.20)	Pyridine:Et_3N (1:1)	20 °C	18 h	50%

**SI24**	4	$Pd(OAc)_2$ (0.10)	0.10	XPhos (0.20)	Toluene:Pyridine (1:1)	20 °C	18 h	2%
**SI25**	4	$Pd(OAc)_2$ (0.10)	0.10	XPhos (0.20)	Toluene:Et_3N (1:1)	40 °C	18 h	72%
**SI26**	3	$Pd(OAc)_2$ (0.10)	0.10	XPhos (0.20)	Toluene:Et_3N (1:1)	40 °C	18 h	50%[d]
**SI27**	4	$Pd(OAc)_2$ (0.10)	0.10	XPhos (0.20)	Toluene:Et_3N (2:1)	40 °C	18 h	50%[d]
**SI28**	4	$Pd(OAc)_2$ (0.10)	0.10	XPhos (0.20)	Toluene:Et_3N (1:2)	40 °C	18 h	20%[d]
**SI29**	4	$Pd(OAc)_2$ (0.10)	0.10	BrettPhos (0.20)	Toluene:Et_3N (1:1)	40 °C	3 h	88%[e]
**SI30**	4	$Pd(OAc)_2$ (0.10)	0.10	BrettPhos (0.20)	Toluene:Et_3N (1:1)	40 °C	3 h	43%[f]

[a] General conditions: porphyrin **5a** (23.5 mg, 0.038 mmol, 1.0 eq), glucal derivative **2a**, TBAF·$3H_2O$ (3.1 or 4.1 eq, depending on **2a**), catalyst, CuI, ligand, solvent system (5.0 ml), argon atmosphere, in sealed tube. [b] Isolated yield, column chromatography. [c] Sugar substrate present as impurity in isolated product. [d] Determined from crude mixture by the ^{1}H NMR experiment with internal standard ($CHBr_3$). [e] Two times higher substrate concentration (2.5 ml of solvent system). [f] Two times lower substrate concentration (10.0 ml of solvent system).

1.2 Ligand optimisation

Entry[a]	Catalyst (eq)	CuI (eq)	Ligand (eq)	Solvent (v/v ratio)	Tempera-ture	Time	Yield[b] **6a**
**SI31**	$Pd(OAc)_2$ (0.10)	0.10	XPhos (0.20)	Toluene:Et_3N (1:1)	40 °C	3 h	75%
**SI32**	$Pd(OAc)_2$ (0.10)	0.10	XantPhos (0.20)	Toluene:Et_3N (1:1)	40 °C	3 h	70%[c]
**SI33**	$Pd(OAc)_2$ (0.10)	0.10	XantPhos (0.10)	Toluene:Et_3N (1:1)	40 °C	3 h	55%[c]
**SI34**	$Pd(OAc)_2$ (0.10)	0.10	DavePhos (0.20)	Toluene:Et_3N (1:1)	40 °C	3 h	76%
**SI35**	$Pd(OAc)_2$ (0.10)	0.10	JohnPhos (0.20)	Toluene:Et_3N (1:1)	40 °C	3 h	79%
**SI36**	$Pd(OAc)_2$ (0.10)	0.10	RuPhos (0.20)	Toluene:Et_3N (1:1)	40 °C	3 h	69%
**SI37**	$Pd(OAc)_2$ (0.10)	0.10	SPhos (0.20)	Toluene:Et_3N (1:1)	40 °C	3 h	52%
**SI38**	$Pd(OAc)_2$ (0.10)	0.10	PPh_3 (0.20)	Toluene:Et_3N (1:1)	40 °C	3 h	35%
**SI39**	$Pd(OAc)_2$ (0.10)	0.10	BINAP (0.20)	Toluene:Et_3N (1:1)	40 °C	3 h	15%[c]
**SI40**	$Pd(OAc)_2$ (0.10)	0.10	BINAP (0.10)	Toluene:Et_3N (1:1)	40 °C	3 h	Traces
**SI41**	$Pd(OAc)_2$ (0.10)	0.10	dppf (0.20)	Toluene:Et_3N (1:1)	40 °C	3 h	47%
**SI42**	$Pd(OAc)_2$ (0.10)	0.10	dppf (0.10)	Toluene:Et_3N (1:1)	40 °C	3 h	52%
**SI43**	$Pd(OAc)_2$ (0.10)	0.10	BrettPhos (0.20)	Toluene:Et_3N (1:1)	40 °C	3 h	82%
**SI44**	$Pd(OAc)_2$ (0.10)	0.10	tBuXPhos (0.20)	Toluene:Et_3N (1:1)	40 °C	3 h	80%

[a] General conditions: porphyrin **5a** (23.5 mg, 0.038 mmol, 1.0 eq), glucal derivative **2a** (4.0 eq), TBAF·$3H_2O$ (4.1 eq), catalyst, CuI, ligand, solvent system (5.0 ml), argon atmosphere, in sealed tube. [b] Isolated yield, column chromatography. [c] Determined from crude mixture by the ^{1}H NMR experiment with internal standard ($CHBr_3$).

2. Synthesis of substrates

2.1 Synthesis and characterisation of peracetylated 2-iodoglycals (**1a-d**)

3,4,6-tri-O-acetyl-2-iodo-D-glucal (**1a**)

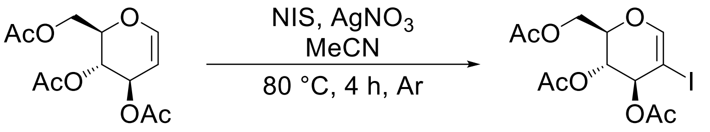

SI1 **1a**

A flame-dried flask, equipped with a stir bar and a condenser was evacuated and backfilled with argon 3 times (Schlenk technique). Next, 40 ml of freshly distilled MeCN (under argon, with CaH_2) was added under constant flow of argon. Solvent was preheated to 60 °C. After that, 3,4,6-tri-*O*-acetyl-D-glucal **SI1** (5.00 g, 18.37 mmol, 1.0 eq), $AgNO_3$ (624 mg, 3.67 mmol, 0.2 eq) and NIS (4.96 g, 22.04 mmol, 1.2 eq) were added under constant flow of argon. Last portion of MeCN (10 ml) was then added and used to flush remains of substrates from flask walls. Mixture was stirred in darkness at 80 °C for 4 h. Upon completion, reaction mixture was filtrated through a viscose wool and washed with EtOAc. Filtrate was concentrated to dryness with silica. Residue was purified by flash chromatography (cyclohexane (until violet iodine was removed) to cyclohexane/ethyl acetate, 8:2, (v/v)) yielding yellowish crystals of glucal **1a** (3.08 g, 42%). The characterisation data were in agreement with those of the literature.[1]

$\boldsymbol{R_f}$ = 0.32 (cyclohexane/ethyl acetate, 8:2, (v/v)).

^{1}**H NMR** (600 MHz, $CDCl_3$) δ [ppm]: 6.79 (d, J = 1.2 Hz, 1H), 5.50 (d, J = 5.2 Hz, 1H), 5.23 (dd, J = 7.0, 5.2 Hz, 1H), 4.44 – 4.36 (m, 2H), 4.23 – 4.16 (m, 1H), 2.11 (s, 3H), 2.08 (s, 3H), 2.07 (s, 3H).

3,4,6-tri-O-acetyl-2-iodo-D-galactal (**1b**)

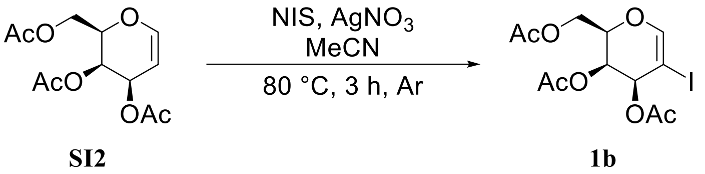

A flame-dried flask, equipped with a stir bar and a condenser was evacuated and backfilled with argon 3 times (Schlenk technique). Next, 10 ml of freshly distilled MeCN (under argon, with CaH_2) was added under constant flow of argon. Solvent was preheated to 60 °C. After that, 3,4,6-tri-*O*-acetyl-D-galactal **SI2** (1.50 g, 5.51 mmol, 1.0 eq), $AgNO_3$ (187 mg, 1.10 mmol, 0.2 eq) and NIS (1.49 g, 6.61 mmol, 1.2 eq) were added under constant flow of argon. Last portion of MeCN (5 ml) was then added and used to flush remains of substrates from flask walls. Mixture was stirred in darkness at 80 °C for 3 h. Upon completion, reaction mixture was filtrated through a short pad of Celite® and washed with EtOAc. Filtrate was concentrated to dryness with silica. Residue was purified by flash chromatography (hexane (until violet iodine was removed) to hexane/ethyl acetate, 7:3, (v/v)) yielding yellowish crystals of galactal **1b** (1.20 g, 55%). The characterisation data were in agreement with those of the literature.[1]

R_f = 0.32 (hexane/ethyl acetate, 7:3, (v/v)).

^{1}H NMR (500 MHz, $CDCl_3$) δ [ppm]: 6.78 (d, J = 1.6 Hz, 1H), 5.59 (ddd, J = 4.6, 1.6, 1.1 Hz, 1H), 5.50 (dd, J = 4.6, 2.1 Hz, 1H), 4.47 – 4.39 (m, 1H), 4.27 (dd, J = 11.8, 7.5 Hz, 1H), 4.20 (dd, J = 11.8, 5.3 Hz, 1H), 2.12 (s, 3H), 2.09 (s, 3H), 2.08 (s, 3H).

3,4-di-O-acetyl-2-iodo-D-xylal (**1c**)

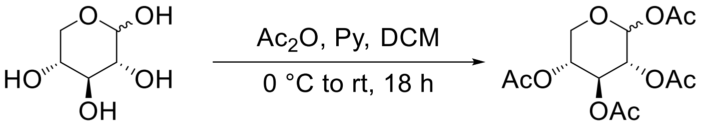

SI3 **SI4**

To a round-bottom flask, D-xylose **SI3** (5.00 g, 33.30 mmol, 1.0 eq) was added. Solid was suspended in DCM (34 ml) and pyridine (16 ml). Resulting mixture was cooled down to 0 °C (ice bath) and acetic anhydride (17.3 ml, 183 mmol, 5.5 eq) was added. Mixture was slowly warmed to room temperature and stirred for 18 h. Upon completion, reaction was quenched with 20 ml of distilled water. Next, mixture was transferred to a separatory funnel, where phases were separated. Aqueous phase was extracted with DCM (2x80 ml). Combined organic phases were washed with 2M HCl (3x80 ml, or until smell of pyridine was no longer noticeable) and with saturated aqueous $NaHCO_3$ solution (1x80 ml). Organic phase was dried over $MgSO_4$ (anh.). Resulting suspension was filtered and concentrated *in vacuo* yielding off-white crystals of peracetylated D-xylose **SI4** (10.49 g, 99%, R_f = 0.36 (hexane/ethyl acetate, 7:3, (v/v)). Obtained product was of sufficient quality and was used directly in the next step.

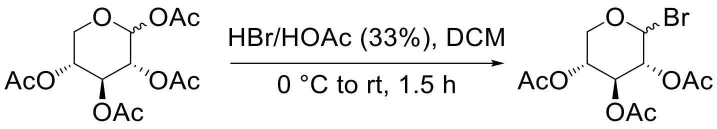

SI4 **SI5**

Peracetylated D-xylose **SI4** (5.62 g, 17.65 mmol, 1.0 eq) was dissolved in 127 ml of DCM in a round-bottom flask. Resulting solution was cooled down to 0 °C (ice bath) and HBr/HOAc (33% wt.%) was slowly added (17.2 g, 12.7 ml, 70.15 mmol, 4.0 eq). Mixture was stirred for 1 h at 0 °C, after which ice bath was removed, and mixture was further stirred for another 0.5 h at room temperature. Upon completion, reaction mixture was diluted with 100 ml of DCM. Mixture was transferred to a separatory funnel, where phases were separated. Organic phase was washed with 100 ml of distilled water, 100 ml of solution (70:30, water/sat. aqueous $NaHCO_3$ solution, v/v), 100 ml of sat. aqueous $NaHCO_3$ solution and 70 ml of brine. Organic phase was dried over $MgSO_4$ (anh.). Resulting suspension was filtered and concentrated *in vacuo*. Residue was purified by crystallisation: dissolved in 25 ml Et_2O (boiling), precipitated with 30 ml hexane, left for 0.5 h at 4 °C. Suspension was filtered yielding peracetylated D-xylosyl bromide **SI5** (3.77 g, 65%, R_f = 0.74 (hexane/ethyl acetate, 7:3, (v/v)) as white crystals.

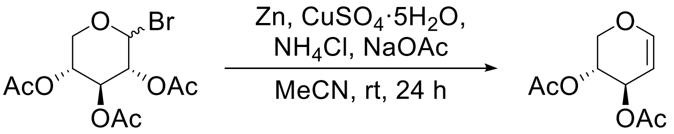

SI5 **SI6**

Peracetylated D-xylosyl bromide **SI5** (2.68 g, 7.91 mmol, 1.0 eq) was dissolved in 16.5 ml of freshly dried MeCN (over CaH_2) in a round-bottom flask. Next, $CuSO_4·5H_2O$ (200 mg, 0.79 mmol, 0.1 eq), NaOAc (1.25 g, 15.20 mmol, 1.92 eq), NH_4Cl (1.24 g, 23.11 mmol, 2.92 eq) and powdered zinc (1.00 g, 15.20 mmol, 1.92 eq) were added. Vessel was then swiftly flushed with argon. Suspension was stirred for 3 h, at room temperature, under argon. Upon completion, reaction mixture was filtered through a short pad of Celite®, washing with EtOAc. Filtrate was concentrated *in vacuo* with silica. Dry residue was purified by flash chromatography (hexane/ethyl acetate, 9:1, (v/v) to hexane/ethyl acetate, 85:15,

(v/v)) yielding 3,4-di-*O*-acetyl-D-xylal **SI6** as white crystals (713 mg, 45%). The characterisation data were in agreement with those of the literature.[2]

***R**$_f$* = 0.49 (hexane/ethyl acetate, 8:2, (v/v)).

^{1}H NMR (600 MHz, $CDCl_3$) δ [ppm]: 6.59 (d, J = 6.5 Hz, 1H), 4.99 – 4.93 (m, 3H), 4.18 (ddd, J = 12.1, 3.4, 1.7 Hz, 1H), 3.96 (dd, J = 12.2, 1.8 Hz, 1H), 2.08 (s, 3H), 2.05 (s, 3H).

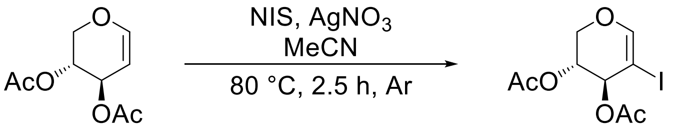

SI6 **1c**

A flame-dried flask, equipped with a stir bar and a condenser was evacuated and backfilled with argon 3 times (Schlenk technique). Next, 6.25 ml of freshly distilled MeCN (under argon, with CaH_2) was added under constant flow of argon. Solvent was preheated to 60 °C. After that, 3,4-di-*O*-acetyl-D-xylal **SI6** (550 mg, 2.75 mmol, 1.0 eq), $AgNO_3$ (68.0 mg, 0.55 mmol, 0.2 eq) and NIS (540 mg, 3.30 mmol, 1.2 eq) were added under constant flow of argon. Last portion of MeCN (2.0 ml) was then added and used to flush remains of substrates from flask walls. Mixture was stirred in darkness at 80 °C for 1 h. Upon completion, reaction mixture was filtrated through a short pad of Celite® and washed with EtOAc. Filtrate was concentrated to dryness with silica. Residue was purified by flash chromatography (hexane (until violet iodine was removed) to hexane/ethyl acetate, 8:2, (v/v)) yielding yellowish crystals of xylal **1c** (580 mg, 64%). The characterisation data were in agreement with those of the literature.[1]

***R**$_f$* = 0.45 (hexane/ethyl acetate, 85:15, (v/v)).

^{1}H NMR (600 MHz, $CDCl_3$) δ [ppm]: 6.92 (s, 1H), 5.27 – 5.25 (m, 1H), 4.97 (td, J = 2.6, 1.6 Hz, 1H), 4.30 (ddd, J = 12.4, 2.8, 1.7 Hz, 1H), 3.97 (dd, J = 12.4, 1.6 Hz, 1H), 2.14 (s, 3H), 2.11 (s, 2H).

3,4-di-O-acetyl-2-iodo-L-rhamnal (**1d**)

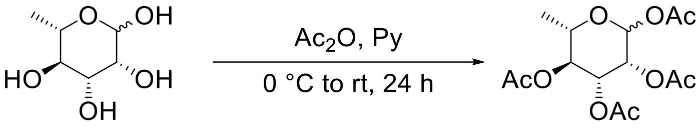

SI7 SI8

To a round-bottom flask, L-rhamnose **SI7** (5.08 g, 30.45 mmol, 1.0 eq) was added. Solid was suspended in pyridine (26 ml). Resulting mixture was cooled down to 0 °C (ice bath) and acetic anhydride (25.0 ml, 265 mmol, 8.7 eq) was added. Mixture was slowly warmed to room temperature and stirred for 24 h. Upon completion, reaction was quenched with 20 ml of distilled water. Next, mixture was transferred to a separatory funnel, where phases were separated. Aqueous phase was extracted with DCM (2x10 ml). Combined organic phases were washed with distilled water (20 ml) HCl (5%, wt.%, 2x20 ml, or until smell of pyridine was no longer noticeable) and with saturated aqueous $NaHCO_3$ solution (1x80 ml). Organic phase was dried over $MgSO_4$ (anh.). Resulting suspension was filtered and concentrated *in vacuo* yielding peracetylated L-rhamnose **SI8** as a pale-yellow oil (8.30 g, 82%). Obtained product was of sufficient quality and was used directly in the next step.

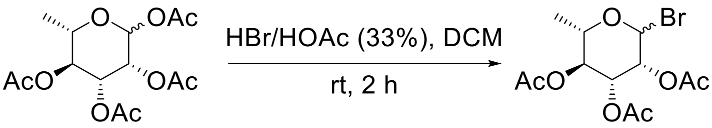

SI8 SI9

Peracetylated L-rhamnose **SI8** (8.30 g, 24.97 mmol, 1.0 eq) was dissolved in 84 ml of DCM in a round-bottom flask. To solution HBr/HOAc (33% wt.%) was slowly added (28.4 g, 21.0 ml, 116 mmol, 4.6 eq). Mixture was stirred for 2 h at room temperature. Upon completion, reaction mixture was diluted with 100 ml of ethyl acetate. Mixture was transferred to a separatory funnel, where phases were separated. Organic phase was washed with 2x100 ml of distilled water, 2x50 ml of $NaHCO_3$ solution (10% wt.%) and 150 ml of brine. Organic phase was dried over $MgSO_4$ (anh.). Resulting suspension was filtered and concentrated *in vacuo*. Crude **SI9** was used in the next step without further purification.

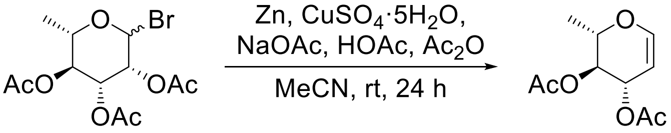

SI9 SI10

Crude peracetylated L-rhamnosyl bromide **SI9** (directly from previous step, 24.97 mmol, 1.0 eq) was dissolved in 76 ml of freshly dried MeCN (over CaH_2) in a round-bottom flask. Next, $CuSO_4{\cdot}5H_2O$ (875 mg, 3.50 mmol, 0.14 eq), NaOAc (4.05 g, 49.40 mmol, 1.92 eq), powdered zinc (3.65 g, 55.80 mmol, 2.23 eq), glacial acetic acid (3.2 ml) and acetic anhydride (3.7 ml) were added. Suspension was stirred for 24 h, at room temperature. Upon completion, reaction mixture was filtered through a short pad of Celite®, washing with EtOAc (220 ml). Filtrate was transferred to a separatory funnel. Organic phase was washed with 2x50 ml distilled water, 1x50 ml sat. aqueous $NaHCO_3$ solution and 1x50 ml brine. Organic phase was dried over $MgSO_4$ (anh.). Resulting suspension was filtered and concentrated *in vacuo*. Dry residue was purified by flash chromatography (hexane/ethyl acetate, 8:2, (v/v)) yielding 3,4-di-*O*-acetyl-L-rhamnal **SI10** as a pale-yellow oil (2.15 g, 40% (over 2 steps)). The characterisation data were in agreement with those of the literature.[3]

R_f = 0.30 (hexane/ethyl acetate, 8:2, (v/v)).

^{1}**H NMR** (500 MHz, $CDCl_3$) δ [ppm]: 6.41 (dd, J = 6.1, 1.5 Hz, 1H), 5.32 (dddd, J = 6.1, 3.1, 1.5, 0.6 Hz, 1H), 5.00 (dd, J = 8.2, 6.1 Hz, 1H), 4.75 (dd, J = 6.1, 3.0 Hz, 1H), 4.13 – 4.04 (m, 1H), 2.06 (s, 3H), 2.02 (s, 3H), 1.29 (d, J = 6.6 Hz, 3H).

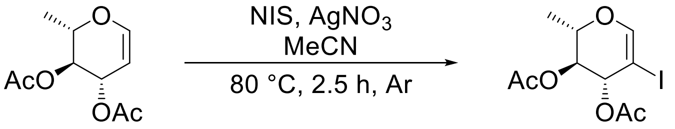

SI10 **1d**

A flame-dried flask, equipped with a stir bar and a condenser was evacuated and backfilled with argon 3 times (Schlenk technique). Next, 12.5 ml of freshly distilled MeCN (under argon, with CaH_2) was added under constant flow of argon. Solvent was preheated to 60 °C. After that, 3,4-di-*O*-acetyl-L-rhamnal **SI10** (1.33 g, 6.20 mmol, 1.0 eq), $AgNO_3$ (210.6 mg, 1.24 mmol, 0.2 eq) and NIS (1.67 g, 7.44 mmol, 1.2 eq) were added under constant flow of argon. Last portion of MeCN (6.0 ml) was then added and used to flush remains of substrates from flask walls. Mixture was stirred in darkness at 80 °C for 2.5 h. Upon completion, reaction mixture was filtrated through a short pad of Celite® and washed with EtOAc. Filtrate was concentrated to dryness with silica. Residue was purified by flash chromatography (hexane (until violet iodine was removed) to hexane/ethyl acetate, 85:15, (v/v)) yielding yellowish oily crystals of rhamnal **1d** (1.41 g, 67%). The characterisation data were in agreement with those of the literature.[4]

$\boldsymbol{R_f}$ = 0.45 (hexane/ethyl acetate, 85:15, (v/v)).

^{1}**H NMR** (600 MHz, $CDCl_3$) δ [ppm]: 6.76 (d, J = 1.2 Hz, 1H), 5.48 (ddd, J = 5.3, 1.3, 0.8 Hz, 1H), 5.03 (dd, J = 7.3,5.3 Hz, 1H), 4.26 (p, J = 7.3 Hz, 1H), 2.11 (s, 3H), 2.08 (s, 3H), 1.32 (d, J = 6.7 Hz, 3H).

2.2 Synthesis and characterisation of 2-trimethylsilylethynylated glycals (**2a-d**)

3,4,6-tri-O-acetyl-2-(2-trimethylsilylethynyl)-D-glucal (**2a**)

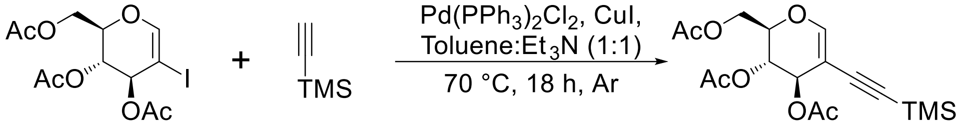

1a **2a**

To a sealed tube, glucal **1a** (500 mg, 1.26 mmol, 1.0 eq), $Pd(PPh_3)_2Cl_2$ (44.1 mg, 0.063 mmol, 0.05 eq), CuI (24.0 mg, 0.126 mmol, 0.1 eq) and toluene (7.0 ml) were added. The vessel was equipped with a stir bar and flushed with argon. Ethynyltrimethylsilane (0.21 ml, 148 mg, 1.51 mmol, 1.2 eq) and triethylamine (7.0 ml) were added subsequently through a rubber septum, under argon. Vessel was tightly sealed and the mixture was stirred at 70 °C for 18 h. Upon completion, reaction vessel was washed with $CHCl_3$ and crude mixture was transferred to a flask. The mixture was concentrated to dryness with silica. Residual solid was purified by flash chromatography (hexane/ethyl acetate, 7:3, (v/v)) yielding yellowish oil of glucal **2a** (432 mg, 93%). The characterisation data were in agreement with those of the literature.[5]

R_f = 0.40 (hexane/ethyl acetate, 7:3, (v/v)).

^{1}H NMR (500 MHz, $CDCl_3$) δ [ppm]: 6.88 (d, J = 1.1 Hz, 1H), 5.55 – 5.53 (m, 1H), 5.18 (dd, J = 7.4, 5.7 Hz, 1H), 4.39 (dd, J = 12.2, 5.9 Hz, 1H), 4.32 – 4.27 (m, 1H), 4.18 (dd, J = 12.2, 3.2 Hz, 1H), 2.09 (s, 3H), 2.08 (s, 3H), 2.07 (s, 3H), 0.15 (s, 9H).

3,4,6-tri-O-acetyl-2-(2-trimethylsilylethynyl)-D-galactal (**2b**)

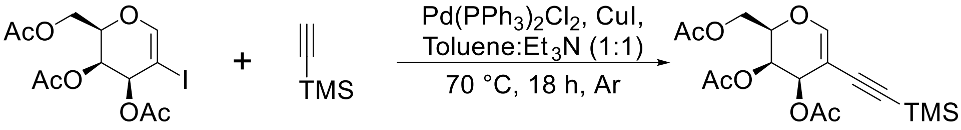

1b **2b**

To a sealed tube, galactal **1b** (941 mg, 2.36 mmol, 1.0 eq), $Pd(PPh_3)_2Cl_2$ (82.8 mg, 0.118 mmol, 0.05 eq), CuI (44.9 mg, 0.236 mmol, 0.1 eq) and toluene (13.0 ml) were added. The vessel was equipped with a stir bar and flushed with argon. Ethynyltrimethylsilane (0.39 ml, 279 mg, 2.84 mmol, 1.2 eq) and triethylamine (13.0 ml) were added subsequently through a rubber septum, under argon. Vessel was tightly sealed and the mixture was stirred at 70 °C for 18 h. Upon completion, reaction vessel was washed with $CHCl_3$ and crude mixture was transferred to a flask. The mixture was concentrated to dryness with silica. Residual solid was purified by flash chromatography (hexane/ethyl acetate, 7:3, (v/v)) yielding pale-yellow crystals of galactal **2b** (795 mg, 92%). The characterisation data were in agreement with those of the literature.[6]

R_f = 0.32 (hexane/ethyl acetate, 7:3, (v/v)).

^{1}H NMR (600 MHz, $CDCl_3$) δ [ppm]: 6.88 (d, J = 1.6 Hz, 1H), 5.67 (ddd, J = 4.5, 1.6, 1.0 Hz, 1H), 5.43 (dd, J = 4.5, 1.8 Hz, 1H), 4.34 – 4.30 (m, 1H), 4.28 – 4.15 (m, 2H), 2.13 (s, 3H), 2.08 (s, 3H), 2.06 (s, 3H), 0.16 (s, 9H).

3,4-di-O-acetyl-2-(2-trimethylsilylethynyl)-D-xylal (**2c**)

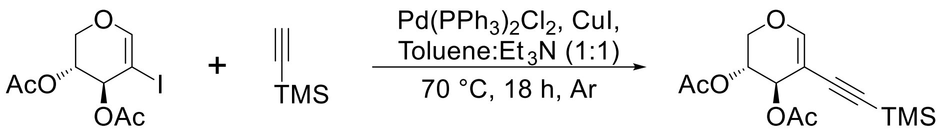

1c **2c**

To a sealed tube, xylal **1c** (580 mg, 1.78 mmol, 1.0 eq), $Pd(PPh_3)_2Cl_2$ (62.4 mg, 0.090 mmol, 0.05 eq), CuI (32.1 mg, 0.180 mmol, 0.1 eq) and toluene (10.0 ml) were added. The vessel was equipped with a stir bar and flushed with argon. Ethynyltrimethylsilane (0.29 ml, 210 mg, 2.14 mmol, 1.2 eq) and triethylamine (10.0 ml) were added subsequently through a rubber septum, under argon. Vessel was tightly sealed and the mixture was stirred at 70 °C for 18 h. Upon completion, reaction vessel was washed with $CHCl_3$ and crude mixture was transferred to a flask. The mixture was concentrated to dryness with silica. Residual solid was purified by flash chromatography (hexane/ethyl acetate, 7:3, (v/v)) yielding yellowish oil of xylal **2c** (485 mg, 91%).

$\boldsymbol{R_f}$ = 0.63 (hexane/ethyl acetate, 7:3, (v/v)), $[\boldsymbol{\alpha}]_{\boldsymbol{D}}^{\mathbf{20}}$= −269.5 (0.50, $CHCl_3$).

^{1}H NMR (600 MHz, $CDCl_3$) δ [ppm]: 7.02 (br s, 1H, H-1$_{glycal}$), 5.26 (dd, J = 2.9, 1.7 Hz, 1H, H-3$_{glycal}$), 4.91 – 4.90 (m, 1H, H-4$_{glycal}$), 4.23 (ddd, J = 12.3, 3.0, 1.8 Hz, 1H, H-5$_{glycal}$), 3.92 (dd, J = 12.4, 1.8 Hz, 1H, H-5'$_{glycal}$) 2.11 (s, 3H, C$\underline{H}_3$COO-), 2.08 (s, 3H, C$\underline{H}_3$COO-), 0.16 (s, 9H, (C$\underline{H}_3$)$_3$Si-).

^{13}C NMR (151 MHz, $CDCl_3$) δ [ppm]: 169.7, 169.5, 153.9, 100.8, 95.5, 94.3, 66.4, 64.9, 63.7, 20.9, 20.9, -0.05.

HRMS (ESI+) *m/z*: $[M+H]^+$ calcd for $C_{14}H_{21}O_5Si$ 297.1153; found 297.1153.

3,4-di-O-acetyl-2-(2-trimethylsilylethynyl)-L-rhamnal (**2d**)

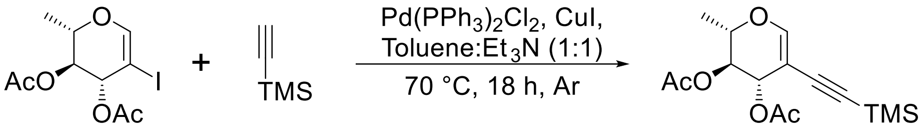

1d **2d**

To a sealed tube, rhamnal **1d** (500 mg, 1.47 mmol, 1.0 eq), $Pd(PPh_3)_2Cl_2$ (51.9 mg, 0.074 mmol, 0.05 eq), CuI (28.0 mg, 0.147 mmol, 0.1 eq) and toluene (7.8 ml) were added. The vessel was equipped with a stir bar and flushed with argon. Ethynyltrimethylsilane (0.25 ml, 174 mg, 1.77 mmol, 1.2 eq) and triethylamine (7.8 ml) were added subsequently through a rubber septum, under argon. Vessel was tightly sealed and the mixture was stirred at 70 °C for 18 h. Upon completion, reaction vessel was washed with $CHCl_3$ and crude mixture was transferred to a flask. The mixture was concentrated to dryness with silica. Residual solid was purified by flash chromatography (hexane/ethyl acetate, 85:15, (v/v)) yielding yellowish oil of rhamnal **2d** (405 mg, 89%).

$\boldsymbol{R_f}$ = 0.59 (hexane/ethyl acetate, 7:3, (v/v)), $[\alpha]_D^{20}$= −14.9 (1.00, $CHCl_3$).

^{1}H NMR (500 MHz, $CDCl_3$) δ [ppm]: 6.85 (d, *J* = 1.2 Hz, 1H, H-1_{glycal}), 5.52 (ddd, *J* = 5.9, 1.2, 0.7 Hz, 1H, H-3_{glycal}), 4.97 (dd, *J* = 7.6, 5.9 Hz, 1H, H-4_{glycal}), 4.19 – 4.12 (m, 1H, H-5_{glycal}), 2.07 (s, 3H, C$\underline{H}_3$COO-), 2.07 (s, 3H, C$\underline{H}_3$COO-), 1.31 (d, *J* = 6.6 Hz, 3H, C$\underline{H}_3$-), 0.14 (s, 9H, (C$\underline{H}_3$)$_3$Si-).

^{13}C NMR (126 MHz, $CDCl_3$) δ [ppm]: 170.1, 169.7, 152.0, 99.5, 96.8, 94.9, 73.1, 70.8, 67.7, 20.9, 20.8, 16.4, -0.1.

HRMS (ESI+) *m/z*: $[M+H]^+$ calcd for $C_{15}H_{23}O_5Si$ 311.1309; found 311.1304.

2.3 Synthesis and characterisation of di(pyrrol-2-yl)methane

di(1H-pyrrol-2-yl)methane (**3**)

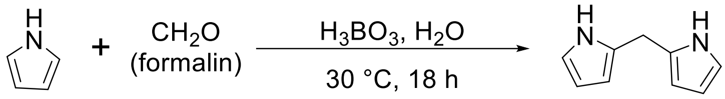

3

To a round-bottom flask, distilled water (20 ml), H_3BO_3 (247.3 mg, 4.00 mmol, 0.2 eq) and formaldehyde (37% wt.%, 1.5 ml, 20.2 mmol, 1.0 eq) were added. Next, reaction vessel was equipped with a stir bar and heated to 30 °C. Then, pyrrole (7.0 ml, 6.76 g, 100.8 mmol, 5 eq) was added dropwise over a period of 30 minutes. Reaction vessel was protected from light and mixture was stirred for 18 h. Upon completion, reaction mixture was transferred to a separatory funnel and washed with DCM (3x25 ml). Organic phases were combined and concentrated to dryness with generous amount of silica. Extra care was taken to make sure that temperature and pressure does not exceed 50 °C and 0.1 bar (to protect product **3** from evaporating). Residual solid was purified by flash chromatography (hexane/dichloromethane, 1:1, (v/v) to pure dichloromethane) yielding **3** as a white solid (720.2 mg, 24%). The characterisation data were in agreement with those of the literature.[7]

$\boldsymbol{R_f}$= 0.44 (dichloromethane).

^{1}H NMR (600 MHz, $CDCl_3$) δ [ppm]: 7.70 (br s, 2H), 6.66 – 6.61 (m, 1H), 6,18 (dd, J = 5.7, 2.8 Hz, 1H), 6,08 – 6,04 (m, 2H), 3,95 (s, 2H).

2.4 Synthesis and characterisation of *trans*-A_2B_2 porphyrins (**4a-h**)

5,15-diphenylporphyrin (**4a**)

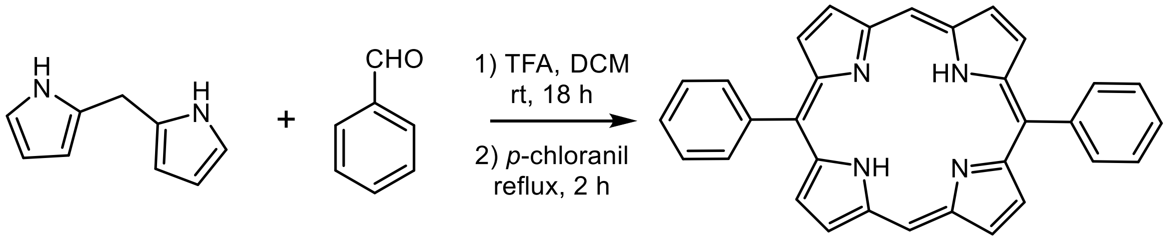

3 **4a**

To a round-bottom flask equipped with a stir bar dichloromethane (240 ml), **3** (200 mg, 1.37 mmol, 1.0 eq) and benzaldehyde (0.14 ml, 145 mg, 1.37 mmol, 1.0 eq) were added. Next, stirring was started and solution of TFA in DCM (12 mg/ml, 0.105 mol/dm^3) was added (8.0 ml, 96 mg, 0.84 mmol, 0.61 eq) dropwise. Reaction was protected from light and stirred at room temperature for 18 h. After that, *p*-chloranil (505 mg, 2.05 mmol, 1.5 eq) was added and temperature was raised to reflux for 2 h. Upon completion, triethylamine (1.0 ml) was added to the reaction vessel. Reaction mixture was then concentrated to dryness with silica. Residual solid was purified by flash chromatography (hexane/dichloromethane, 1:1, (v/v)) yielding 5,15-diphenylporphyrin **4a** (183 mg, 58%) as a violet crystalline solid. If *p*-chloranil was detected in the product by TLC, it would be washed with MeOH, affording pure porphyrin after drying. The characterisation data were in agreement with those of the literature.[8]

R$_f$ = 0.41 (hexane/dichloromethane, 1:1, (v/v)).

^{1}H NMR (600 MHz, $CDCl_3$) δ [ppm]: 10.32 (br s, 2H), 9.40 (d, *J* = 4.5 Hz, 4H), 9.09 (d, *J* = 4.5 Hz, 4H), 8.32 – 8.26 (m, 4H), 7.84 – 7.80 (m, 6H), -3.09 (br s, 2H).

di-tert-butyl 4,4'-(porphyrin-5,15-diyl)dibenzoate (**4b**)

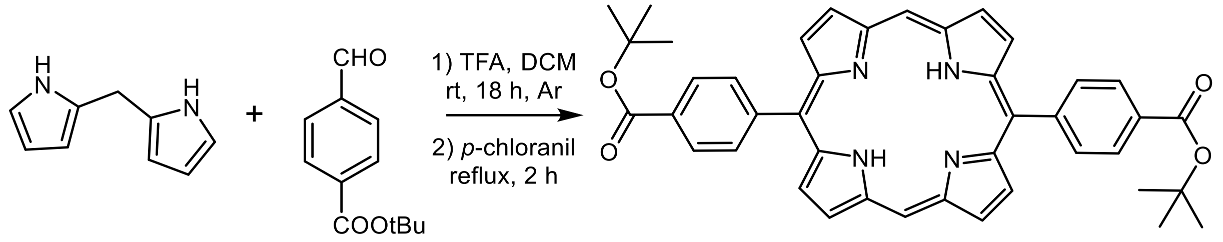

3 **4b**

To a round-bottom flask equipped with a stir bar dichloromethane (160 ml), **3** (100 mg, 0.684 mmol, 1.0 eq) and benzaldehyde (141 mg, 0.684 mmol, 1.0 eq) were added. Next, stirring was started and solution was degassed with argon for 30 min. After that, solution of TFA in DCM (12 mg/ml, 0.105 mol/dm^3) was added (4.0 ml, 48 mg, 0.42 mmol, 0.61 eq) dropwise to the reaction vessel. Reaction was protected from light and stirred at room temperature for 18 h. After that, *p*-chloranil (253 mg, 1.03 mmol, 1.5 eq) was added and temperature was raised to reflux for 2 h. Upon completion, triethylamine (0.5 ml) was added to the reaction vessel. Reaction mixture was then concentrated to dryness with silica. Residual solid was purified by flash chromatography (hexane/dichloromethane, 1:1, (v/v) to pure dichloromethane) yielding di-*tert*-butyl 4,4'-(porphyrin-5,15-diyl)dibenzoate **4b** (111.2 mg, 49%) as a violet crystalline solid. If *p*-chloranil was detected in the product by TLC, it would be washed with MeOH, affording pure porphyrin after drying.

R_f = 0.52 (dichloromethane), mp: > 220 °C.

^{1}H NMR (600 MHz, $CDCl_3$) δ [ppm]: 10.33 (s, 2H, H_{meso}), 9.41 (d, J = 4.6 Hz, 4H, H_β), 9.04 (d, J = 4.5 Hz, 4H, H_β), 8.47 – 8.44 (m, 4H, H-2,2'$_{aryl}$), 8.35 – 8.33 (m, 4H, H-3,3'$_{aryl}$), 1.80 (s, 18H, -C($C\underline{H}_3$)$_3$), -3.13 (br s, 2H, NH).

^{13}C NMR (151 MHz, $CDCl_3$) δ [ppm]: 166.2, 146.9, 145.8, 145.5, 134.9, 132.1, 131.6, 131.0, 128.2, 118.3, 105.8, 81.6, 28.6.

UV-Vis ($CHCl_3$) λ_{max} [nm] (log ε): 631.1 (3.35), 575.1 (3.78), 538.1 (3.85), 503.3 (4.21), 408.8 (5.55, Soret band).

MS (ESI+, isotopic profile) *m/z* (% rel. int.): $[M+H]^+$ calcd for $C_{42}H_{39}N_4O_4$ 663.3; found 666.3 (2), 665.3 (12), 664.3 (48), 663.3 (100).

HRMS (ESI+) *m/z*: $[M+H]^+$ calcd for $C_{42}H_{39}N_4O_4$ 663.2966; found 663.2965.

5,15-bis(3,5-dimethoxyphenyl)porphyrin (**4c**)

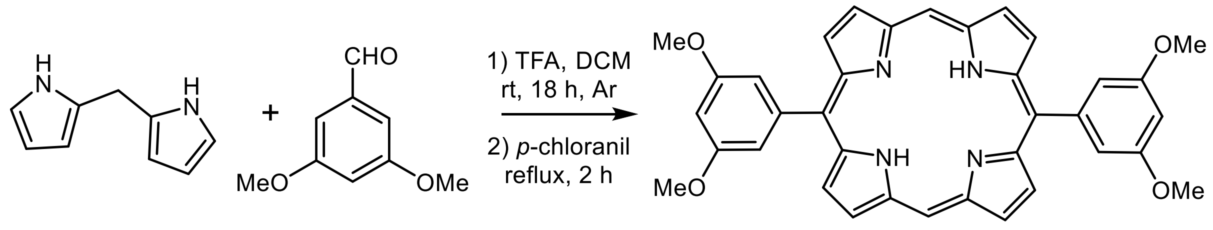

3 **4c**

To a round-bottom flask equipped with a stir bar dichloromethane (120 ml), **3** (100 mg, 0.684 mmol, 1.0 eq) and benzaldehyde (114 mg, 0.684 mmol, 1.0 eq) were added. Next, stirring was started and solution was degassed with argon for 30 min. After that, solution of TFA in DCM (12 mg/ml, 0.105 mol/dm^3) was added (4.0 ml, 48 mg, 0.42 mmol, 0.61 eq) dropwise to the reaction vessel. Reaction was protected from light and stirred at room temperature for 18 h. After that, *p*-chloranil (253 mg, 1.03 mmol, 1.5 eq) was added and temperature was raised to reflux for 2 h. Upon completion, triethylamine (0.5 ml) was added to the reaction vessel. Reaction mixture was then concentrated to dryness with silica. Residual solid was purified by flash chromatography (hexane/chloroform, 1:1, (v/v) to pure chloroform) yielding 5,15-bis(3,5-dimethoxyphenyl)porphyrin **4c** (99.2 mg, 50%) as a violet crystalline solid. If *p*-chloranil was detected in the product by TLC, it would be washed with MeOH, affording pure porphyrin after drying. The characterisation data were in agreement with those of the literature.[9]

R_f = 0.53 (chloroform).

^{1}H NMR (600 MHz, $CDCl_3$) δ [ppm]: 10.31 (br s, 2H), 9.39 (d, J = 4.5 Hz, 4H), 9.18 (d, J = 4.5 Hz, 4H), 7.46 (d, J = 2.3 Hz, 4H), 6.93 (t, J = 2.3 Hz, 2H), 4.00 (s, 12H), -3.15 (br s, 2H).

5,15-bis(4-(tert-butyl)phenyl)porphyrin (**4d**)

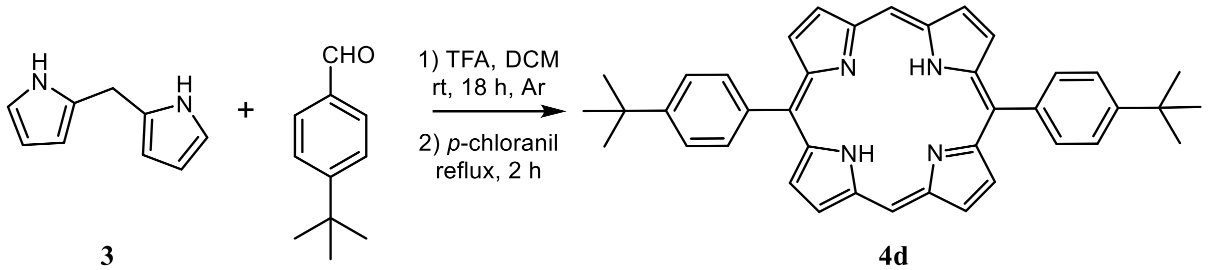

3 **4d**

To a round-bottom flask equipped with a stir bar dichloromethane (160 ml), **3** (100 mg, 0.684 mmol, 1.0 eq) and benzaldehyde (0.11 ml, 111 mg, 0.684 mmol, 1.0 eq) were added. Next, stirring was started and solution was degassed with argon for 30 min. After that, solution of TFA in DCM (12 mg/ml, 0.105 mol/dm^3) was added (4.0 ml, 48 mg, 0.42 mmol, 0.61 eq) dropwise to the reaction vessel. Reaction was protected from light and stirred at room temperature for 18 h. After that, *p*-chloranil (253 mg, 1.03 mmol, 1.5 eq) was added and temperature was raised to reflux for 2 h. Upon completion, triethylamine (0.5 ml) was added to the reaction vessel. Reaction mixture was then concentrated to dryness with silica. Residual solid was purified by flash chromatography (hexane/dichloromethane, 1:1, (v/v) to pure dichloromethane to dichloromethane/chloroform, 1:1, (v/v)) yielding, after crystallisation (chloroform and methanol mixture), 5,15-bis(4-(*tert*-butyl)phenyl)porphyrin **4d** (44.5 mg, 24%) as a violet crystalline solid. The characterisation data were in agreement with those of the literature.[10]

$\boldsymbol{R_f}$ = 0.48 (hexane/dichloromethane, 1:1, (v/v)).

^{1}H NMR (600 MHz, $CDCl_3$) δ [ppm]: 10.31 (s, 2H), 9.40 (d, *J* = 4.6 Hz, 4H), 9.13 (d, *J* = 4.6 Hz, 4H), 8.25 – 8.19 (m, 4H), 7.86 – 7.80 (m, 4H), 1.65 (s, 18H), -3.05 (br s, 2H).

5,15-bis(5-ethylthiophen-2-yl)porphyrin (**4e**)

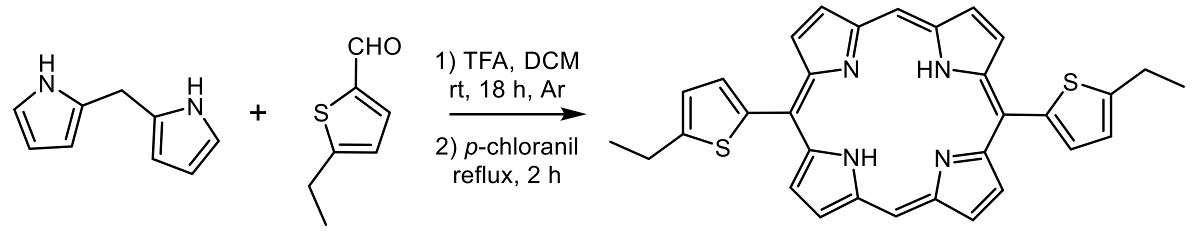

3 **4e**

To a round-bottom flask equipped with a stir bar dichloromethane (160 ml), **3** (100 mg, 0.684 mmol, 1.0 eq) and 5-ethylthiophene-2-carbaldehyde (0.09 ml, 96 mg, 0.684 mmol, 1.0 eq) were added. Next, stirring was started and solution was degassed with argon for 30 min. After that, solution of TFA in DCM (12 mg/ml, 0.105 mol/dm^3) was added (4.0 ml, 48 mg, 0.42 mmol, 0.61 eq) dropwise to the reaction vessel. Reaction was protected from light and stirred at room temperature for 18 h. After that, *p*-chloranil (253 mg, 1.03 mmol, 1.5 eq) was added and temperature was raised to reflux for 2 h. Upon completion, triethylamine (0.5 ml) was added to the reaction vessel. Reaction mixture was then concentrated to dryness with silica. Residual solid was purified by flash chromatography (hexane/chloroform, 1:1, (v/v)) yielding 5,15-bis(5-ethylthiophen-2-yl)porphyrin **4e** (48.7 mg, 27%) as a violet crystalline solid. If *p*-chloranil was detected in the product by TLC, it would be washed with MeOH, affording pure porphyrin after drying.

$\boldsymbol{R_f}$ = 0.50 (hexane/chloroform, 1:1, (v/v)), mp: > 220 °C.

^{1}H NMR (600 MHz, $CDCl_3$) δ [ppm]: 10.28 (s, 2H, H_{meso}), 9.37 (q, *J* = 4.5 Hz, 8H, H_β), 7.75 (d, *J* = 3.2 Hz, 2H, H-3_{aryl}), 7.24 (d, *J* = 3.3 Hz, 2H, H-4_{aryl}), 3.22 (q, *J* = 7.5 Hz, 4H, -C$\underline{H}_2$-), 1.62 (t, *J* = 7.5 Hz, 6H, -C$\underline{H}_3$), -2.95 (br s, 2H, NH).

^{13}C NMR (151 MHz, $CDCl_3$) δ [ppm]: 133.7, 131.8, 131.2, 123.2, 106.0, 23.9, 16.3.

UV-Vis ($CHCl_3$) λ_{max} [nm] (log ε): 638.2 (3.56), 581.0 (3.73), 547.9 (4.03), 509.8 (4.10), 415.8 (5.42, Soret band).

MS (ESI+, isotopic profile) *m/z* (% rel. int.): $[M+H]^+$ calcd for $C_{32}H_{27}N_4S_2$ 531.2; found 534.2 (3), 533.2 (7), 532.2 (36), 531.2 (100).

HRMS (ESI+) *m/z*: $[M+H]^+$ calcd for $C_{32}H_{27}N_4S_2$ 531.1672; found 531.1672.

5,15-bis(4-chlorophenyl)porphyrin (**4f**)

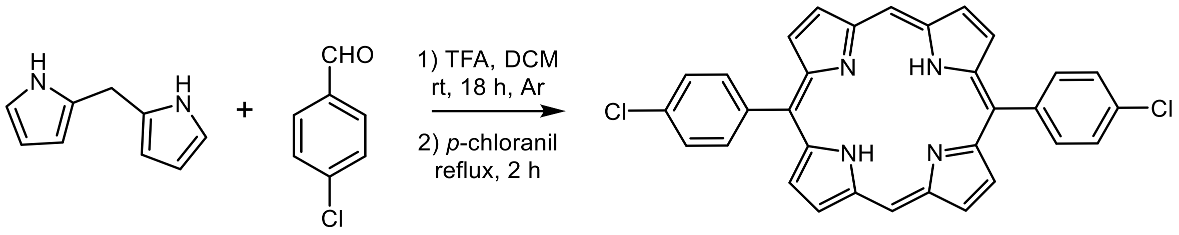

3 **4f**

To a round-bottom flask equipped with a stir bar dichloromethane (240 ml), **3** (150 mg, 1.026 mmol, 1.0 eq) and benzaldehyde (0.12 ml, 190 mg, 1.026 mmol, 1.0 eq) were added. Next, stirring was started and solution was degassed with argon for 30 min. After that, solution of TFA in DCM (12 mg/ml, 0.105 mol/dm^3) was added (6.0 ml, 72 mg, 0.63 mmol, 0.61 eq) dropwise to the reaction vessel. Reaction was protected from light and stirred at room temperature for 18 h. After that, *p*-chloranil (378 mg, 1.54 mmol, 1.5 eq) was added and temperature was raised to reflux for 2 h. Upon completion, triethylamine (1.0 ml) was added to the reaction vessel. Reaction mixture was then concentrated to dryness with silica. Residual solid was purified by flash chromatography (hexane/dichloromethane, 1:1, (v/v)) yielding 5,15-bis(4-chlorophenyl)porphyrin **4f** (108.7 mg, 34%) as a violet crystalline solid. The characterisation data were in agreement with those of the literature.[11]

$\boldsymbol{R_f}$ = 0.53 (hexane/dichloromethane, 1:1, (v/v)).

^{1}H NMR (600 MHz, $CDCl_3$) δ [ppm]: 10.34 (s, 2H), 9.42 (d, *J* = 4.3 Hz, 4H), 9.06 (d, *J* = 4.7 Hz, 4H), 8.22 – 8.20 (m, 4H), 7.82 – 7.79 (m, 4H).

5,15-bis(3-bromophenyl)porphyrin (**4g**)

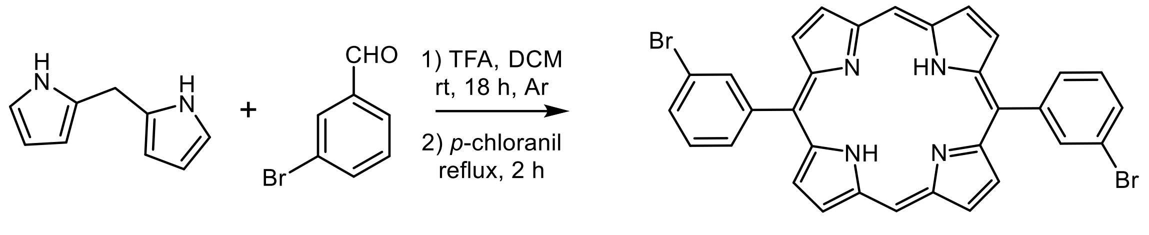

3 **4g**

To a round-bottom flask equipped with a stir bar dichloromethane (160 ml), **3** (100 mg, 0.684 mmol, 1.0 eq) and benzaldehyde (96 mg, 0.684 mmol, 1.0 eq) were added. Next, stirring was started and solution was degassed with argon for 30 min. After that, solution of TFA in DCM (12 mg/ml, 0.105 mol/dm^3) was added (4.0 ml, 48 mg, 0.42 mmol, 0.61 eq) dropwise to the reaction vessel. Reaction was protected from light and stirred at room temperature for 18 h. After that, *p*-chloranil (253 mg, 1.03 mmol, 1.5 eq) was added and temperature was raised to reflux for 2 h. Upon completion, triethylamine (0.5 ml) was added to the reaction vessel. Reaction mixture was then concentrated to dryness with silica. Residual solid was purified by flash chromatography (hexane/dichloromethane, 1:1, (v/v)) yielding, 5,15-bis(3-bromophenyl)porphyrin **4g** (97,5 mg, 54%) as a violet crystalline solid. The characterisation data were in agreement with those of the literature.[12]

R$_f$ = 0,51 (hexane/dichloromethane, 1:1, (v/v)).

^{1}H NMR (600 MHz, $CDCl_3$) δ [ppm]: 10.36 (s, 2H), 9.43 (d, *J* = 4.5 Hz, 4H), 9.07 (d, *J* = 4.5 Hz, 4H), 8.45 (br s, 2H), 8.12 (d, *J* = 7.4 Hz, 2H), 7.92 (ddd, *J* = 8.4, 1.9, 1.0 Hz, 2H), 7.69 (dd, *J* = 8.2, 7.5 Hz, 2H), -3.18 (br s, 2H).

5,15-bis(3-cyanophenyl)porphyrin (**4h**)

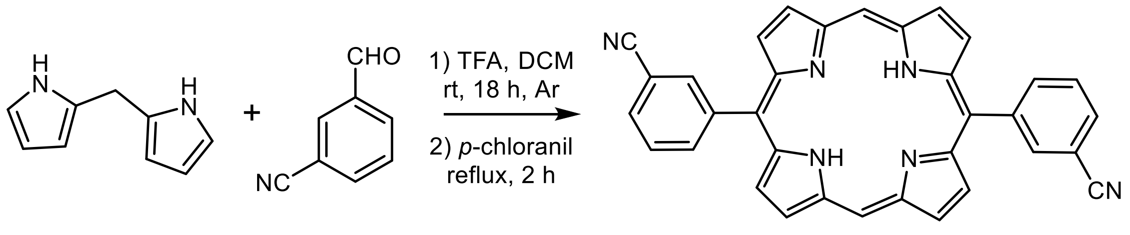

3 **4h**

To a round-bottom flask equipped with a stir bar dichloromethane (120 ml), **3** (100 mg, 0.684 mmol, 1.0 eq) and benzaldehyde (90 mg, 0.684 mmol, 1.0 eq) were added. Next, stirring was started and solution was degassed with argon for 30 min. After that, solution of TFA in DCM (12 mg/ml, 0.105 mol/dm^3) was added (4.0 ml, 48 mg, 0.42 mmol, 0.61 eq) dropwise to the reaction vessel. Reaction was protected from light and stirred at room temperature for 18 h. After that, *p*-chloranil (253 mg, 1.03 mmol, 1.5 eq) was added and temperature was raised to reflux for 2 h. Upon completion, triethylamine (0.5 ml) was added to the reaction vessel. Reaction mixture was then concentrated to dryness with silica. Residual solid was purified by flash chromatography (hexane/dichloromethane, 1:1, (v/v) to pure dichloromethane) yielding 5,15-bis(3-cyanophenyl)porphyrin **4h** (63.8 mg, 36%) as a violet crystalline solid. If *p*-chloranil was detected in the product by TLC, it would be washed with MeOH, affording pure porphyrin after drying.

R_f = 0.49 (dichloromethane), mp: > 220 °C.

^{1}H NMR (500 MHz, $CDCl_3$) δ [ppm]: 10.40 (s, 2H, H_{meso}), 9.47 (d, J = 4.6 Hz, 4H, H_β), 8.98 (d, J = 4.6 Hz, 4H, H_β), 8.58 (d, J = 1.4 Hz, 2H, H-2_{aryl}), 8.52 (d, J = 7.6 Hz, 2H, H-4_{aryl}), 8.16 – 8.13 (m, 2H, H-6_{aryl}), 7.96 (t, J = 7.8 Hz, 1H, H-5_{aryl}), -3.05 (br s, 2H, NH).

^{13}C NMR (151 MHz, $CDCl_3$) δ [ppm]: 143.3, 142.8, 132.5, 131.7, 130.6, 128.1, 118.9, 116.4, 106.1.

UV-Vis ($CHCl_3$) λ_{max} [nm] (log ε): 629.0 (3.37), 575.0 (3.75), 535.8 (3.78), 501.9 (4.24), 407.5 (5.49, Soret band).

MS (ESI+) *m/z* (% rel. int.): 515.2 (7), 514.2 (36), 513.2 (100).

HRMS (ESI+): $C_{34}H_{21}N_6$ $[M+H]^+$ *m/z* calc. 513.1822, found 513.1820.

MS (ESI+, isotopic profile) *m/z* (% rel. int.): $[M+H]^+$ calcd for $C_{34}H_{21}N_6$ 513.2; found 515.2 (7), 514.2 (36), 513.2 (100).

HRMS (ESI+) *m/z*: $[M+H]^+$ calcd for $C_{34}H_{21}N_6$ 513.1822; found 513.1820.

2.5 Synthesis and characterisation of dibrominated *trans*-A_2B_2 porphyrins (**5a-h**)

Bromination of *trans*-A_2B_2 porphyrins (**4a-h**) was carried out with some modification of the known protocol.[13] For porphyrins exhibiting very low solubility in pure chloroform, we observed incomplete reaction. This issue was resolved by adding a small amount of methanol (ca. 10%) to the reaction mixture, which results in near-quantitative conversion.

5,15-dibromo-10,20-diphenylporphyrin (**5a**)

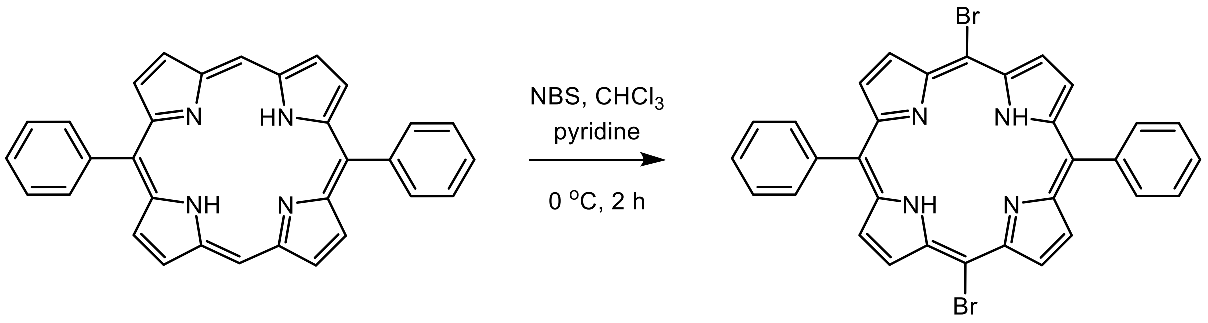

4a **5a**

To a round-bottom flask, porphyrin **4a** (262 mg, 0.566 mmol, 1.0 eq), chloroform (250 ml) and pyridine (2.03 ml) were added. Resulting solution was placed in an ice bath. Next, NBS (212 mg, 1.19 mmol, 2.1 eq) was added and mixture was stirred for 2 h in around 0 °C. After that, reaction mixture was concentrated to dryness (making sure, that no smell of pyridine is present after the evaporation). Residual solid was then suspended in methanol and filtrated through G3 fritted glass funnel. Next, porphyrin sediment was washed with methanol 3 times. The residue was dissolved in chloroform and concentrated. After drying, dibrominated porphyrin **5a** (350 mg, 99%) was obtained as a violet-red crystalline solid. The characterisation data were in agreement with those of the literature.[14]

R_f= 0.64 (hexane/chloroform, 1:1, (v/v)).

^{1}H NMR (600 MHz, $CDCl_3$) δ [ppm]: 9.62 (d, *J* = 4.7 Hz, 4H), 8.84 (br s, 4H), 8.18 – 8.15 (m, 4H), 7.84 – 7.75 (m,6H), -2.71 (br s, 2H).

di-tert-butyl 4,4'-(10,20-dibromoporphyrin-5,15-diyl)dibenzoate (**5b**)

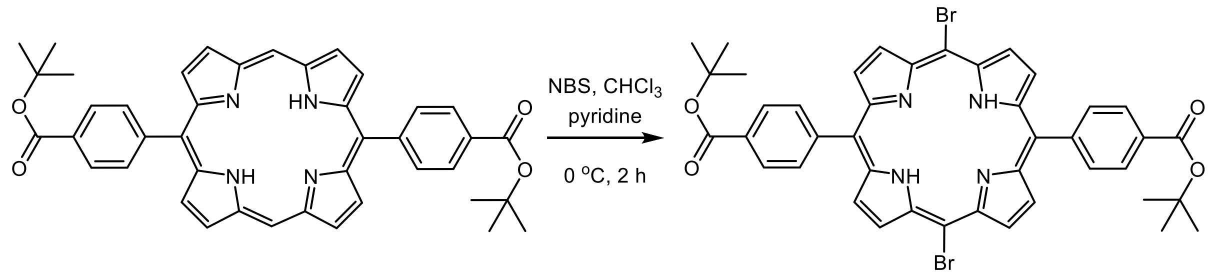

4b **5b**

To a round-bottom flask, porphyrin **4b** (111 mg, 0.168 mmol, 1.0 eq), chloroform (78 ml) and pyridine (0.60 ml) were added. Resulting solution was placed in an ice bath. Next, NBS (63 mg, 0.354 mmol, 2.1 eq) was added and mixture was stirred for 2 h in around 0 °C. After that, reaction mixture was concentrated to dryness (making sure, that no smell of pyridine is present after the evaporation). Residual solid was then suspended in methanol and filtrated through G3 fritted glass funnel. Next, porphyrin sediment was washed with methanol 3 times. The residue was dissolved in chloroform and concentrated. After drying, dibrominated porphyrin **5b** (136 mg, 99%) was obtained as a violet-red crystalline solid.

$\boldsymbol{R_f}$ = 0.62 (dichloromethane), mp: > 220 °C.

^{1}H NMR (600 MHz, $CDCl_3$) δ [ppm]: 9.62 (d, J = 4.8 Hz, 4H, H_β), 8.78 (d, J = 4.9 Hz, 4H, H_β), 8.43 – 8.39 (m, 4H, H-2,2'$_{aryl}$), 8.23 – 8.20 (m, 4H, H-3,3'$_{aryl}$), 1.78 (s, 18H, -C($\underline{H}_3$)$_3$), -2.75 (br s, 2H, NH).

^{13}C NMR (151 MHz, $CDCl_3$) δ [ppm]: 166.0, 145.7, 134.5, 132.0, 128.0, 120.5, 104.2, 81.7, 28.5.

UV-Vis ($CHCl_3$) λ_{max} [nm] (log ε): 658.1 (3.71), 601.3 (3.73), 556.6 (4.06), 521.4 (4.22), 422.7 (5.47, Soret band).

MS (ESI+, isotopic profile) *m/z* (% rel. int.): $[M+H]^+$ calcd for $C_{42}H_{37}Br_2N_4O_4$ 819.1; found 826.1 (1), 825.1 (6), 824.1 (24), 823.1 (49), 822.1 (46), 821.1 (100), 820.1 (25), 819.1 (52).

HRMS (ESI+) *m/z*: $[M+H]^+$ calcd for $C_{42}H_{37}Br_2N_4O_4$ 819.1176; found 819.1178.

di-tert-butyl 4,4'-(10,20-dibromoporphyrin-5,15-diyl)dibenzoate (**5c**)

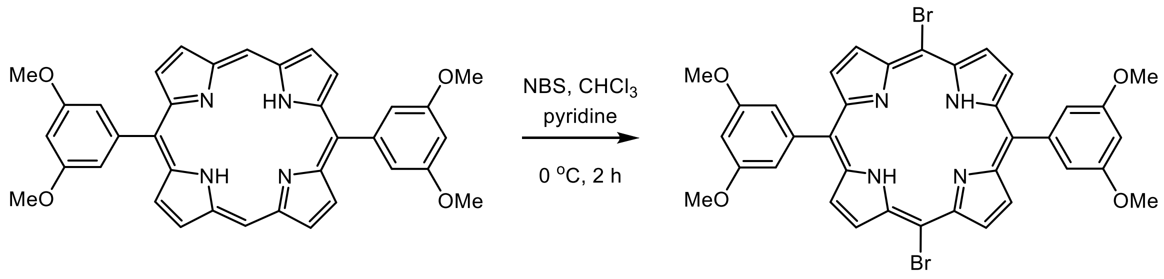

4c **5c**

To a round-bottom flask, porphyrin **4c** (99 mg, 0.170 mmol, 1.0 eq), chloroform (80 ml) and pyridine (0.61 ml) were added. Resulting solution was placed in an ice bath. Next, NBS (64 mg, 0.357 mmol, 2.1 eq) was added and mixture was stirred for 2 h in around 0 °C. After that, reaction mixture was concentrated to dryness (making sure, that no smell of pyridine is present after the evaporation). Residual solid was then suspended in methanol and filtrated through G3 fritted glass funnel. Next, porphyrin sediment was washed with methanol 3 times. The residue was dissolved in chloroform and concentrated. After drying, dibrominated porphyrin **5c** (121 mg, 96%) was obtained as a violet-red crystalline solid.

R_f = 0.65 (dichloromethane), mp: > 220 °C.

^{1}H NMR (600 MHz, $CDCl_3$) δ [ppm]: 9.61 (d, J = 4.8 Hz, 4H, H_β), 8.94 (br s, 4H, H_β), 7.34 (d, J = 2.3 Hz, 4H, H-2,2'$_{aryl}$), 6.92 (t, J = 2.3 Hz, 2H, H-4$_{aryl}$), 3.98 (s, 12H, -OC$\underline{H}_3$), -2.73 (br s, 2H, NH).

^{13}C NMR (151 MHz, $CDCl_3$) δ [ppm]: 159.1, 114.1, 103.9, 100.5, 55.8.

UV-Vis ($CHCl_3$) λ_{max} [nm] (log ε): 657.3 (3.61), 599.9 (3.67), 555.6 (3.93), 520.7 (4.20), 424.1 (5.45, Soret band).

MS (ESI+, isotopic profile) m/z (% rel. int.): $[M+H]^+$ calcd for $C_{36}H_{29}Br_2N_4O_4$ 739.1; found 746.1 (1), 745.1 (4), 744.1 (22), 743.1 (52), 742.1 (43), 741.1 (100), 740.1 (20), 739.1 (44).

HRMS (ESI+) m/z: $[M+H]^+$ calcd for $C_{36}H_{29}Br_2N_4O_4$ 739.0550; found 739.0549.

5,15-dibromo-10,20-bis(4-(tert-butyl)phenyl)porphyrin (**5d**)

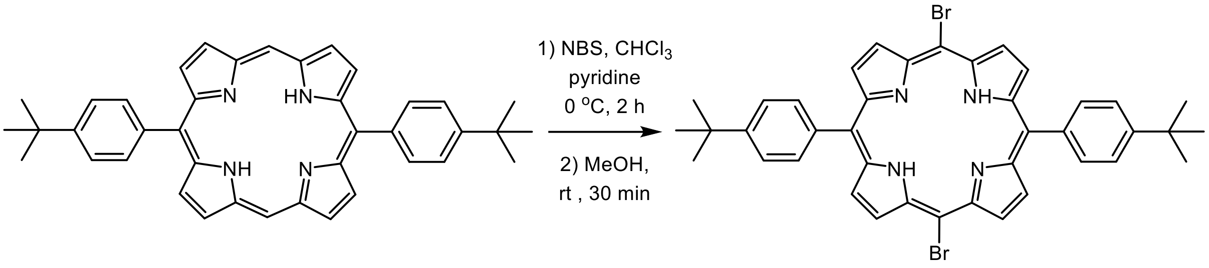

4d **5d**

To a round-bottom flask, porphyrin **4d** (116 mg, 0.201 mmol, 1.0 eq), chloroform (93 ml) and pyridine (0.72 ml) were added. Resulting solution was placed in an ice bath. Next, NBS (75 mg, 0.422 mmol, 2.1 eq) was added and mixture was stirred for 2 h in around 0 °C. After analysis with TLC (which detected still unreacted substrate), mixture was slowly heated to room temperature and methanol (10 ml) was added into the reaction. Resulting solution was stirred for 30 min. After that, reaction mixture was concentrated to dryness (making sure, that no smell of pyridine is present after the evaporation). Residual solid was then suspended in methanol and filtrated through G3 fritted glass funnel. Next, porphyrin sediment was washed with methanol 3 times. The residue was dissolved in chloroform and concentrated. After drying, dibrominated porphyrin **5d** (125 mg, 85%) was obtained as a violet-red crystalline solid. The characterisation data were in agreement with those of the literature.[15]

$\boldsymbol{R_f}$ = 0.69 (hexane/dichloromethane, 1:1, (v/v)).

^{1}H NMR (600 MHz, $CDCl_3$) δ [ppm]: 9.61 (br s, 4H), 8.89 (br s, 4H), 8.11 – 8.08 (m, 4H), 7.80 – 7.77 (m, 4H), 1.54 (s, 18H), -2.69 (br s, 2H).

5,15-dibromo-10,20-bis(5-ethylthiophen-2-yl)porphyrin (**5e**)

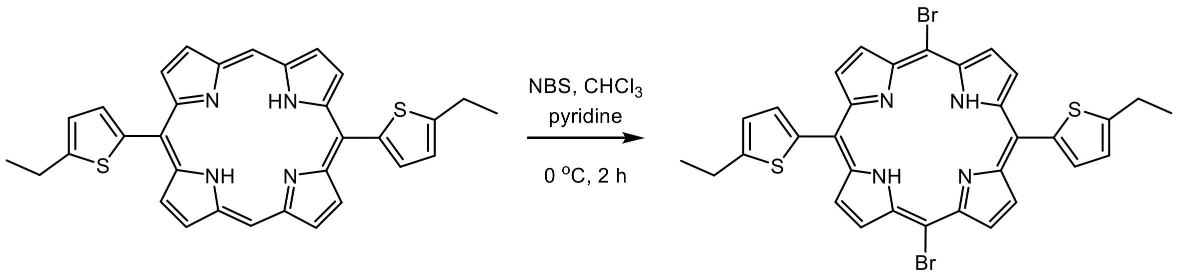

4e **5e**

To a round-bottom flask, porphyrin **4e** (50 mg, 0.094 mmol, 1.0 eq), chloroform (44 ml) and pyridine (0.34 ml) were added. Resulting solution was placed in an ice bath. Next, NBS (35 mg, 0.197 mmol, 2.1 eq) was added and mixture was stirred for 2 h in around 0 °C. After that, reaction mixture was concentrated to dryness (making sure, that no smell of pyridine is present after the evaporation). Residual solid was then suspended in methanol and filtrated through G3 fritted glass funnel. Next, porphyrin sediment was washed with methanol 3 times. The residue was dissolved in chloroform and concentrated. After drying, dibrominated porphyrin **5e** (60 mg, 93%) was obtained as a violet-red crystalline solid.

$\boldsymbol{R_f}$ = 0.68 (hexane/chloroform, 1:1, (v/v)), mp: > 220 °C.

^{1}H NMR (600 MHz, $CDCl_3$) δ [ppm]: 9.59 (d, J = 4.6 Hz, 4H, H_β), 9.12 (br s, 4H, H_β), 7.68 (d, J = 3.3 Hz, 2H, H-3_{aryl}), 7.20 (d, J = 3.2 Hz, 2H, H-4_{aryl}), 3.19 (q, J = 7.5 Hz, 4H, -C$\underline{H}_2$-), 1.60 (t, J = 7.5 Hz, 6H, -C$\underline{H}_3$), -2.64 (br s, 2H, NH).

^{13}C NMR (151 MHz, $CDCl_3$) δ [ppm]: 150.9, 139.2, 134.2, 122.9, 113.9, 104.5, 23.9, 16.2.

UV-Vis ($CHCl_3$) λ_{max} [nm] (log ε): 666.5 (3.65), 604.9 (3.69), 566.2 (4.05), 527.2 (4.13), 429.3 (5.45, Soret band).

MS (ESI+, isotopic profile) *m/z* (% rel. int.): $[M+H]^+$ calcd for $C_{32}H_{25}Br_2N_4S_2$ 687.0; found 694.0 (1), 693.0 (4), 692.0 (19), 691.0 (52), 690.0 (34), 689.0 (100), 688.0 (16), 687.0 (41).

HRMS (ESI+) *m/z*: $[M+H]^+$ calcd for $C_{32}H_{25}Br_2N_4S_2$ 686.9882; found 686.9884.

5,15-dibromo-10,20-bis(4-chlorophenyl)porphyrin (**5f**)

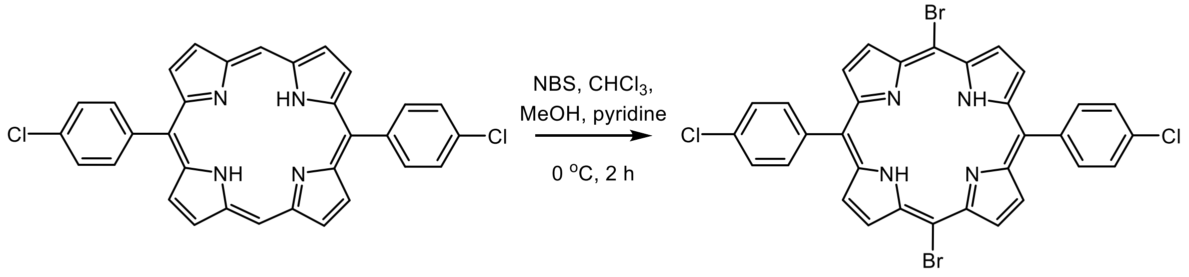

4f **5f**

To a round-bottom flask, porphyrin **4f** (108 mg, 0.203 mmol, 1.0 eq), chloroform (96 ml), methanol (10 ml) and pyridine (0.74 ml) were added. Resulting solution was placed in an ice bath. Next, NBS (76 mg, 0.428 mmol, 2.1 eq) was added and mixture was stirred for 2 h in around 0 °C. After that, reaction mixture was concentrated to dryness (making sure, that no smell of pyridine is present after the evaporation). Residual solid was then suspended in methanol and filtrated through G3 fritted glass funnel. Next, porphyrin sediment was washed with methanol 3 times. The residue was dissolved in chloroform and concentrated. After drying, dibrominated porphyrin **5f** (107 mg, 76%) was obtained as a violet-red crystalline solid.

R_f = 0.76 (hexane/dichloromethane, 1:1, (v/v)), mp: > 220 °C.

^{1}H NMR (500 MHz, pyridine-d$_6$, 60 °C) δ [ppm]: 9.78 (d, J = 4.8 Hz, 4H, H$_\beta$), 8.91 (d, J = 4.8 Hz, 4H, H$_\beta$), 8.19 – 8.16 (m, 4H, H-3,3'$_{aryl}$), 7.86 – 7.83 (m, 4H, H-2,2'$_{aryl}$), -2.57 (br s, 2H, NH).

Due to low solubility in common solvents, the ^{13}C NMR spectrum of **5f** cannot be obtained.

UV-Vis ($CHCl_3$) λ_{max} [nm] (log ε): 658.3 (3.66), 601.0 (3.67), 556.0 (4.01), 521.1 (4.20), 421.9 (5.50, Soret band).

MS (ESI+, isotopic profile) m/z (% rel. int.): [M+H]$^+$ calcd for $C_{32}H_{19}Br_2Cl_2N_4$ 686.9; found 695.9 (2), 694.9 (4), 693.9 (11), 692.9 (31), 691.9 (31), 690.9 (88), 689.9 (34), 688.9 (100), 687.9 (13), 686.9 (39).

HRMS (ESI+) m/z: [M+H]$^+$ calcd for $C_{32}H_{19}Br_2Cl_2N_4$ 686.9348; found 686.9342.

5,15-dibromo-10,20-bis(3-bromophenyl)porphyrin (**5g**)

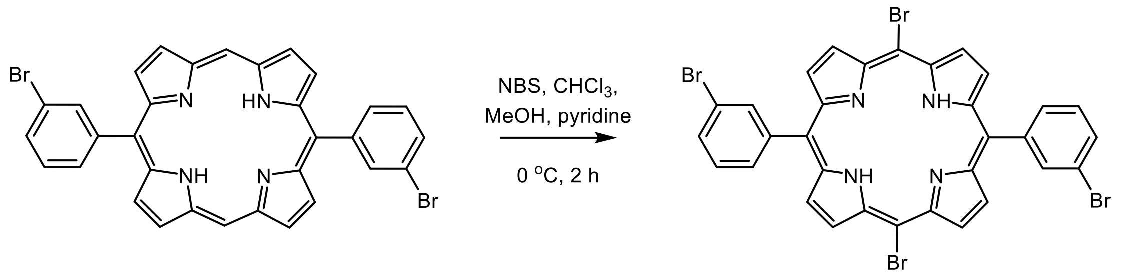

4g **5g**

To a round-bottom flask, porphyrin **4g** (110 mg, 0.177 mmol, 1.0 eq), chloroform (83 ml), methanol (10 ml) and pyridine (0.64 ml) were added. Resulting solution was placed in an ice bath. Next, NBS (66 mg, 0.372 mmol, 2.1 eq) was added and mixture was stirred for 2 h in around 0 °C. After that, reaction mixture was concentrated to dryness (making sure, that no smell of pyridine is present after the evaporation). Residual solid was then suspended in methanol and filtrated through G3 fritted glass funnel. Next, porphyrin sediment was washed with methanol 3 times. The residue was dissolved in chloroform and concentrated. After drying, dibrominated porphyrin **5g** (136 mg, 99%) was obtained as a violet-red crystalline solid.

***R**$_f$* = 0.70 (hexane/dichloromethane, 1:1, (v/v)), mp: > 220 °C.

^{1}H NMR (600 MHz, pyridine-d$_6$, 100 °C) δ [ppm]: 9.74 (d, *J* = 4.8 Hz, 4H, H$_\beta$), 8.89 (d, *J* = 4.9 Hz, 4H, H$_\beta$), 8.52 (t, *J* = 1.9 Hz, 2H, H-2$_{aryl}$), 8.19 (dt, *J* = 7.5, 1.3 Hz, 2H, H-4$_{aryl}$), 8.05 (ddd, *J* = 8.2, 2.0, 1.0 Hz, 2H, H-6$_{aryl}$), 7.67 (t, *J* = 7.7 Hz, 2H, H-5$_{aryl}$), -2.58 (br s, 2H, NH).

Due to low solubility in common solvents, the ^{13}C NMR spectrum of **5g** cannot be obtained.

UV-Vis ($CHCl_3$) λ_{max} [nm] (log ε): 658.1 (3.58), 600.0 (3.64), 555.0 (3.96), 520.6 (4.19), 421.4 (5.48, Soret band).

MS (ESI+, isotopic profile) *m/z* (% rel. int.): $[M+H]^+$ calcd for $C_{32}H_{19}Br_4N_4$ 774.8; found 783.8 (6), 782.8 (15), 781.8 (22), 780.8 (62), 779.8 (32), 778.8 (100), 777.8 (21), 776.8 (70), 775.8 (8), 774.8 (19).

HRMS (ESI+) *m/z*: $[M+H]^+$ calcd for $C_{32}H_{19}Br_4N_4$ 774.8338; found 774.8334.

5,15-dibromo-10,20-bis(3-cyanophenyl)porphyrin (**5h**)

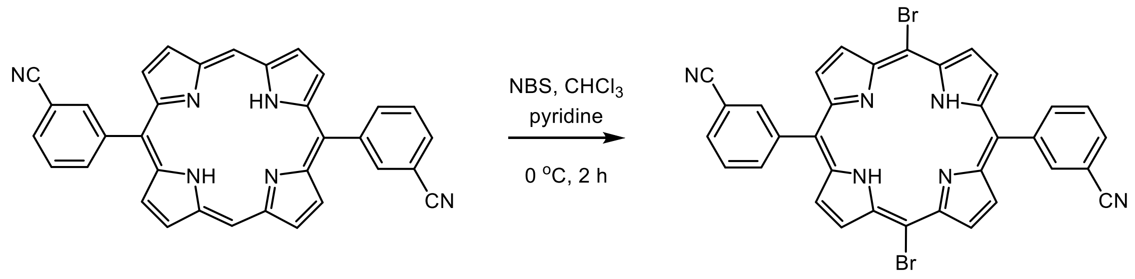

4h **5h**

To a round-bottom flask, porphyrin **4h** (60 mg, 0.117 mmol, 1.0 eq), chloroform (55 ml) and pyridine (0.42 ml) were added. Resulting solution was placed in an ice bath. Next, NBS (44 mg, 0.246 mmol, 2.1 eq) was added and mixture was stirred for 2 h in around 0 °C. After that, reaction mixture was concentrated to dryness (making sure, that no smell of pyridine is present after the evaporation). Residual solid was then suspended in methanol and filtrated through G3 fritted glass funnel. Next, porphyrin sediment was washed with methanol 3 times. The residue was dissolved in chloroform and concentrated. After drying, dibrominated porphyrin **5h** (76 mg, 97%) was obtained as a violet-red crystalline solid.

$\boldsymbol{R_f}$ = 0.64 (dichloromethane), mp: > 220 °C.

^{1}H NMR (600 MHz, $CDCl_3$) δ [ppm]: 9.66 (d, J = 4.8 Hz, 4H, H_β), 8.72 (br s, 4H, H_β), 8.47 (br s, 2H, H-2_{aryl}), 8.40 (d, J = 7.6 Hz, 2H, H-4_{aryl}), 8.14 (dt, J = 8.0, 1.4 Hz, 2H, H-6_{aryl}), 7.92 (t, J = 7.7 Hz, 2H, H-5_{aryl}), -2.79 (br s, 2H, NH).

^{13}C NMR (151 MHz, $CDCl_3$) δ [ppm]: 142.8, 138.3, 137.1, 132.1, 128.0, 118.8, 118.6, 111.8, 104.7.

UV-Vis ($CHCl_3$) λ_{max} [nm] (log ε): 658.1 (3.58), 600.0 (3.69), 554.0 (3.99), 520.5 (4.23), 421.7 (5.51, Soret band).

MS (ESI+, isotopic profile) m/z (% rel. int.): $[M+H]^+$ calcd for $C_{34}H_{19}Br_2N_6$ 669.0; found 674.0 (19), 673.0 (50), 672.0 (37), 671.0 (100), 670.0 (18), 669.0 (50).

HRMS (ESI+) m/z: $[M+H]^+$ calcd for $C_{34}H_{19}Br_2N_6$ 669.0033; found 669.0031.

3. Synthesis of new compounds

3.1 Synthesis and characterisation of new *trans*-A_2B_2 glycoporphyrins with different sugar motifs (**6a-d**)

5,15-bis(2-(3,4,6-tri-O-acetyl-D-glucal-2-yl)ethynyl)-10,20-diphenylporphyrin (**6a**)

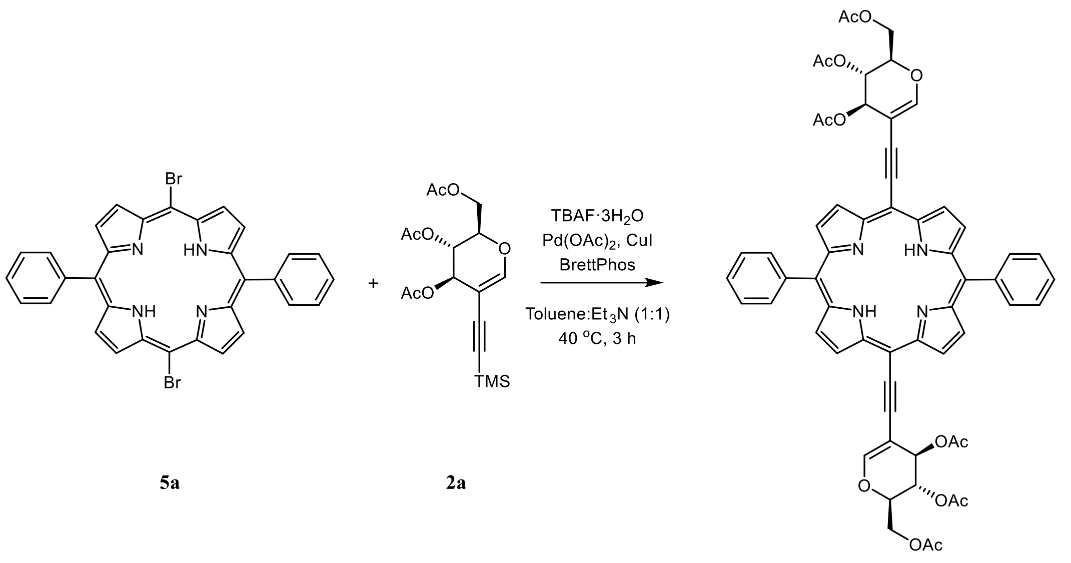

5a **2a** **6a**

To a sealed tube porphyrin **5a** (23.5 mg, 0.038 mmol, 1.0 eq), glucal **2a** (56.0 mg, 0.152 mmol, 4.0 eq), TBAF·$3H_2O$ (49.0 mg, 0.155 mmol, 4.1 eq), $Pd(OAc)_2$ (0.9 mg, 0.0038 mmol, 0.10 eq), CuI (0.7 mg, 0.0038 mmol, 0.10 eq), BrettPhos (4.1 mg, 0.0076 mmol, 0.20 eq) and toluene (1.25 ml) were added. Next, reaction vessel was flushed with argon and triethylamine (1.25 ml) was added. Reaction was stirred for 3 h at 40 °C. Upon completion, reaction vessel was washed with dichloromethane and diluted reaction mixture was concentrated to dryness with silica. Residual solid was then purified with flash chromatography (hexane/dichloromethane, 1:1. (v/v), to pure dichloromethane, to dichloromethane/ethyl acetate/triethylamine, 90:10:1, (v/v/v)) yielding glycoporphyrin **6a** (35.0 mg, 88%) as a violet-green crystalline solid. Analytical sample was obtained by crystallisation (THF/petroleum ether, solvents added at room temperature (0.45 mL THF and 1.0 mL petroleum ether), heated to boiling and left in freezer for 18 h).

R R_f = 0.61 (dichloromethane/ethyl acetate/triethylamine, 90:10:1, (v/v/v)), mp: > 220 °C.

^{1}H NMR (600 MHz, $CDCl_3$) δ [ppm]: 9.47 (d, J = 4.6 Hz, 4H, H_β), 8.80 (d, J = 4.5 Hz, 4H, H_β), 8.19 (d, J = 6.6 Hz, 4H, H-2,2'$_{aryl}$), 7.83 – 7.76 (m, 6H, H-3,3',4$_{aryl}$), 7.39 (br s, 2H, H-1$_{glycal}$), 6.00 (d, J = 5.3 Hz, 2H, H-3$_{glycal}$), 5.42 (t, J = 5.9 Hz, 2H, H-4$_{glycal}$), 4.53 (dd, J = 12.4, 5.4 Hz, 2H, H-6$_{glycal}$), 4.42 (br s, 2H, H-5$_{glycal}$), 4.30 (dd, J = 12.5, 2.7 Hz, 2H, H-6'$_{glycal}$), 2.24 (s, 6H, C$\underline{H}_3$COO-), 2.17 (s, 6H, C$\underline{H}_3$COO-), 2.17 (s, 6H, C$\underline{H}_3$COO-), -1.98 (br s, 2H, NH).

^{13}C NMR (151 MHz, $CDCl_3$) δ [ppm]: 170.7, 170.5, 169.7, 151.4, 141.5, 134.6, 128.1, 127.0, 122.0, 101.3, 98.1, 92.7, 91.7, 74.8, 67.5, 66.8, 61.3, 21.3, 21.0, 20.9.

UV-Vis (DMF) λ_{max} [nm] (log ε): 696.2 (4.30), 605.8 (4.61), 444.2 (5.43, Soret band).

MS (ESI+, isotopic profile) *m/z* (% rel. int.): $[M+H]^+$ calcd for $C_{60}H_{51}N_4O_{14}$ 1051.3; found 1054.3 (6), 1053.3 (25), 1052.3 (66), 1051.3 (100).

HRMS (ESI+) *m/z*: $[M+H]^+$ calcd for $C_{60}H_{51}N_4O_{14}$ 1051.3396; found 1051.3400.

Procedure for an increased scale:

To a sealed tube porphyrin **5a** (235.7 mg, 0.380 mmol, 1.0 eq), glucal **2a** (560.2 mg, 1.52 mmol, 4.0 eq), TBAF·3H_2O (491.6 mg, 1.56 mmol, 4.1 eq), Pd(OAc)$_2$ (8.5 mg, 0.038 mmol, 0.10 eq), CuI (7.2 mg, 0.038 mmol, 0.10 eq), BrettPhos (40.8 mg, 0.076 mmol, 0.20 eq) and toluene (25.0 ml) were added. Next, reaction vessel was flushed with argon and triethylamine (25.0 ml) was added. Reaction was stirred for 18 h at 40 °C. Upon completion, reaction vessel was washed with dichloromethane and diluted reaction mixture was concentrated to dryness with silica. Residual solid was then purified with flash chromatography (hexane/dichloromethane, 1:1. (v/v), to pure dichloromethane, to dichloromethane/ethyl acetate/triethylamine, 90:10:1, (v/v/v) to dichloromethane/ethyl acetate/triethylamine, 80:20:1, (v/v/v)) yielding glycoporphyrin **6a** (364.8 mg, 91%) as a violet-green crystalline solid.

5,15-bis(2-(3,4,6-tri-O-acetyl-D-galactal-2-yl)ethynyl)-10,20-diphenylporphyrin (**6b**)

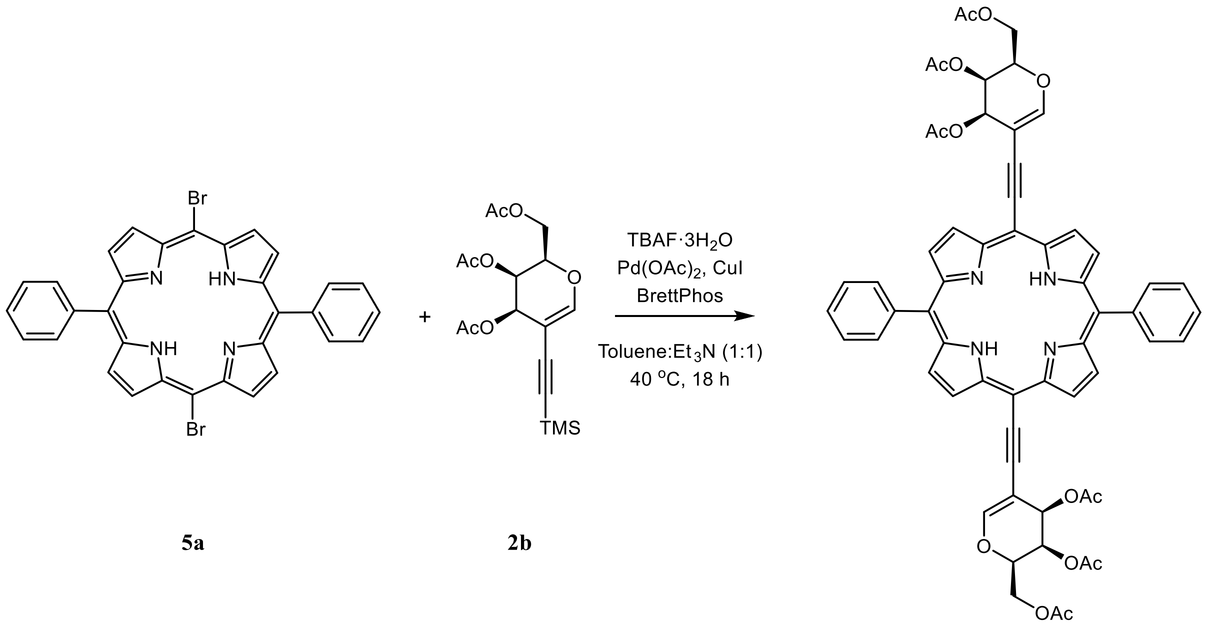

5a **2b** **6b**

To a sealed tube porphyrin **5a** (23.5 mg, 0.038 mmol, 1.0 eq), galactal **2b** (56.0 mg, 0.152 mmol, 4.0 eq), TBAF·3H_2O (49.0 mg, 0.155 mmol, 4.1 eq), $Pd(OAc)_2$ (0.9 mg, 0.0038 mmol, 0.10 eq), CuI (0.7 mg, 0.0038 mmol, 0.10 eq), BrettPhos (4.1 mg, 0.0076 mmol, 0.20 eq) and toluene (2.5 ml) were added. Next, reaction vessel was flushed with argon and triethylamine (2.5 ml) was added. Reaction was stirred for 18 h at 40°C. Upon completion, reaction vessel was washed with dichloromethane and diluted reaction mixture was concentrated to dryness with silica. Residual solid was then purified with flash chromatography (hexane/dichloromethane, 1:1. (v/v), to pure dichloromethane, to dichloromethane/ethyl acetate/triethylamine, 90:10:1, (v/v/v)) yielding glycoporphyrin **6b** (32.8 mg, 82%) as a violet-green crystalline solid. Analytical sample was obtained by crystallisation (THF/petroleum ether, solvents added at room temperature, heated to boiling and left in freezer for 18 h).

R_f = 0.58 (dichloromethane/ethyl acetate/triethylamine, 90:10:1, (v/v/v)), mp: > 220 °C.

^{1}H NMR (600 MHz, $CDCl_3$) δ [ppm]: 9.46 (d, *J* = 4.6 Hz, 4H, H_β), 8.77 (d, *J* = 4.5 Hz, 4H, H_β), 8.16 (d, *J* = 7.0 Hz, 4H, H-2,2'$_{aryl}$), 7.82 – 7.75 (m, 6H, H-3,3',4$_{aryl}$), 7.44 (br s, 2H, H-1$_{glycal}$), 6.18 (d, *J* = 4.2 Hz, 2H, H-3$_{glycal}$), 5.71 (d, *J* = 4.4 Hz, 2H, H-4$_{glycal}$), 4.60 (t, *J* = 5.5 Hz, 2H, H-5$_{glycal}$), 4.46 – 4.34 (m, 4H, H-6,6'$_{glycal}$), 2.28 (s, 6H, C$\underline{H}_3$COO-), 2.25 (s, 6H, C$\underline{H}_3$COO-), 2.16 (s, 6H, C$\underline{H}_3$COO-), -1.96 (br s, 2H, NH).

^{13}C NMR (151 MHz, $CDCl_3$) δ [ppm]: 170.7, 170.4, 151.3, 141.4, 134.5, 128.1, 127.1, 122.0, 101.4, 98.1, 93.2, 91.8, 74.1, 65.1, 63.4, 61.9, 21.18, 20.93, 20.90.

UV-Vis (DMF) λ_{max} [nm] (log ε): 698.7 (4.01), 608.9 (4.32), 445.8 (5.15, Soret band).

MS (ESI+, isotopic profile) *m/z* (% rel. int.): $[M+H]^+$ calcd for $C_{60}H_{51}N_4O_{14}$ 1051.3; found 1054.3 (6), 1053.3 (25), 1052.3 (66), 1051.3 (100).

HRMS (ESI+) *m/z*: $[M+H]^+$ calcd for $C_{60}H_{51}N_4O_{14}$ 1051.3396; found 1051.3390.

5,15-bis(2-(3,4-di-O-acetyl-D-xylal-2-yl)ethynyl)-10,20-diphenylporphyrin (**6c**)

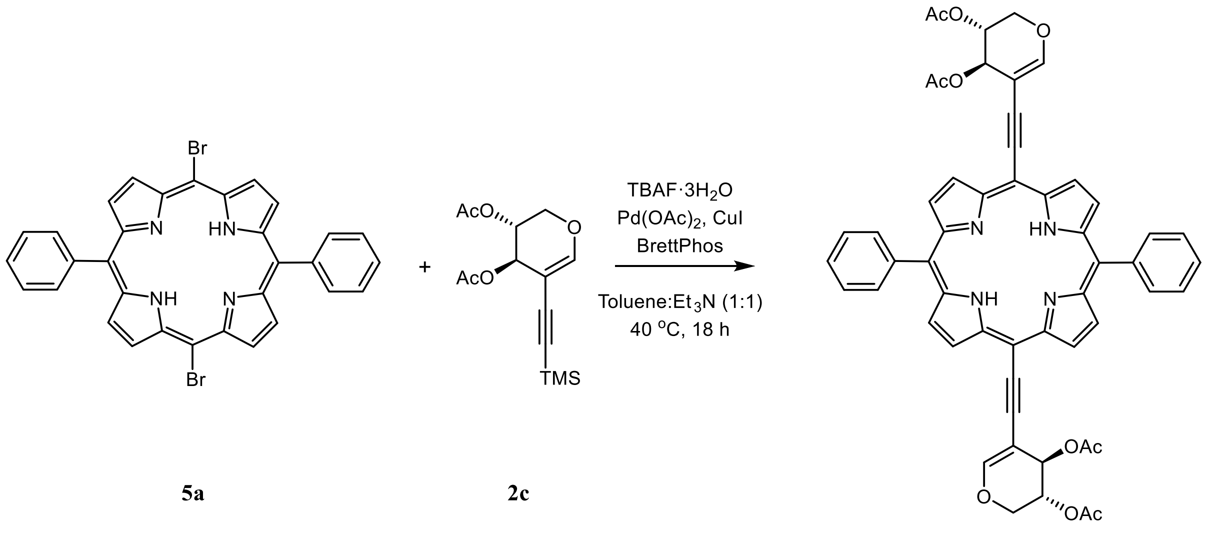

5a **2c** **6c**

To a sealed tube porphyrin **5a** (23.5 mg, 0.038 mmol, 1.0 eq), xylal **2c** (45.0 mg, 0.152 mmol, 4.0 eq), TBAF·3H_2O (49.0 mg, 0.155 mmol, 4.1 eq), Pd(OAc)$_2$ (0.9 mg, 0.0038 mmol, 0.10 eq), CuI (0.7 mg, 0.0038 mmol, 0.10 eq), BrettPhos (4.1 mg, 0.0076 mmol, 0.20 eq) and toluene (2.5 ml) were added. Next, reaction vessel was flushed with argon and triethylamine (2.5 ml) was added. Reaction was stirred for 18 h at 40°C. Upon completion, reaction vessel was washed with dichloromethane and diluted reaction mixture was concentrated to dryness with silica. Residual solid was then purified with flash chromatography (hexane/dichloromethane, 1:1. (v/v), to pure dichloromethane, to dichloromethane/ethyl acetate/triethylamine, 95:5:1, (v/v/v)) yielding glycoporphyrin **6c** (15.7 mg, 46%) as a violet-green crystalline solid. Analytical sample was obtained by crystallisation (THF/petroleum ether, solvents added at room temperature, heated to boiling and left in freezer for 18 h).

$\boldsymbol{R_f}$ = 0.74 (hexane/ethyl acetate, 2:8, (v/v)), mp: 198-200 °C.

^{1}H NMR (600 MHz, $CDCl_3$) δ [ppm]: 9.52 (d, J = 4.6 Hz, 4H, H_β), 8.78 (d, J = 4.6 Hz, 4H, H_β), 8.17 (d, J = 6.9 Hz, 4H, H-2,2'$_{aryl}$), 7.82 – 7.75 (m, 6H, H-3,3',4$_{aryl}$), 7.61 (br s, 2H, H-1$_{glycal}$), 5.90 (br s, 2H, H-3$_{glycal}$), 5.18 (br s, 2H, H-4$_{glycal}$), 4.50 (br d, J = 12.1, 2H, H-5$_{glycal}$), 4.24 (br d, J = 12.1 Hz, 2H, H-5'$_{glycal}$), 2.28 (s, 6H, C$\underline{H}_3$COO-), 2.21 (s, 6H, C$\underline{H}_3$COO-), -1.95 (br s, 2H, NH).

^{13}C NMR (151 MHz, $CDCl_3$) δ [ppm]: 170.0, 169.9, 153.8, 141.6, 134.6, 128.1, 127.0, 121.8, 101.6, 96.7, 93.5, 92.1, 66.8, 65.0, 64.3, 29.9, 21.4, 21.2.

UV-Vis (DMF) λ_{max} [nm] (log ε): 696.9 (3.91), 607.2 (4.25), 444.8 (5.15, Soret band).

MS (ESI+, isotopic profile) m/z (% rel. int.): $[M+H]^+$ calcd for $C_{54}H_{43}N_4O_{10}$ 907.3; found 910.3 (4), 909.3 (20), 908.3 (62), 907.3 (100).

HRMS (ESI+) m/z: $[M+H]^+$ calcd for $C_{54}H_{43}N_4O_{10}$ 907.2974; found 907.2980.

5,15-bis(2-(3,4-di-O-acetyl-L-rhamnal-2-yl)ethynyl)-10,20-diphenylporphyrin (**6d**)

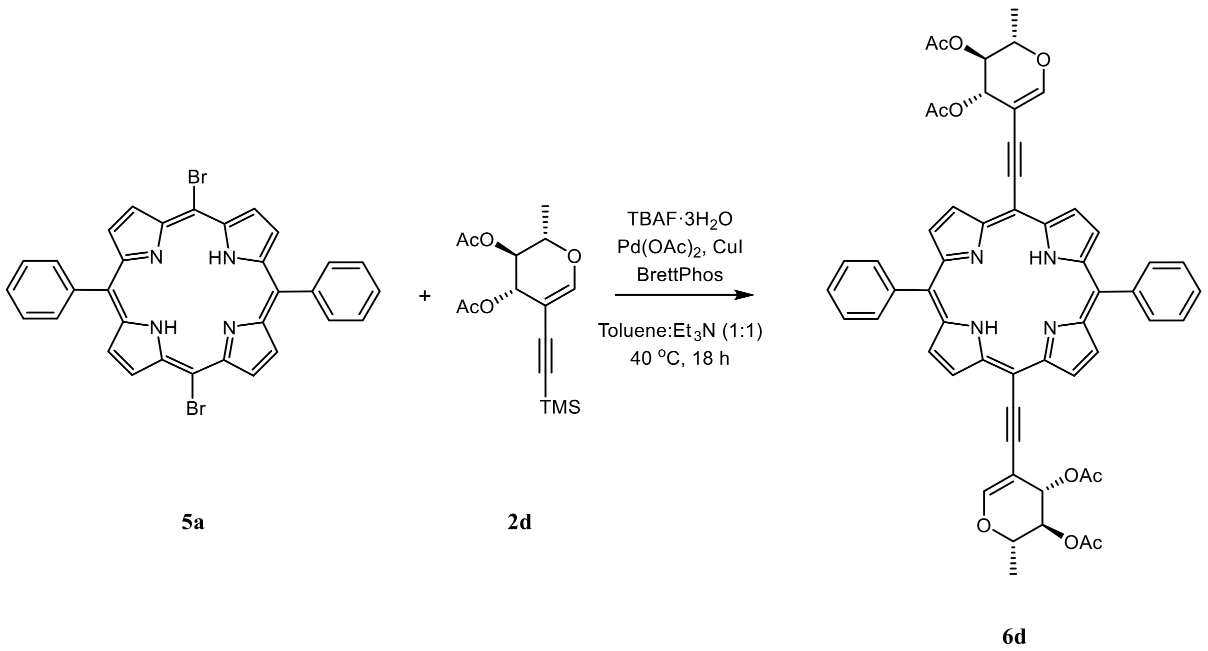

To a sealed tube porphyrin **5a** (23.5 mg, 0.038 mmol, 1.0 eq), rhamnal **2d** (47.2 mg, 0.152 mmol, 4.0 eq), TBAF·3H_2O (49.0 mg, 0.155 mmol, 4.1 eq), $Pd(OAc)_2$ (0.9 mg, 0.0038 mmol, 0.10 eq), CuI (0.7 mg, 0.0038 mmol, 0.10 eq), BrettPhos (4.1 mg, 0.0076 mmol, 0.20 eq) and toluene (2.5 ml) were added. Next, reaction vessel was flushed with argon and triethylamine (2.5 ml) was added. Reaction was stirred for 18 h at 40°C. Upon completion, reaction vessel was washed with dichloromethane and diluted reaction mixture was concentrated to dryness with silica. Residual solid was then purified with flash chromatography (hexane/dichloromethane, 1:1. (v/v), to pure dichloromethane, to dichloromethane/ethyl acetate/triethylamine, 95:5:1, (v/v/v)) yielding glycoporphyrin **6d** (25.0 mg, 70%) as a violet-green crystalline solid. Analytical sample was obtained by crystallisation (THF/petroleum ether, solvents added at room temperature, heated to boiling and left in freezer for 18 h).

$\boldsymbol{R_f}$ = 0.78 (hexane/ethyl acetate, 2:8, (v/v)), mp: > 220 °C.

^{1}H NMR (600 MHz, $CDCl_3$) δ [ppm]: 9.47 (d, J = 4.6 Hz, 4H, H_β), 8.77 (d, J = 4.6 Hz, 4H, H_β), 8.17 (d, J = 6.9 Hz, 4H, H-2,2'$_{aryl}$), 7.82 – 7.75 (m, 6H, H-3,3',4$_{aryl}$), 7.44 (br s, 2H, H-1$_{glycal}$), 6.07 (d, J = 5.4 Hz, 2H, H-3$_{glycal}$), 5.26 (t, J = 6.2 Hz, 2H, H-4$_{glycal}$), 4.46 (p, J = 6.3 Hz, 2H, H-5$_{glycal}$), 2.24 (s, 6H, C$\underline{H}_3$COO-), 2.19 (s, 6H, C$\underline{H}_3$COO-), 1.52 (d, J = 6.3 Hz, 6H, C$\underline{H}_3$-), -1.96 (br s, 2H, NH).

^{13}C NMR (151 MHz, $CDCl_3$) δ [ppm]: 170.7, 170.0, 152.0, 141.5, 134.6, 128.1, 127.0, 121.9, 101.5, 97.7, 92.4, 92.4, 73.6, 71.4, 68.0, 21.3, 21.1, 16.7.

UV-Vis (DMF) λ_{max} [nm] (log ε): 697.8 (4.11), 607.8 (4.44), 444.9 (5.32, Soret band).

MS (ESI+, isotopic profile) *m/z* (% rel. int.): $[M+H]^+$ calcd for $C_{56}H_{47}N_4O_{10}$ 935.3; found 940.3 (2), 939.3 (4), 938.3 (6), 937.3 (22), 936.3 (65), 935.3 (100).

HRMS (ESI+) *m/z*: $[M+H]^+$ calcd for $C_{56}H_{47}N_4O_{10}$ 935.3287; found 935.3283.

3.2 Synthesis and characterisation of new glycoporphyrins with different aryl fragments (**7b-h**)

5,15-bis(2-(3,4,6-tri-O-acetyl-D-glucal-2-yl)ethynyl)-10,20-bis(4-(tert-butoxycarbonyl)phenyl)porphyrin (**7b**)

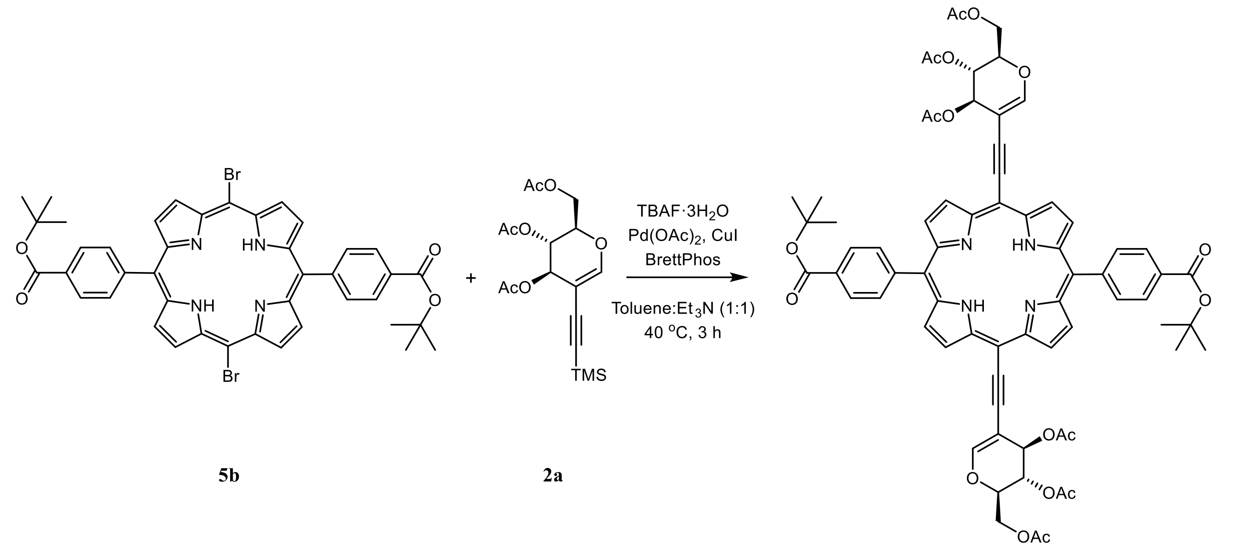

5b **2a** **7b**

To a sealed tube porphyrin **5b** (31.2 mg, 0.038 mmol, 1.0 eq), glucal **2a** (56.0 mg, 0.152 mmol, 4.0 eq), TBAF·3H_2O (49.0 mg, 0.155 mmol, 4.1 eq), $Pd(OAc)_2$ (0.9 mg, 0.0038 mmol, 0.10 eq), CuI (0.7 mg, 0.0038 mmol, 0.10 eq), BrettPhos (4.1 mg, 0.0076 mmol, 0.20 eq) and toluene (2.5 ml) were added. Next, reaction vessel was flushed with argon and triethylamine (2.5 ml) was added. Reaction was stirred for 3 h at 40°C. Upon completion, reaction vessel was washed with dichloromethane and diluted reaction mixture was concentrated to dryness with silica. Residual solid was then purified with flash chromatography (hexane/dichloromethane, 1:1. (v/v), to pure dichloromethane, to dichloromethane/ethyl acetate/triethylamine, 90:10:1, (v/v/v)) yielding glycoporphyrin **7b** (43.0 mg, 90%) as a violet-green crystalline solid. Analytical sample was obtained by crystallisation (THF/petroleum ether, solvents added at room temperature, heated to boiling and left in freezer for 18 h).

$\boldsymbol{R_f}$= 0.51 (dichloromethane/ethyl acetate/triethylamine, 90:10:1, (v/v/v)), mp: > 220 °C.

^{1}H NMR (600 MHz, $CDCl_3$) δ [ppm]: 9.48 (d, J = 4.7 Hz, 4H, H_β), 8.73 (d, J = 4.7 Hz, 4H, H_β), 8.42 – 8.39 (m, 4H, H-3,3'$_{aryl}$), 8.24 – 8.21 (m, 4H, H-2,2'$_{aryl}$), 7.47 (d, J = 1.1 Hz, 2H, H-1$_{glycal}$), 6.08 (d, J = 5.3 Hz, 2H, H-3$_{glycal}$), 5.46 (dd, J = 6.6, 5.2 Hz, 2H, H-4$_{glycal}$), 4.63 – 4.55 (m, 4H, H-5,6$_{glycal}$), 4.41 – 4.36 (m, 2H, H-6'$_{glycal}$), 2.24 (s, 6H, $C\underline{H}_3$COO-), 2.18 (s, 6H, $C\underline{H}_3$COO-), 2.17 (s, 6H, $C\underline{H}_3$COO-), 1.78 (s, 18H, $(C\underline{H}_3)_3$C-), -2.01 (br s, 2H, NH).

^{13}C NMR (151 MHz, $CDCl_3$) δ [ppm]: 170.7, 170.5, 169.7, 166.0, 151.6, 145.7, 134.4, 131.9, 128.1, 120.9, 101.7, 98.1, 92.5, 92.1, 81.7, 74.9, 67.4, 66.9, 61.4, 28.5, 21.3, 21.0, 20.9.

UV-Vis (DMF) λ_{max} [nm] (log ε): 697.4 (4.24), 607.2 (4.61), 446.8 (5.44, Soret band).

MS (ESI+, isotopic profile) *m/z* (% rel. int.): $[M+H]^+$ calcd for $C_{70}H_{67}N_4O_{18}$ 1251.4; found 1255.4 (2), 1254.4 (11), 1253.4 (36), 1252.4 (79), 1251.4 (100).

HRMS (ESI+) *m/z*: $[M+H]^+$ calcd for $C_{70}H_{67}N_4O_{18}$ 1251.4445; found 1251.4451.

5,15-bis(2-(3,4,6-tri-O-acetyl-D-glucal-2-yl)ethynyl)-10,20-bis(3,5-dimethoxyphenyl)porphyrin (**7c**)

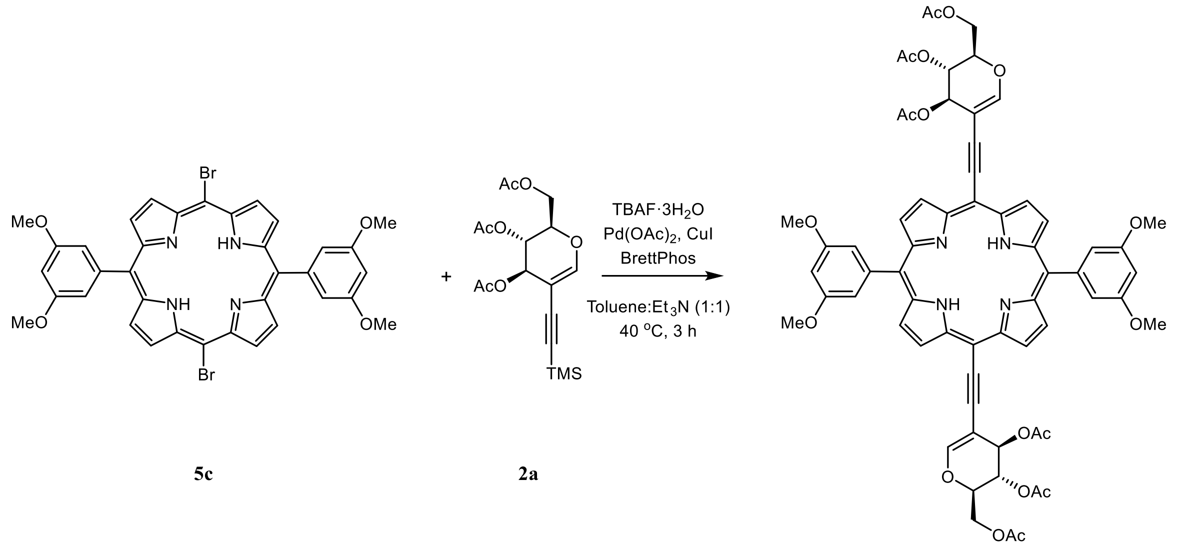

5c **2a** **7c**

To a sealed tube porphyrin **5c** (28.1 mg, 0.038 mmol, 1.0 eq), glucal **2a** (56.0 mg, 0.152 mmol, 4.0 eq), TBAF·3H_2O (49.0 mg, 0.155 mmol, 4.1 eq), Pd(OAc)$_2$ (0.9 mg, 0.0038 mmol, 0.10 eq), CuI (0.7 mg, 0.0038 mmol, 0.10 eq), BrettPhos (4.1 mg, 0.0076 mmol, 0.20 eq) and toluene (2.5 ml) were added. Next, reaction vessel was flushed with argon and triethylamine (2.5 ml) was added. Reaction was stirred for 3 h at 40°C. Upon completion, reaction vessel was washed with dichloromethane and diluted reaction mixture was concentrated to dryness with silica. Residual solid was then purified with flash chromatography (hexane/dichloromethane, 1:1. (v/v), to pure dichloromethane, to dichloromethane/ethyl acetate/triethylamine, 90:10:1, (v/v/v)) yielding glycoporphyrin **7c** (31.7 mg, 71%) as a violet-green crystalline solid. Analytical sample was obtained by crystallisation (chloroform/cyclohexane, solvents added at room temperature, heated to boiling and left in refrigerator for 30 min).

R_f = 0.31 (dichloromethane/ethyl acetate/triethylamine, 90:10:1, (v/v/v)), mp: > 220 °C.

^{1}H NMR (600 MHz, CDCl$_3$) *δ* [ppm]: 9.46 (d, *J* = 4.7 Hz, 4H, $H_β$), 8.88 (d, *J* = 4.7 Hz, 4H, $H_β$), 7.46 (d, *J* = 1.0 Hz, 2H, H-1$_{glycal}$), 7.35 (d, *J* = 2.2 Hz, 4H, H-2,2'$_{aryl}$), 6.91 (t, *J* = 2.3 Hz, 2H, H-4$_{aryl}$), 6.08 (d, *J* = 5.3 Hz, 2H, H-3$_{glycal}$), 5.46 (dd, *J* = 6.5, 5.2 Hz, 2H, H-4$_{glycal}$), 4.62 – 4.55 (m, 4H, H-5,6$_{glycal}$), 4.38 (dd, *J* = 11.6, 2.4 Hz, 2H, H-6'$_{glycal}$), 3.98 (s, 12H, C$\underline{H}_3$O-), 2.25 (s, 6H, C$\underline{H}_3$COO-), 2.19 (s, 6H, C$\underline{H}_3$COO-), 2.18 (s, 6H, C$\underline{H}_3$COO-), -2.03 (br s, 2H, NH).

^{13}C NMR (151 MHz, CDCl$_3$) *δ* [ppm]: 170.7, 170.5, 169.7, 159.2, 151.4, 143.3, 121.6, 113.9, 101.2, 100.4, 98.2, 92.7, 91.7, 74.9, 67.5, 66.9, 61.4, 55.8, 21.3, 21.0, 20.9.

UV-Vis (DMF) λ_{max} [nm] (log *ε*): 695.6 (4.20), 605.4 (4.53), 447.1 (5.37, Soret band).

MS (ESI+, isotopic profile) *m/z* (% rel. int.): $[M+H]^+$ calcd for $C_{64}H_{59}N_4O_{18}$ 1171.4; found 1174.4 (8), 1173.4 (28), 1172.4 (72), 1171.4 (100).

HRMS (ESI+) *m/z*: $[M+H]^+$ calcd for $C_{64}H_{59}N_4O_{18}$ 1171.3818; found 1171.3818.

5,15-bis(2-(3,4,6-tri-O-acetyl-D-glucal-2-yl)ethynyl)-10,20-bis(4-(tert-butyl)phenyl)porphyrin (**7d**)

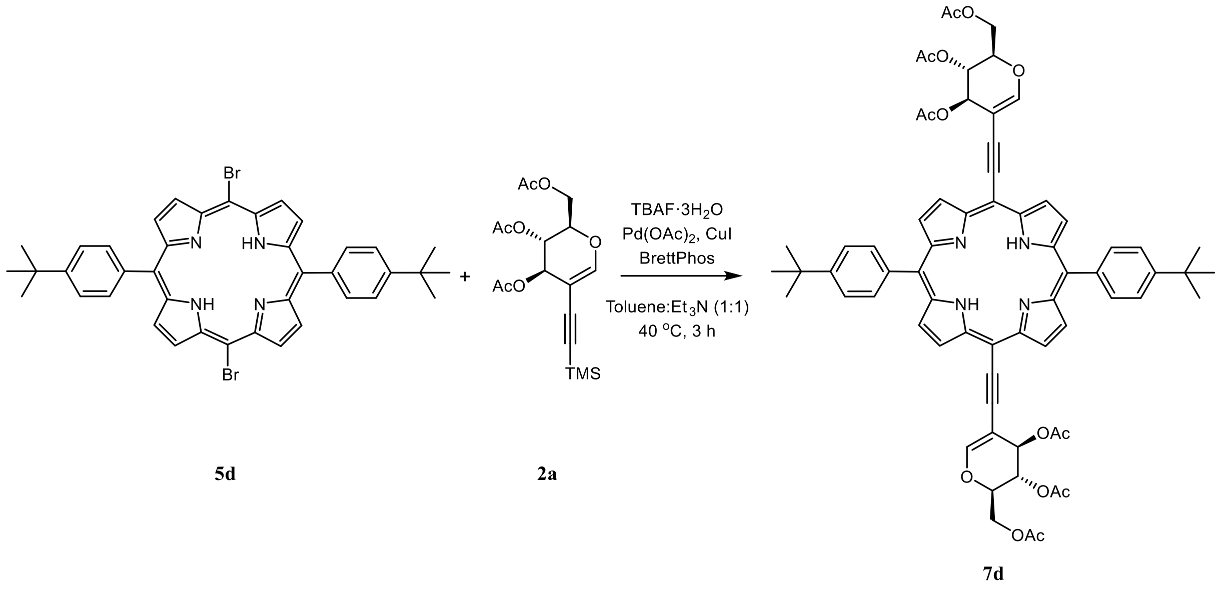

To a sealed tube porphyrin **5d** (27.8 mg, 0.038 mmol, 1.0 eq), glucal **2a** (56.0 mg, 0.152 mmol, 4.0 eq), TBAF·3H_2O (49.0 mg, 0.155 mmol, 4.1 eq), $Pd(OAc)_2$ (0.9 mg, 0.0038 mmol, 0.10 eq), CuI (0.7 mg, 0.0038 mmol, 0.10 eq), BrettPhos (4.1 mg, 0.0076 mmol, 0.20 eq) and toluene (2.5 ml) were added. Next, reaction vessel was flushed with argon and triethylamine (2.5 ml) was added. Reaction was stirred for 3 h at 40°C. Upon completion, reaction vessel was washed with dichloromethane and diluted reaction mixture was concentrated to dryness with silica. Residual solid was then purified with flash chromatography (hexane/dichloromethane, 1:1. (v/v), to pure dichloromethane, to dichloromethane/ethyl acetate/triethylamine, 90:10:1, (v/v/v)) yielding glycoporphyrin **7d** (20.1 mg, 45%) as a violet-green crystalline solid. Analytical sample was obtained by crystallisation (THF/petroleum ether, solvents added at room temperature, heated to boiling and left in freezer for 18 h).

***R**$_f$*= 0.67 (dichloromethane/ethyl acetate/triethylamine, 90:10:1, (v/v/v)), mp: > 220 °C.

^{1}H NMR (600 MHz, $CDCl_3$) *δ* [ppm]: 9.46 (d, *J* = 4.6 Hz, 4H, $H_β$), 8.83 (d, *J* = 4.6 Hz, 4H, $H_β$), 8.11 – 8.08 (m, 4H, H-2,2'$_{aryl}$), 7.80 – 7.77 (m, 4H, H-3,3'$_{aryl}$), 7.45 (br s, 2H, H-1$_{glycal}$), 6.09 (br s, 2H, H-3$_{glycal}$), 5.46 (t, *J* = 5.9 Hz, 2H, H-4$_{glycal}$), 4.61 – 4.53 (m, 4H, H-5,6$_{glycal}$), 4.37 (br d, *J* = 11.9 Hz, 2H, H-6'$_{glycal}$), 2.24 (s, 6H, C$\underline{H}_3$COO-), 2.18 (s, 6H, C$\underline{H}_3$COO-), 2.17 (s, 6H, C$\underline{H}_3$COO-), 1.62 (s, 18H, (C$\underline{H}_3$)$_3$C-), -1.98 (br s, 2H, NH).

^{13}C NMR (151 MHz, $CDCl_3$) *δ* [ppm]: 170.7, 170.6, 169.7, 151.0, 138.4, 134.5, 124.0, 122.2, 101.1, 98.3, 92.9, 91.6, 74.9, 67.5, 66.9, 61.4, 35.1, 31.9, 21.3, 21.0, 20.9.

UV-Vis (DMF) $λ_{max}$ [nm] (log *ε*): 698.5 (4.34), 607.9 (4.64), 445.1 (5.45, Soret band).

MS (ESI+, isotopic profile) *m/z* (% rel. int.): $[M+H]^+$ calcd for $C_{68}H_{67}N_4O_{14}$ 1163.5; found 1167.5 (2), 1166.5 (9), 1165.5 (32), 1164.5 (75), 1163.5 (100).

HRMS (ESI+) *m/z*: $[M+H]^+$ calcd for $C_{68}H_{67}N_4O_{14}$ 1163.4648; found 1163.4649.

5,15-bis(2-(3,4,6-tri-O-acetyl-D-glucal-2-yl)ethynyl)-10,20-bis(5-ethylthiophen-2-yl)porphyrin (**7e**)

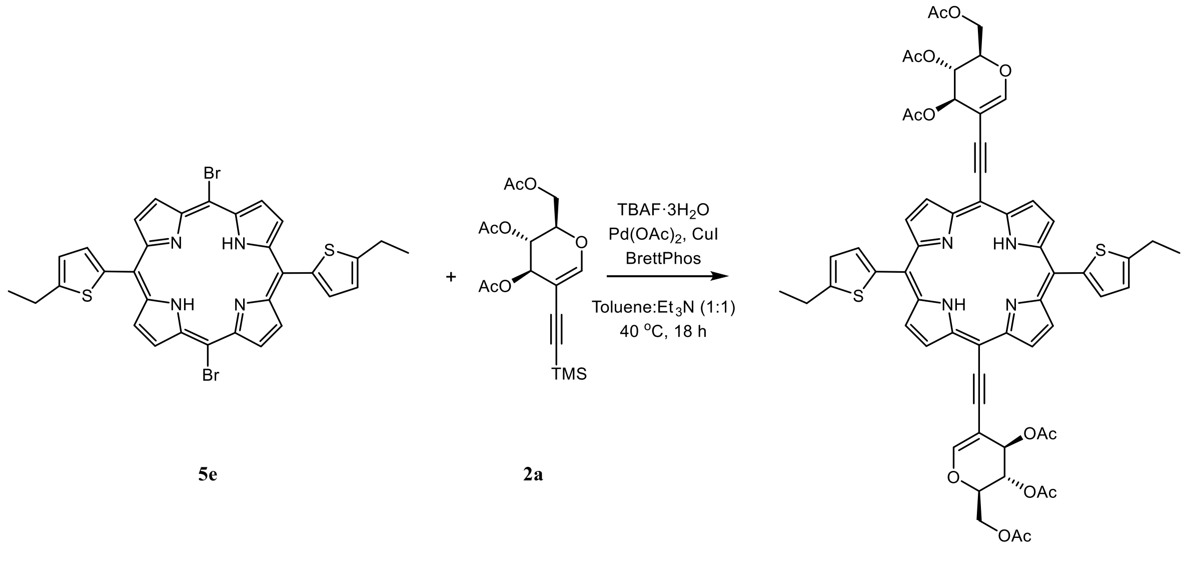

To a sealed tube porphyrin **5e** (26.2 mg, 0.038 mmol, 1.0 eq), glucal **2a** (56.0 mg, 0.152 mmol, 4.0 eq), TBAF·3H_2O (49.0 mg, 0.155 mmol, 4.1 eq), $Pd(OAc)_2$ (0.9 mg, 0.0038 mmol, 0.10 eq), CuI (0.7 mg, 0.0038 mmol, 0.10 eq), BrettPhos (4.1 mg, 0.0076 mmol, 0.20 eq) and toluene (2.5 ml) were added. Next, reaction vessel was flushed with argon and triethylamine (2.5 ml) was added. Reaction was stirred for 18 h at 40°C. Upon completion, reaction vessel was washed with dichloromethane and diluted reaction mixture was concentrated to dryness with silica. Residual solid was then purified with flash chromatography (hexane/dichloromethane, 1:1. (v/v), to pure dichloromethane, to dichloromethane/ethyl acetate/triethylamine, 90:10:1, (v/v/v)) yielding glycoporphyrin **7e** (21.6 mg, 51%) as a violet-green crystalline solid. Analytical sample was obtained by crystallisation (THF/petroleum ether, solvents added at room temperature, heated to boiling and left in freezer for 18 h).

$\boldsymbol{R_f}$ = 0.55 (dichloromethane/ethyl acetate/triethylamine, 90:10:1, (v/v/v)), mp: > 220 °C.

^{1}H NMR (600 MHz, $CDCl_3$) δ [ppm]: 9.45 (d, J = 4.6 Hz, 4H, H_β), 9.07 (d, J = 4.6 Hz, 4H, H_β), 7.67 (d, J = 3.3 Hz, 2H, H-2_{aryl}), 7.47 (br s, 2H, H-1_{glycal}), 7.20 (d, J = 3.2 Hz, 2H, H-3_{aryl}), 6.09 (d, J = 5.3 Hz, 2H, H-3_{glycal}), 5.47 (t, J = 5.7 Hz, 2H, H-4_{glycal}), 4.63 – 4.57 (m, 4H, H-$5,6_{glycal}$), 4.41 – 4.37 (m, 2H, H-$6'_{glycal}$), 3.19 (q, J = 7.6 Hz, 4H, -C$\underline{H}_2$-), 2.26 (s, 6H, C$\underline{H}_3$COO-), 2.19 (s, 6H, C$\underline{H}_3$COO-), 2.18 (s, 6H, C$\underline{H}_3$COO-), 1.60 (t, J = 7.5 Hz, 6H, -C$\underline{H}_3$), -1.93 (br s, 2H, NH).

^{13}C NMR (151 MHz, $CDCl_3$) δ [ppm]: 170.7, 170.6, 169.7, 151.5, 150.8, 139.2, 133.8, 123.0, 114.3, 101.7, 98.2, 92.7, 91.8, 74.9, 67.5, 66.9, 61.4, 23.9, 21.3, 21.0, 20.9, 16.2.

UV-Vis (DMF) λ_{max} [nm] (log ε): 703.1 (3.79), 613.0 (4.27), 452.0 (5.24, Soret band).

MS (ESI+, isotopic profile) *m/z* (% rel. int.): $[M+H]^+$ calcd for $C_{60}H_{55}N_4O_{14}S_2$ 1119.3; found 1124.3 (4), 1123.3 (7), 1122.3 (9), 1121.3 (27), 1120.3 (70), 1119.3 (100).

HRMS (ESI+) *m/z*: $[M+H]^+$ calcd for $C_{60}H_{55}N_4O_{14}S_2$ 1119.3151; found 1119.3143.

5,15-bis(2-(3,4,6-tri-O-acetyl-D-glucal-2-yl)ethynyl)-10,20-bis(4-chlorophenyl)porphyrin (**7f**)

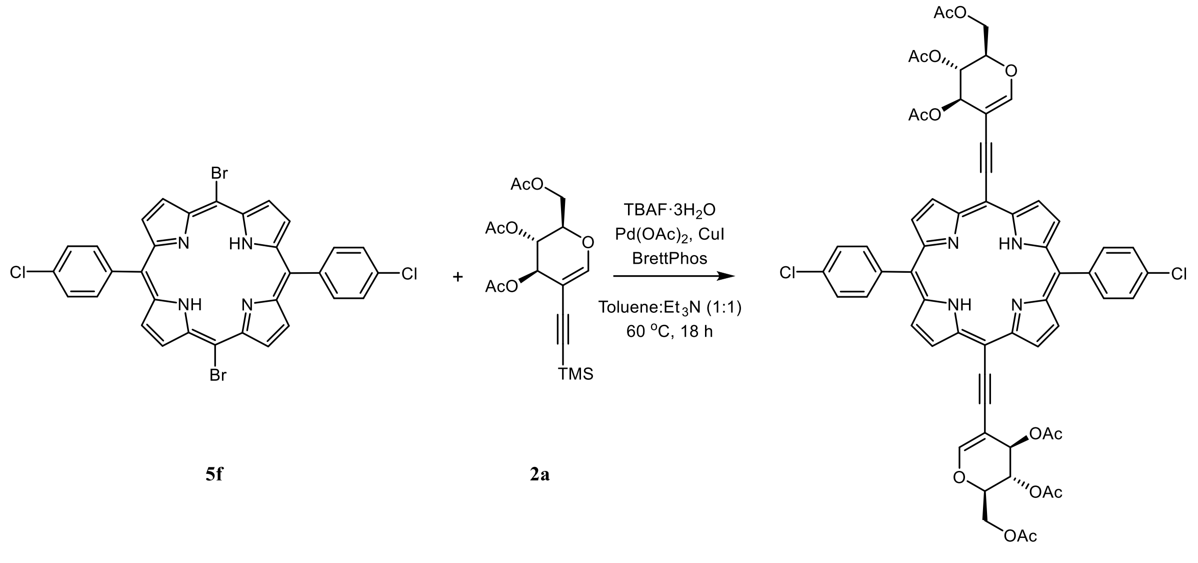

5f **2a** **7f**

To a sealed tube porphyrin **5f** (26.2 mg, 0.038 mmol, 1.0 eq), glucal **2a** (56.0 mg, 0.152 mmol, 4.0 eq), TBAF·3H_2O (49.0 mg, 0.155 mmol, 4.1 eq), $Pd(OAc)_2$ (0.9 mg, 0.0038 mmol, 0.10 eq), CuI (0.7 mg, 0.0038 mmol, 0.10 eq), BrettPhos (4.1 mg, 0.0076 mmol, 0.20 eq) and toluene (2.5 ml) were added. Next, reaction vessel was flushed with argon and triethylamine (2.5 ml) was added. Reaction was stirred for 18 h at 60°C. Upon completion, reaction vessel was washed with chloroform and diluted reaction mixture was concentrated to dryness with silica. Residual solid was then purified with flash chromatography (hexane/dichloromethane, 1:1. (v/v), to pure dichloromethane, to dichloromethane/ethyl acetate/triethylamine, 90:10:1, (v/v/v)) yielding glycoporphyrin **7f** (22.8 mg, 54%) as a violet-green crystalline solid. Analytical sample was obtained by crystallisation (THF/petroleum ether, solvents added at room temperature, heated to boiling and left in freezer for 18 h).

R_f = 0.72 (dichloromethane/ethyl acetate/triethylamine, 90:10:1, (v/v/v)), mp: > 220 °C.

^{1}H NMR (600 MHz, $CDCl_3$) δ [ppm]: 9.48 (d, J = 4.6 Hz, 4H, H_β), 8.75 (d, J = 4.6 Hz, 4H, H_β), 8.11 – 8.08 (m, 4H, H-2,2'$_{aryl}$), 7.78 – 7.75 (m, 4H, H-3,3'$_{aryl}$), 7.46 (br s, 2H, H-1$_{glycal}$), 6.07 (d, J = 5.2 Hz, 2H, H-3$_{glycal}$), 5.46 (dd, J = 6.4, 5.2 Hz, 2H, H-4$_{glycal}$), 4.61 – 4.55 (m, 4H, H-5,6$_{glycal}$), 4.37 (dd, J = 11.8, 2.5 Hz, 2H, H-6'$_{glycal}$), 2.24 (s, 6H, C$\underline{H}_3$COO-), 2.19 (s, 6H, C$\underline{H}_3$COO-), 2.18 (s, 6H, C$\underline{H}_3$COO-), -2.04 (br s, 2H, NH).

^{13}C NMR (151 MHz, $CDCl_3$) δ [ppm]: 170.7, 170.5, 169.7, 151.6, 139.9, 135.5, 134.7, 127.3, 120.5, 101.6, 98.1, 92.5, 92.1, 74.9, 67.4, 66.9, 61.4, 21.3, 21.0, 20.9.

UV-Vis (DMF) λ_{max} [nm] (log ε): 697.1 (4.07), 607.0 (4.43), 445.3 (5.30, Soret band).

MS (ESI+, isotopic profile) *m/z* (% rel. int.): $[M+H]^+$ calcd for $C_{60}H_{49}Cl_2N_4O_{14}$ 1119.3; found 1125.3 (2), 1124.3 (9), 1123.3 (18), 1122.3 (43), 1121.3 (75), 1120.3 (64), 1119.3 (100).

HRMS (ESI+) *m/z*: $[M+H]^+$ calcd for $C_{60}H_{49}Cl_2N_4O_{14}$ 1119.2617; found 1119.2592.

5,15-bis(2-(3,4,6-tri-O-acetyl-D-glucal-2-yl)ethynyl)-10,20-bis(3-bromophenyl)porphyrin (**7g**)

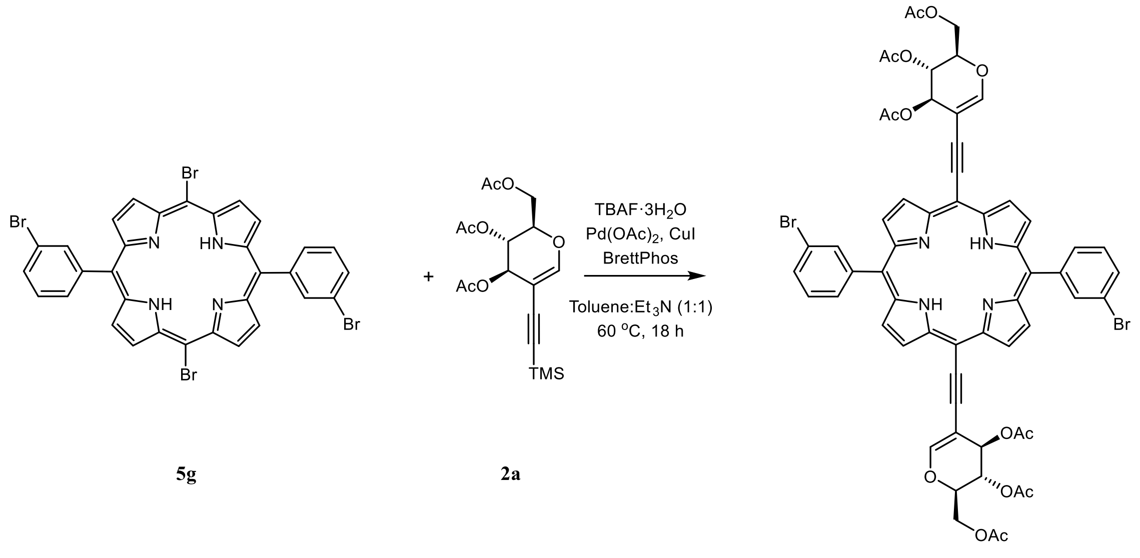

5g **2a** **7g**

To a sealed tube porphyrin **5g** (29.6 mg, 0.038 mmol, 1.0 eq), glucal **2a** (56.0 mg, 0.152 mmol, 4.0 eq), TBAF·3H_2O (49.0 mg, 0.155 mmol, 4.1 eq), Pd(OAc)$_2$ (0.9 mg, 0.0038 mmol, 0.10 eq), CuI (0.7 mg, 0.0038 mmol, 0.10 eq), BrettPhos (4.1 mg, 0.0076 mmol, 0.20 eq) and toluene (2.5 ml) were added. Next, reaction vessel was flushed with argon and triethylamine (2.5 ml) was added. Reaction was stirred for 18 h at 60°C. Upon completion, reaction vessel was washed with chloroform and diluted reaction mixture was concentrated to dryness with silica. Residual solid was then purified with flash chromatography (hexane/dichloromethane, 1:1. (v/v), to pure dichloromethane, to dichloromethane/ethyl acetate/triethylamine, 90:10:1, (v/v/v)) yielding glycoporphyrin **7g** (23.5 mg, 51%) as a violet-green crystalline solid. Analytical sample was obtained by crystallisation (THF/petroleum ether, solvents added at room temperature, heated to boiling and left in freezer for 18 h).

R$_f$ = 0.53 (dichloromethane/ethyl acetate/triethylamine, 90:10:1, (v/v/v)), mp: > 220 °C.

^{1}H NMR (600 MHz, CDCl$_3$) δ [ppm]: 9.49 (d, J = 4.6 Hz, 4H, H_β), 8.76 (d, J = 4.6 Hz, 4H, H_β), 8.33 (d, J = 1.8 Hz, 2H, H-2$_{aryl}$), 8.10 (d, J = 7.5 Hz, 2H, H-4$_{aryl}$),7.96 (dd, J = 8.2, 1.1 Hz, 2H, H-6$_{aryl}$), 7.65 (t, J = 7.8 Hz, 2H, H-5$_{aryl}$), 7.46 (br s, 2H, H-1$_{glycal}$), 6.08 (d, J = 5.2 Hz, 2H, H-3$_{glycal}$), 5.46 (t, J = 5.7 Hz, 2H, H-4$_{glycal}$), 4.63 – 4.56 (m, 4H, H-5,6$_{glycal}$), 4.40 – 4.36 (m, 2H, H-6'$_{glycal}$), 2.26 – 2.24 (m, 6H, C$\underline{H}_3$COO-), 2.19 (s, 6H, C$\underline{H}_3$COO-), 2.18 (s, 6H, C$\underline{H}_3$COO-), -2.05 (br s, 2H, NH).

^{13}C NMR (151 MHz, CDCl$_3$) δ [ppm]: 170.7, 170.5, 169.7, 151.6, 143.5, 137.1, 133.1, 131.4, 128.5, 121.4, 120.1, 101.8, 98.1, 92.5, 92.2, 74.9, 67.4, 66.9, 61.4, 21.3, 21.0, 20.9.

UV-Vis (DMF) λ_{max} [nm] (log ε): 696.2 (4.12), 607.0 (4.48), 445.9 (5.35, Soret band).

MS (ESI+, isotopic profile) *m/z* (% rel. int.): $[M+H]^+$ calcd for $C_{60}H_{49}Br_2N_4O_{14}$ 1207.2; found 1214.2 (3), 1213.2 (13), 1212.2 (34), 1211.2 (58), 1210.2 (65), 1209.2 (100), 1208.2 (32), 1207.2 (47).

HRMS (ESI+) *m/z*: $[M+H]^+$ calcd for $C_{60}H_{49}Br_2N_4O_{14}$ 1207.1607; found 1207.1591.

5,15-bis(2-(3,4,6-tri-O-acetyl-D-glucal-2-yl)ethynyl)-10,20-bis(3-cyanophenyl)porphyrin (**7h**)

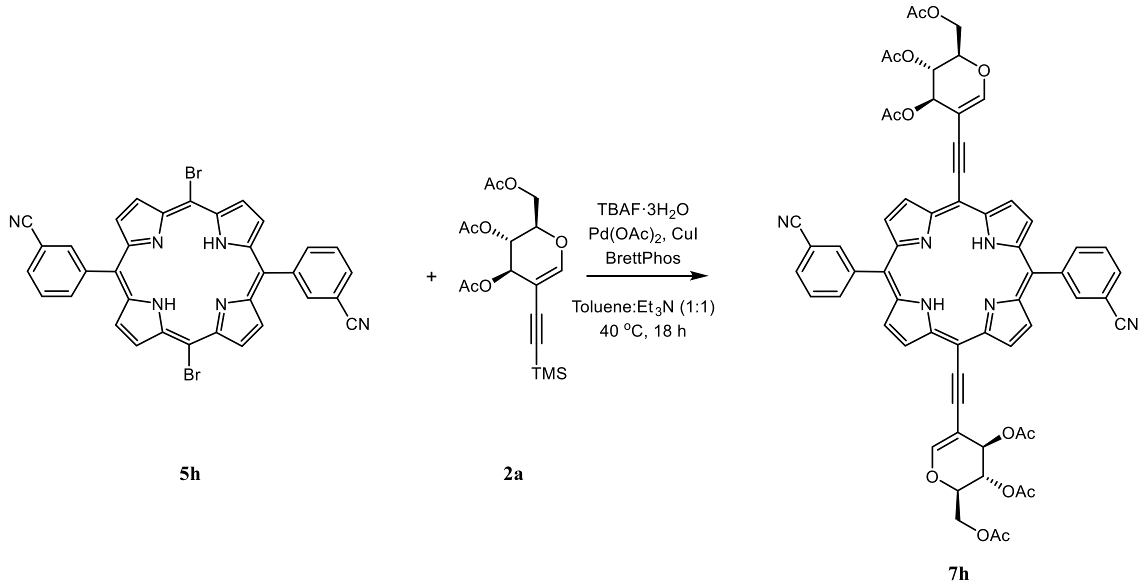

To a sealed tube porphyrin **5h** (25.5 mg, 0.038 mmol, 1.0 eq), glucal **2a** (56.0 mg, 0.152 mmol, 4.0 eq), TBAF·3H_2O (49.0 mg, 0.155 mmol, 4.1 eq), $Pd(OAc)_2$ (0.9 mg, 0.0038 mmol, 0.10 eq), CuI (0.7 mg, 0.0038 mmol, 0.10 eq), BrettPhos (4.1 mg, 0.0076 mmol, 0.20 eq) and toluene (2.5 ml) were added. Next, reaction vessel was flushed with argon and triethylamine (2.5 ml) was added. Reaction was stirred for 18 h at 40°C. Upon completion, reaction vessel was washed with dichloromethane and diluted reaction mixture was concentrated to dryness with silica. Residual solid was then purified with flash chromatography (hexane/dichloromethane, 1:1. (v/v), to pure dichloromethane, to dichloromethane/ethyl acetate/triethylamine, 90:10:1, (v/v/v)) yielding after crystallisation (THF/petroleum ether, solvents added at room temperature, heated to boiling and left in freezer for 18 h) glycoporphyrin **7h** (12.9 mg, 31%) as a violet-green crystalline solid.

R$_f$= 0.36 (dichloromethane/ethyl acetate/triethylamine, 90:10:1, (v/v/v)), mp: > 220 °C.

^{1}H NMR (600 MHz, $CDCl_3$) δ [ppm]: 9.52 (br s, 4H, H_β), 8.66 (d, J = 4.7 Hz, 4H, H_β), 8.56 (br s, 2H, H-2_{aryl}), 8.40 (d, J = 7.6 Hz, 2H, H-4_{aryl}), 8.13 (dt, J = 8.0, 1.4 Hz, 2H, H-6_{aryl}), 7.92 (t, J = 7.8 Hz, 2H, H-5_{aryl}), 7.49 (br s, 2H, H-1_{glycal}), 6.09 (d, 2H, J = 5.2 Hz H-3_{glycal}), 5.46 (t, J = 5.7 Hz, 2H, H-4_{glycal}), 4.63 – 4.58 (m, 4H, H-$5,6_{glycal}$), 4.41 – 4.37 (m, 2H, H-$6'_{glycal}$), 2.27 – 2.22 (m, 6H, C<u>H</u>$_3$COO-), 2.19 (s, 6H, C<u>H</u>$_3$COO-), 2.18 (s, 6H, C<u>H</u>$_3$COO-), -2.04 (br s, 2H, NH).

^{13}C NMR (151 MHz, $CDCl_3$) δ [ppm]: 170.7, 170.5, 169.7, 151.9, 142.8, 138.2, 137.0, 132.0, 128.1, 119.0, 98.0, 75.0, 67.3, 66.9, 61.3, 21.3, 21.0, 20.9.

UV-Vis (DMF) λ_{max} [nm] (log ε): 696.1 (3.98), 607.0 (4.35), 445.9 (5.22, Soret band).

MS (ESI+, isotopic profile) m/z (% rel. int.): $[M+H]^+$ calcd for $C_{62}H_{49}N_6O_{14}$ 1101.3; found 1104.3 (7), 1104.3 (26), 1104.3 (68), 1104.3 (100).

HRMS (ESI+) m/z: $[M+H]^+$ calcd for $C_{62}H_{49}N_6O_{14}$ 1101.3301; found 1101.3284.

3.2 Synthesis and characterisation of deprotected glycoporphyrin **8a**

5,15-bis(2-(D-glucal-2-yl)ethynyl)-10,20-diphenylporphyrin (**8a**)

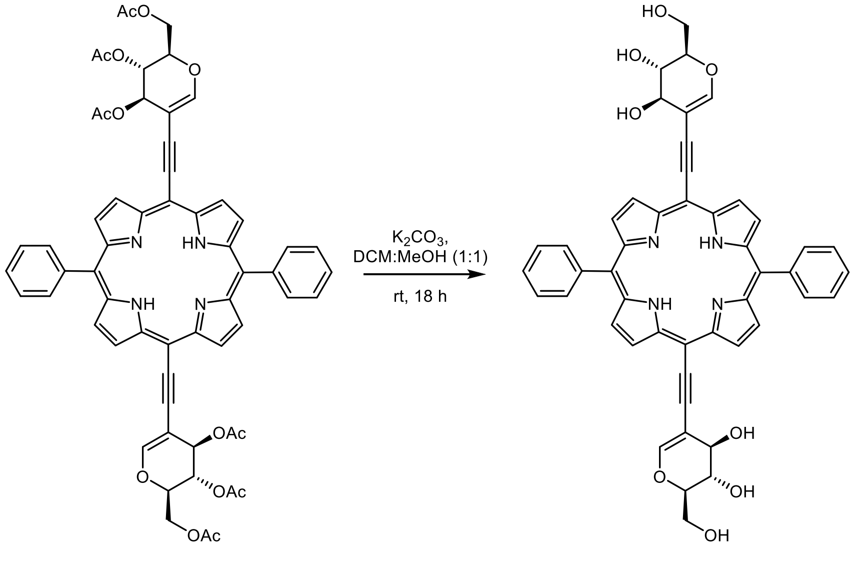

To a sealed tube glycoporphyrin **6a** (100.0 mg, 0.095 mmol, 1.0 eq), K_2CO_3 (78.9 mg, 0.571 mmol, 6.0 eq), dichloromethane (2.5 ml) and methanol (2.5 ml) were added. Reaction was stirred for 18 h at room temperature. Upon completion, reaction vessel was washed with mixture of DCM and MeOH (1:1, (v/v)) and diluted reaction mixture was concentrated to dryness with silica. Residual solid was then purified with flash chromatography (dichloromethane/methanol, 9:1. (v/v), to dichloromethane/methanol, 8:2. (v/v), dichloromethane/methanol, 1:1. (v/v)). After drying, product was washed with distilled water and dissolved with a mixture of distilled water and acetone (1:1, (v/v)). It was then concentrated to dryness yielding deprotected glycoporphyrin **8a** (63.7 mg, 84%) as a violet-green crystalline solid. Analytical sample was obtained by crystallisation (THF/petroleum ether, solvents added at room temperature, heated to boiling and left in freezer for 18 h).

$\boldsymbol{R_f}$ = 0.23 (dichloromethane/methanol, 9:1, (v/v)), mp: > 220 °C.

^{1}H NMR (600 MHz, DMSO-d$_6$) δ [ppm]: 9.69 (d, J = 4.7 Hz, 4H, H_β), 8.71 (d, J = 4.6 Hz, 4H, H_β), 8.22 – 8.19 (m, 4H, H-2,2'$_{aryl}$), 7.90 – 7.83 (m, 4H, H-3,3',4$_{aryl}$), 7.58 (d, J = 1.2 Hz, 2H, H-1$_{glycal}$), 5.90 (d, J = 7.0 Hz, 2H, OH-C-3$_{glycal}$), 5.53 (d, J = 5.4 Hz, 2H, OH-C-4$_{glycal}$), 4.87 (t, J = 5.9 Hz, 2H, OH-C-5$_{glycal}$), 4.43 (t, J = 6.7 Hz 2H, H-3$_{glycal}$), 4.06 – 4.02 (m, 2H, H-5$_{glycal}$), 4.61 – 4.53 (m, 2H, H-6$_{glycal}$), 3.82 – 3.75 (m, 2H, H-5,6'$_{glycal}$), -1.87 (br s, 2H, NH).

^{13}C NMR (151 MHz, DMSO-d$_6$) δ [ppm]: 151.3, 140.5, 134.3, 128.3, 127.2, 121.5, 102.4, 101.5, 97.2, 91.0, 80.9, 69.3, 68.2, 60.1.

UV-Vis (DMF) λ_{max} [nm] (log ε): 705.3 (4.24), 616.8 (4.56), 449.4 (5.34, Soret band).

MS (ESI+, isotopic profile) m/z (% rel. int.): $[M+H]^+$ calcd for $C_{48}H_{39}N_4O_8$ 799.3; found 802.3 (4), 801.3 (16), 800.3 (53), 799.3 (100).

HRMS (ESI+) m/z: $[M+H]^+$ calcd for $C_{48}H_{39}N_4O_8$ 799.2762; found 799.2773.

4. Synthesis of known porphyrins for ROS studies

5,10,15,20-tetraphenylporphyrin (**TPP**)

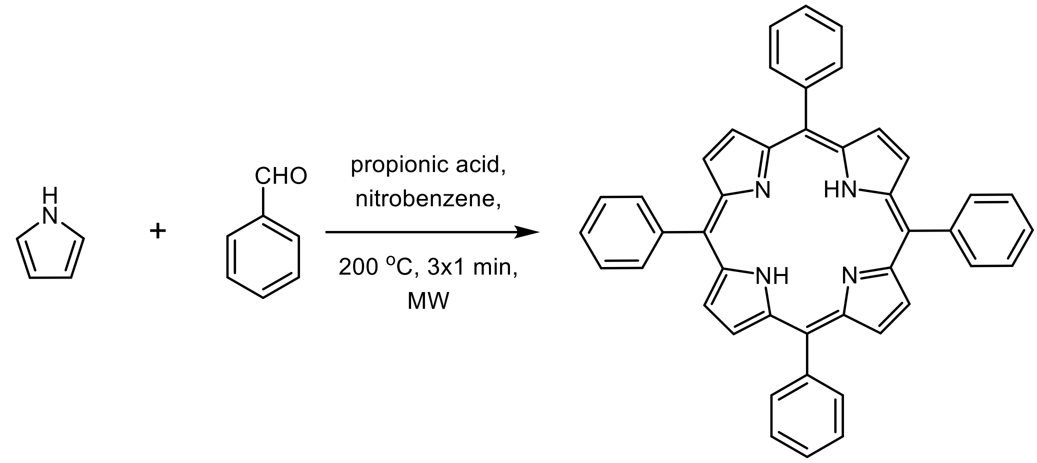

TPP

To the 10 ml microwave reactor, pyrrole (0.69 ml, 671 mg, 10.0 mmol, 1.0 eq), benzaldehyde (1.02 ml, 1061 mg, 10.0 mmol, 1.0 eq), propionic acid (3.5 ml) and nitrobenzene (1.5 ml) were added. Reactor was then sealed. Reaction mixture was rapidly heated in microwaves to 200 °C and kept at this temperature for 1 min. Next, it was cooled down to 70 °C. This cycle was repeated 3 times. On the last cycle, reaction mixture was cooled down to 55 °C. Upon completion, reaction mixture was transferred to round-bottom flask (using dichloromethane to dilute the solution) and concentrated to dryness with silica. Residual solid was then purified with flash chromatography (pure dichloromethane) yielding porphyrin **TPP** as a violet crystalline solid (410.5 mg, 27%). The characterisation data were in agreement with those of the literature.[16]

$\boldsymbol{R_f}$ = 0.49 (hexane/dichloromethane, 1:1, (v/v)).

^{1}H NMR (600 MHz, $CDCl_3$) δ [ppm]: 8.85 (s, 8H), 8.24 – 8.21 (m, 8H), 7.80 – 7.73 (m, 12H), -2.76 (br s, 2H).

5,15-diphenyl-10,20-bis((trimethylsilyl)ethynyl)porphyrin (**EtDPP**)

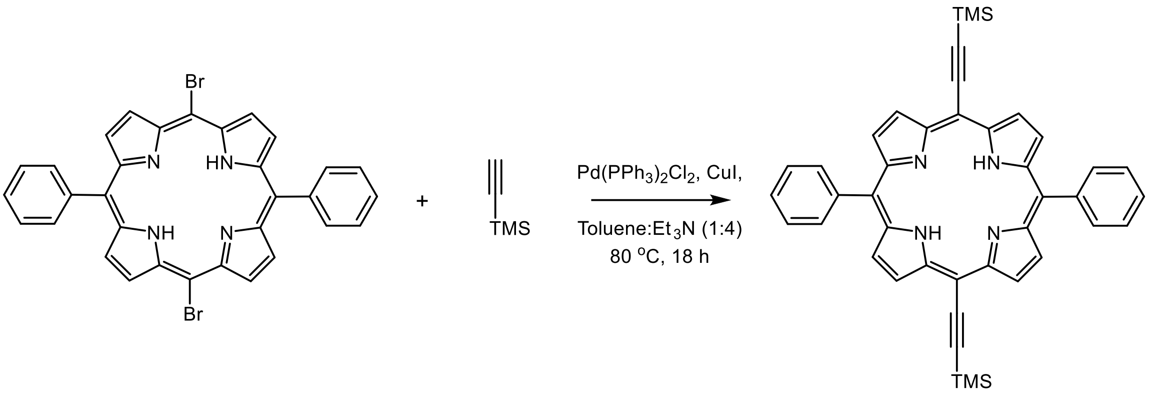

5a **EtDPP**

To a sealed tube porphyrin **5a** (50.0 mg, 0.081 mmol, 1.0 eq), ethyltrimethylsilane (49 µl, 34.9 mg, 0.355 mmol, 4.4 eq), $Pd(PPh_3)_2Cl_2$ (5.7 mg, 0.0081 mmol, 0.10 eq), CuI (1.7 mg, 0.0091 mmol, 0.11 eq) and toluene (1.7 ml) were added. Next, reaction vessel was flushed with argon and triethylamine (5.0 ml) was added. Reaction was stirred for 18 h at 80°C. Upon completion, reaction vessel was washed with chloroform and diluted reaction mixture was concentrated to dryness with silica. Residual solid was then purified with flash chromatography (hexane/dichloromethane, 2:1. (v/v)) yielding porphyrin **EtDPP** (36.4 mg, 69%) as a violet-red crystalline solid. The characterisation data were in agreement with those of the literature.[17]

$\boldsymbol{R_f}$ = 0.43 (hexane/dichloromethane, 2:1, (v/v)).

^{1}H NMR (600 MHz, $CDCl_3$) δ [ppm]: 9.62 (d, J = 4.6 Hz, 4H), 8.83 (d, J = 4.6 Hz, 4H), 8.20 – 8.18 (m, 4H), 7.82 – 7.76 (m, 6H), 0.61 (s, 18H), -2.18 (br s, 2H).

1 S. Dharuman and Y. D. Vankar, *Org. Lett.*, 2014, **16**, 1172–1175.

2 E. W. Mora Flores, D. Suarez, M. L. Uhrig and A. Postigo, *J. Org. Chem.*, 2023, **88**, 16803–16816.

3 J. T. Dixon, F. R. van Heerden and C. W. Holzapfel, *Tetrahedron: Asymmetry*, 2005, **16**, 393–401.

4 M. Malinowski, T. Van Tran, M. de Robichon, N. Lubin-Germain and A. Ferry, *Adv. Synth. Catal.*, 2020, **362**, 1184–1189.

5 B. Godlewski, D. Baran, M. de Robichon, A. Ferry, S. Ostrowski and M. Malinowski, *Org. Chem. Front.*, 2022, **9**, 2396–2404.

6 D. C. Koester and D. B. Werz, *Beilstein J. Org. Chem.*, 2012, **8**, 675–682.

7 A. J. F. . Sobral, N. G. C. . Rebanda, M. da Silva, S. H. Lampreia, M. Ramos Silva, A. M. Beja, J. . Paixão and A. M. d'. Rocha Gonsalves, *Tetrahedron Lett.*, 2003, **44**, 3971–3973.

8 R. S. Kumar, H. Kim, N. Mergu and Y.-A. Son, *Res. Chem. Intermed.*, 2020, **46**, 313–328.

9 S. Juillard, Y. Ferrand, G. Simonneaux and L. Toupet, *Tetrahedron*, 2005, **61**, 3489–3495.

10 M. Handayani, S. Gohda, D. Tanaka and T. Ogawa, *Chem. – A Eur. J.*, 2014, **20**, 7655–7664.

11 C.-H. Shu, Y.-L. Xie, A. Wang, K.-J. Shi, W.-F. Zhang, D.-Y. Li and P.-N. Liu, *Chem. Commun.*, 2018, **54**, 12626–12629.

12 Z. Abada, L. Ferrié, B. Akagah, A. T. Lormier and B. Figadère, *Tetrahedron Lett.*, 2011, **52**, 3175–3178.

13 A. T. Marques, S. M. A. Pinto, C. J. P. Monteiro, J. S. Seixas de Melo, H. D. Burrows, U. Scherf, M. J. F. Calvete and M. M. Pereira, *J. Polym. Sci. Part A Polym. Chem.*, 2012, **50**, 1408–1417.

14 G. Kuang, Q. Zhang, D. Y. Li, X. S. Shang, T. Lin, P. N. Liu and N. Lin, *Chem. – A Eur. J.*, 2015, **21**, 8028–8032.

15 Y. Yamada, T. Kubota, M. Nishio and K. Tanaka, *J. Am. Chem. Soc.*, 2014, **136**, 6505–6509.

16 H. He, Z.-Y. Qiu, Z. Yin, J. Kong, J.-S. Dang, H. Lei, W. Zhang and R. Cao, *Chem. Commun.*, 2024, **60**, 5916–5919.

17 B. Temelli, M. Gündüz and D. Yüksel, *Tetrahedron*, 2018, **74**, 4476–4488.

5. Reactive oxygen species generation plots of new glycoporphyrins

ROS generation plot of **6a** irradiated with 690 nm light

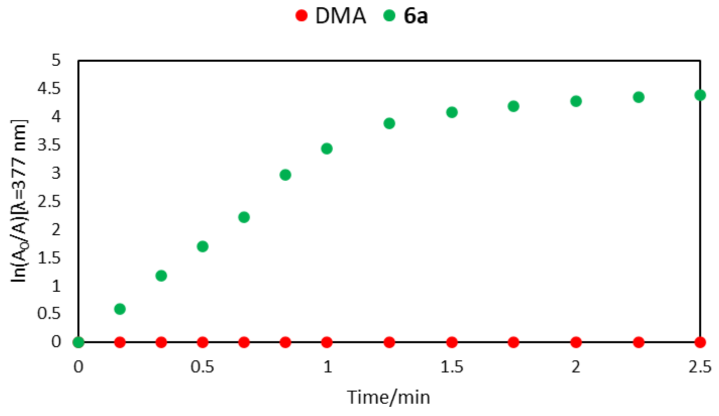

ROS generation plot of **6b** irradiated with 690 nm light

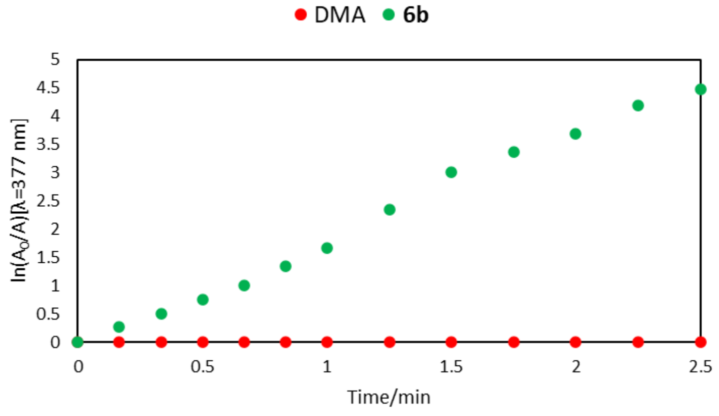

ROS generation plot of **6c** irradiated with 690 nm light

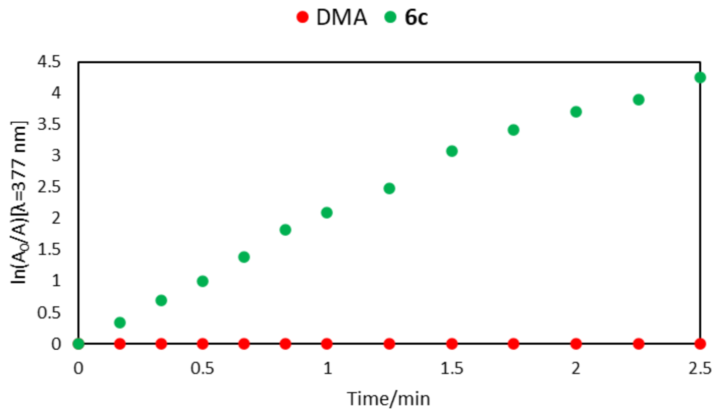

ROS generation plot of **6d** irradiated with 690 nm light

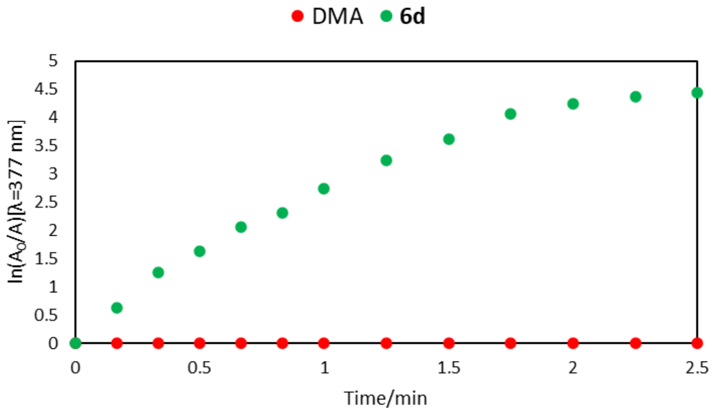

ROS generation plot of **7b** irradiated with 690 nm light

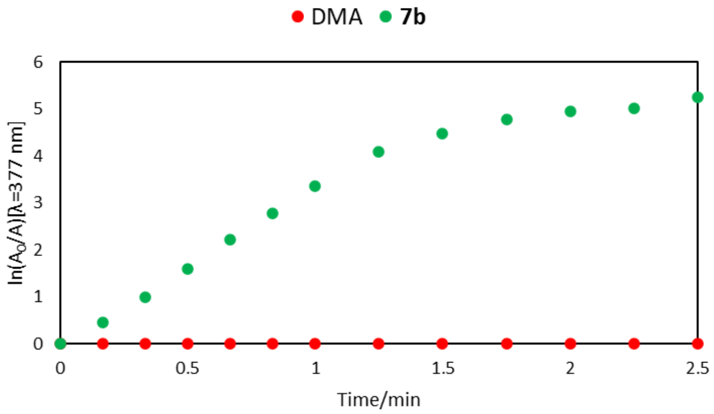

ROS generation plot of **7c** irradiated with 690 nm light

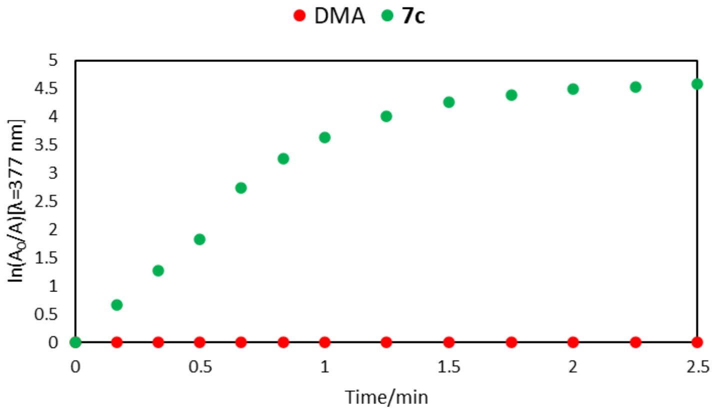

ROS generation plot of **7d** irradiated with 690 nm light

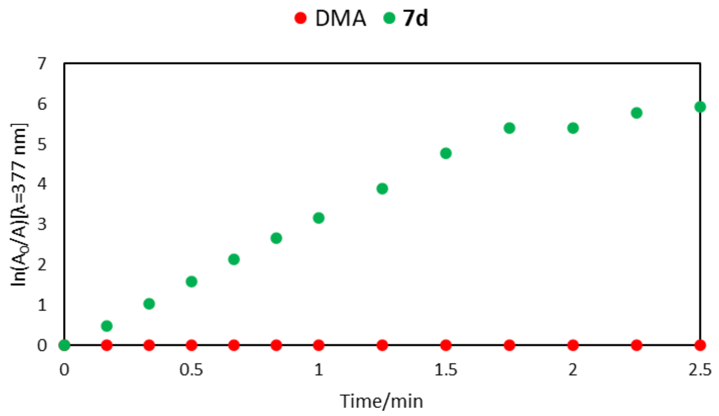

ROS generation plot of **7e** irradiated with 690 nm light

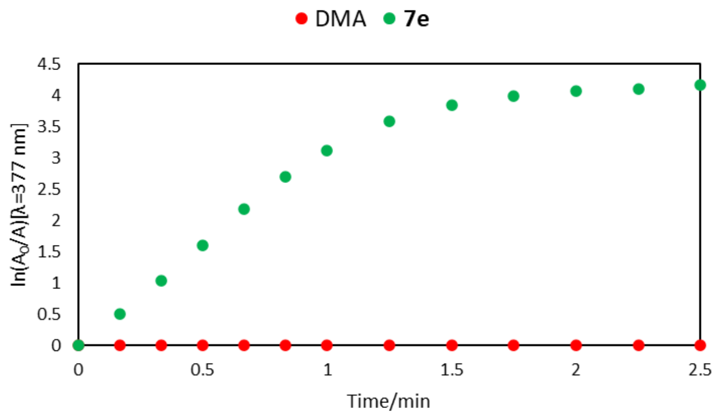

ROS generation plot of **7f** irradiated with 690 nm light

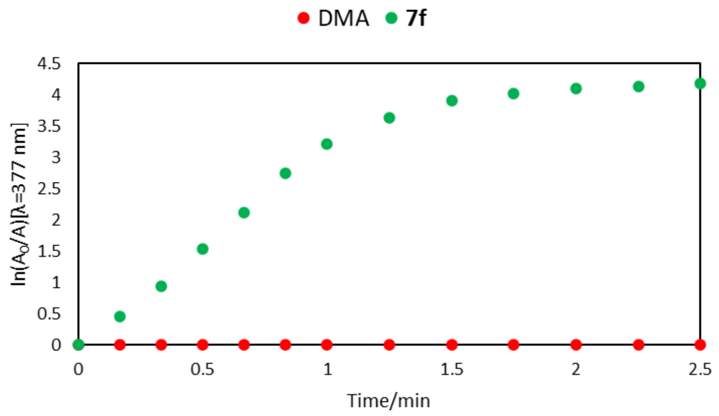

ROS generation plot of **7g** irradiated with 690 nm light

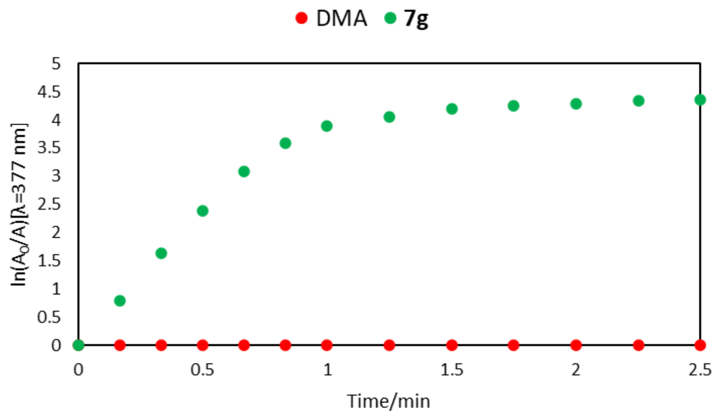

ROS generation plot of **7h** irradiated with 690 nm light

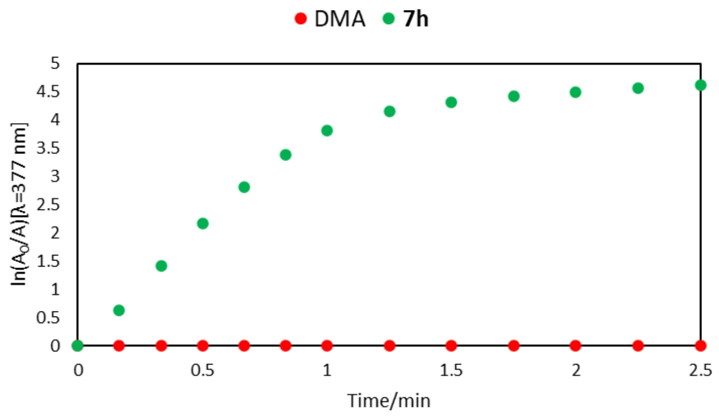

ROS generation plot of **8a** irradiated with 690 nm light

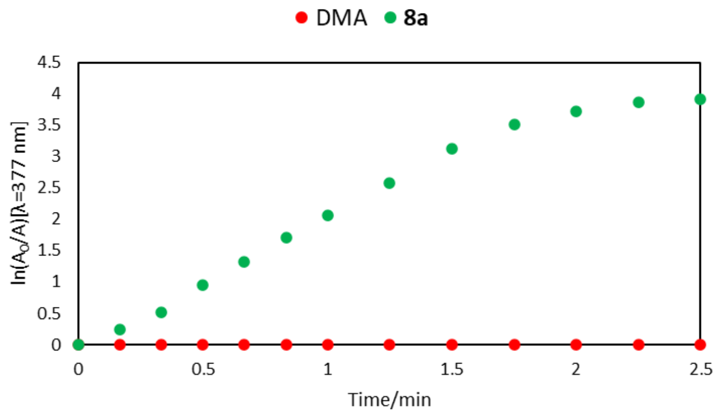

ROS generation plot of **8a** irradiated with 740 nm light

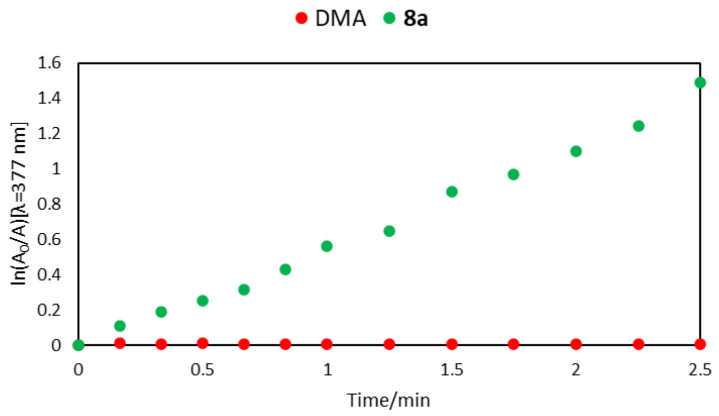

6. UV-vis spectra of new compounds

UV-vis absorbance spectrum of 4b in $CHCl_3$

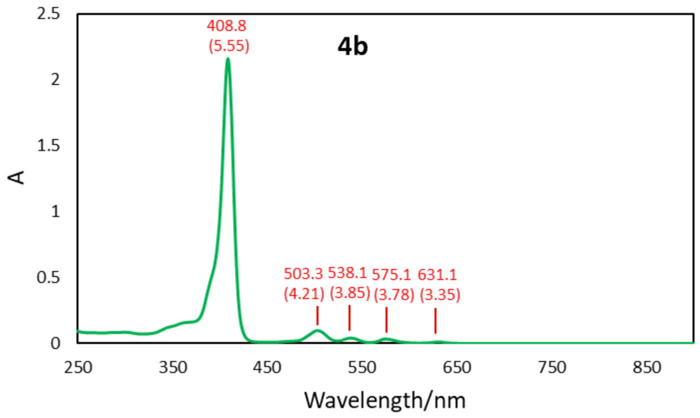

UV-vis absorbance spectrum of 4e in $CHCl_3$

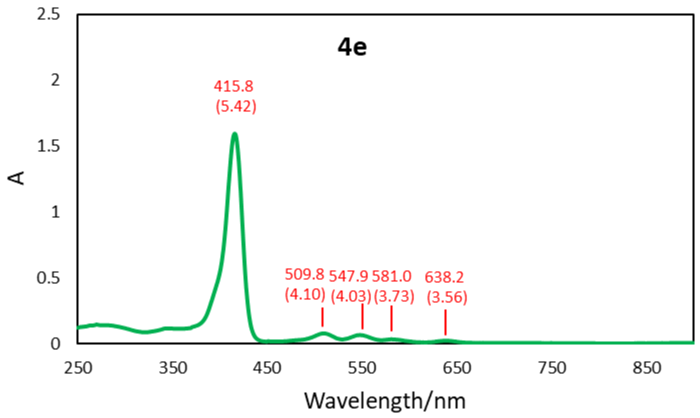

UV-vis absorbance spectrum of 4h in $CHCl_3$

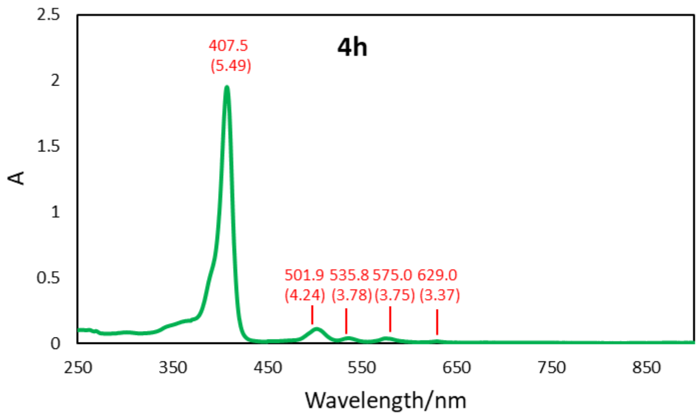

UV-vis absorbance spectrum of 5b in $CHCl_3$

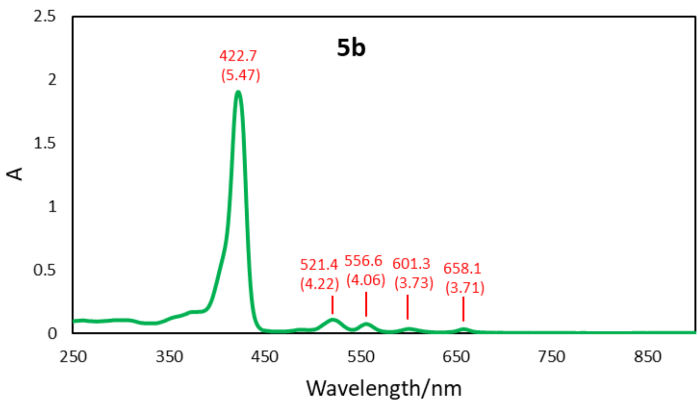

UV-vis absorbance spectrum of 5c in $CHCl_3$

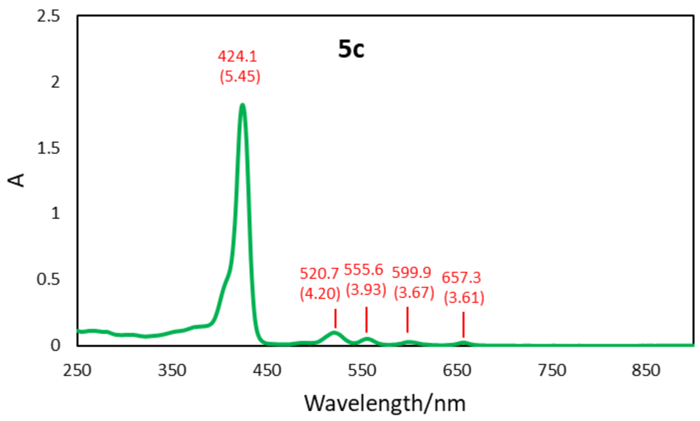

UV-vis absorbance spectrum of 5e in $CHCl_3$

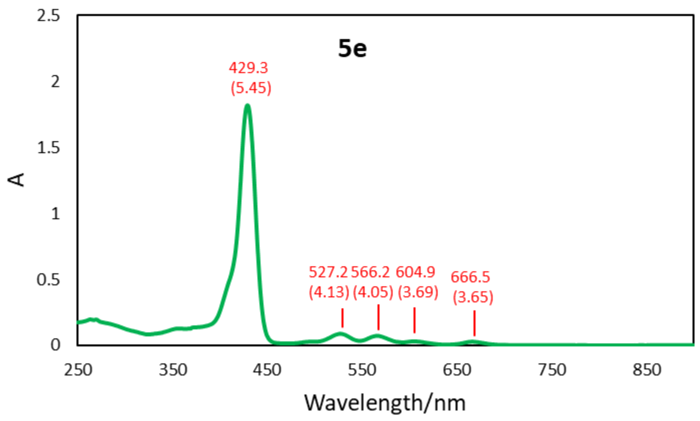

UV-vis absorbance spectrum of 5f in $CHCl_3$

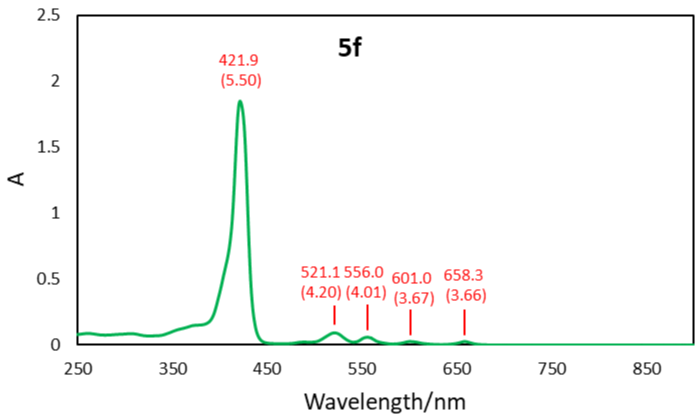

UV-vis absorbance spectrum of 5g in $CHCl_3$

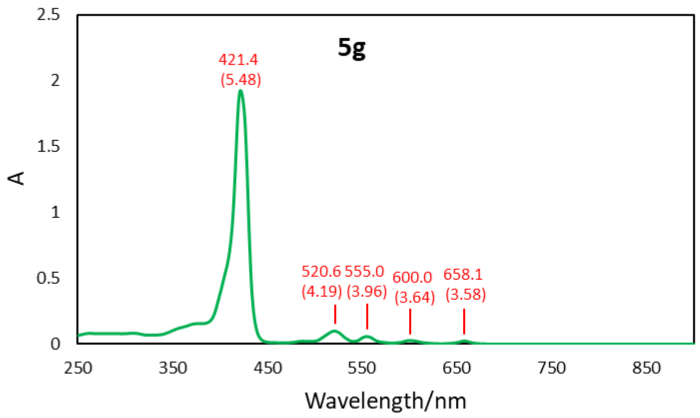

UV-vis absorbance spectrum of 5h in $CHCl_3$

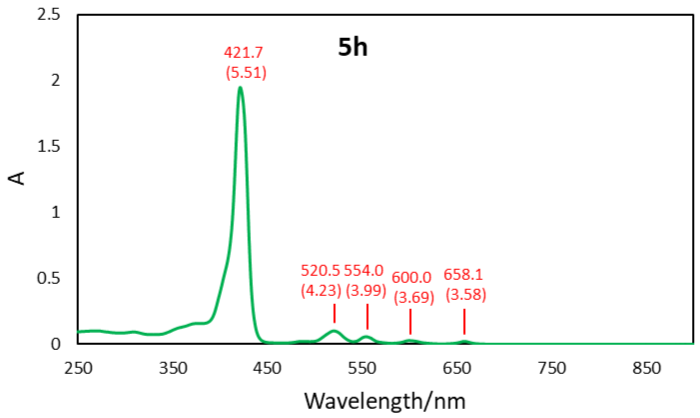

UV-vis absorbance spectrum of 6a in DMF

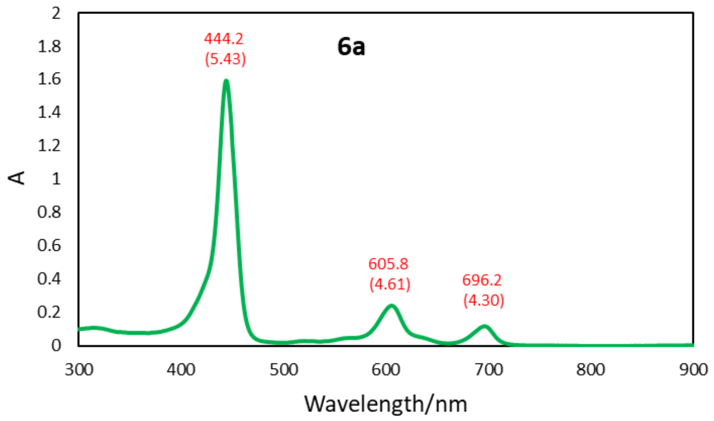

UV-vis absorbance spectrum of 6b in DMF

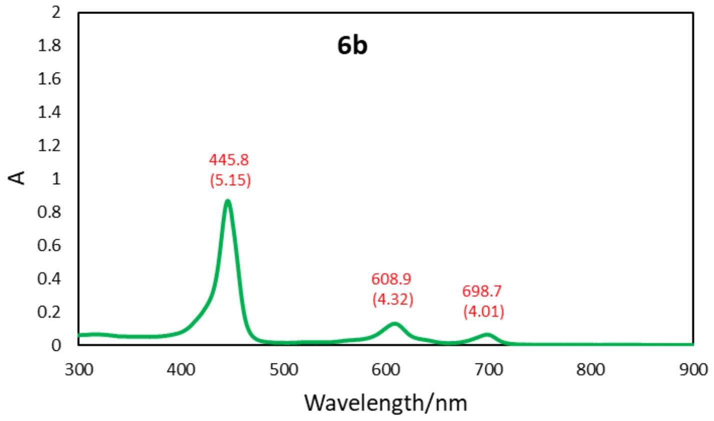

UV-vis absorbance spectrum of 6c in DMF

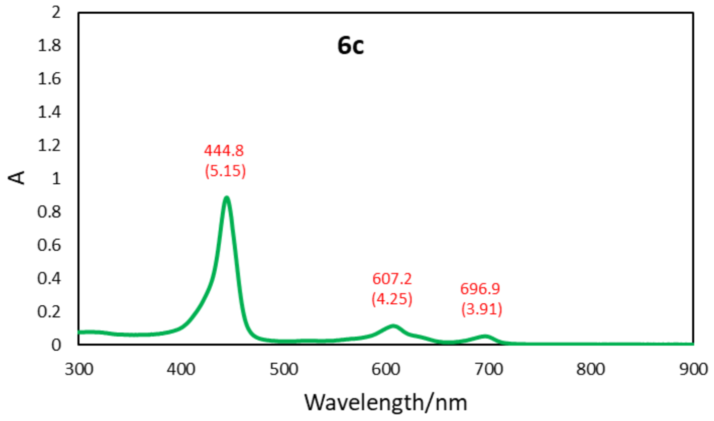

UV-vis absorbance spectrum of 6d in DMF

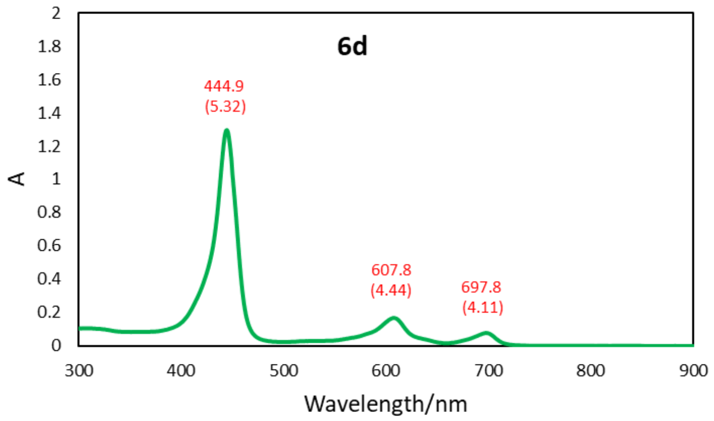

UV-vis absorbance spectrum of 7b in DMF

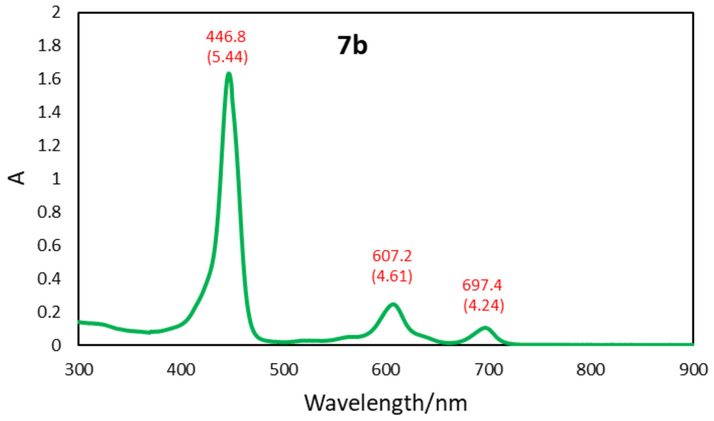

UV-vis absorbance spectrum of 7c in DMF

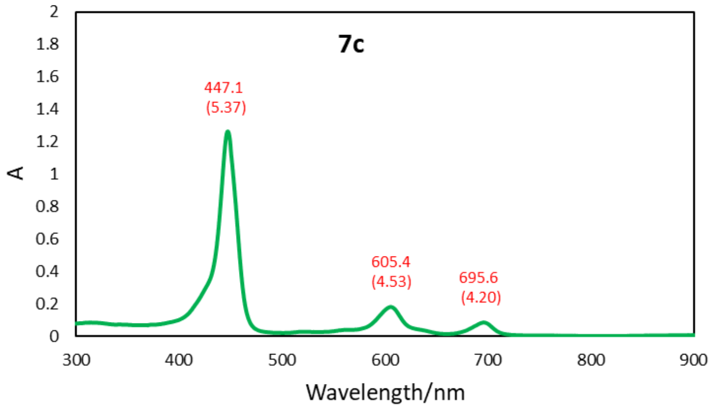

UV-vis absorbance spectrum of 7d in DMF

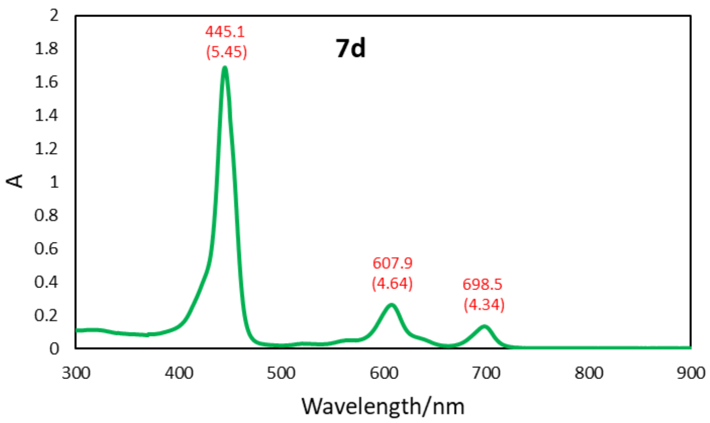

UV-vis absorbance spectrum of 7e in DMF

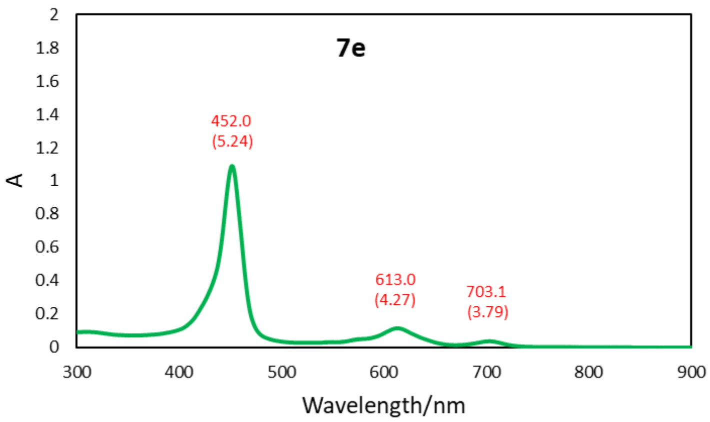

UV-vis absorbance spectrum of 7f in DMF

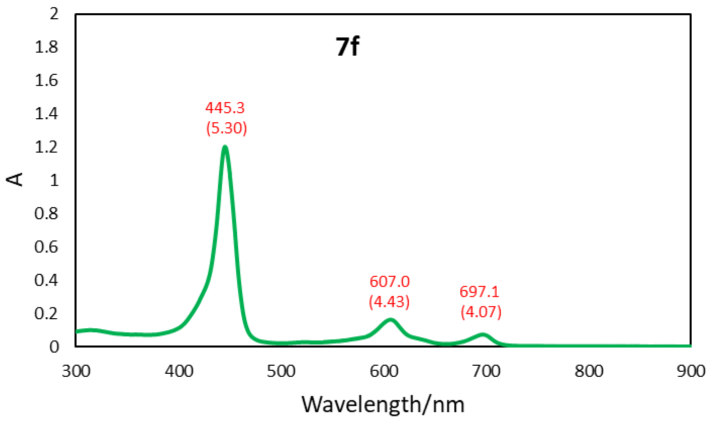

UV-vis absorbance spectrum of 7g in DMF

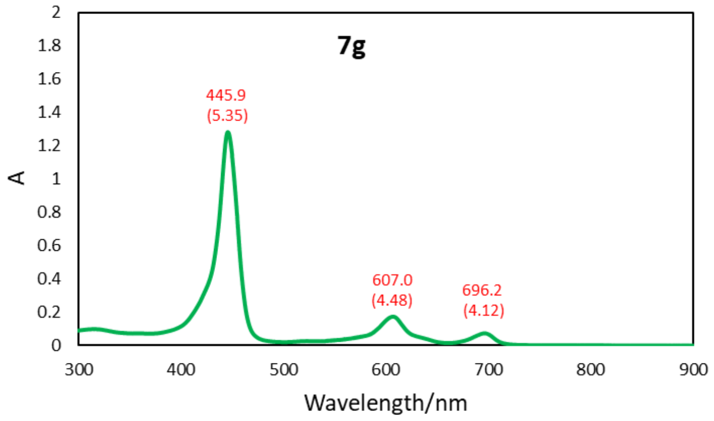

UV-vis absorbance spectrum of 7h in DMF

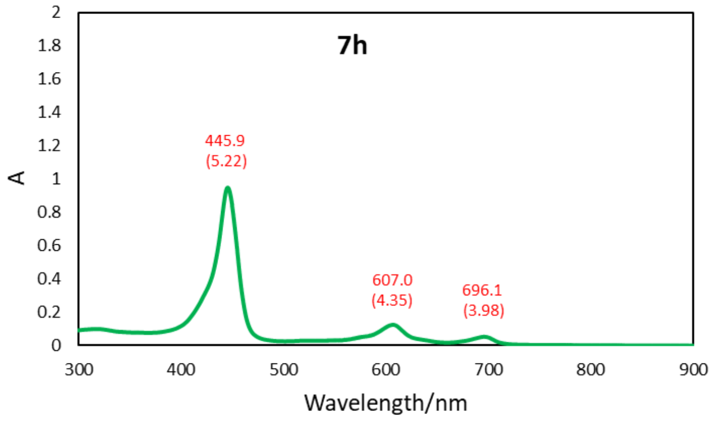

UV-vis absorbance spectrum of 8a in DMF

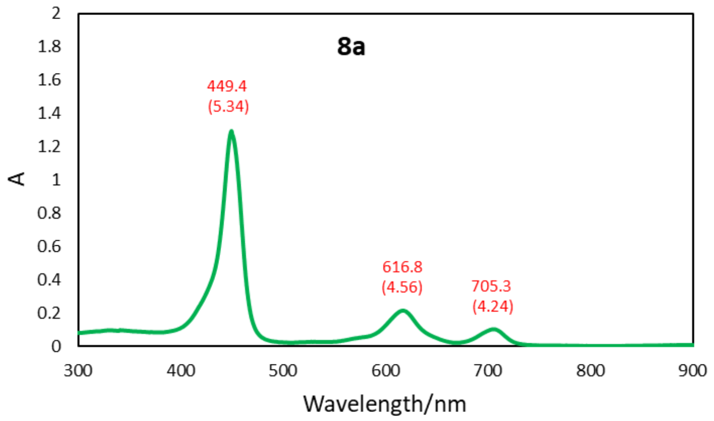

7. NMR spectra of known compounds

^{1}H NMR (600MHz, $CDCl_3$) of 1a

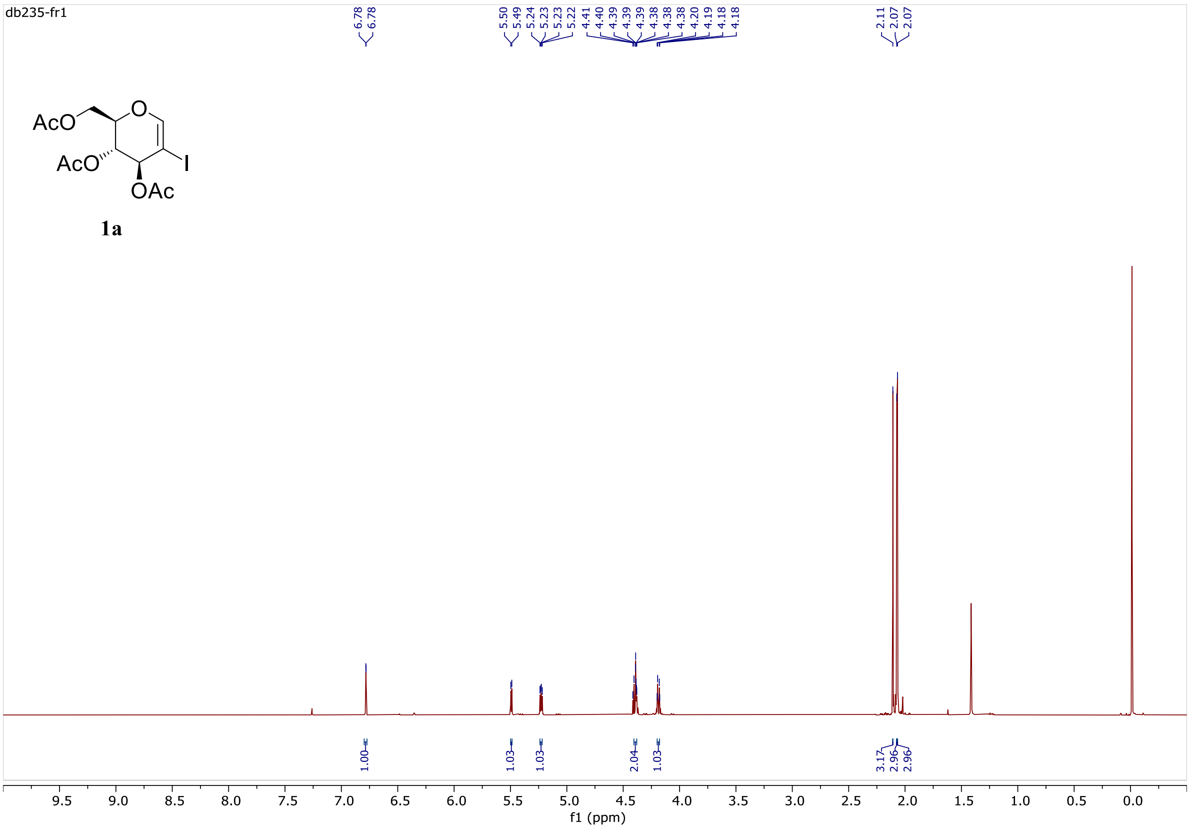

^{1}H NMR (500MHz, $CDCl_3$) of 1b

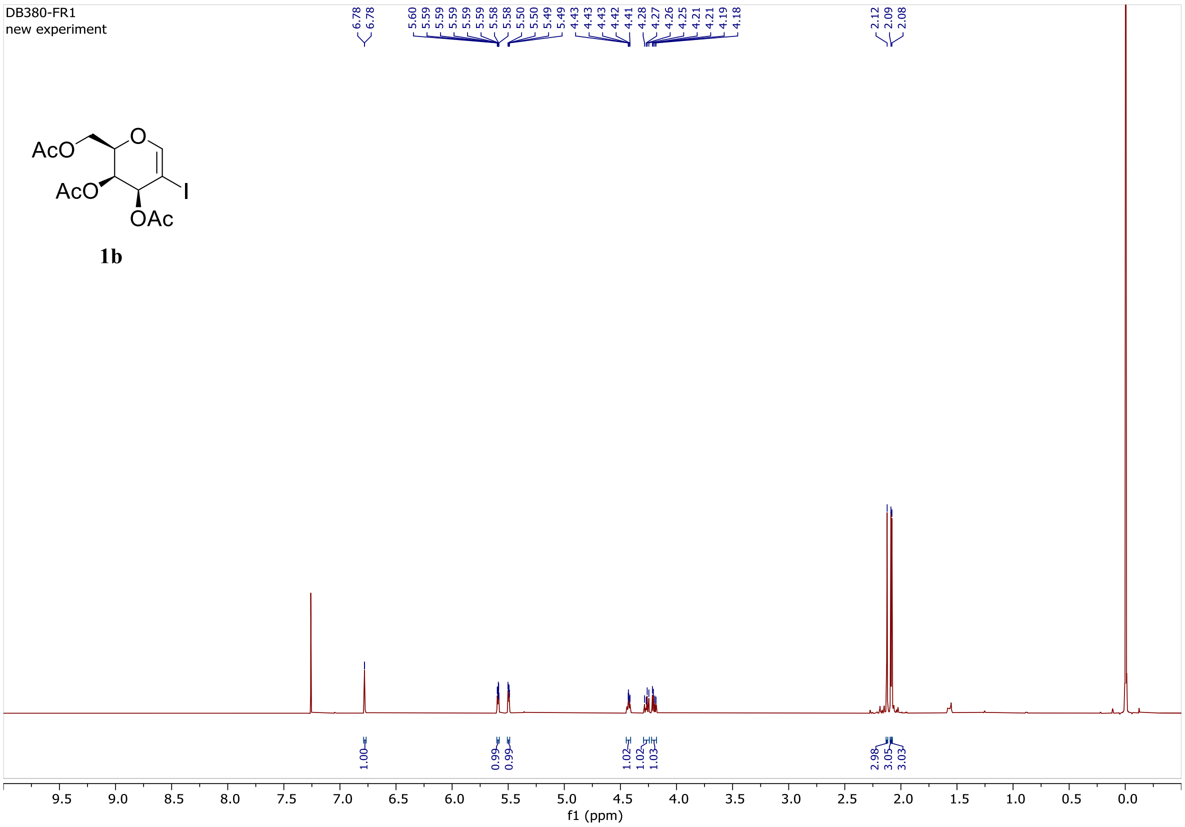

^{1}H NMR (600MHz, $CDCl_3$) of SI6

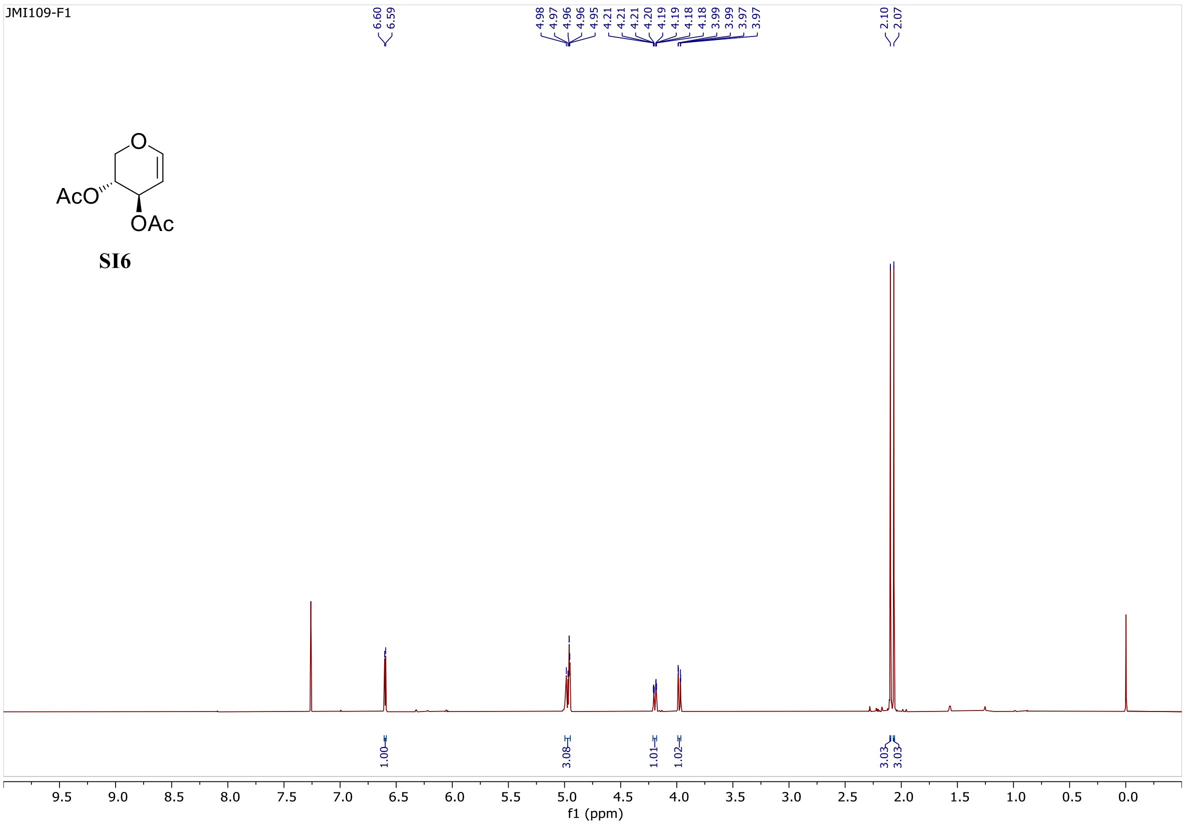

^{1}H NMR (600MHz, $CDCl_3$) of 1c

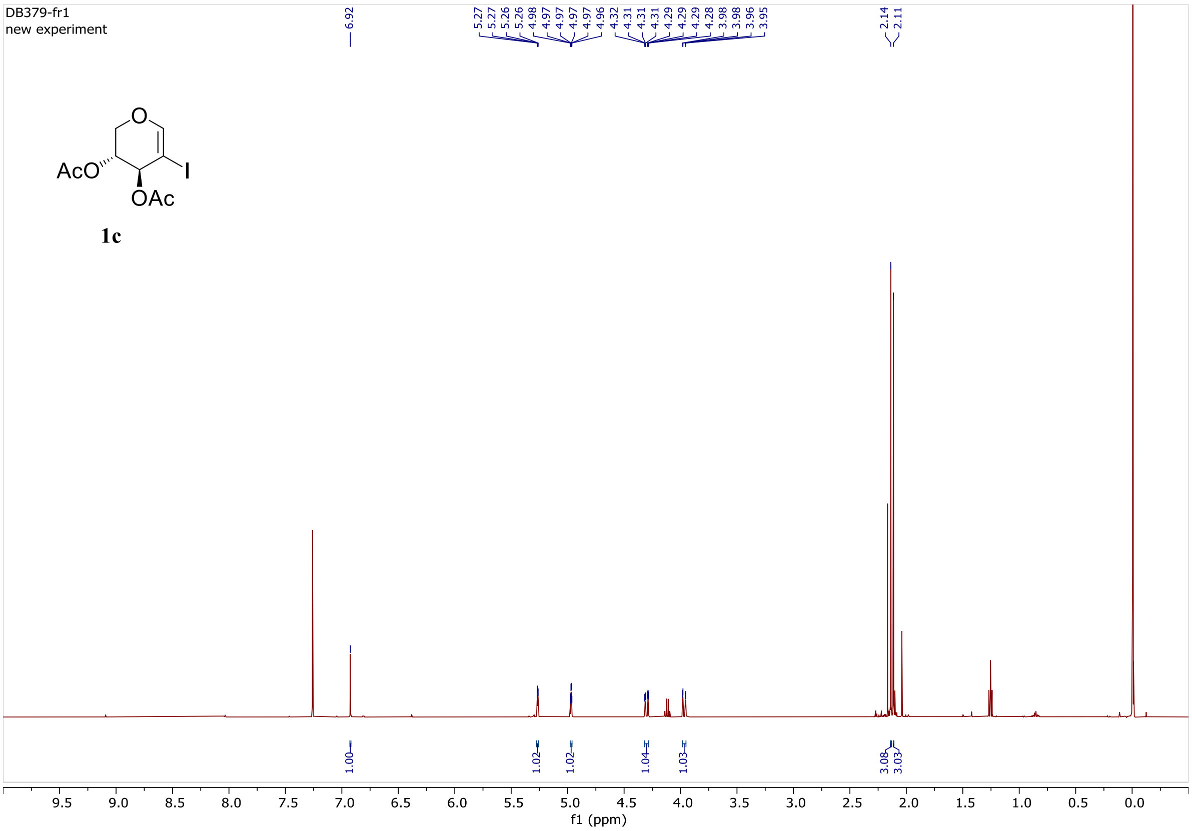

^{1}H NMR (500MHz, $CDCl_3$) of SI10

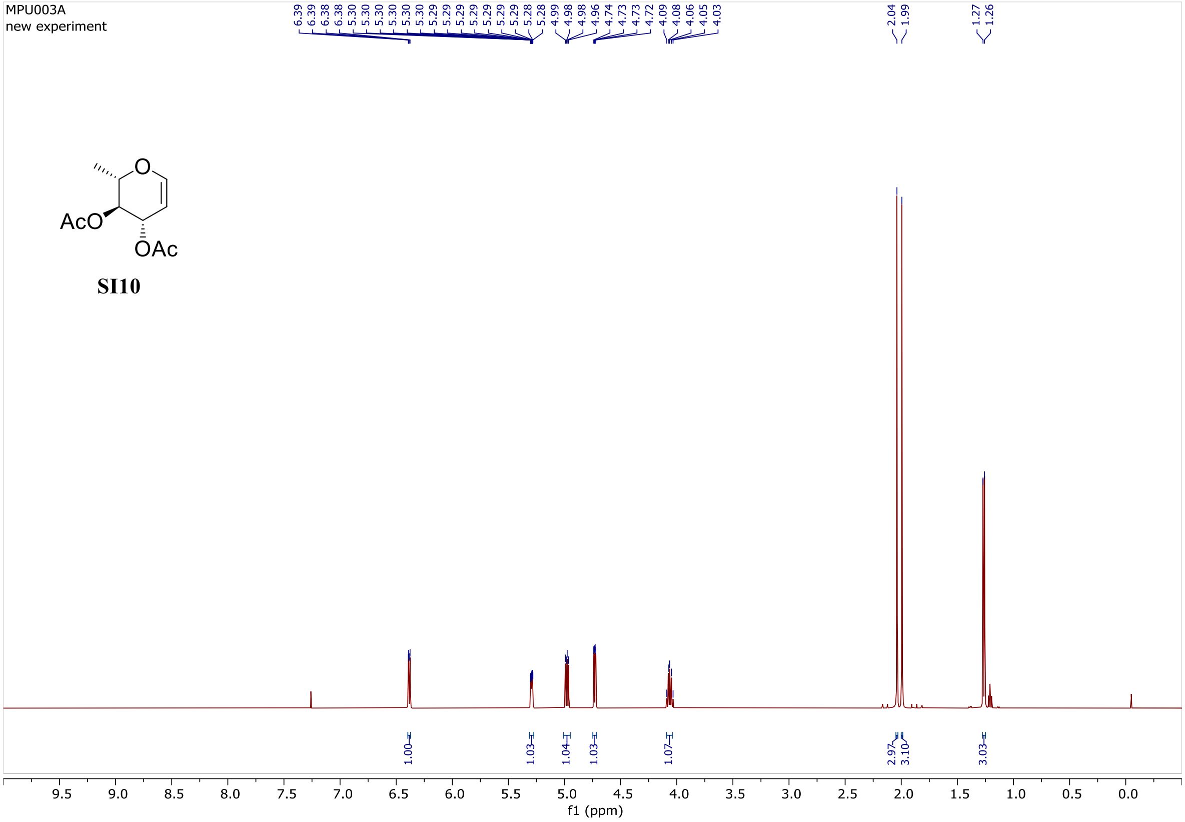

^{1}H NMR (600MHz, $CDCl_3$) of 1d

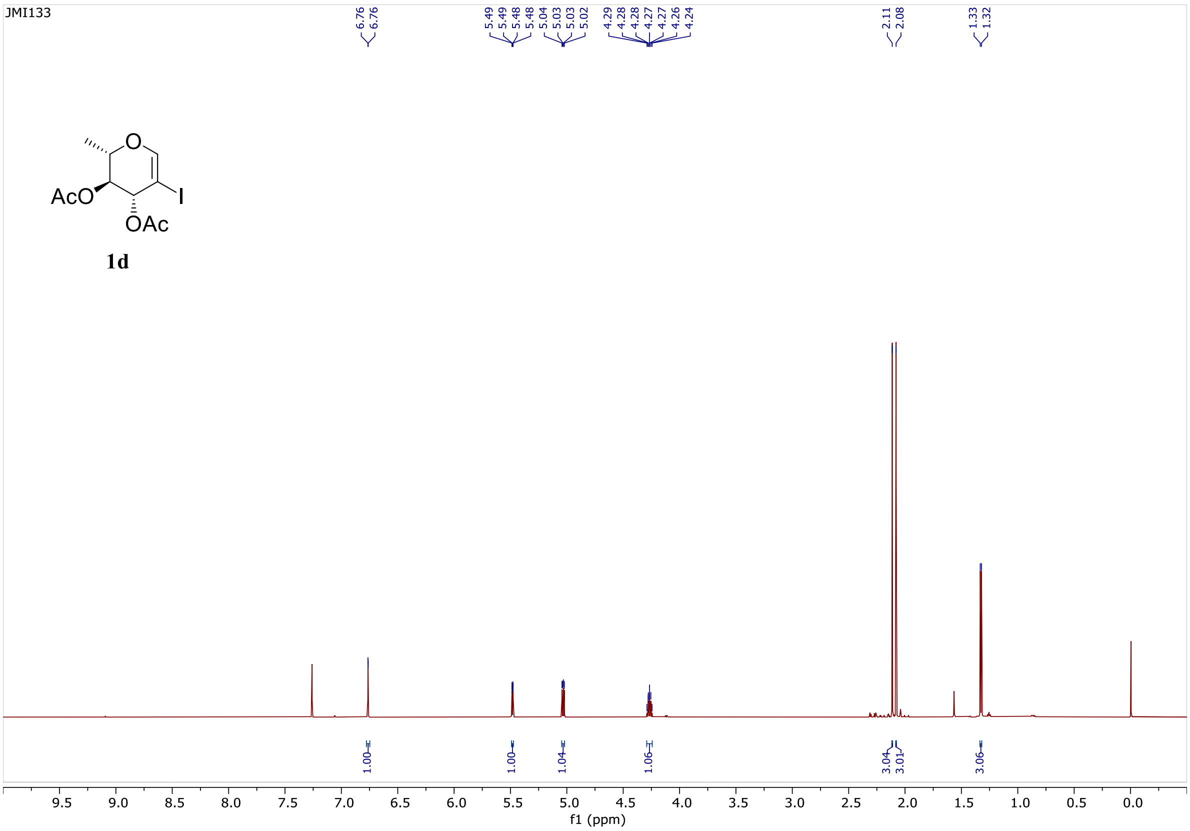

^{1}H NMR (600MHz, $CDCl_3$) of 2a

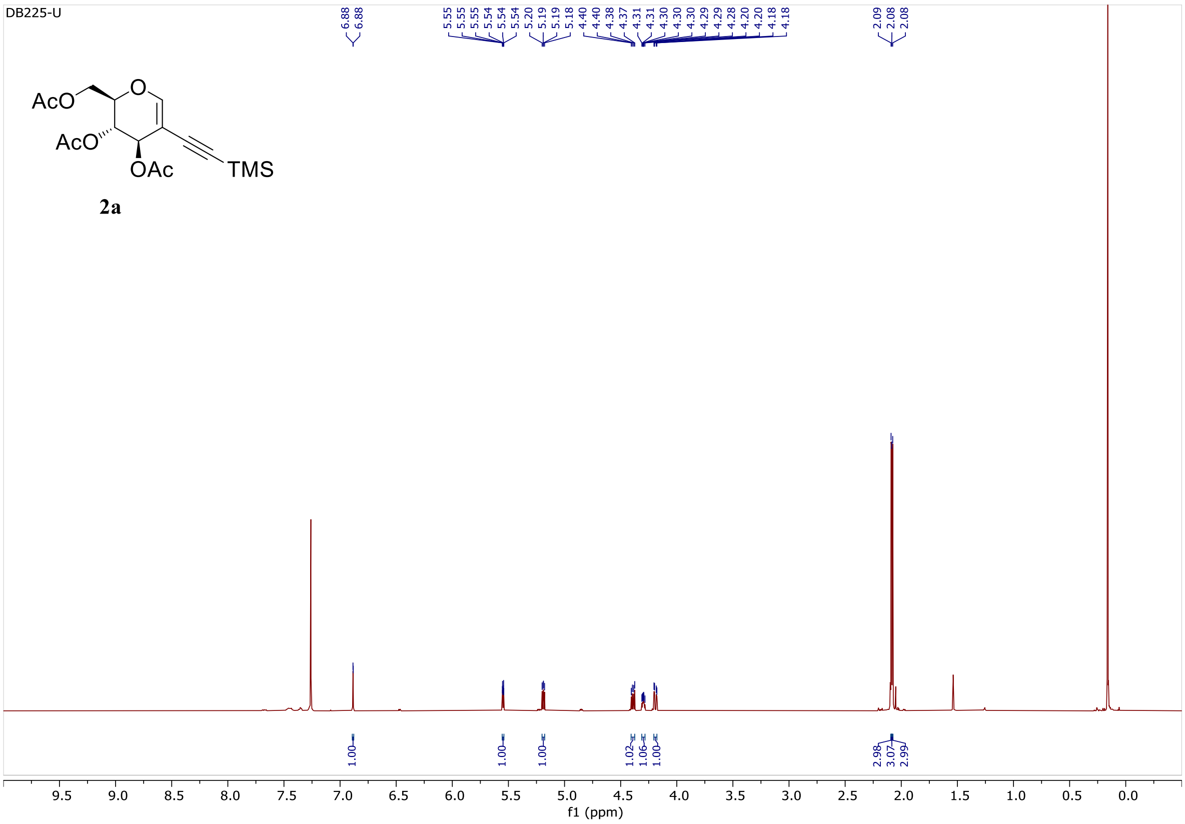

^{1}H NMR (600MHz, $CDCl_3$) of 2b

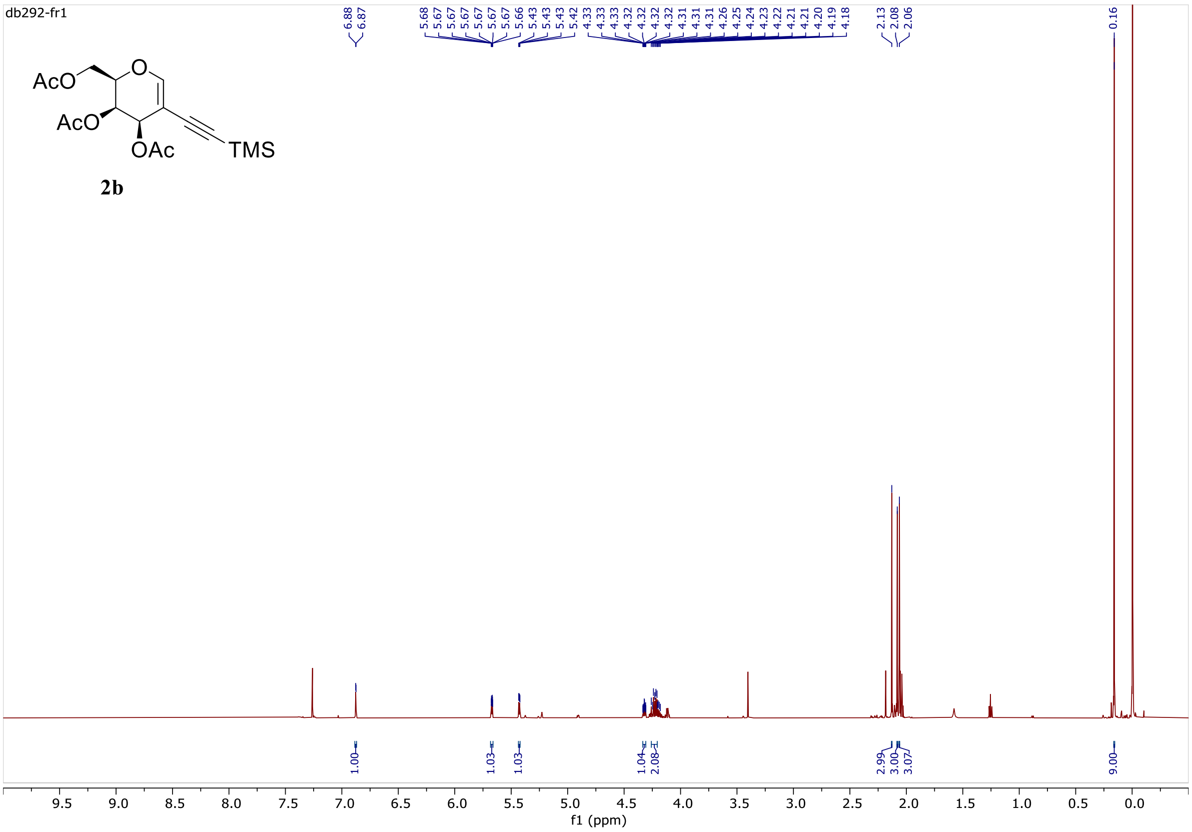

^{1}H NMR (600 MHz, $CDCl_3$) of 3

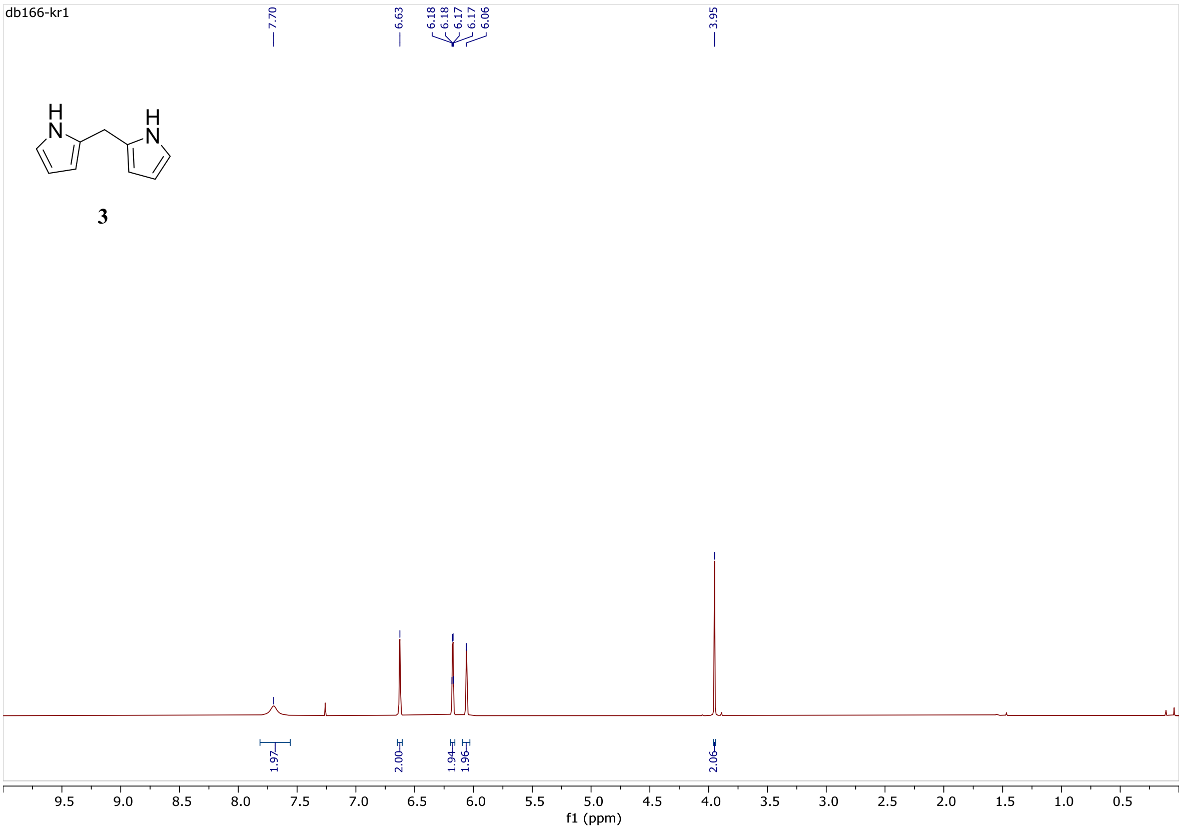

^{13}C NMR (151 MHz, $CDCl_3$) of 3

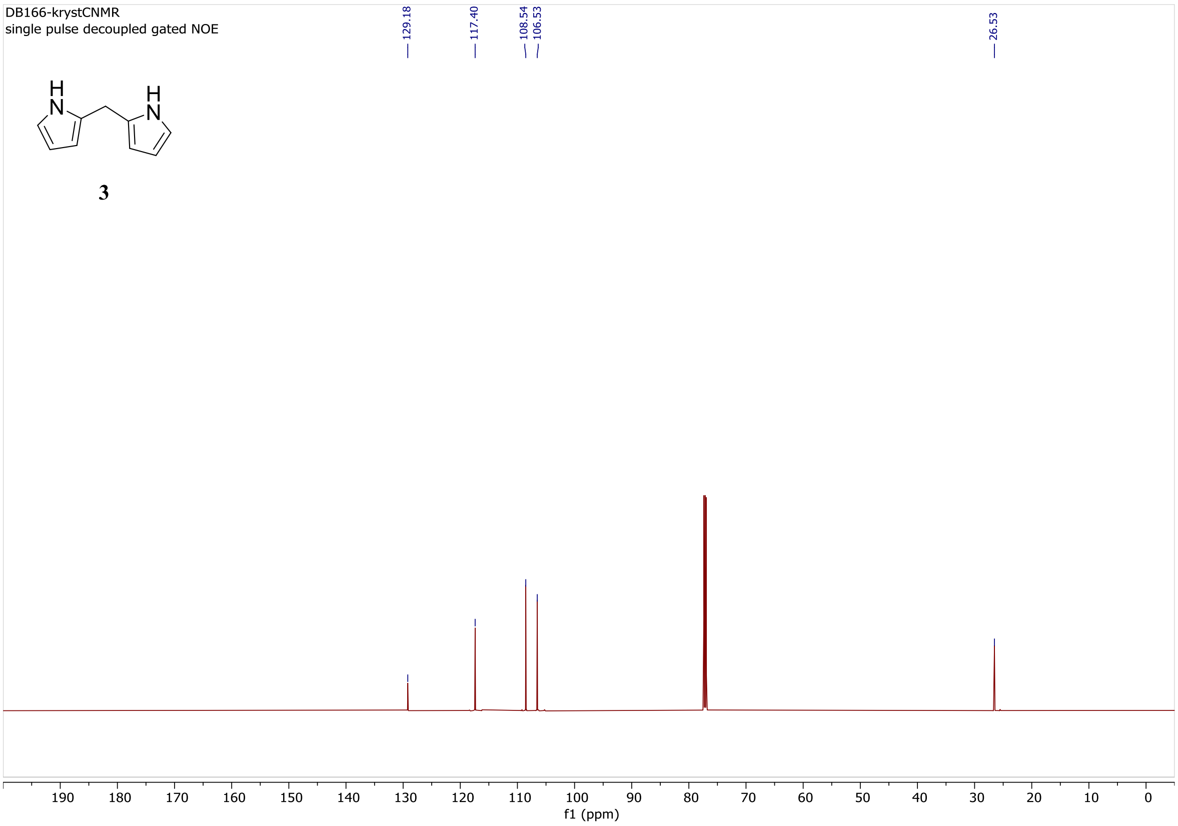

^{1}H NMR (600 MHz, $CDCl_3$) of 4a

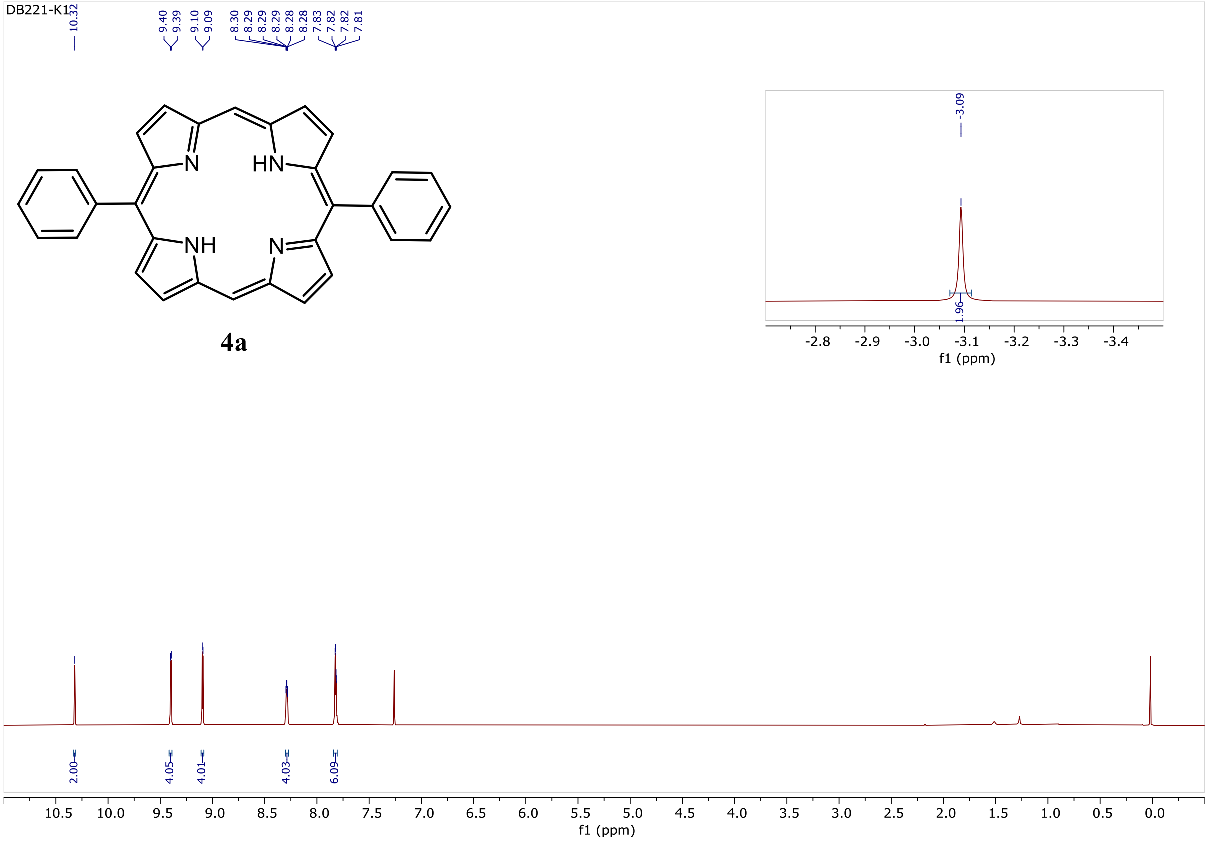

^{13}C NMR (151 MHz, $CDCl_3$) of 4a

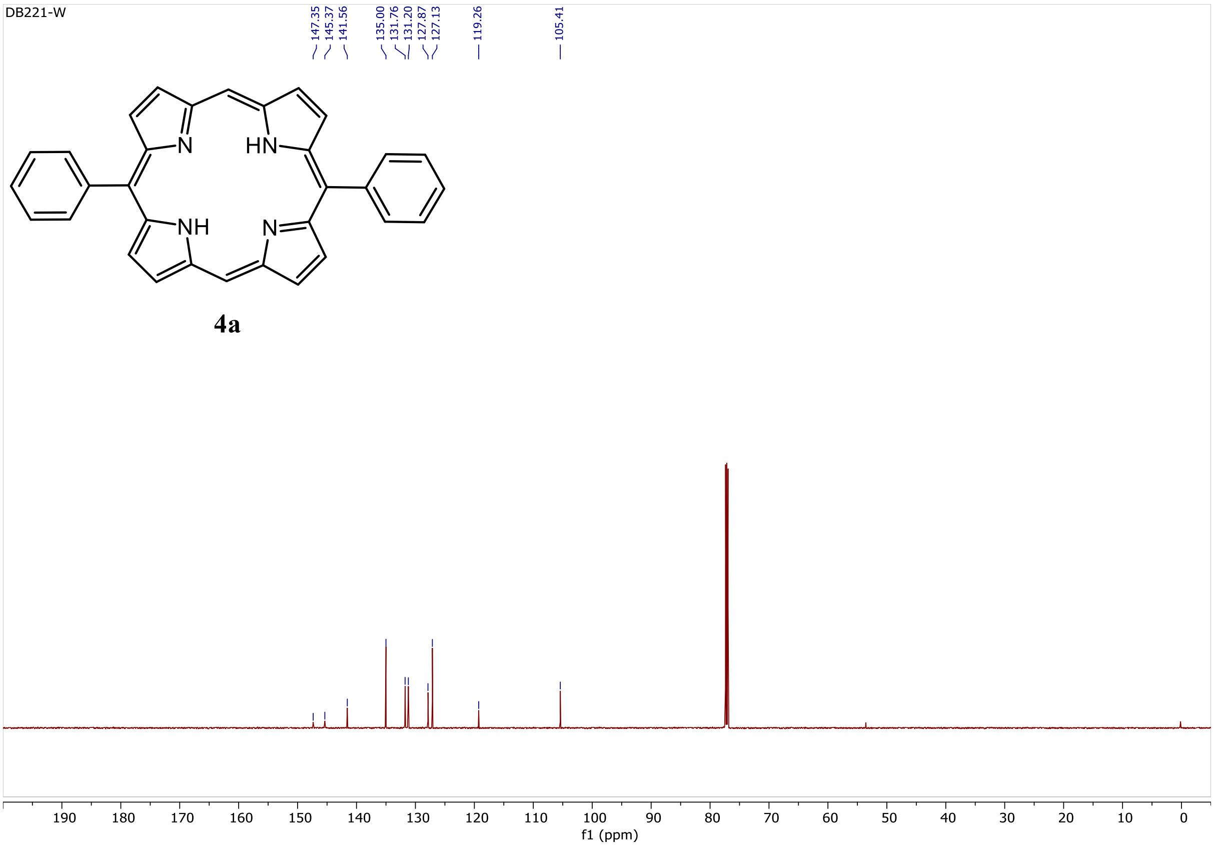

^1H NMR (600MHz, $CDCl_3$) of 4c

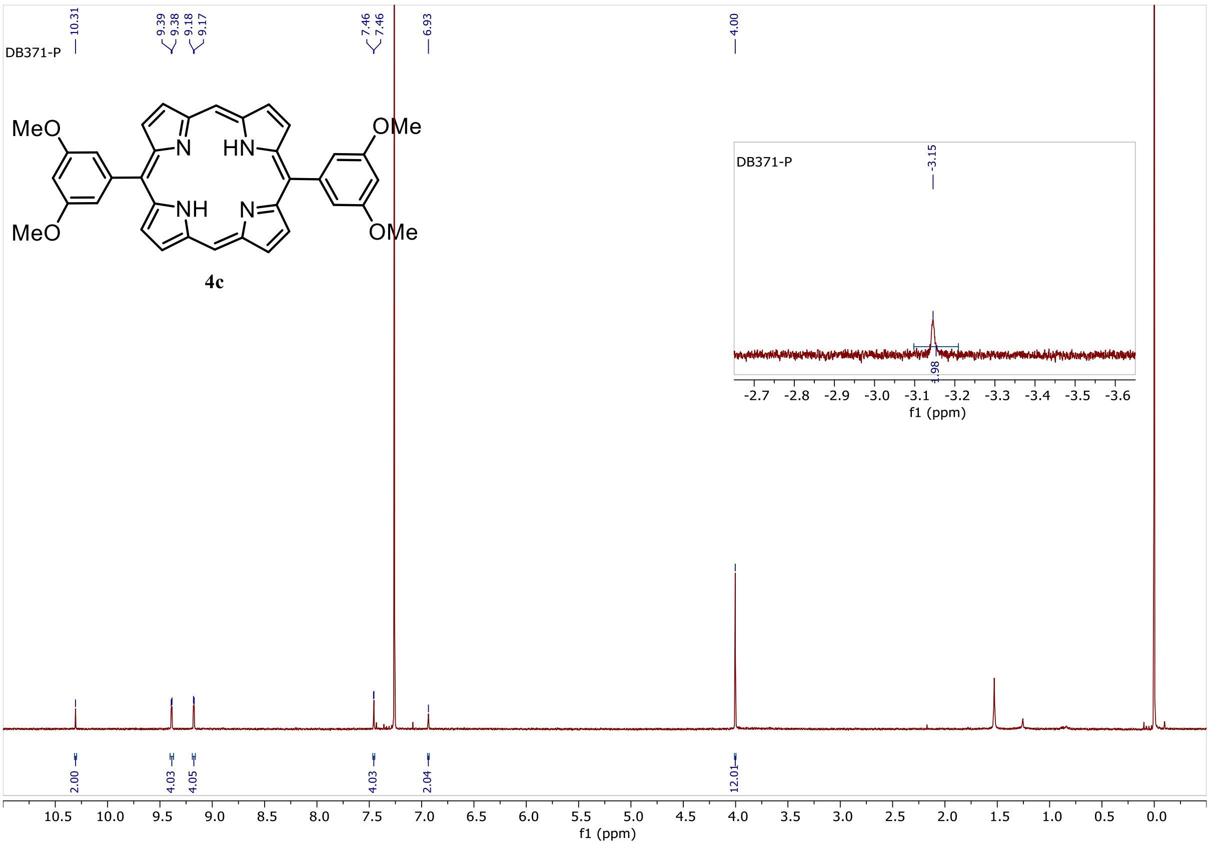

^1H NMR (600MHz, $CDCl_3$) of 4f

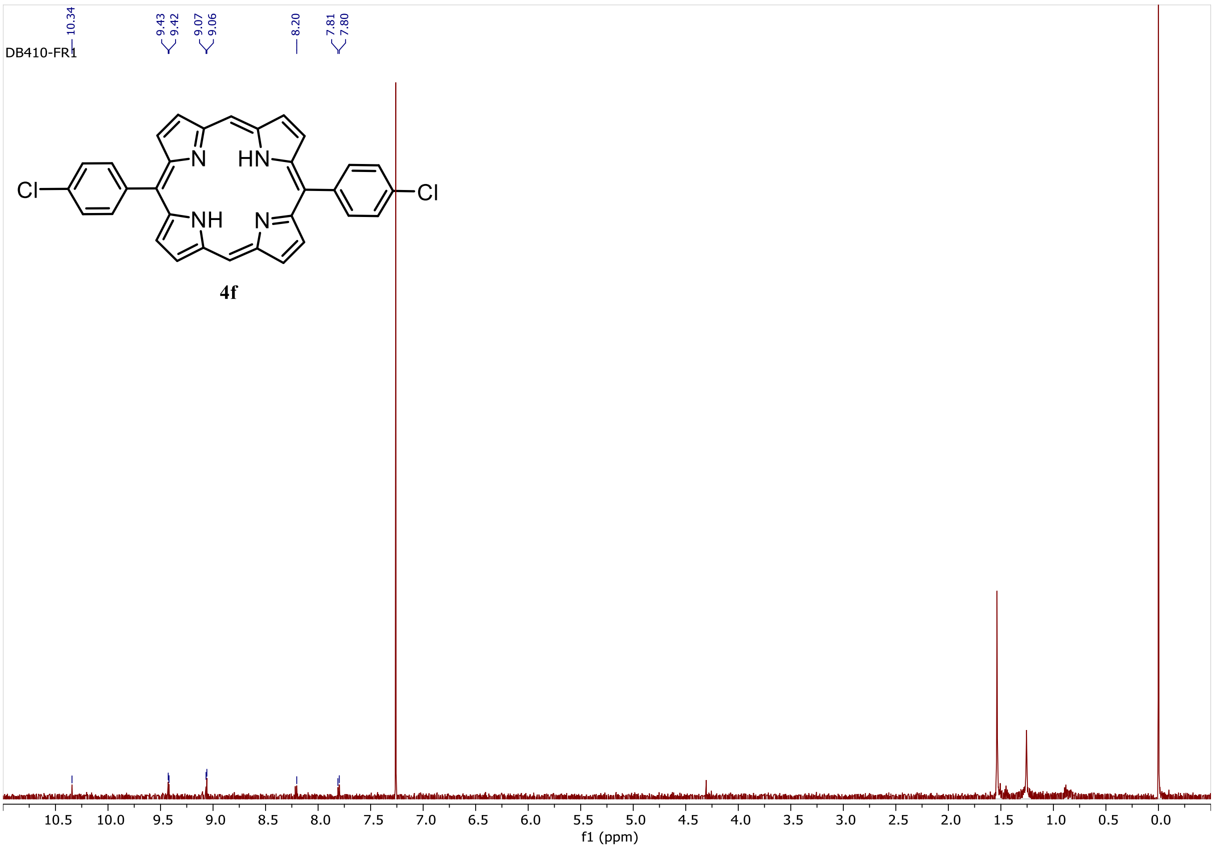

^{1}H NMR (600MHz, $CDCl_3$) of 4g

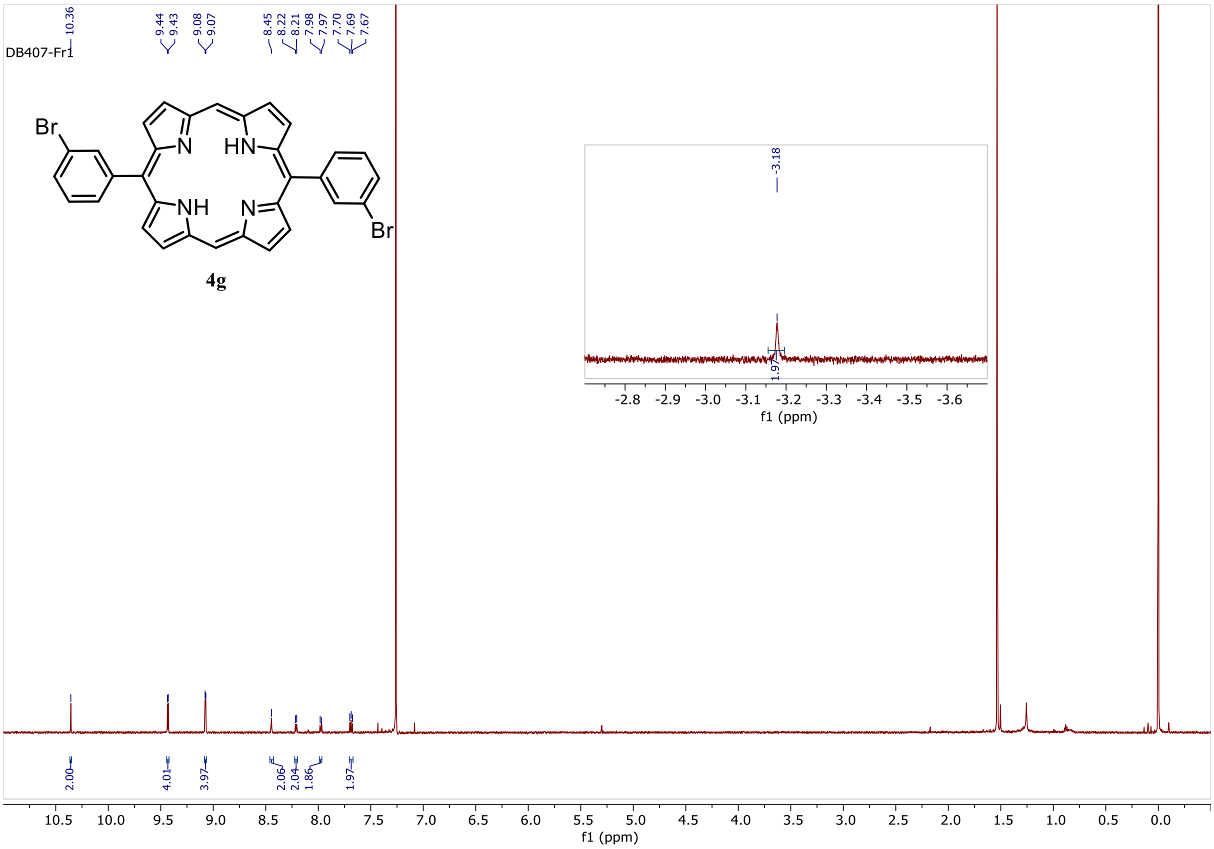

^{1}H NMR (600MHz, $CDCl_3$) of 5a

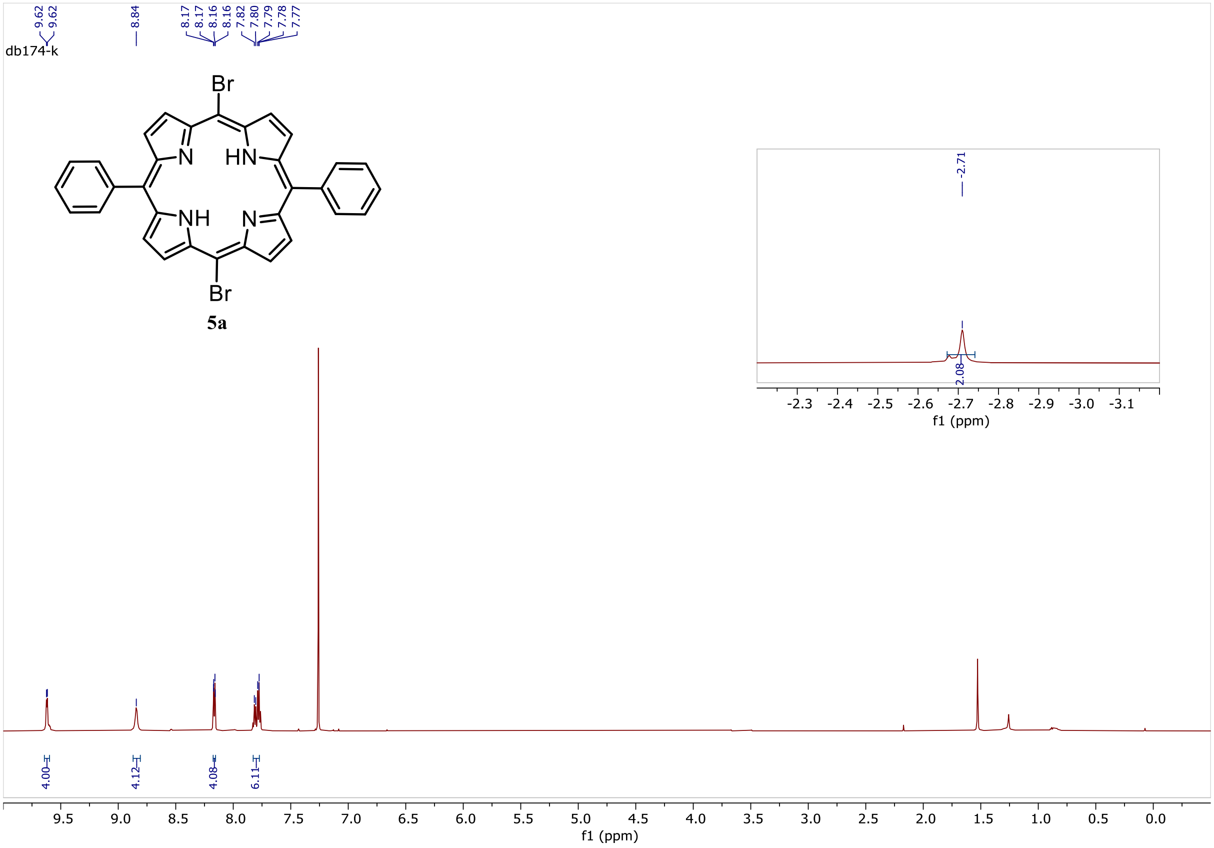

^{1}H NMR (600 MHz, pyridine-d_5) of 5d (for saturated solution at 100 °C)

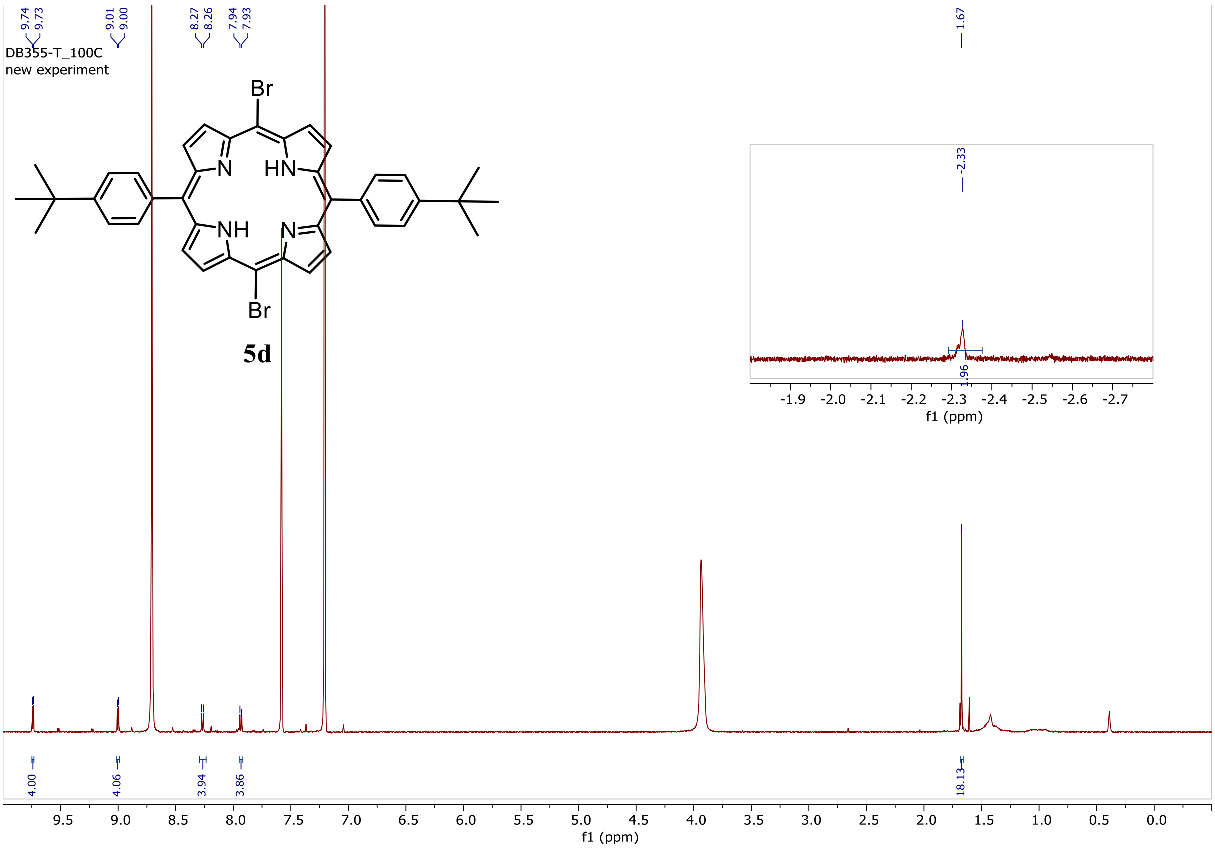

^{1}H NMR (600MHz, $CDCl_3$) of TPP

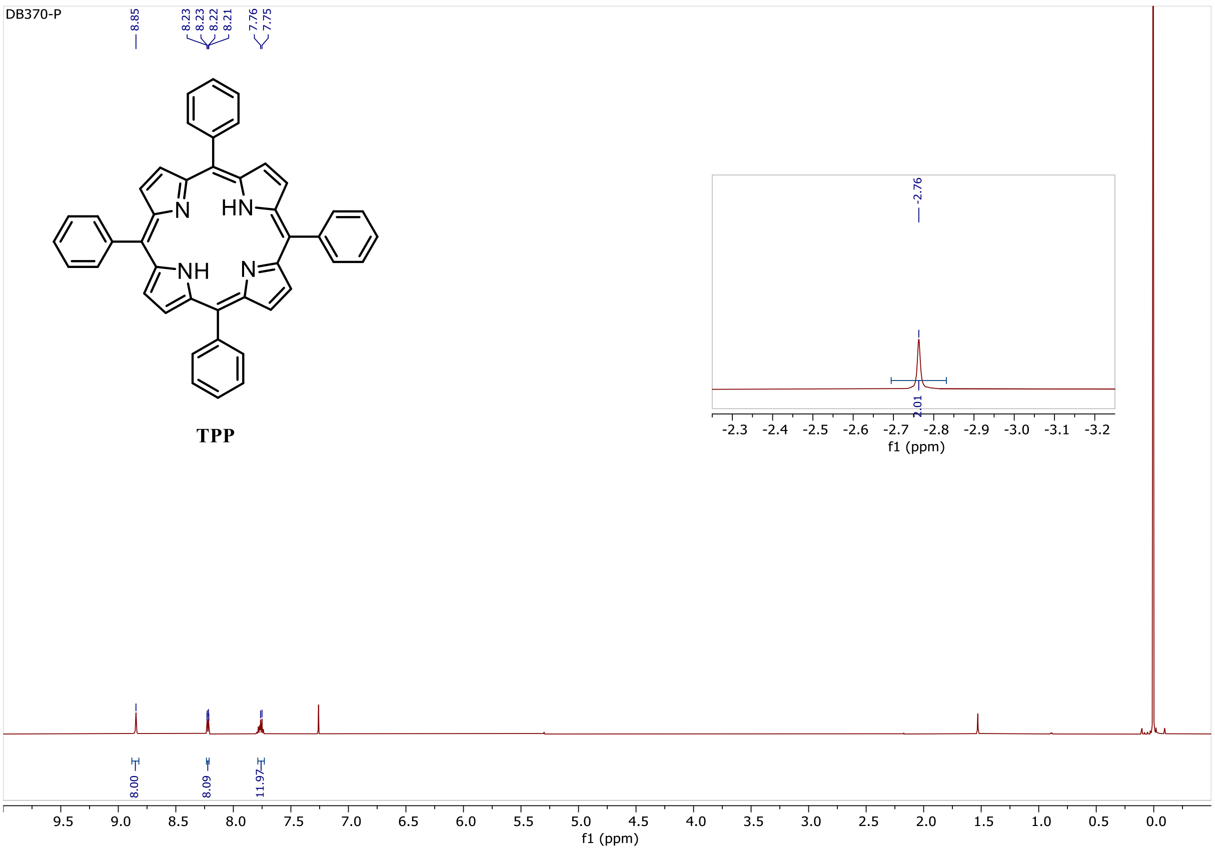

^{1}H NMR (600MHz, $CDCl_3$) of EtDPP

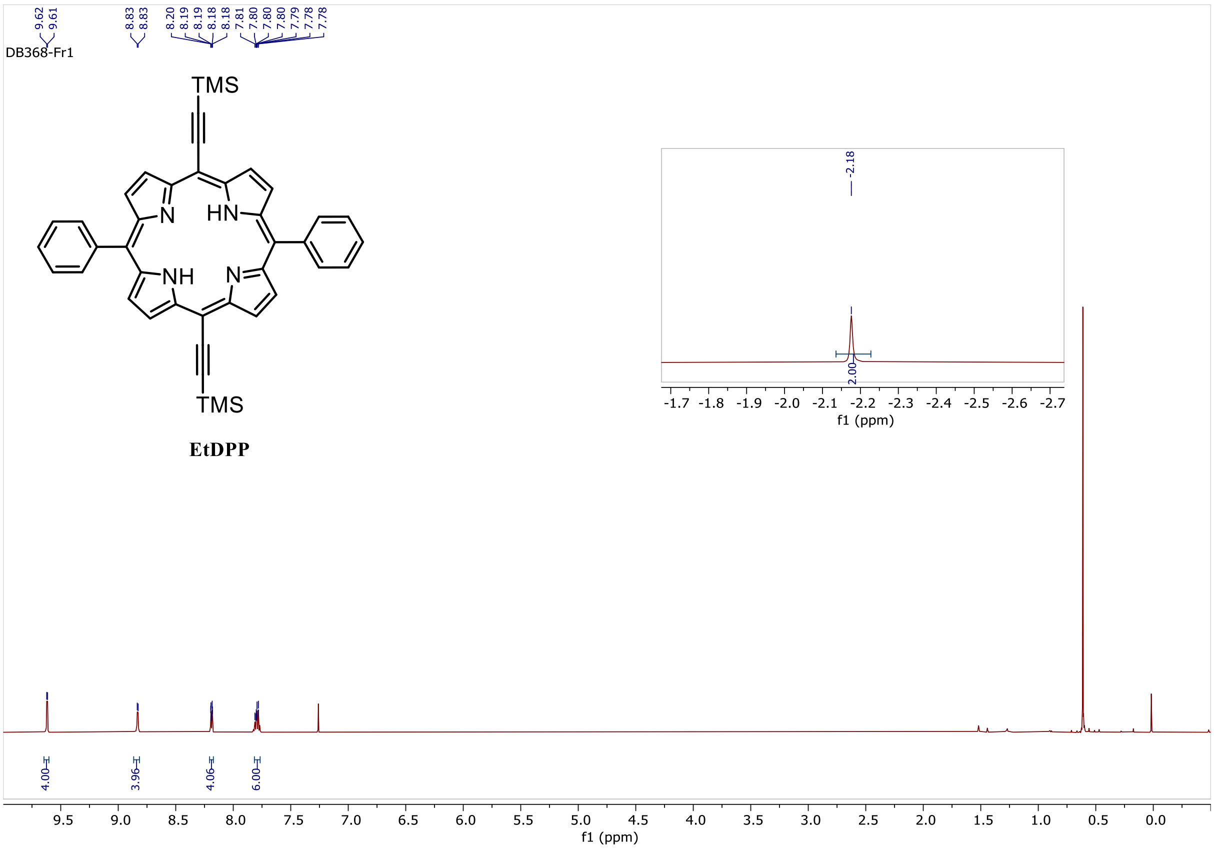

8. NMR spectra of new compounds

^{1}H NMR (600 MHz, $CDCl_3$) of 2c

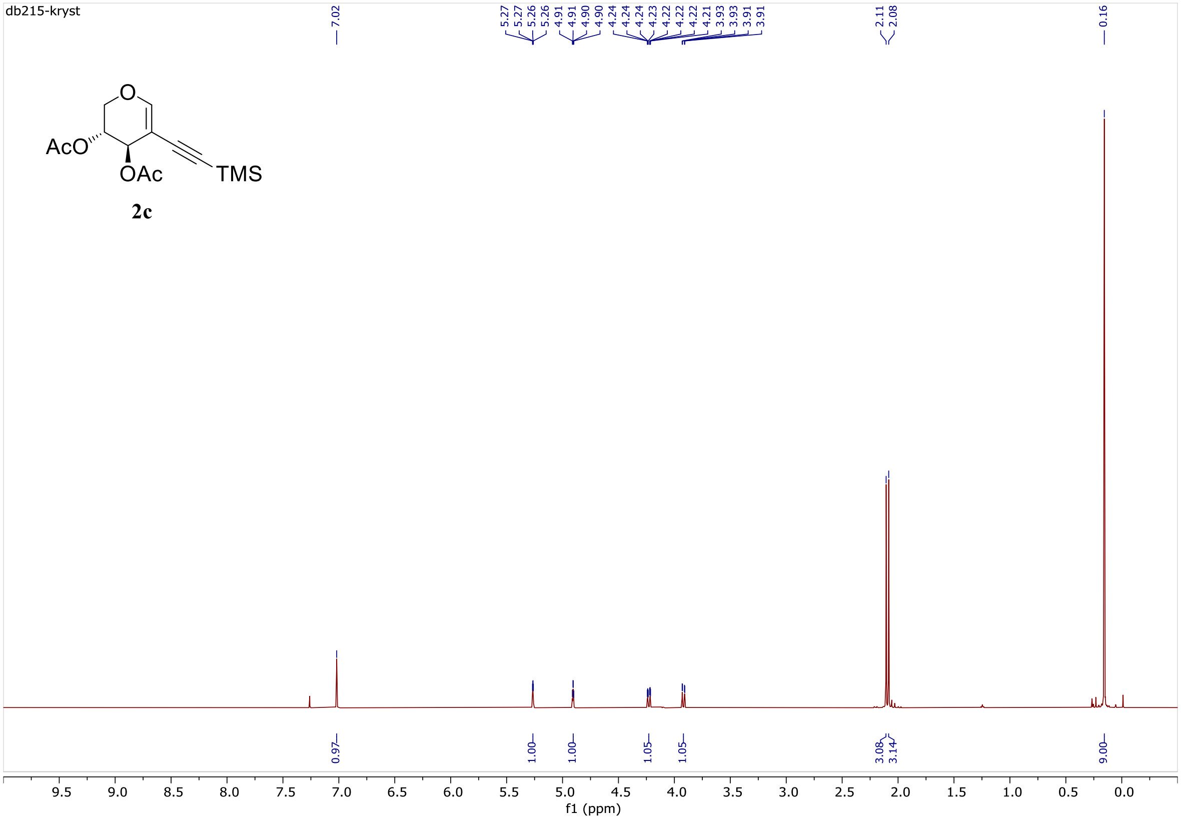

^{13}C NMR (151 MHz, $CDCl_3$) of 2c

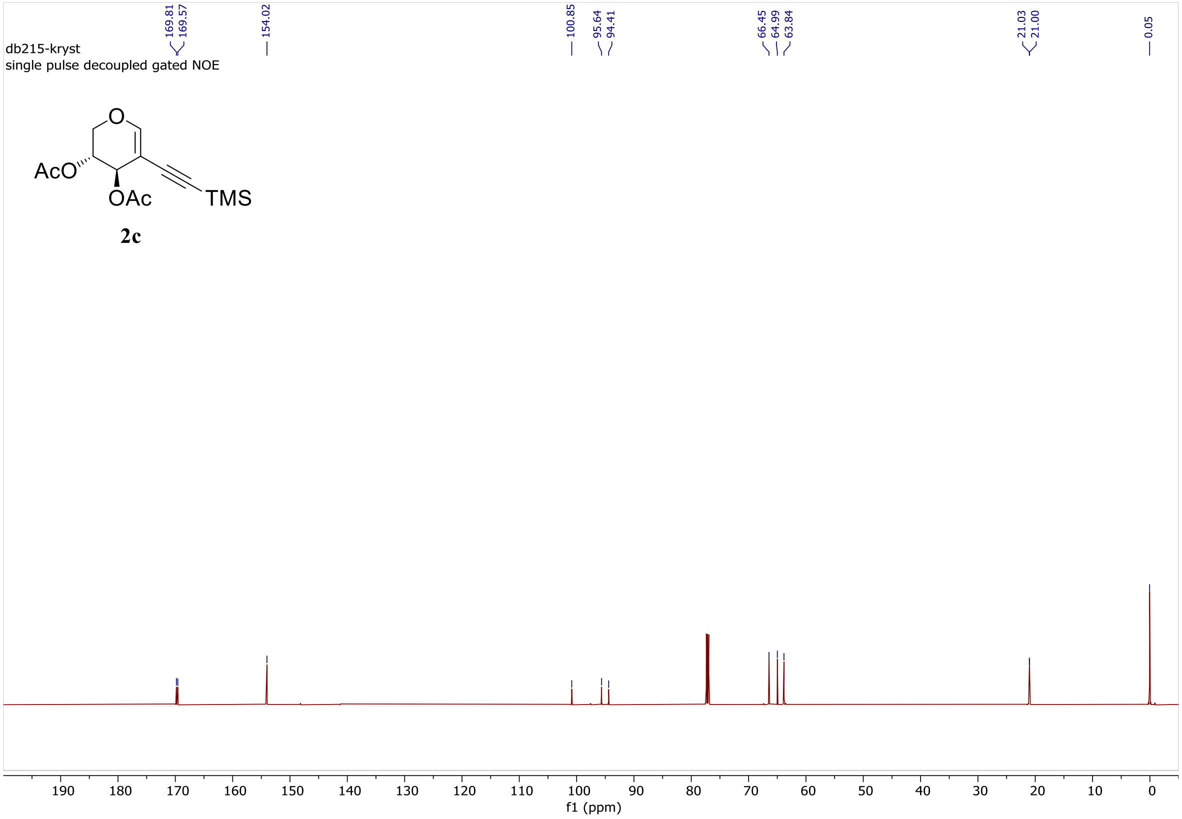

^{1}H NMR (500 MHz, $CDCl_3$) of 2d

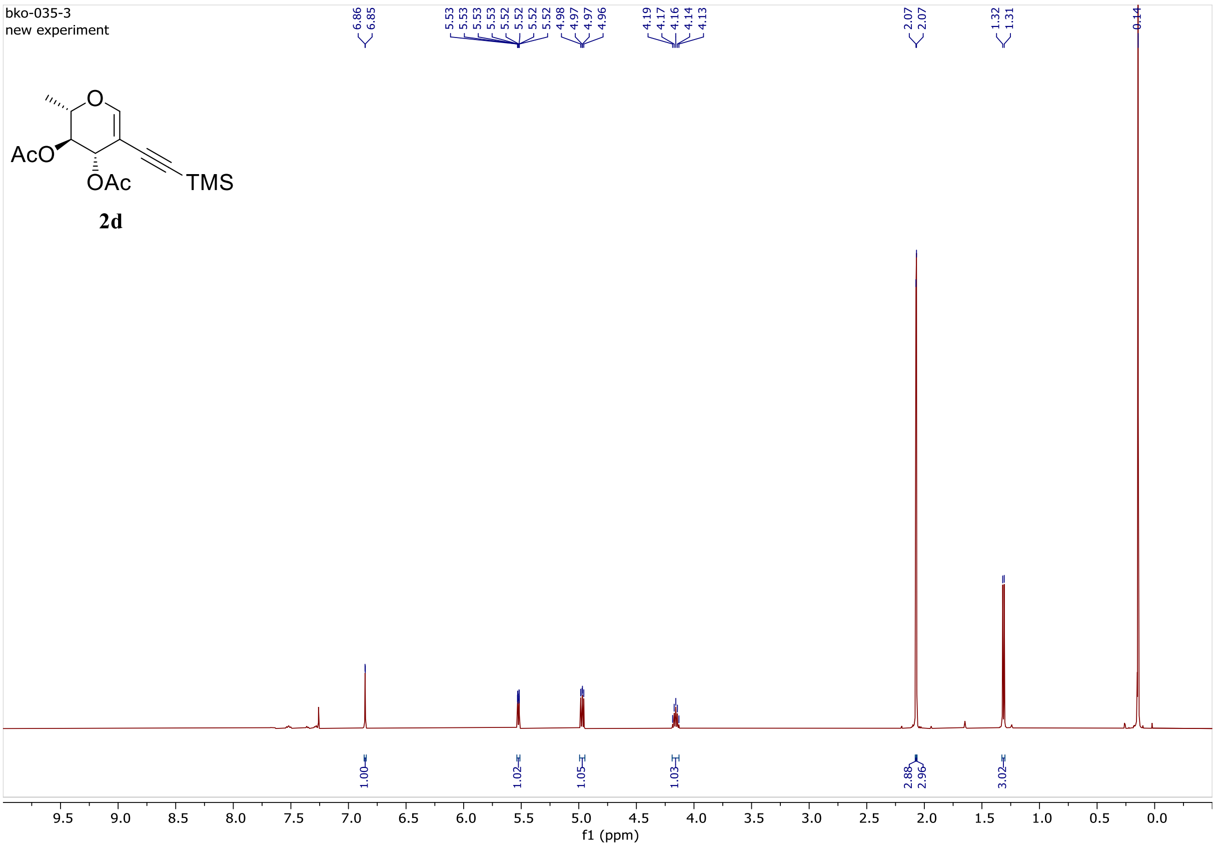

^{13}C NMR (126 MHz, $CDCl_3$) of 2d

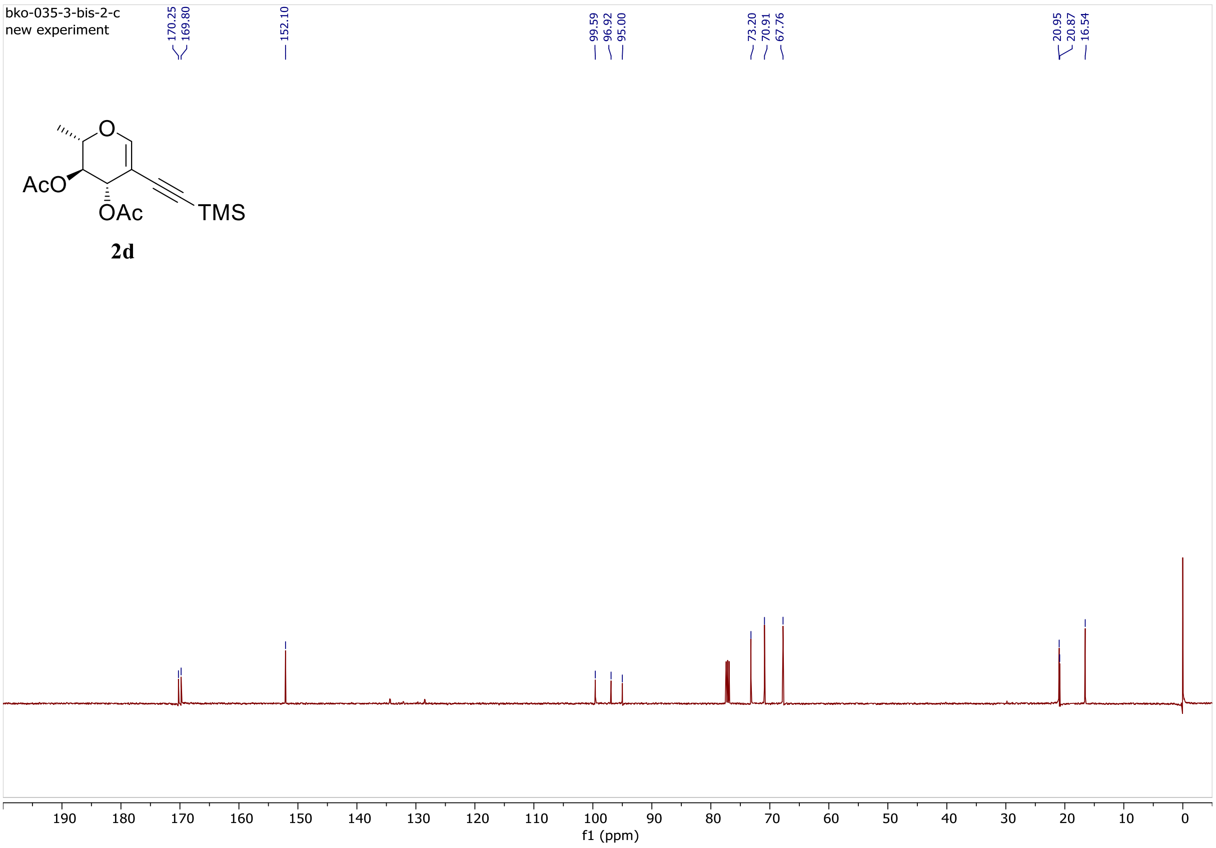

^{1}H NMR (600 MHz, $CDCl_3$) of 4b

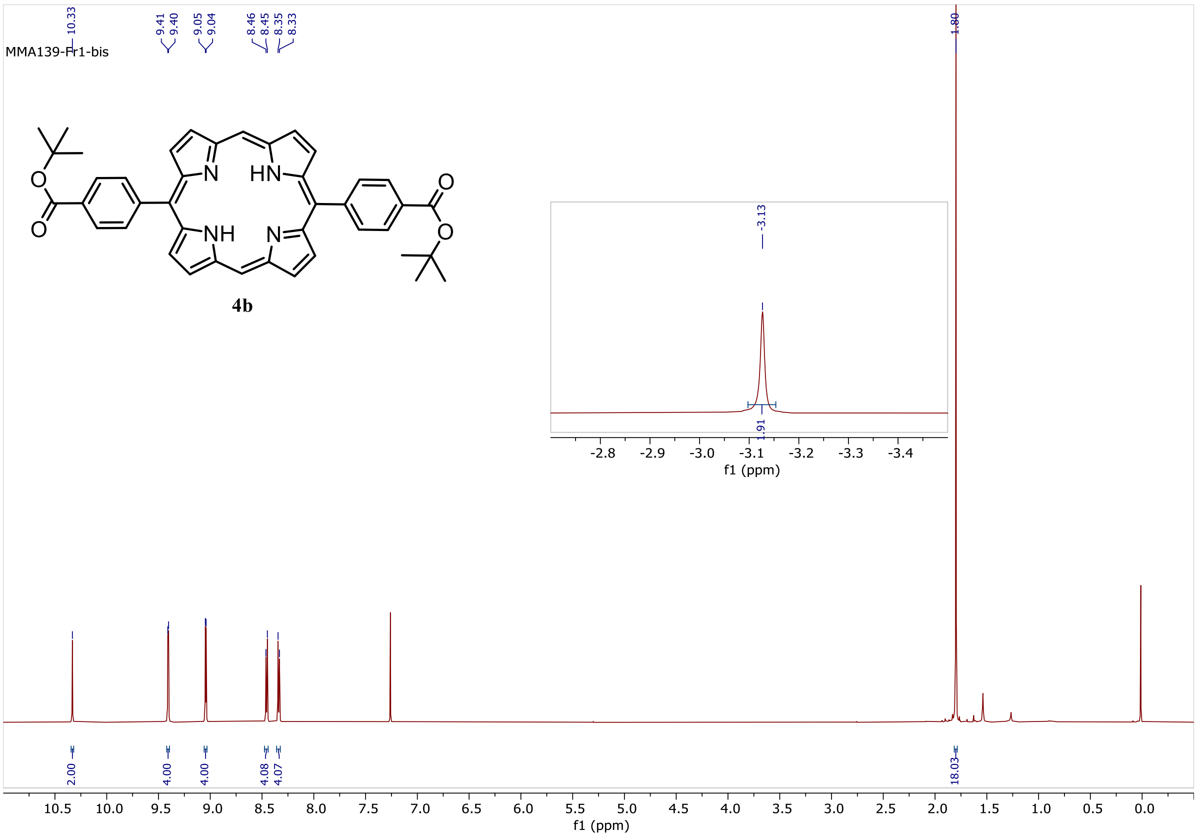

^{13}C NMR (151 MHz, $CDCl_3$) of 4b

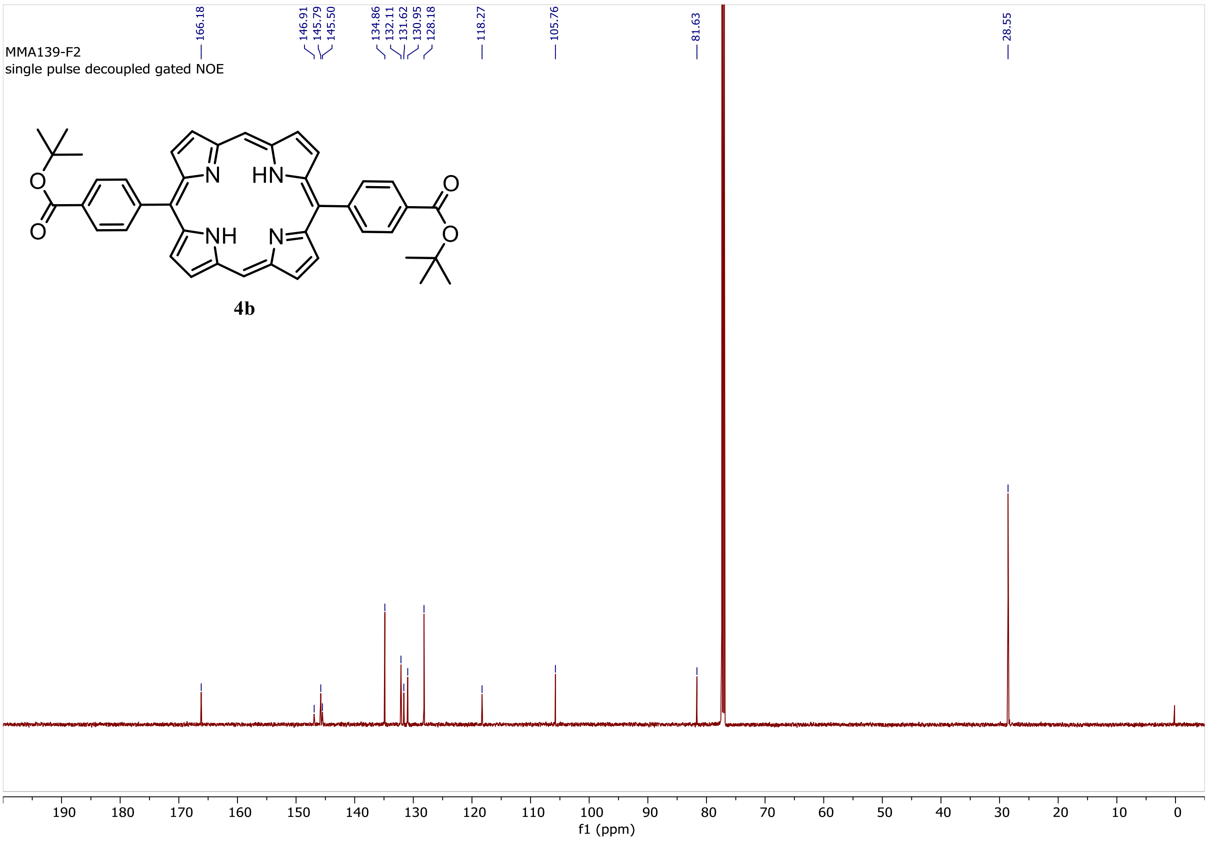

^{1}H NMR (600 MHz, $CDCl_3$) of 4e

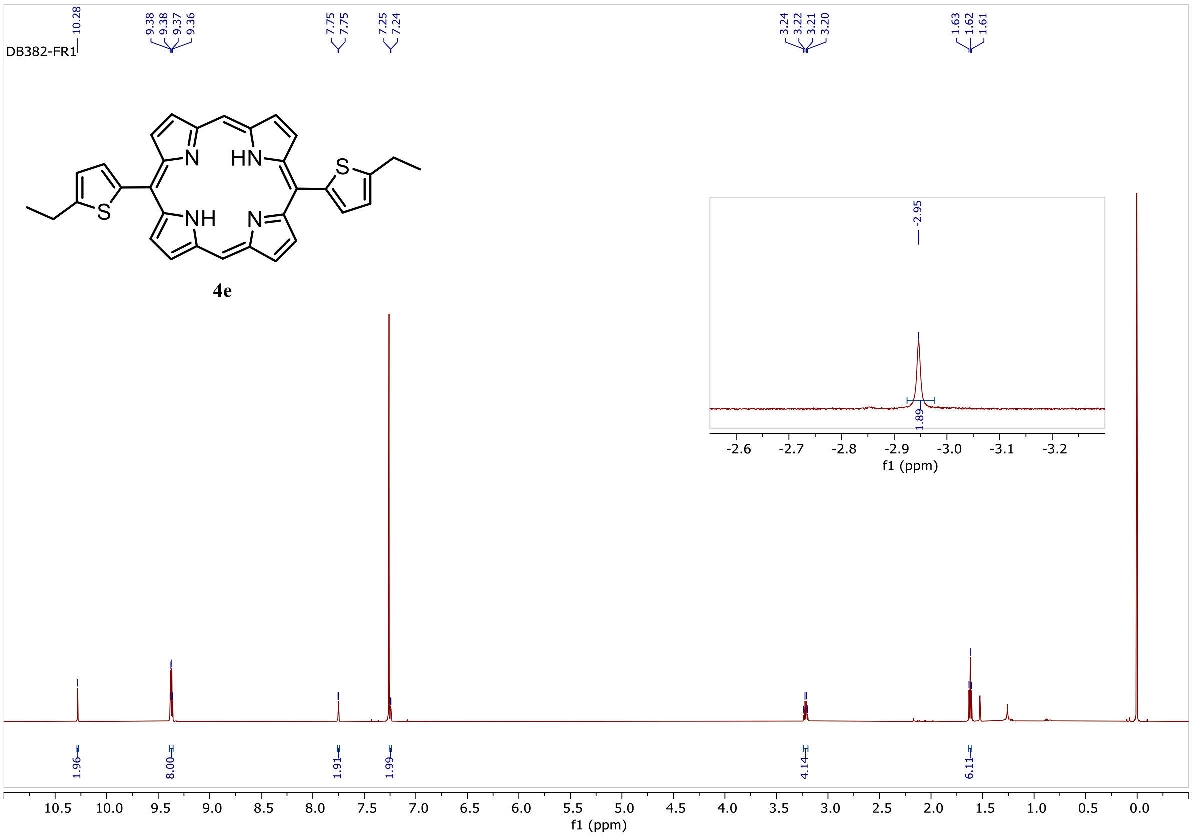

^{13}C NMR (151 MHz, $CDCl_3$) of 4e

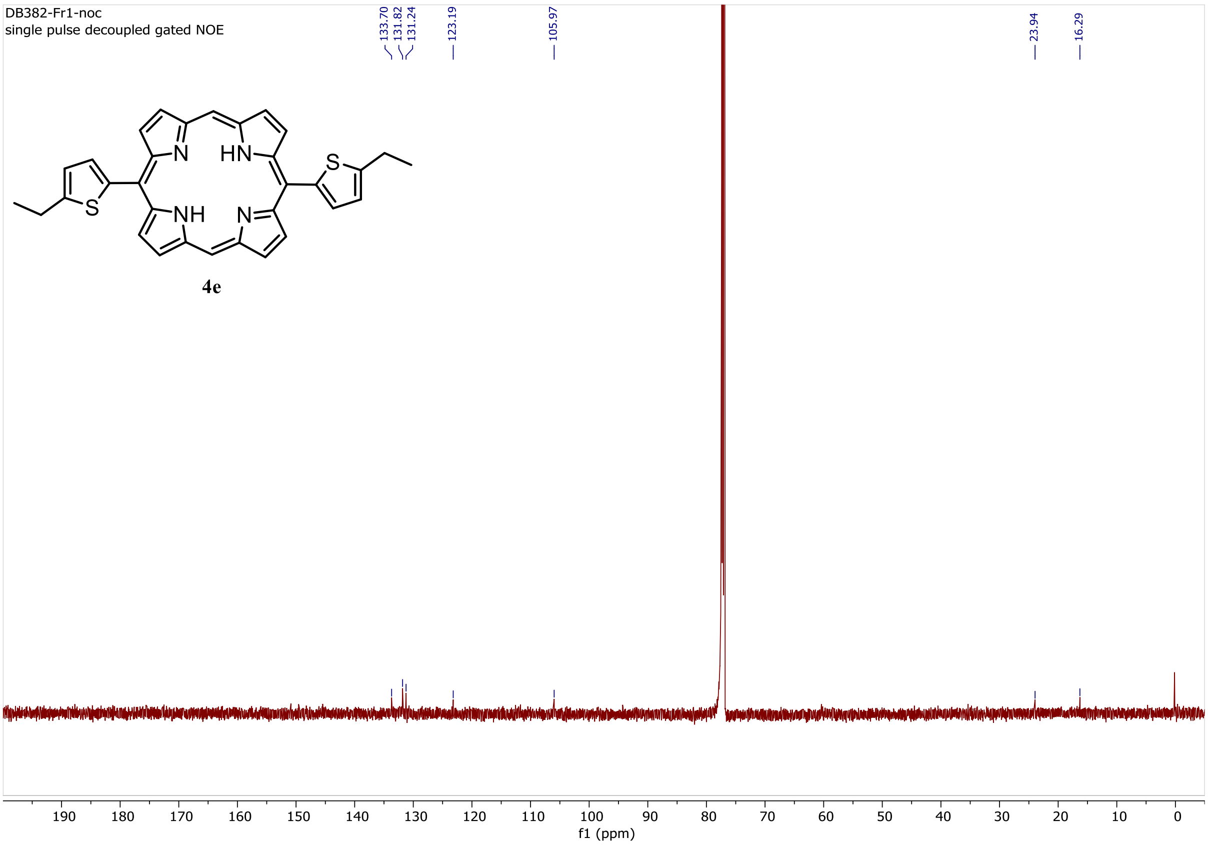

^{1}H NMR (500 MHz, $CDCl_3$) of 4h (for saturated solution)

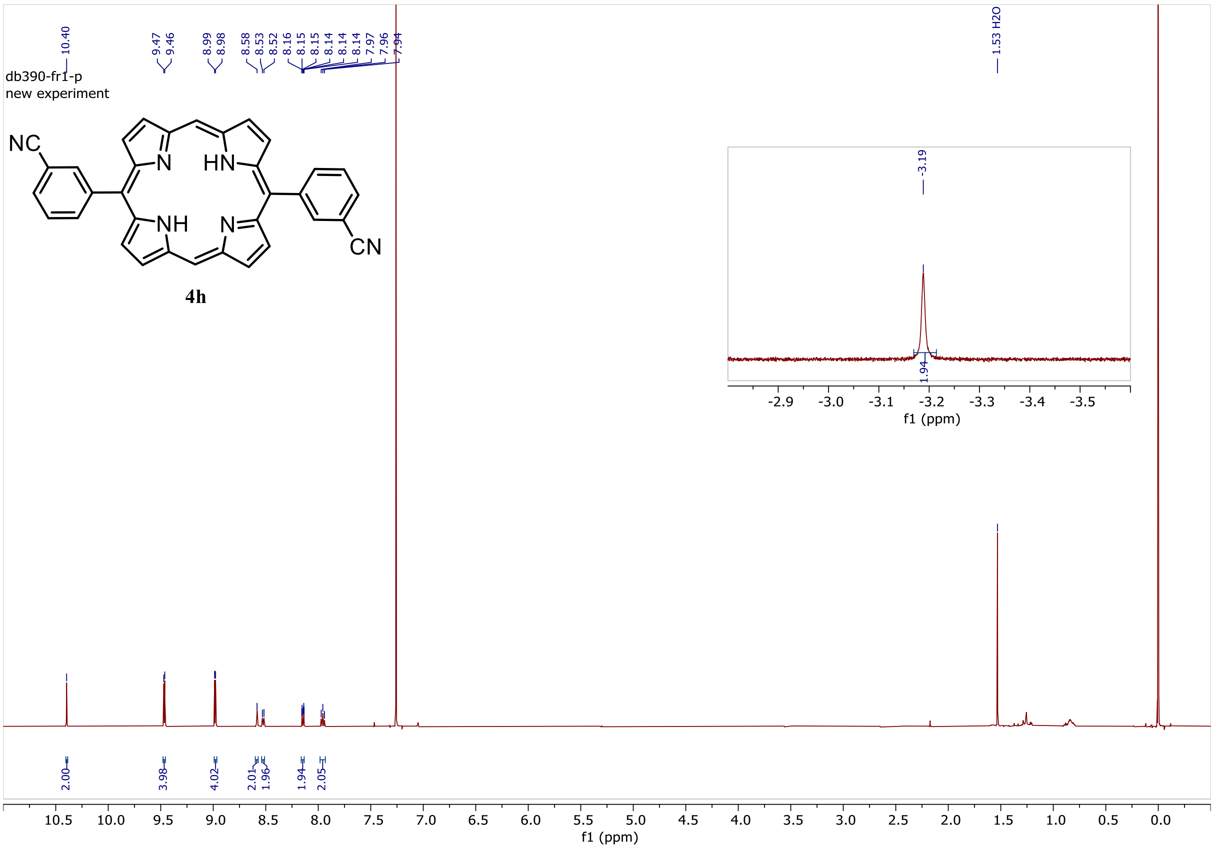

^{13}C NMR (151 MHz, $CDCl_3$) of 4h (for saturated solution)

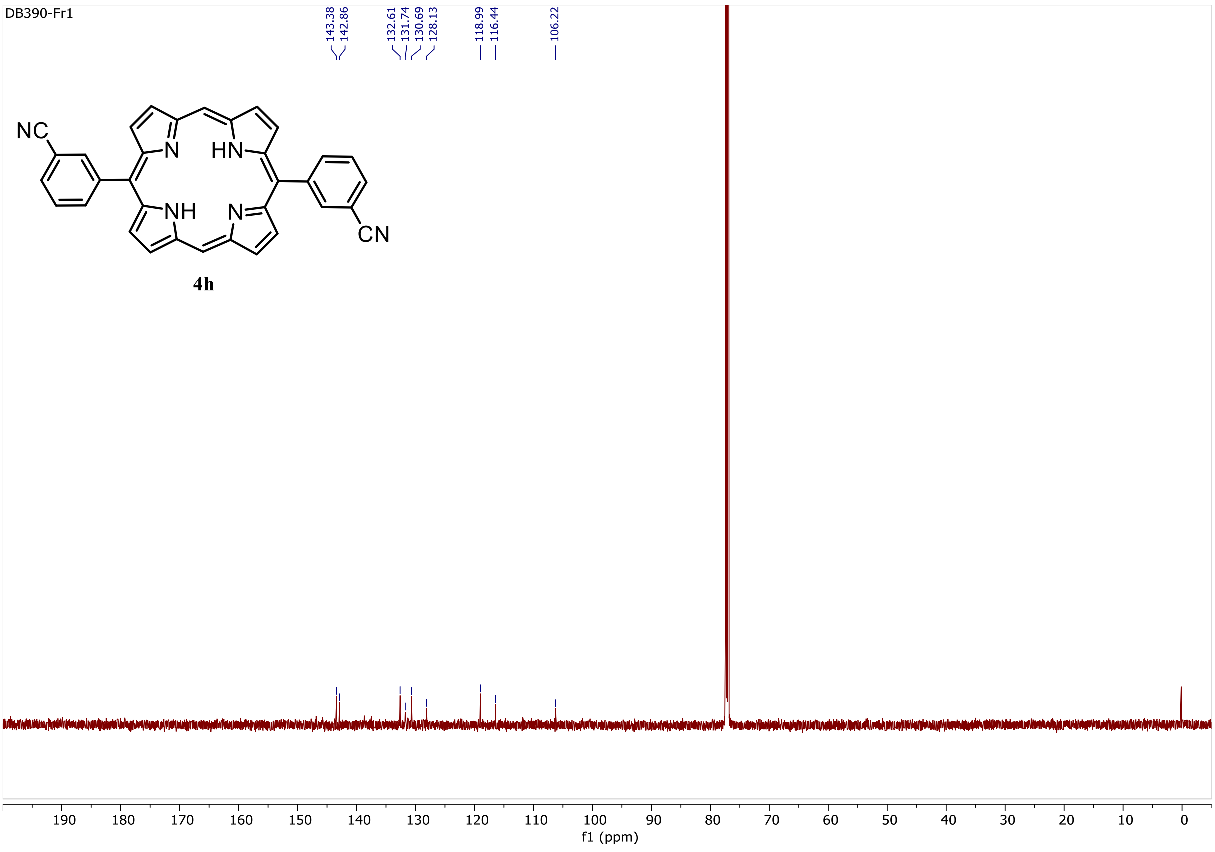

^1H NMR (600 MHz, $CDCl_3$) of 5b

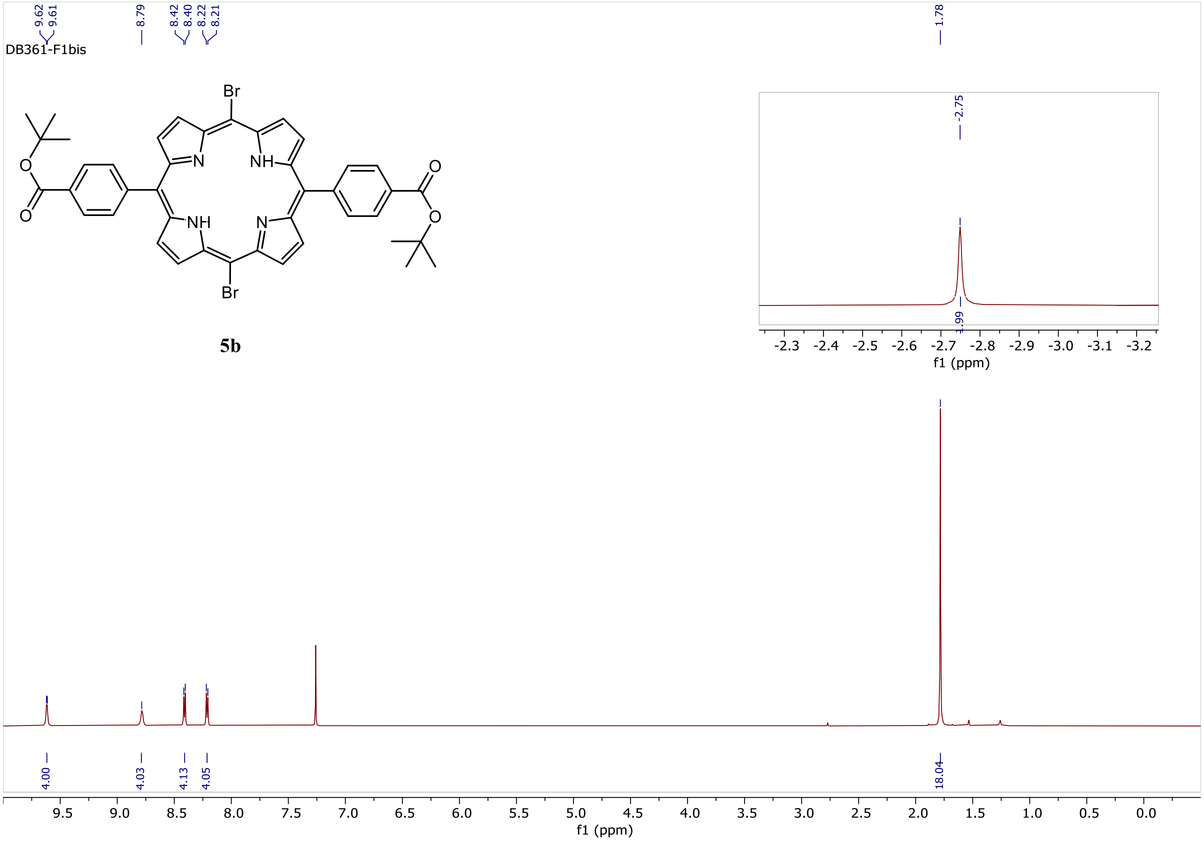

5b

^{13}C NMR (151 MHz, $CDCl_3$) of 5b

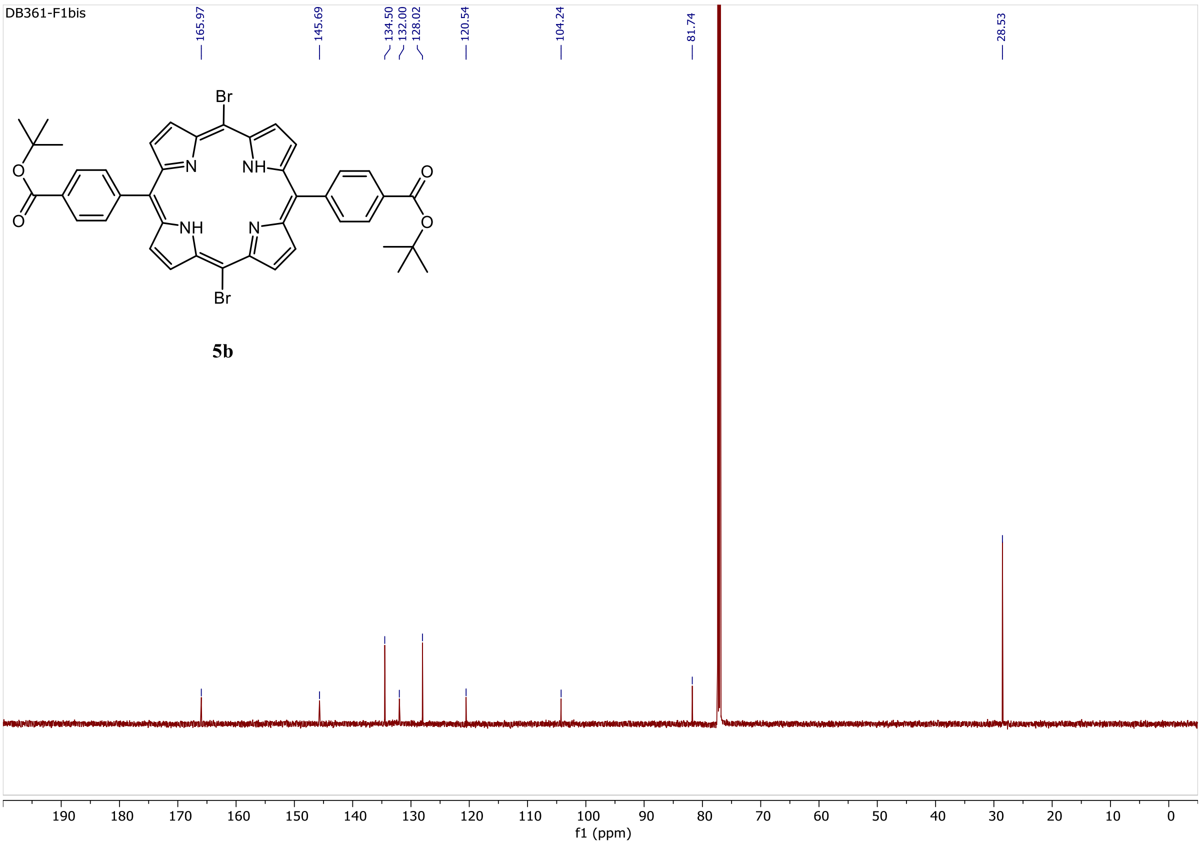

5b

^{1}H **NMR (600 MHz, $CDCl_3$) of 5c** (for saturated solution)

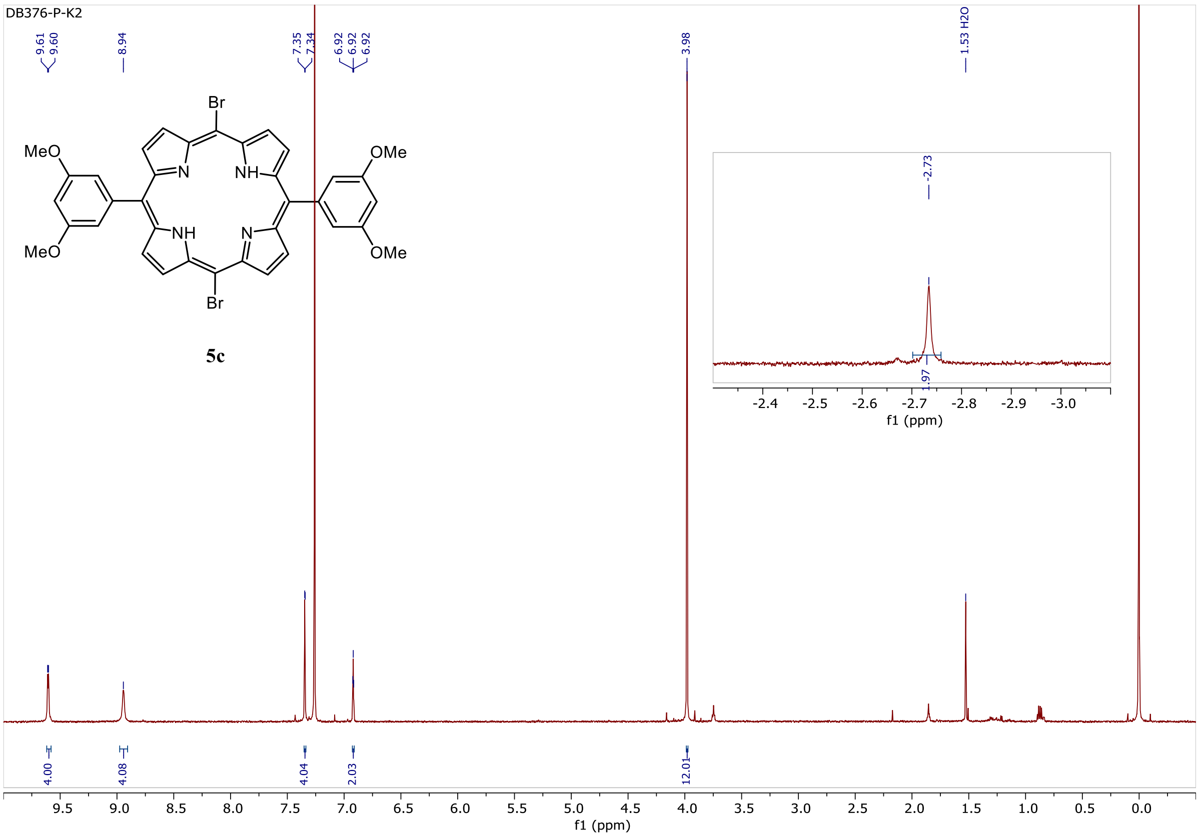

^{13}C **NMR (151 MHz, $CDCl_3$) of 5c** (for saturated solution)

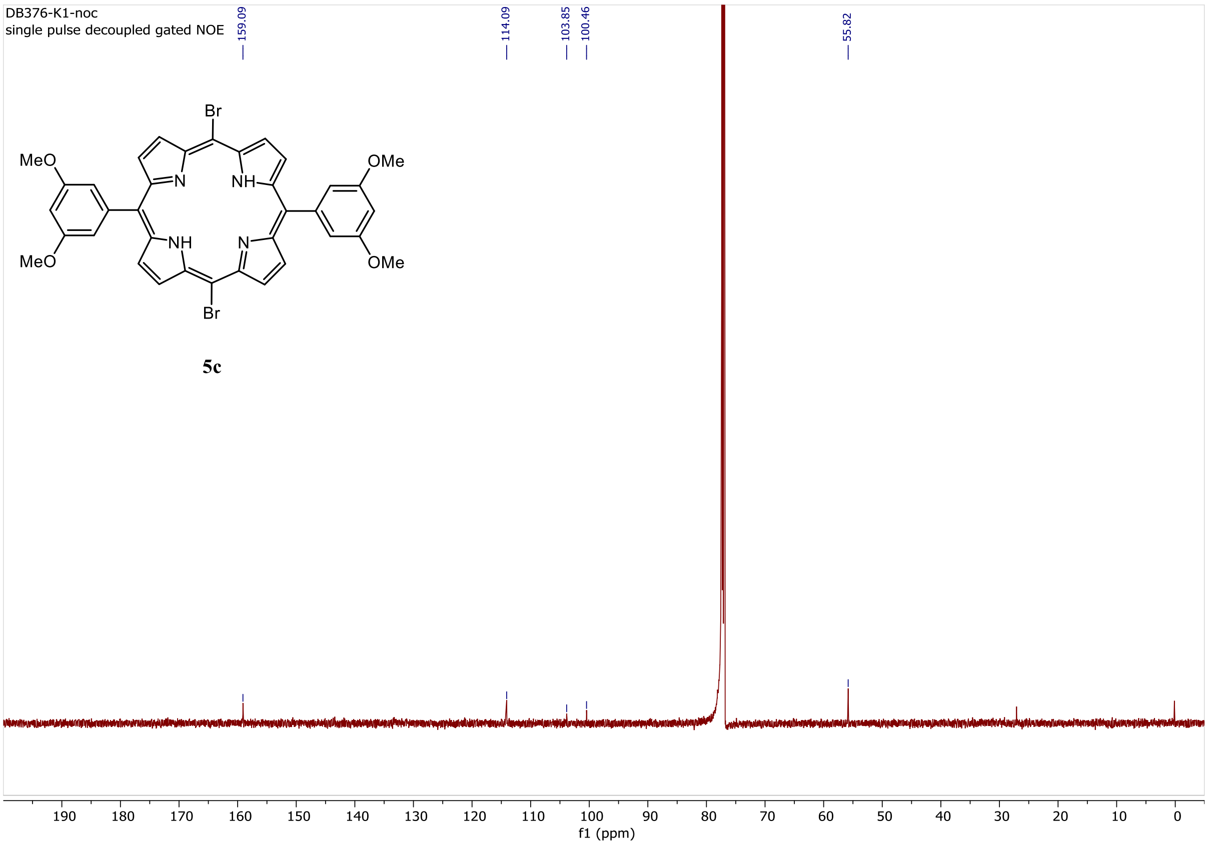

^1H NMR (600 MHz, $CDCl_3$) of 5e

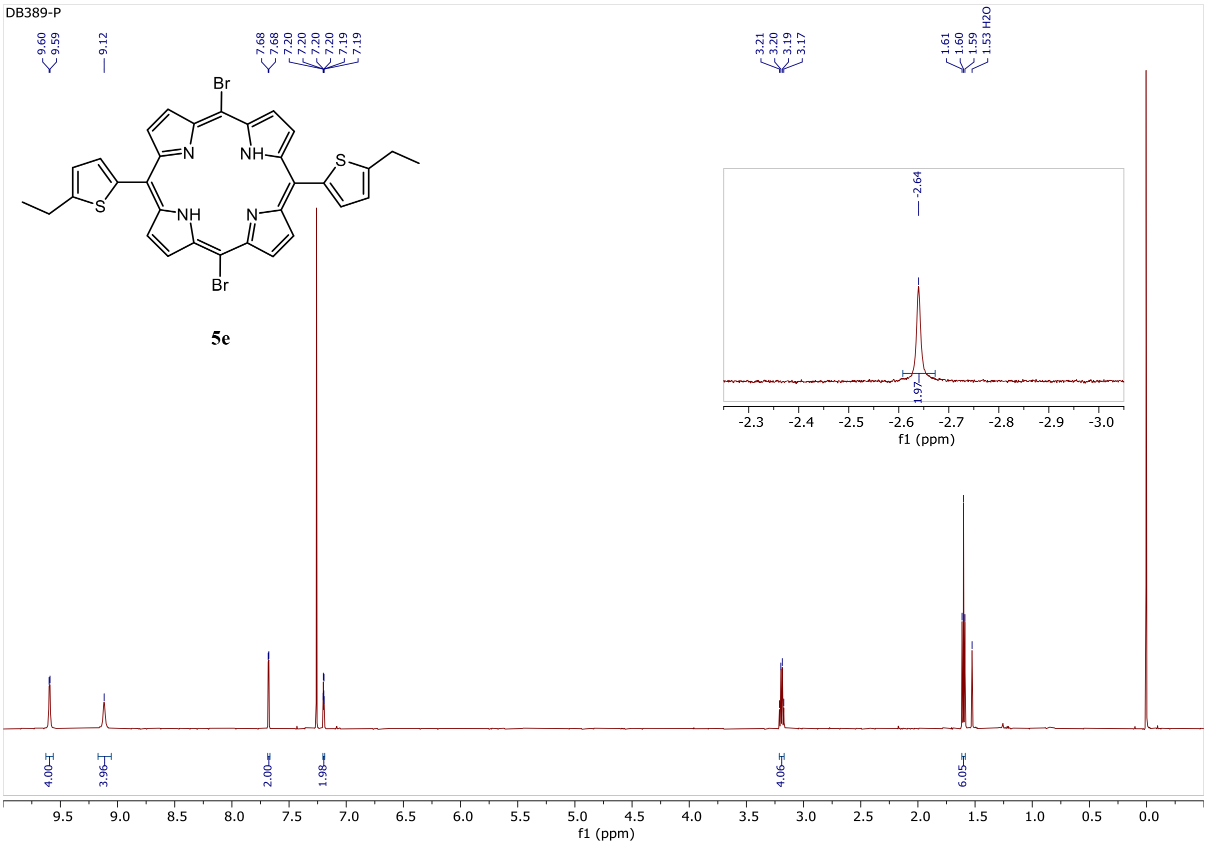

^{13}C NMR (151 MHz, $CDCl_3$) of 5e

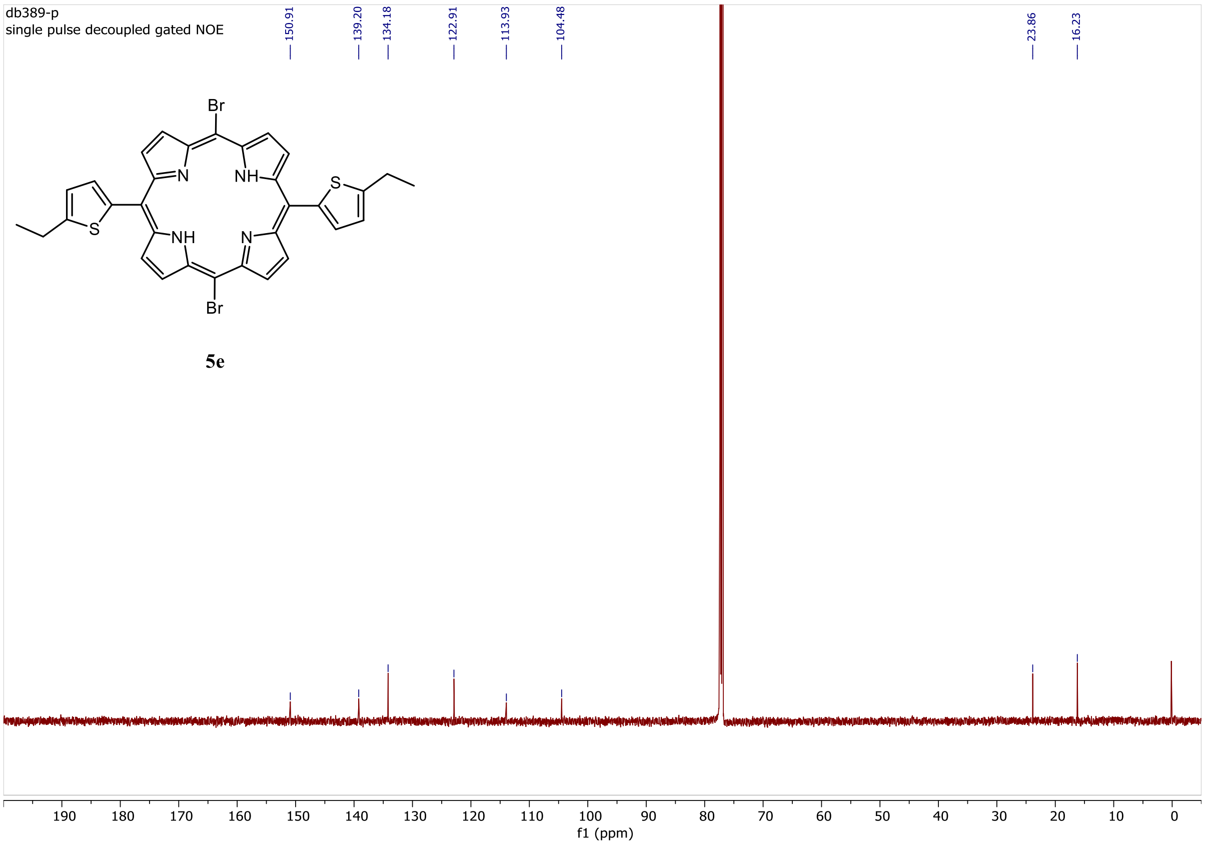

^{1}H NMR (600 MHz, pyridine-d$_5$) of 5f (for saturated solution at 60 °C)

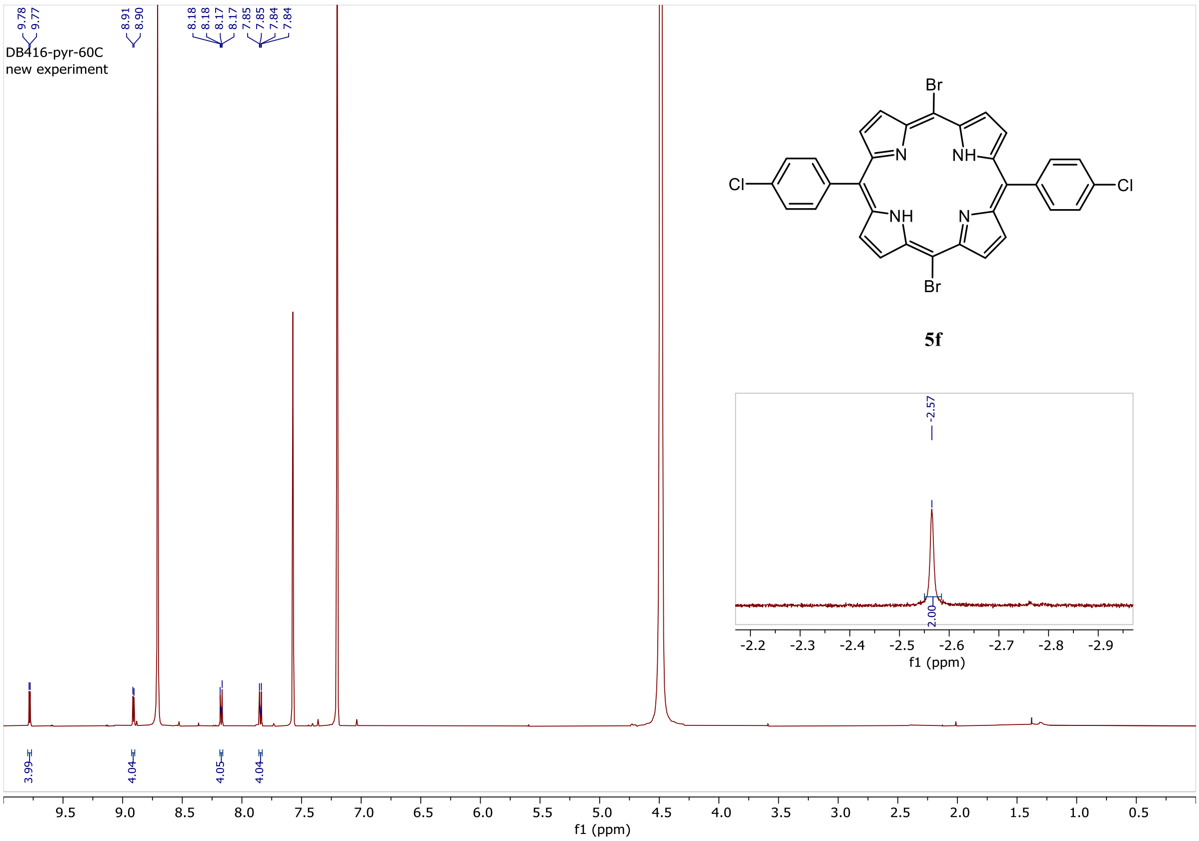

^{1}H NMR (600 MHz, pyridine-d$_5$) of 5g (for saturated solution at 100 °C)

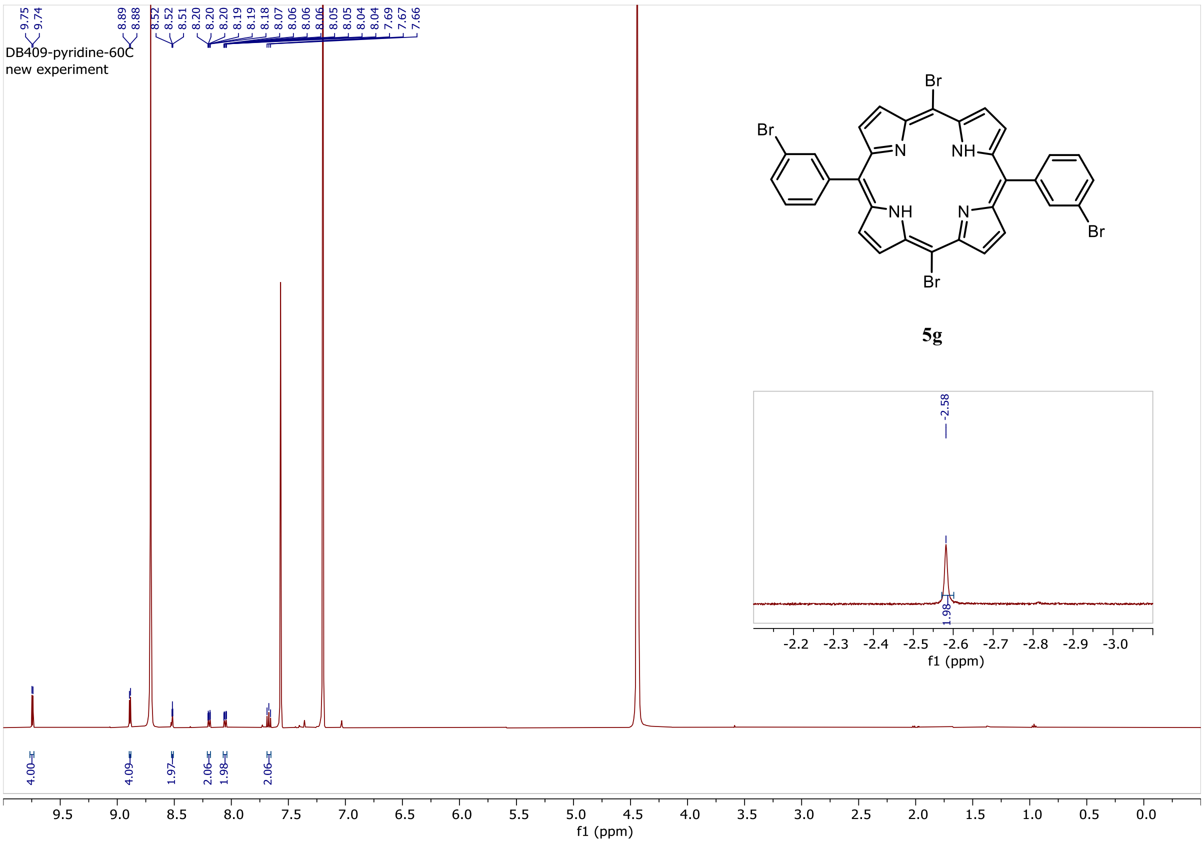

^{1}H NMR (600 MHz, $CDCl_3$) of 5h (for saturated solution)

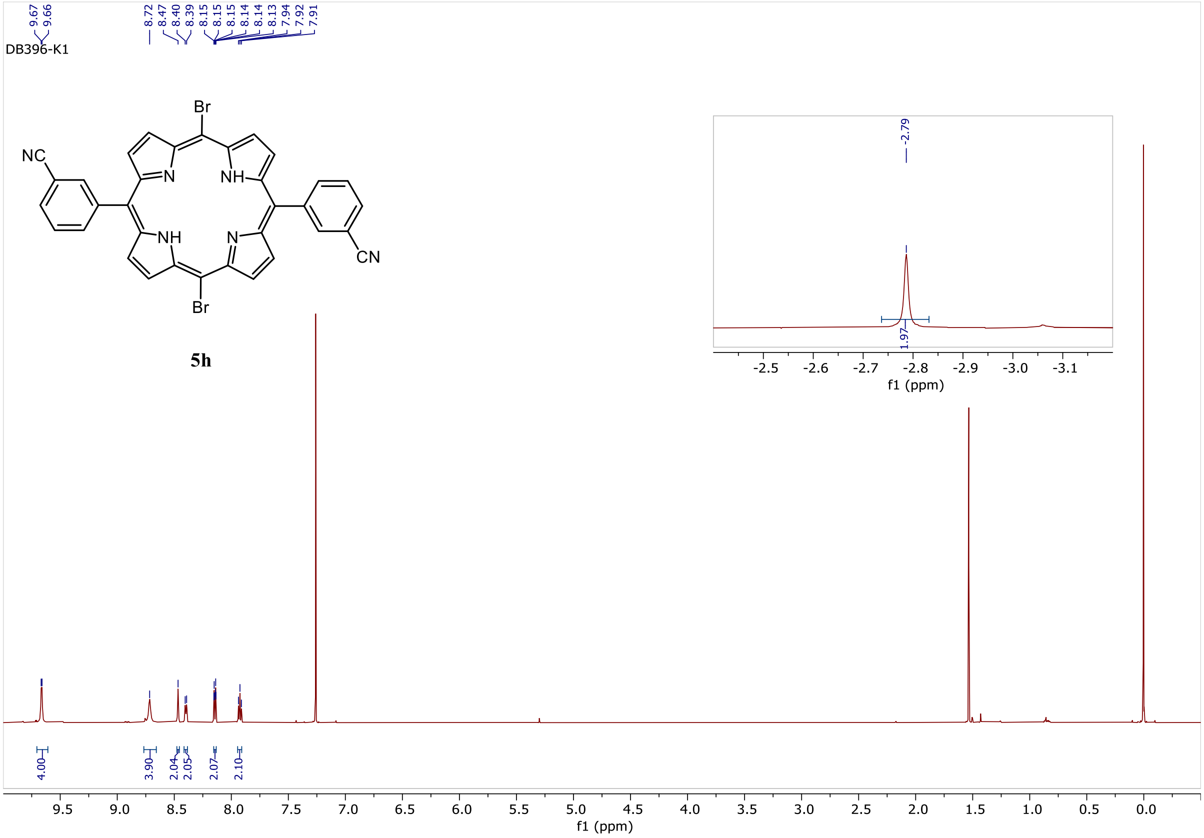

^{13}C NMR (151 MHz, $CDCl_3$) of 5h (for saturated solution)

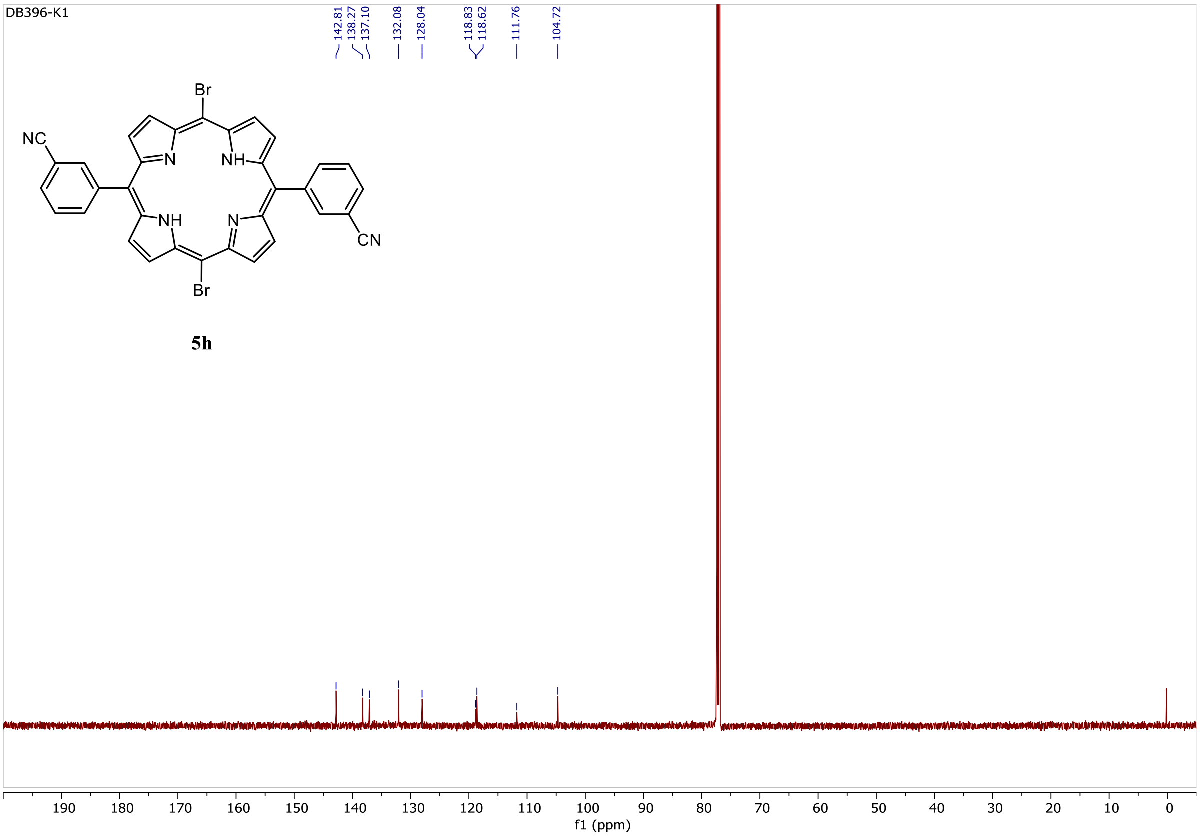

^{1}H NMR (600 MHz, $CDCl_3$) of 6a

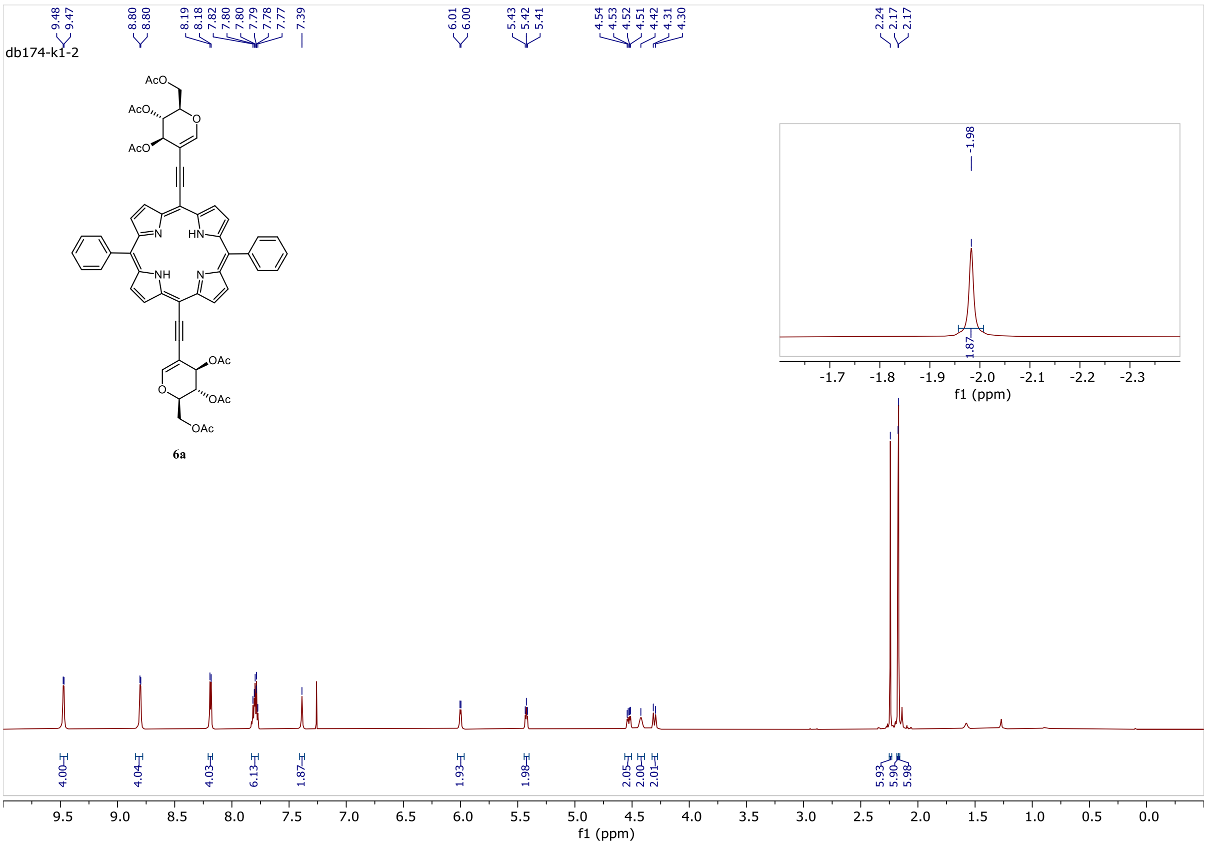

^{13}C NMR (151 MHz, $CDCl_3$) of 6a

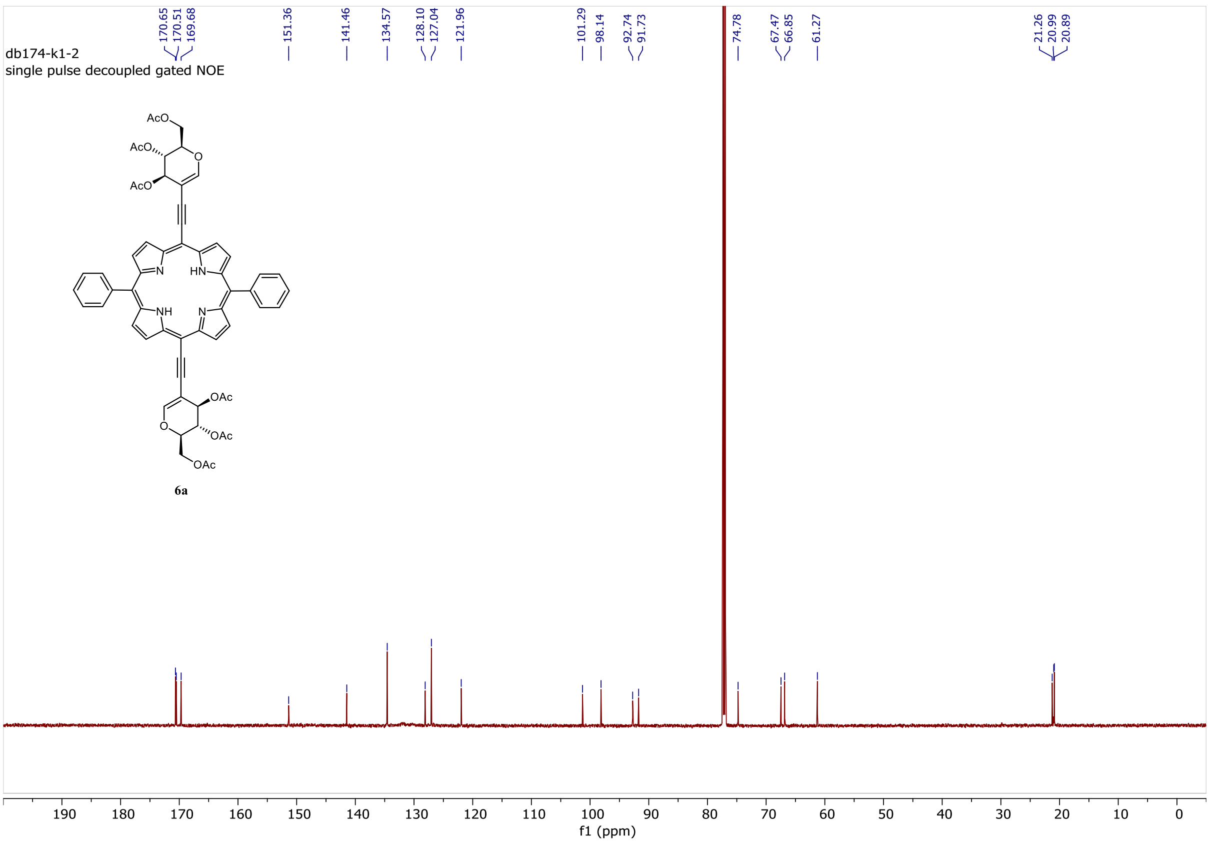

^{1}H NMR (600 MHz, $CDCl_3$) of 6b

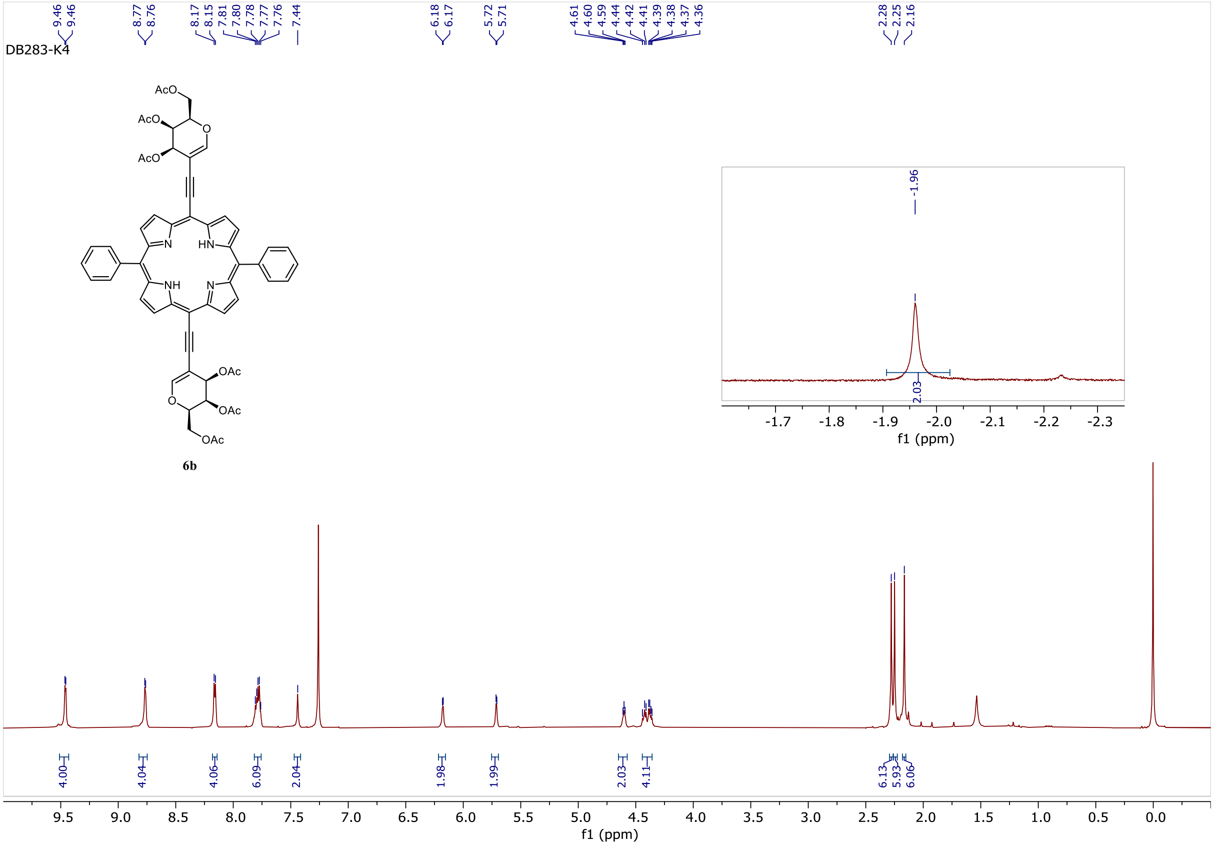

^{13}C NMR (151 MHz, $CDCl_3$) of 6b

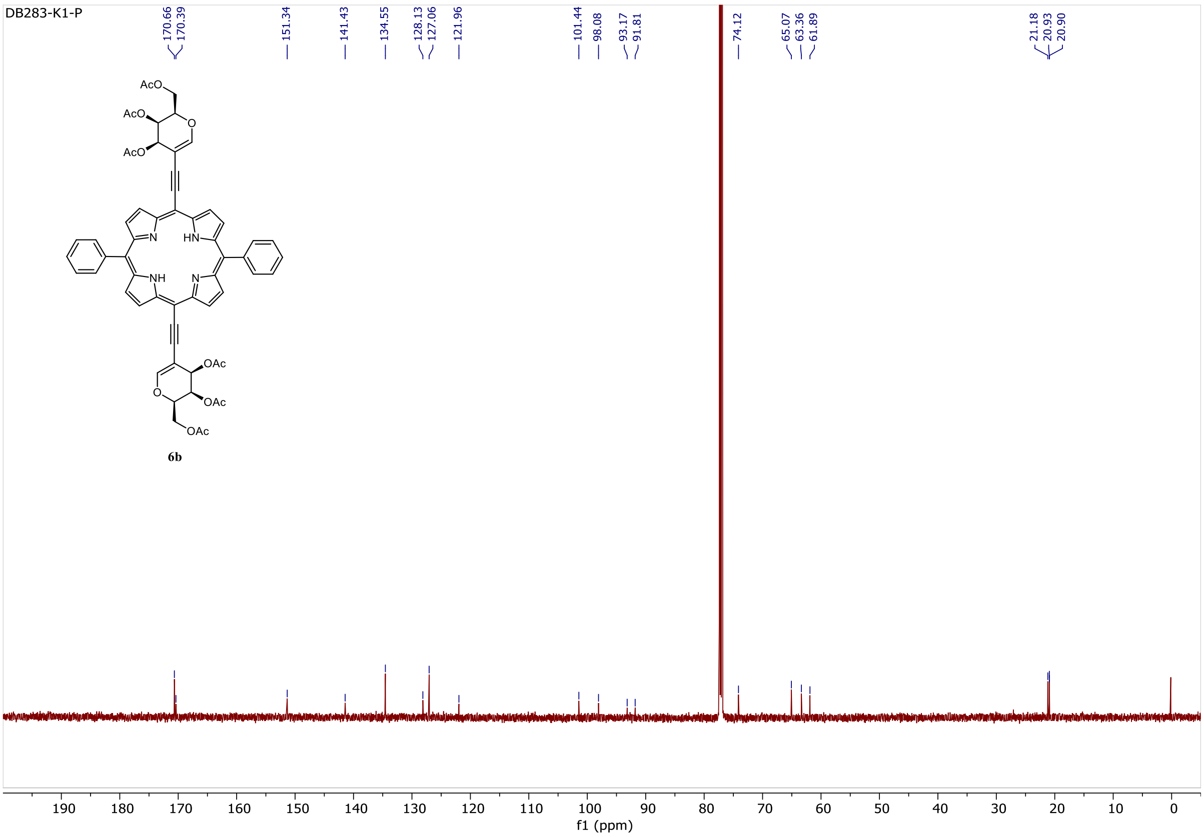

^{1}H NMR (600 MHz, $CDCl_3$) of 6c

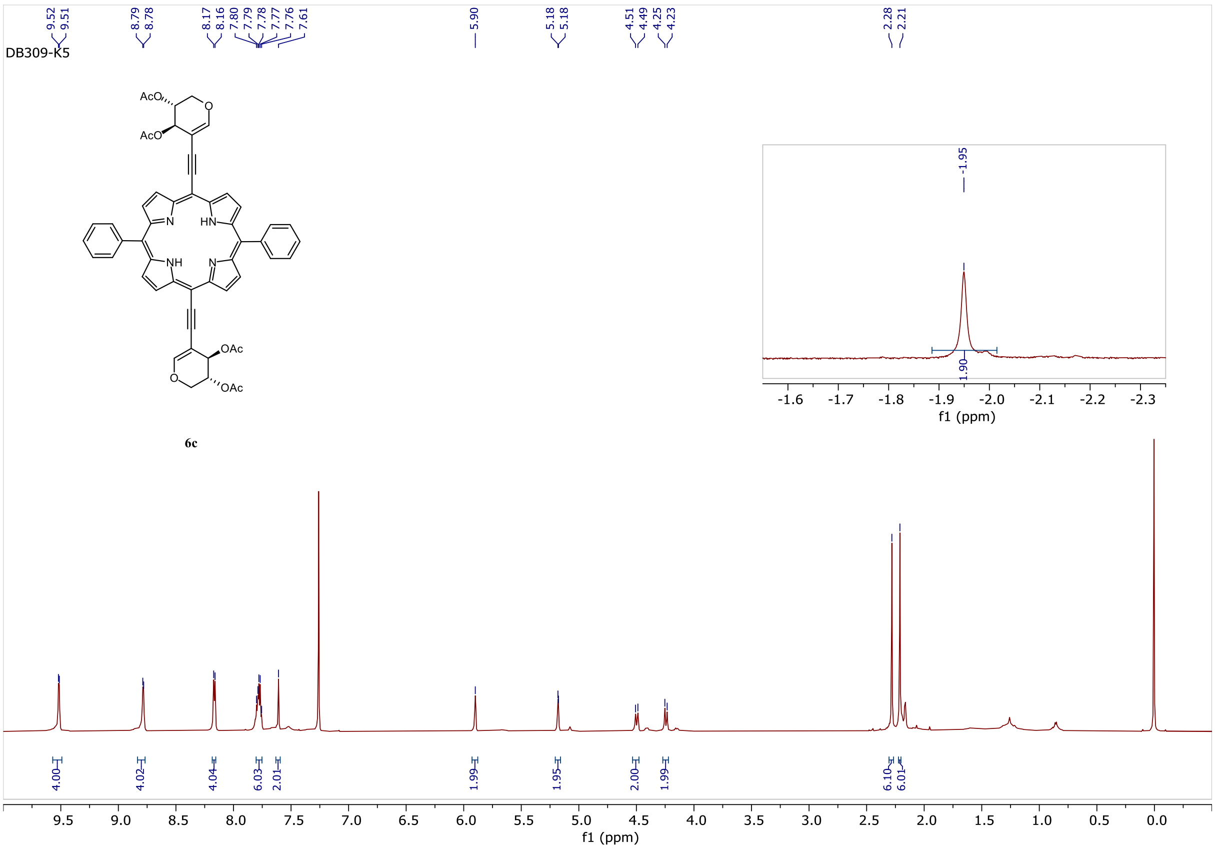

^{13}C NMR (151 MHz, $CDCl_3$) of 6c

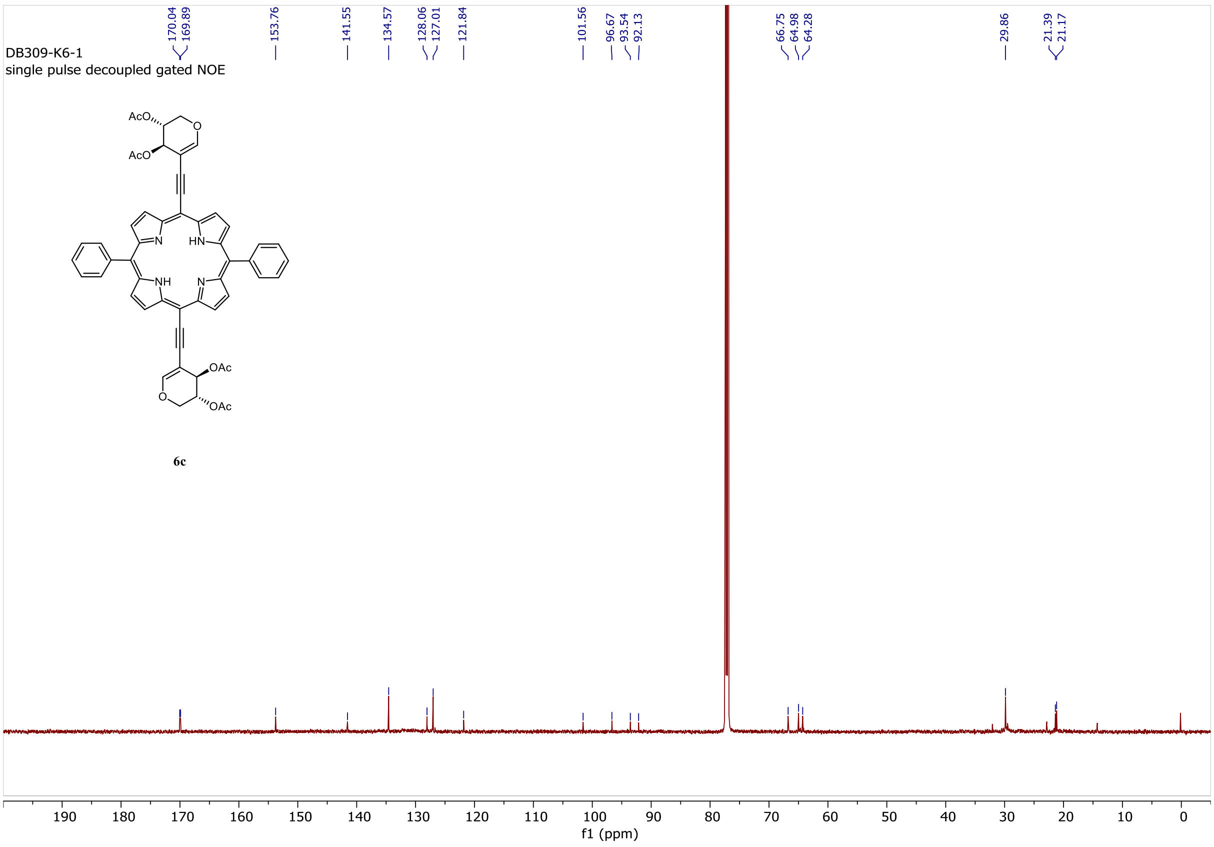

^{1}H NMR (600 MHz, $CDCl_3$) of 6d

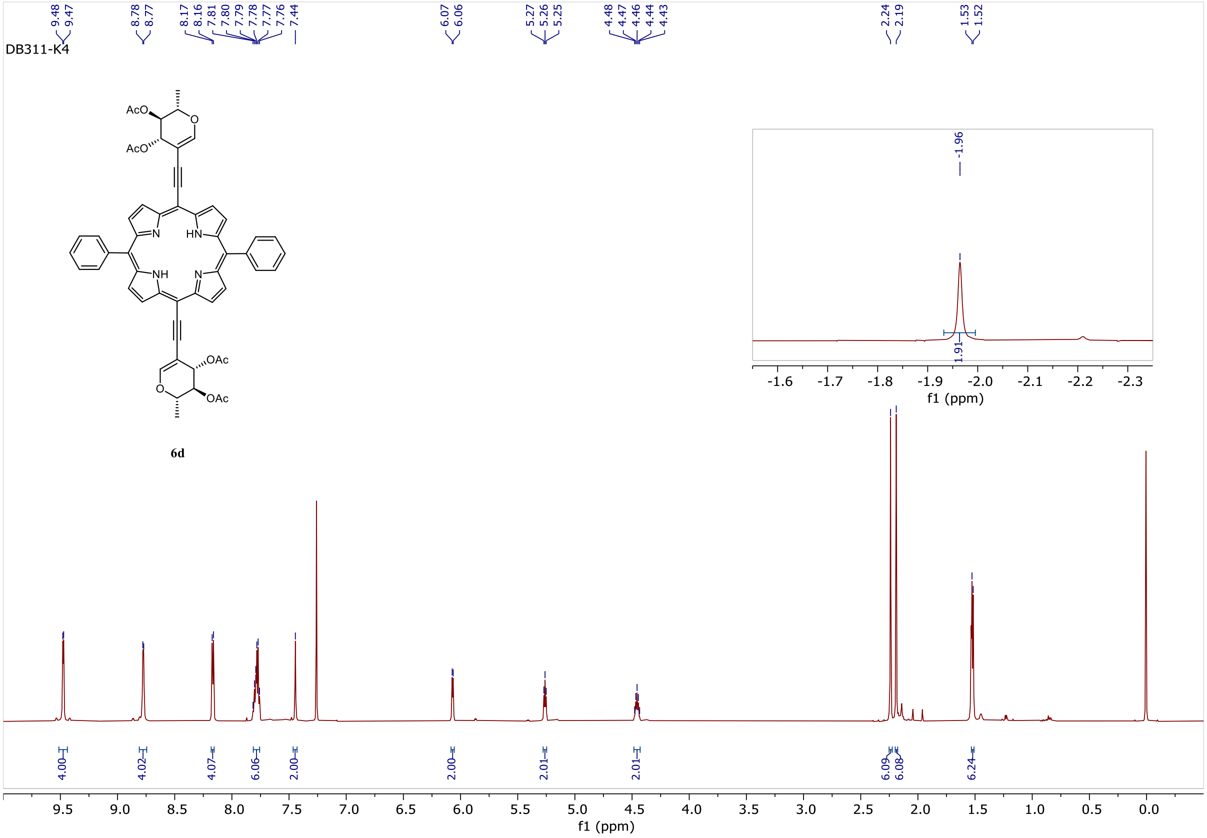

^{13}C NMR (151 MHz, $CDCl_3$) of 6d

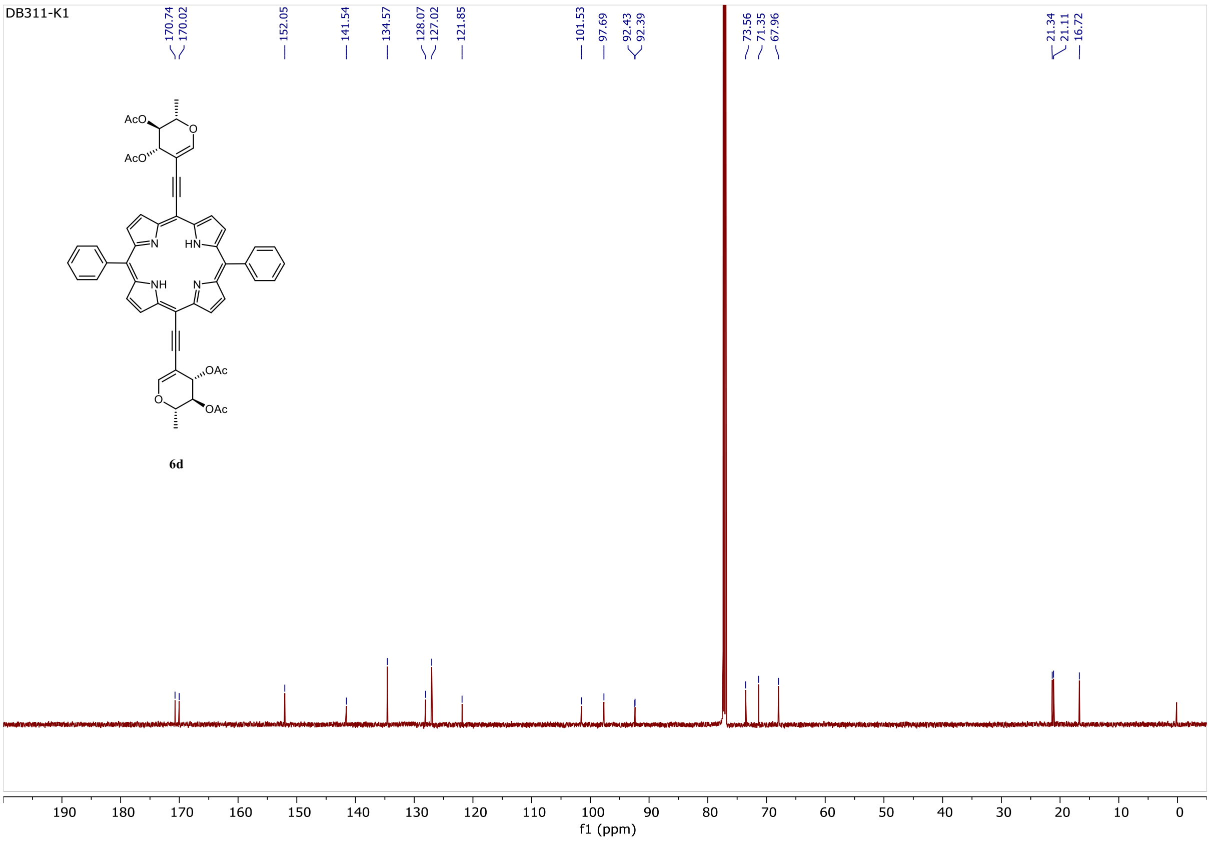

^{1}H NMR (600 MHz, $CDCl_3$) of 7b

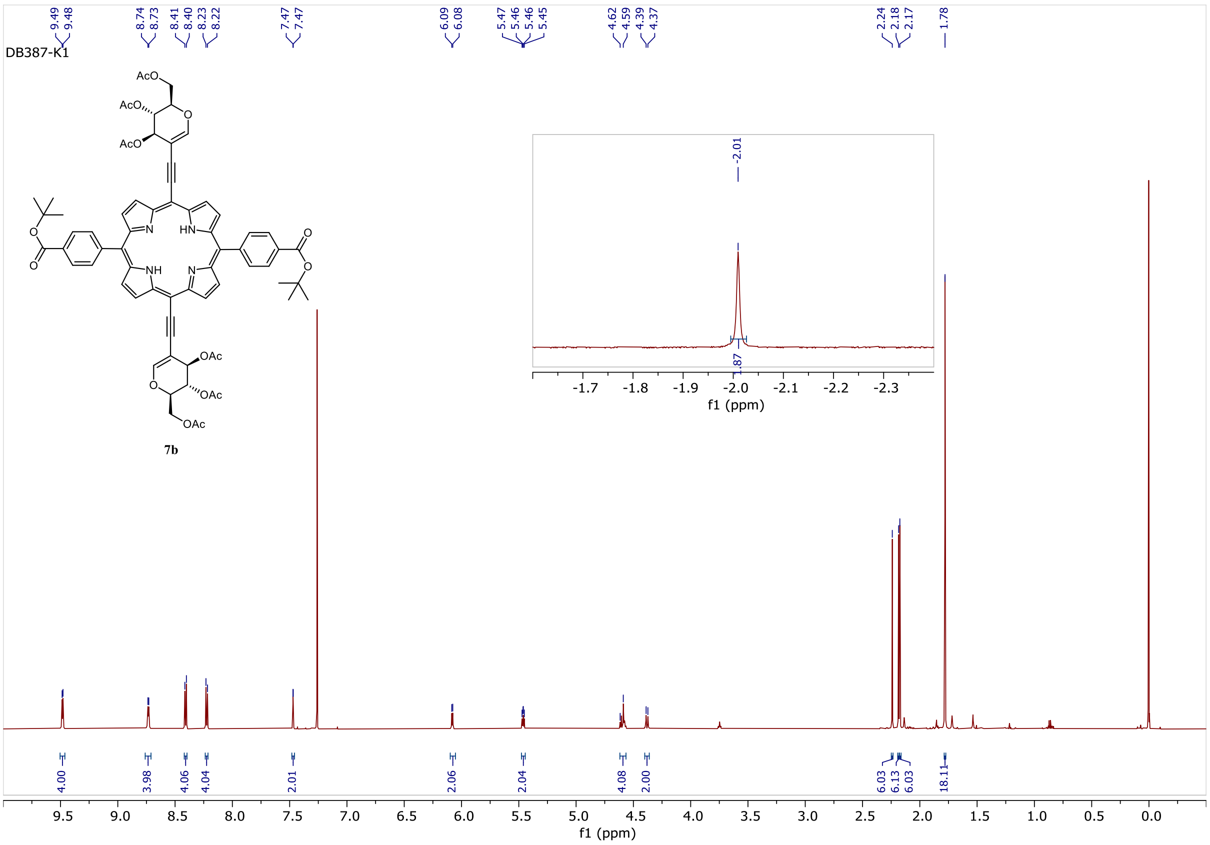

^{13}C NMR (151 MHz, $CDCl_3$) of 7b

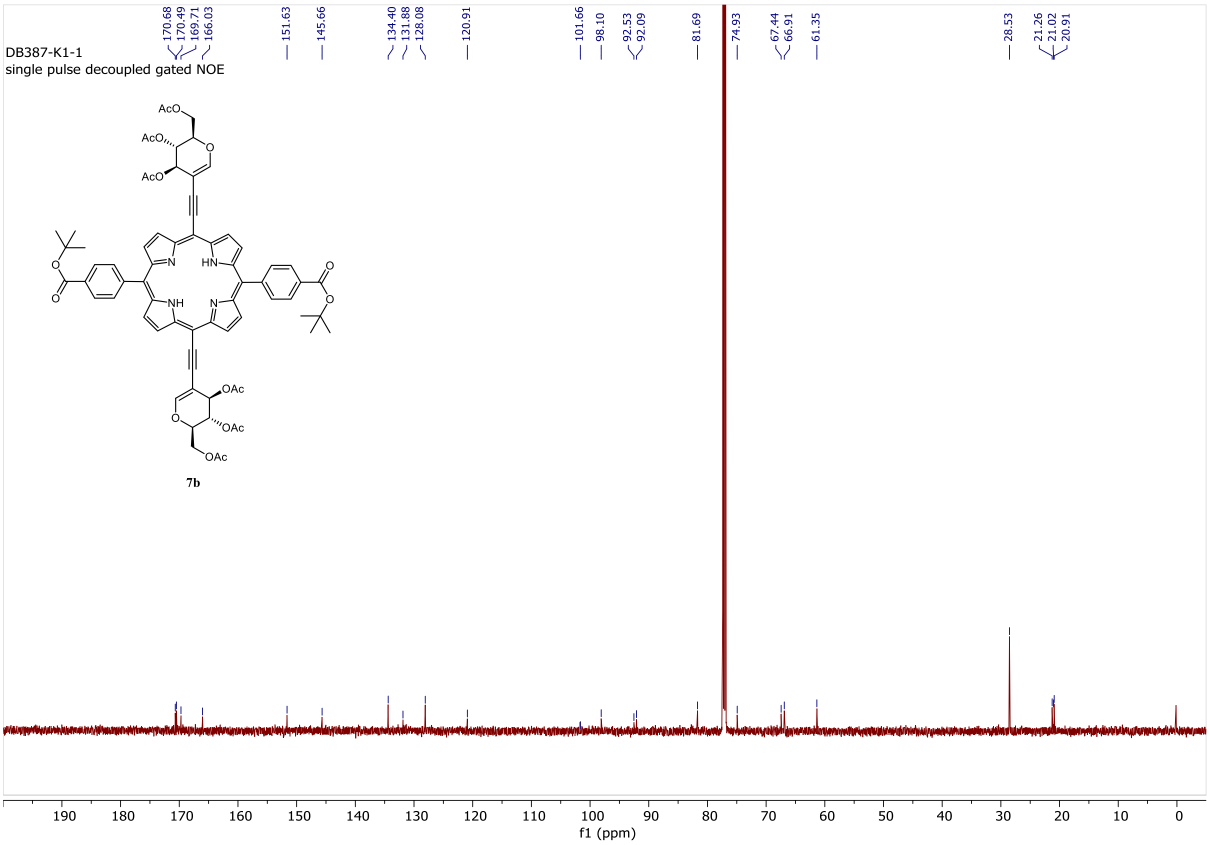

^{1}H NMR (600 MHz, $CDCl_3$) of 7c

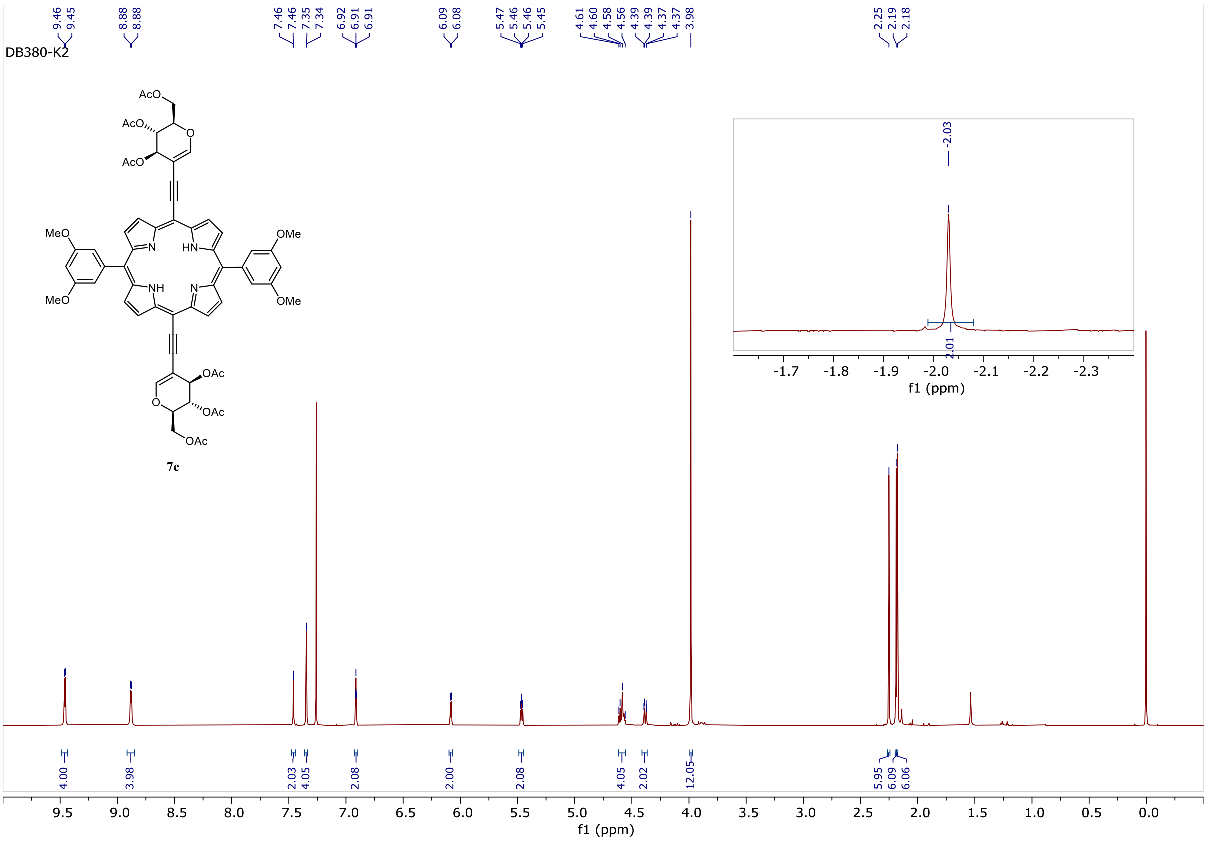

^{13}C NMR (151 MHz, $CDCl_3$) of 7c

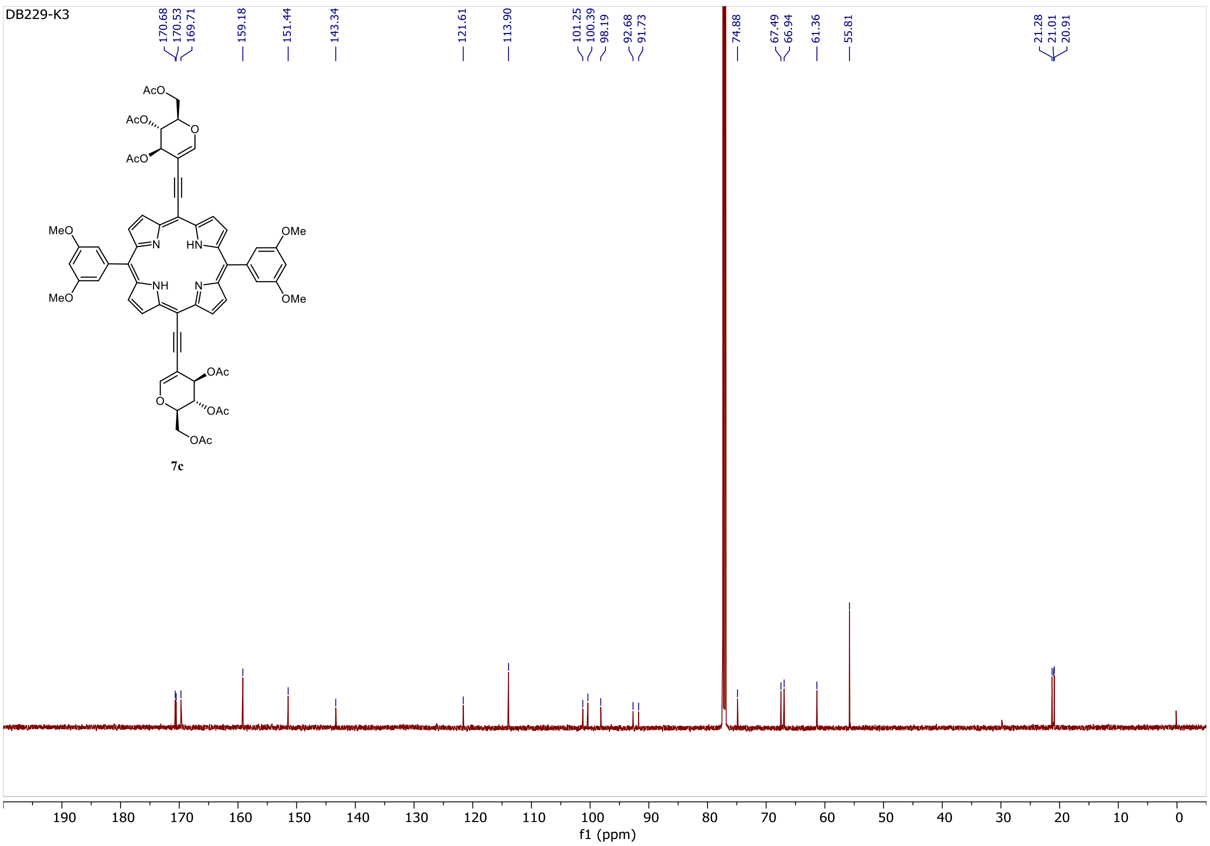

^{1}H NMR (600 MHz, $CDCl_3$) of 7d

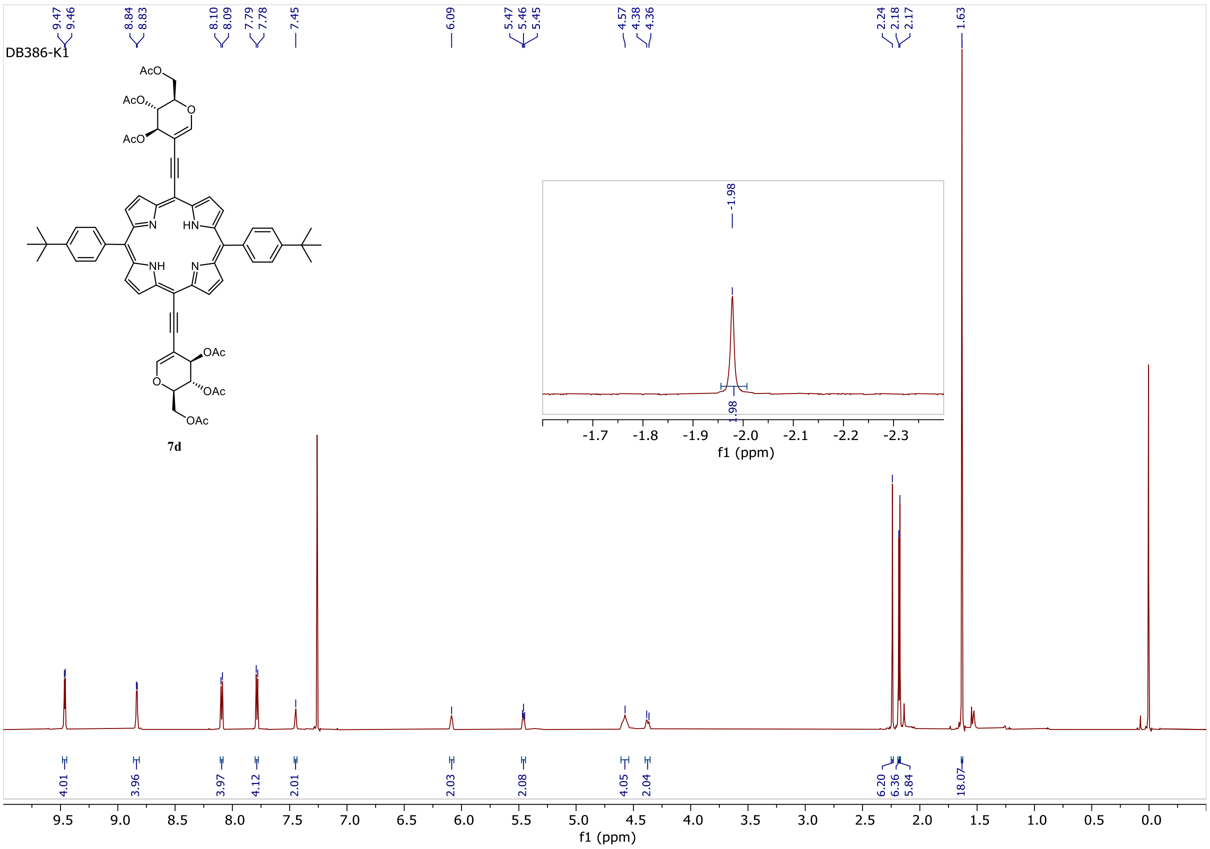

^{13}C NMR (151 MHz, $CDCl_3$) of 7d

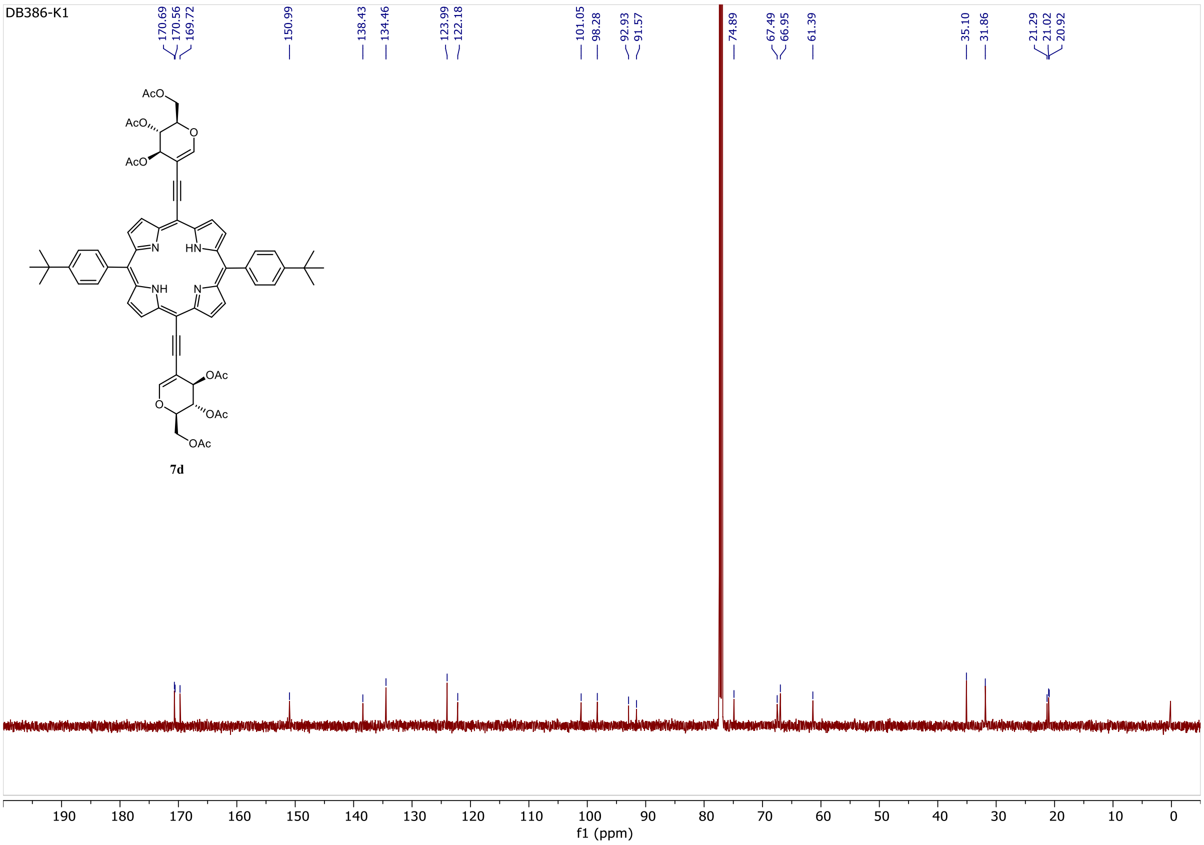

^{1}H NMR (600 MHz, $CDCl_3$) of 7e

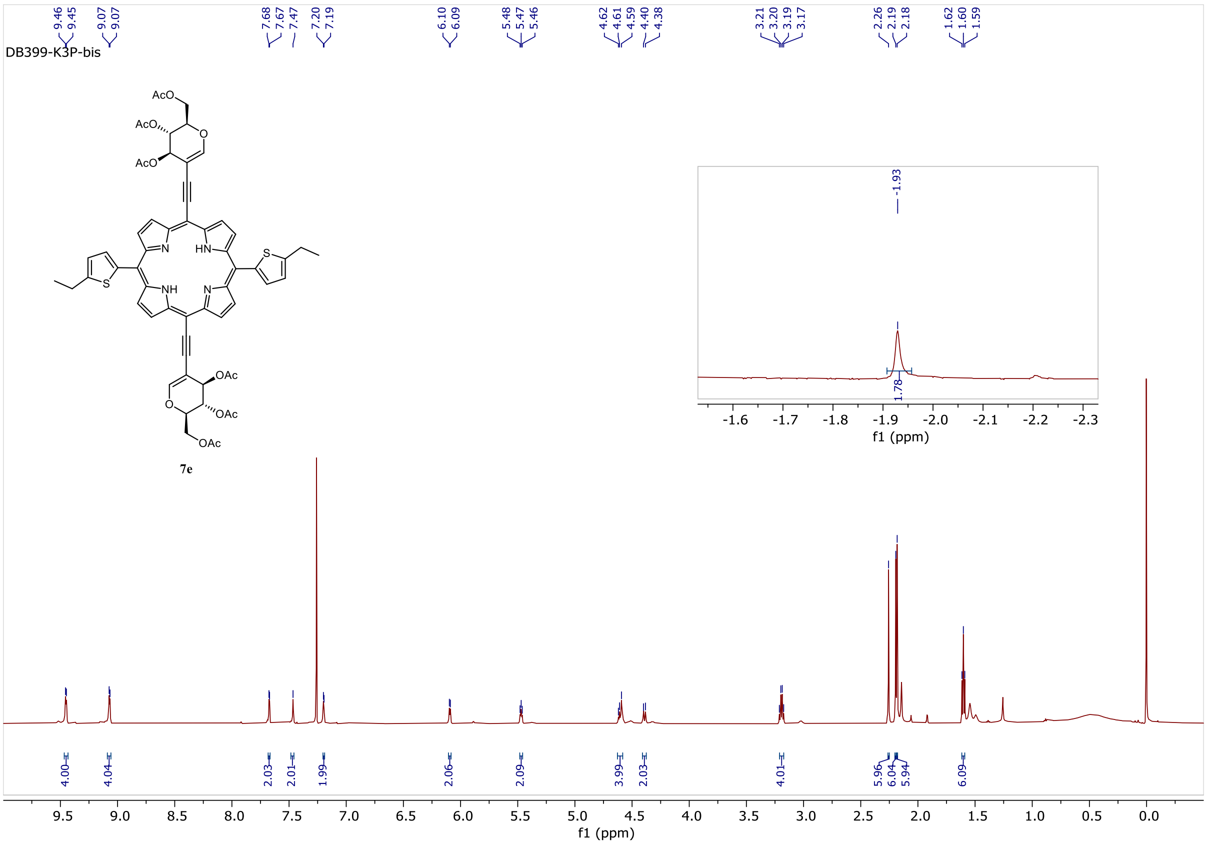

^{13}C NMR (151 MHz, $CDCl_3$) of 7e

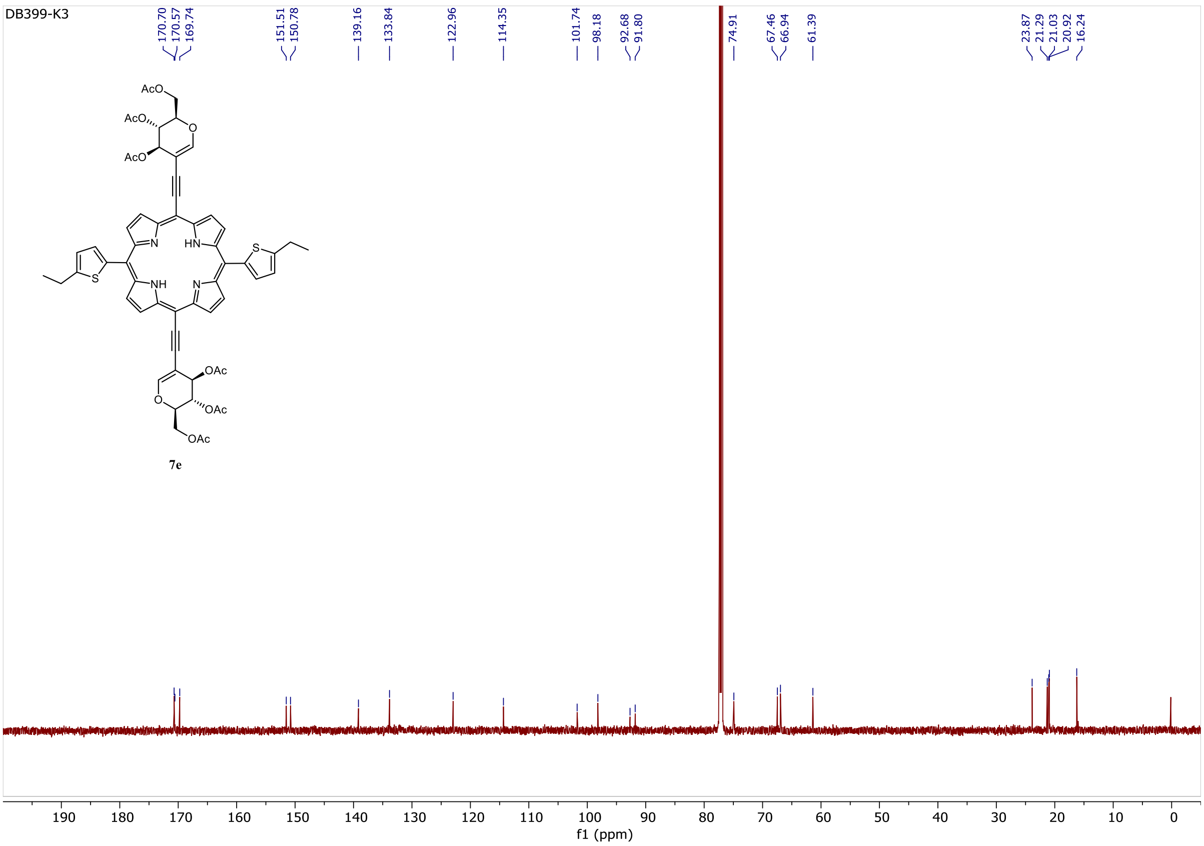

^{1}H NMR (600 MHz, $CDCl_3$) of 7f

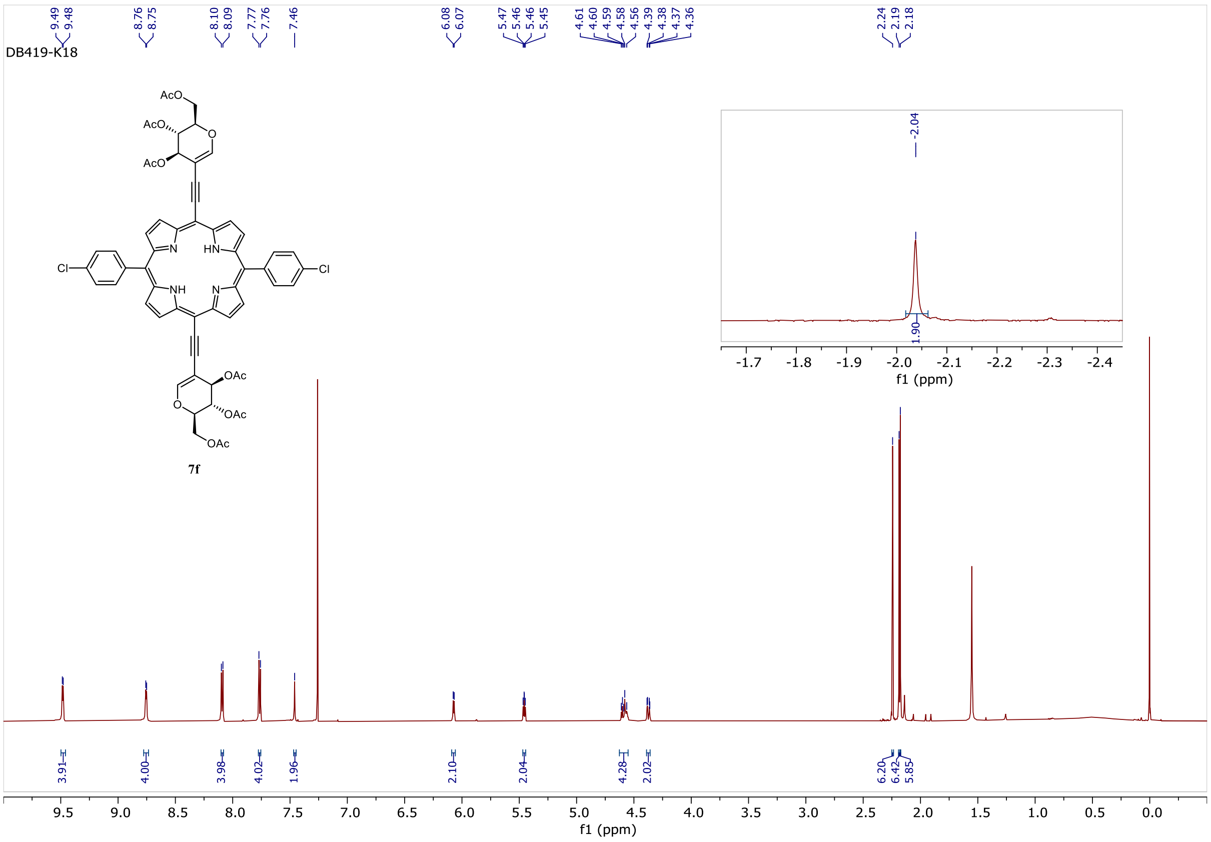

^{13}C NMR (151 MHz, $CDCl_3$) of 7f

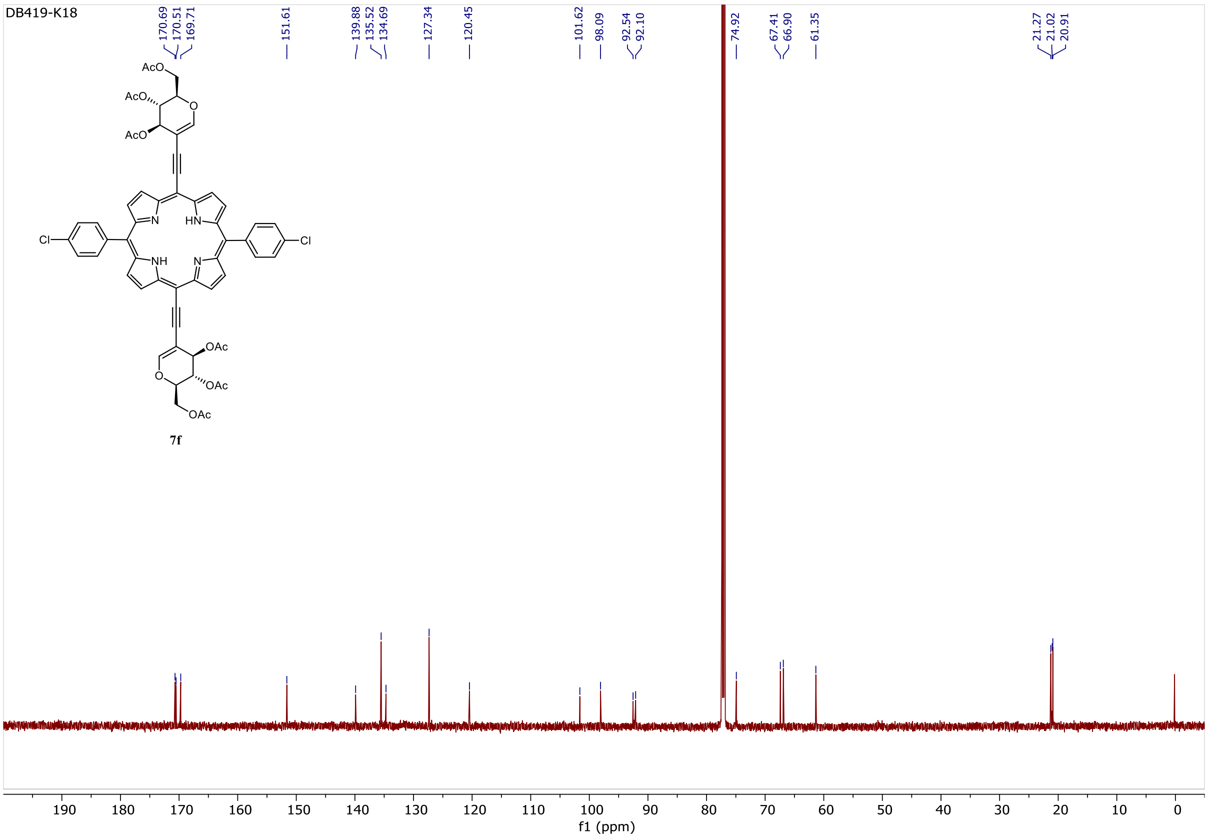

^{1}H NMR (600 MHz, $CDCl_3$) of 7g

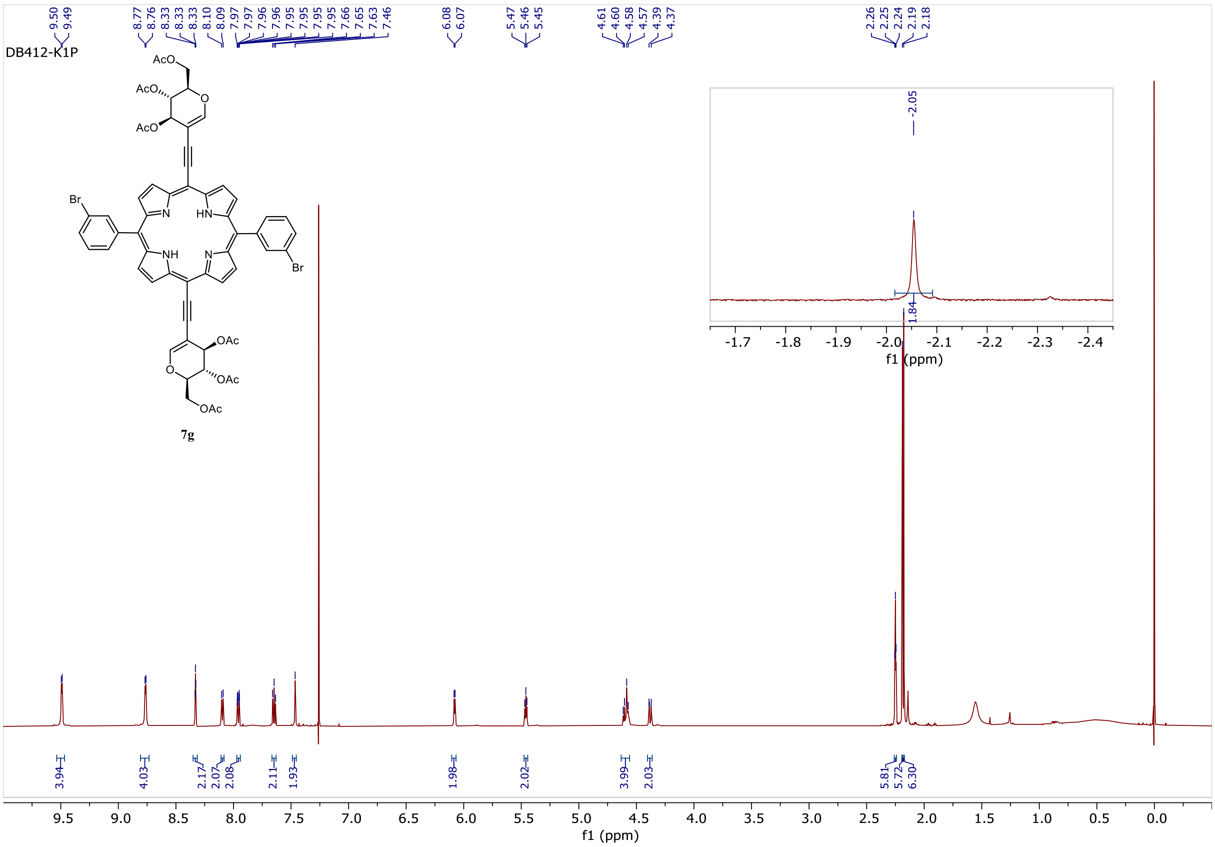

^{13}C NMR (151 MHz, $CDCl_3$) of 7g

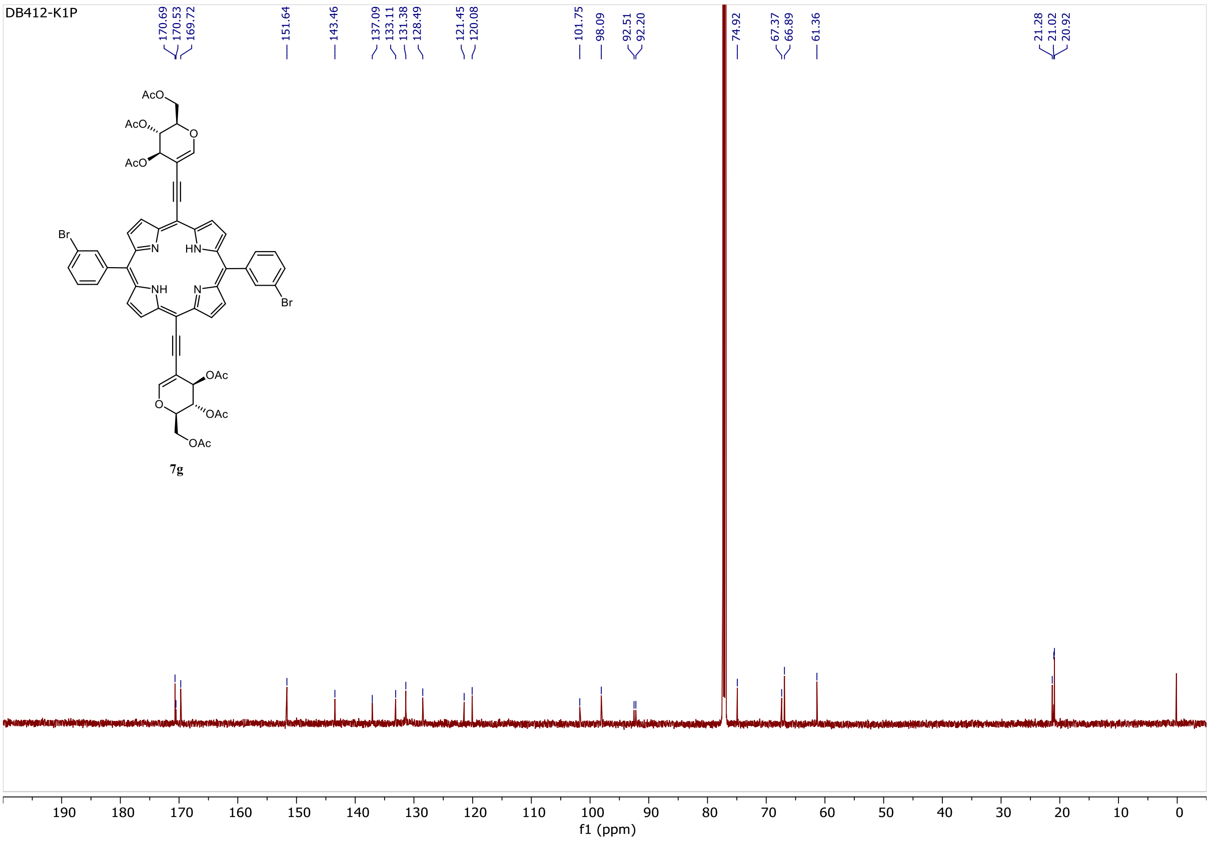

^{1}H NMR (600 MHz, $CDCl_3$) of 7h

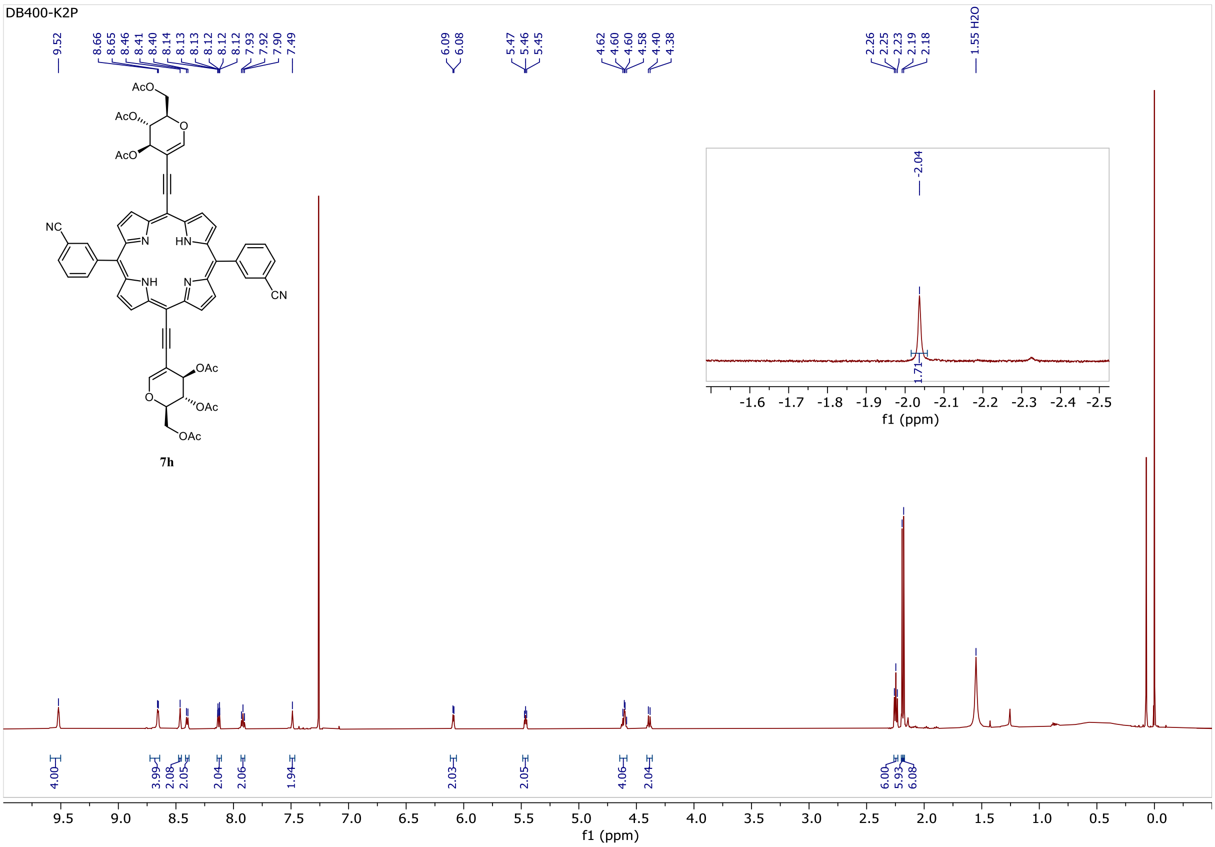

^{13}C NMR (151 MHz, $CDCl_3$) of 7h

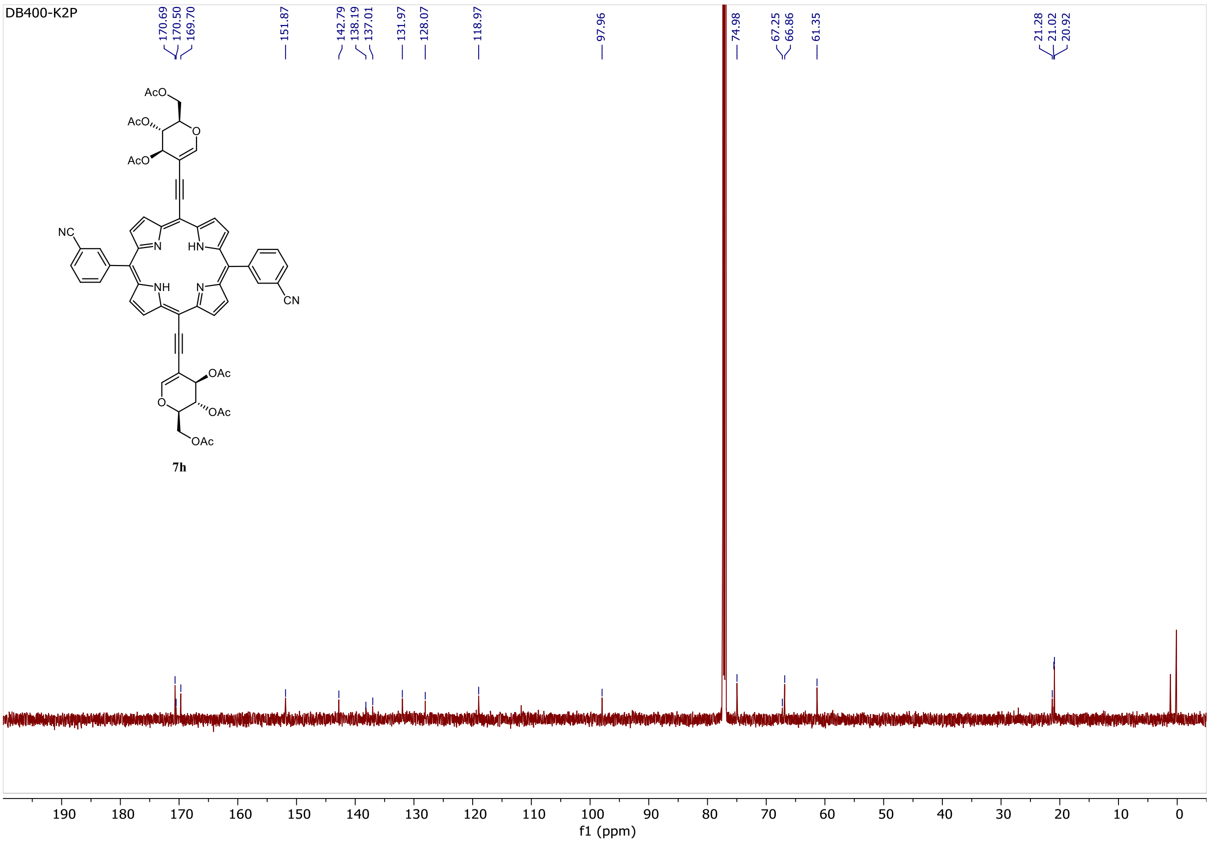

^{1}H NMR (600 MHz, DMSO-d$_6$) of 8a

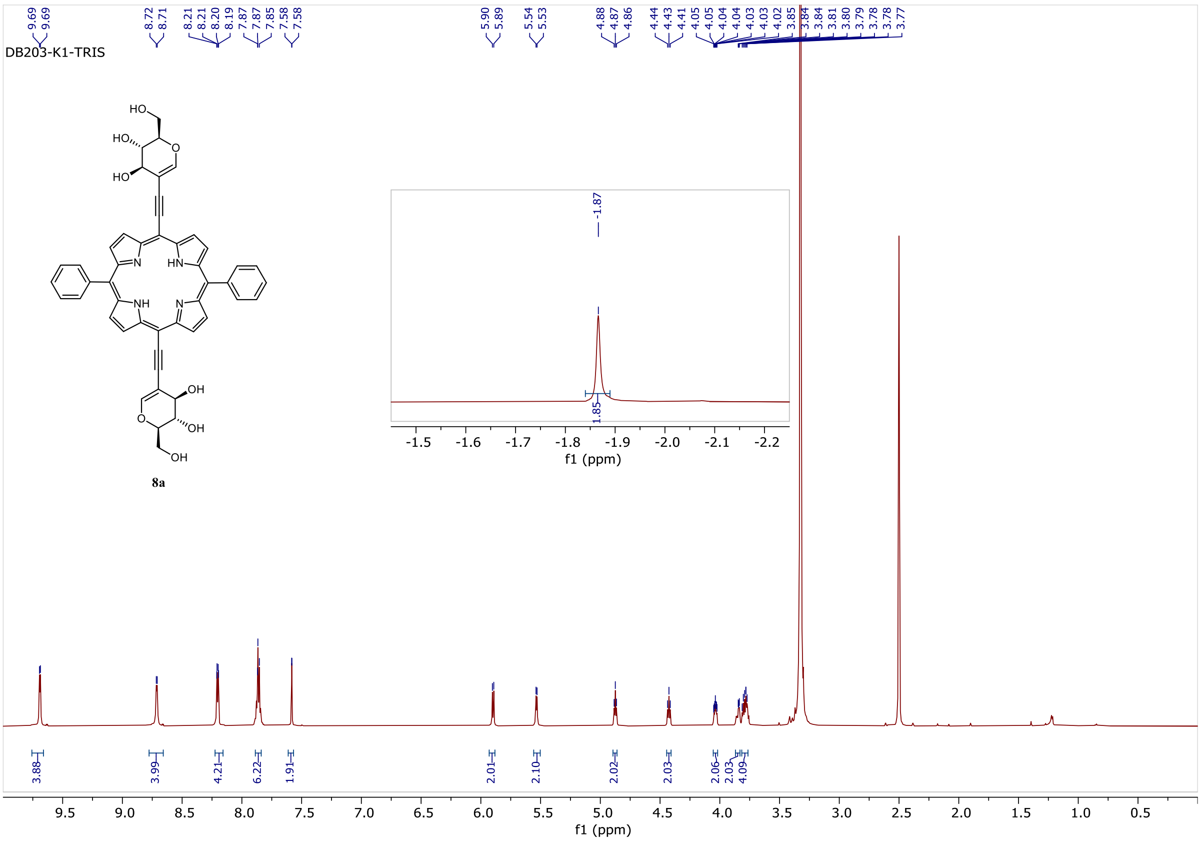

^{13}C NMR (151 MHz, DMSO-d$_6$) of 8a

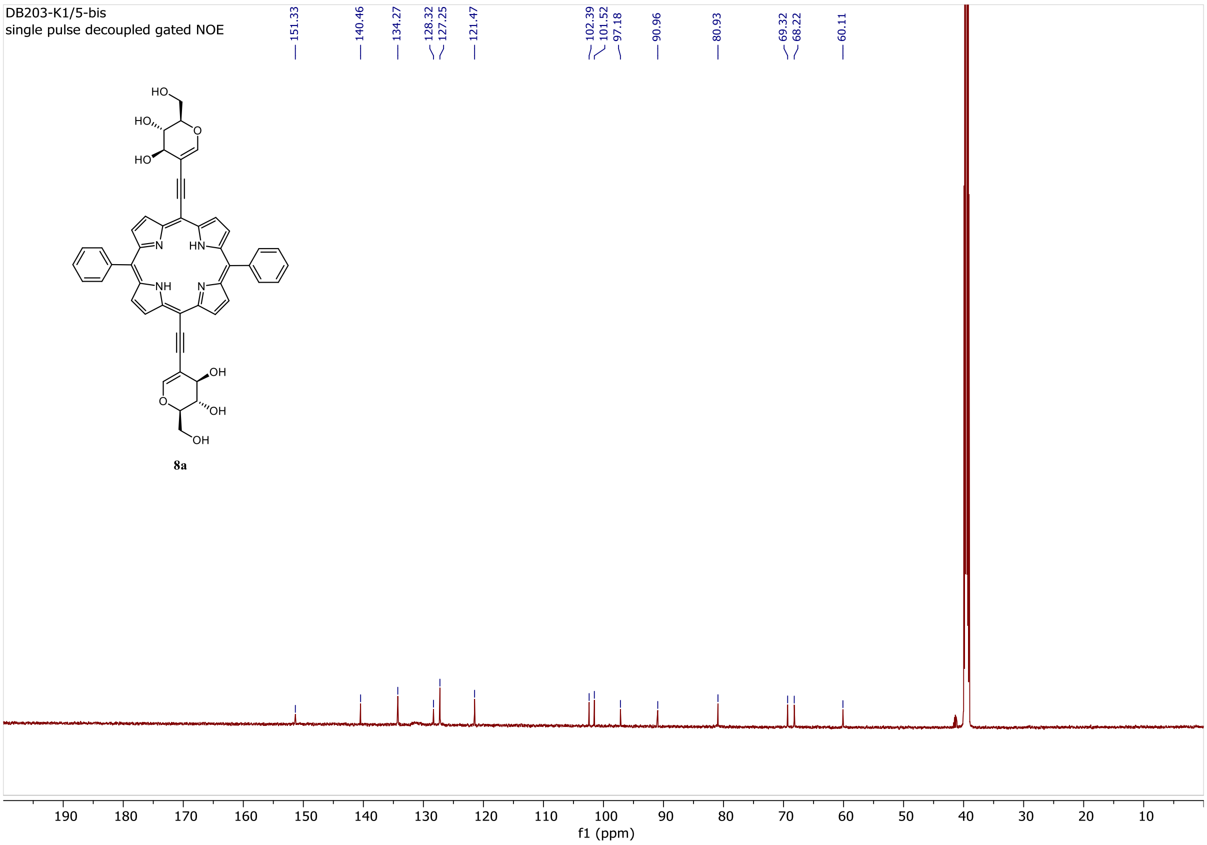

Supplement: Supplementary file 1 [file ol5c02038_si_001.pdf]
